# Multiple Low-Dose Radiation Prevents Type 2 Diabetes-Induced Renal Damage through Attenuation of Dyslipidemia and Insulin Resistance and Subsequent Renal Inflammation and Oxidative Stress

**DOI:** 10.1371/journal.pone.0092574

**Published:** 2014-03-20

**Authors:** Minglong Shao, Xuemian Lu, Weitao Cong, Xiao Xing, Yi Tan, Yunqian Li, Xiaokun Li, Litai Jin, Xiaojie Wang, Juancong Dong, Shunzi Jin, Chi Zhang, Lu Cai

**Affiliations:** 1 School of Public Health of Jilin University, Changchun, China; 2 Chinese-American Research Institute for Diabetic Complications, Wenzhou Medical University, Wenzhou, China; 3 Ruian Center of Chinese-American Research Institute for Diabetic Complications, the Third Affiliated Hospital of the Wenzhou Medical University, Wenzhou, China; 4 School of Pharmacy, Wenzhou Medical College, Wenzhou, China; 5 Changchun Institute for Food and Drug Control, Changchun, China; 6 Kosair Children’s Hospital Research Institute at the Department of Pediatrics, University of Louisville, Louisville, Kentucky, United States of America; 7 Department of Neurosurgery, the First Hospital of Jilin University, Changchun, China; Monash University, Australia

## Abstract

**Background:**

Dyslipidemia and lipotoxicity-induced insulin resistance, inflammation and oxidative stress are the key pathogeneses of renal damage in type 2 diabetes. Increasing evidence shows that whole-body low dose radiation (LDR) plays a critical role in attenuating insulin resistance, inflammation and oxidative stress.

**Objective:**

The aims of the present study were to investigate whether LDR can prevent type 2 diabetes-induced renal damage and the underlying mechanisms.

**Methods:**

Mice were fed with a high-fat diet (HFD, 40% of calories from fat) for 12 weeks to induce obesity followed by a single intraperitoneal injection of streptozotocin (STZ, 50 mg/kg) to develop a type 2 diabetic mouse model. The mice were exposed to LDR at different doses (25, 50 and 75 mGy) for 4 or 8 weeks along with HFD treatment. At each time-point, the kidney weight, renal function, blood glucose level and insulin resistance were examined. The pathological changes, renal lipid profiles, inflammation, oxidative stress and fibrosis were also measured.

**Results:**

HFD/STZ-induced type 2 diabetic mice exhibited severe pathological changes in the kidney and renal dysfunction. Exposure of the mice to LDR for 4 weeks, especially at 50 and 75 mGy, significantly improved lipid profiles, insulin sensitivity and protein kinase B activation, meanwhile, attenuated inflammation and oxidative stress in the diabetic kidney. The LDR-induced anti-oxidative effect was associated with up-regulation of renal nuclear factor E2-related factor-2 (Nrf-2) expression and function. However, the above beneficial effects were weakened once LDR treatment was extended to 8 weeks.

**Conclusion:**

These results suggest that LDR exposure significantly prevented type 2 diabetes-induced kidney injury characterized by renal dysfunction and pathological changes. The protective mechanisms of LDR are complicated but may be mainly attributed to the attenuation of dyslipidemia and the subsequent lipotoxicity-induced insulin resistance, inflammation and oxidative stress.

## Introduction

Diabetic nephropathy (DN) is one of the most severe complications of type 2 diabetes, which accounts for approximately 40% of all new cases who require renal replacement therapy [1], [2]. DN initiates with early-stage diabetic kidney disease (DKD) characterized by renal dysfunction, and finally leading to fibrosis and glomerulosclerosis [3]–[6].

Although the pathogeneses of kidney disease in type 2 diabetes are complicated, dyslipidemia and the subsequent lipotoxicity are regarded as critical inducers of renal damage under diabetic conditions [7]–[9]. Dyslipidemia is characterized by abnormal lipid profiles [10], [11], which contribute to progression of DKD [12]. Furthermore, dyslipidemia always leads to an imbalance between circulating and cytosolic fatty acid (FA) levels, which induce lipotoxicity [13].

Lipotoxicity underlies the insulin-deficient state, especially insulin resistance. Strong evidence showed that excessive FAs and their derivatives serve as signaling molecules that activate protein kinases [13]. These kinases can then impair insulin signaling, finally lead to insulin resistance [14], [15]. It has been reported that lipotoxicity-induced insulin resistance plays a critical role in type 2 diabetes induced renal damage [16]. Moreover, insulin resistance directly induces relaxation of the afferent arteriole, resulting in glomerular hyperfiltration, angiogenesis and mesangial cell proliferation, then contributing to the development of DN [17], [18]. Lipotoxicity also induces oxidative stress by production of reactive oxygen species (ROS) which causes damage to the glomeruli and glomerular glycocalyx and increases the permeability of the glomerular filtration barrier, which may contribute to the progression of DKD [19], [20].

Inflammation is another key consequence of lipotoxicity in type 2 diabetes, which also strongly exacerbates kidney injury in type 2 diabetes characterized by increasing the release of multiple inflammatory factors [21], [22]. Therefore, attenuation of dyslipidemia and the subsequent lipotoxicity might be a novel therapeutic strategy for DKD in type 2 diabetes.

Various therapeutic approaches have been investigated, but most if not all drugs need to be excreted via the kidney, which will further increase the renal working load in diabetic patients [23]. Based on this concern, low-dose ionizing radiation (LDR) as a non-invasive approach has been investigated to prevent chronic renal diseases [24], [25]. Our previous study also determined that LDR significantly prevented renal damage in type 1 diabetic mice [23]. We also found that LDR prevented type 1 diabetes-induced renal oxidative stress via up-regulation of multiple anti-oxidants levels [26]. However, whether LDR can induce similarly beneficial effects on renal damage in type 2 diabetes is still unknown. To this end, we used the strategy of high-fat diet (HFD) feeding plus streptozotocin (STZ) injection to develop the type 2 diabetic mice models [27], [28]. Then we investigated whether exposure to LDR could attenuate dyslipidemia, inflammation and oxidative stress and enhanced insulin sensitivity in type 2 diabetes.

## Materials and Methods

### Ethics Statement

The protocol was approved by the Committee on the Ethics of Animal Experiments of Jilin University, Jilin, China (Permit Number: 2007-0011). All surgery was performed under sodium pentobarbital anesthesia and all efforts were made to minimize suffering.

### Animals and Induction of Type 2 Diabetes

Male C57BL/6J mice, 8 weeks old (18–22 g of body weight), were purchased from the experimental Animal Center of Beijing University of Medical Science (Beijing, China) and allowed to acclimate for 2 weeks. All mice were housed in the animal facility of the Jilin University Animal Center at 22°C with a 12:12-h light-dark cycle and free access to rodent chow and tap water.

In order to establish type 2 diabetic model, the combined strategy of a HFD (Shanghai SLAC laboratory Animal Co., Ltd., 40% of calories from fat) and STZ (Sigma Chemical, St. Louis, MO) treatment was applied in our study. The mice were fed with HFD for 12 weeks to induce obesity, characterized by abnormal glucose tolerance and insulin resistance. The age-matched nondiabetic mice were fed with a standard diet (SD, Shanghai SLAC laboratory Animal Co., Ltd., 10% of calories from fat). Then, the mice fed with HFD were intraperitoneally given a single injection of STZ at 50 mg/kg body weight. In addition, the mice fed with SD were given an injection of equivalent volume of citrate buffer. Blood glucose level was examined using a FreeStyle glucose meter (Abbott Diabetes Care, Alameda, CA) 3 days after the injection of STZ. Mice were considered diabetic when blood glucose exceeded 12 mmol/L. Next, mice were continued to feed with the corresponding diet for another 4 or 8 weeks.

### Whole-body Low dose Rate X-ray Radiation

A 180-kVp X-ray generator (Model XSZ-Z20/20, China) was utilized to deliver radiation at dose rate of 12.5 mGy/min (120 kv, 13 mA). LDR was given to mice over whole body every other day at a dose of 25, 50 or 75 mGy for 4 or 8 weeks, respectively. Mice from both the diabetic and age-matched nondiabetic groups were randomly divided into the following groups: control group (Con), control plus 50 mGy group (Con/50 mGy), diabetes mellitus group (DM) and DM plus LDR at 25, 50 or 75 mGy (DM/25 mGy, DM/50 mGy, DM/75 mGy). The body weight, kidney weight and tibia length of the mice in all groups were examined at each time point.

### Glucose Tolerance Test and Insulin Tolerance Test

To assess glucose tolerance, mice were intraperitoneally injected with D-glucose (1.5 g/kg) after an overnight fasting (12 h) with free access to water, and venous blood was collected 30 min before (time 0) and after injection at 0, 15, 30, 60 and 120 min from the tail of each mouse, and glucose was measured using a FreeStyle glucose meter. To assess insulin tolerance, a single dose of Novolin R regular insulin (Novo Nordisk A/S, Denmark) (0.5 units/kg or 1 unit/kg) was intraperitoneally administered to the mice after fasting for 4 h with free access to water, and the blood glucose level was measured as described above.

### Measurements of Urine and Blood Metabolites

Twenty-four-hour urine collections were obtained by placing the mice in metabolic cages during the final 3 days before the mice were sacrificed. Urinary protein, urinary albumin and urinary creatinine were measured using enzyme-linked immunosorbent assay (ELISA) kits purchased from R&D (Itasea, MN) according to the manufacturer’s instructions. Briefly, the kits assayed mouse urine protein or albumin and creatinine level in the sample, using purified mouse urinary protein, urinary albumin and urinary creatinine antibody to coat a 96-well plate. Different concentrations of standards, matrix blank and testing samples were added to wells after adding the specified volume of diluents. Followed by 30 min incubation at 37°C and washing, a horseradish peroxidase (HRP) conjugate reagent was added to the wells, except blank wells. After incubation and then washing, a peroxidase substrate-tetramethylbenzidine (TMB) was added to the wells and incubated for 10 min. Then stop solution was added and absorbance was measured at 450 nm within 10 min with a reference wavelength of 650 nm by an ELISA Reader (Molecular Devices MAX 190, USA). The concentration of target factors in the sample was calculated using a corresponding standard plot of the optical density vs. the concentration of the standards.

Blood samples were taken from the retro-orbital venous plexus and allowed to clot (kept still) at room temperature for 30 min. Serums were collected by centrifugation at 2,000×*g* for 20 min at 4°C. Serum triglycerides (TG), total cholesterol (CHO), high-density lipoprotein (HDL)-cholesterol and low-density lipoprotein (LDL)-cholesterol levels were determined using an Olympus Au800 automatic biochemical analyzer (Olympus, Japan) at the Maternal and Child Health Hospital in Changchun, China.

### Quantification of Renal Lipid and Lipid Peroxidation

For the measurement of TG and free fatty acid (FFA) content, 50–80 mg of renal tissue from each mouse was homogenized in 250 µL of buffer containing 150 mM NaCl and 10 mM Tris (pH 7.5), and extracted with 200 µL of methanol and 400 µL of chloroform. The chloroform layer containing TG was evaporated overnight and resuspended in 70% ethanol; TG and FFA content was measured using a commercial ELISA kit (R&D) at 450 nm and expressed relative to the protein content. To determine malondialdehyde (MDA) content, kidney tissues were homogenized in ice-cold 0.05 mM phosphate-buffered saline (PBS; pH 7.4, 1: 10 wt/vol) and centrifuged at 15,000×*g* for 15 min before the supernatants were collected. All these steps were performed at 4°C. The supernatants were then used for measurement of MDA according to the manufacturer’s instructions (R&D). The protein levels of the samples were measured using the Coomassie Brilliant Blue G-250 method, with bovine serum albumin as the standard. The data were expressed as milligrams per gram of tissue or nanomoles per milligram of protein.

### Total Protein Extraction and Western Blotting

Western blotting assays were performed as described previously [29]. Briefly, renal tissues were homogenized in lysis buffer. Proteins were collected by centrifugation at 12,000×*g* and 4°C. The total protein sample was run on 10.6% SDS-polyacrylamide gel electrophoresis, transferred onto nitrocellulose membranes and incubated in blocking buffer (5% milk and 0.5% bovine serum albumin) for 1 h at room temperature and then incubated overnight at 4°C with following primary antibodies: nuclear factor E2-related factor-2 (Nrf-2, 1: 1000), superoxide dismutase-1 (SOD-1, 1: 2000), NAD(P)H:quinone oxidoreductase-1 (NQO-1, 1: 1000), heme oxygenase-1 (HO-1, 1: 2000), 3-nitrotyrosine (3-NT, 1: 1000), intercellular adhesion molecule-1 (ICAM-1, 1: 2000), plasminogen activator inhibitor-1 (PAI-1, 1: 2000), tumor necrosis factor α (TNF-α, 1: 1000), which were purchased from Abcam (Cambridge, MA); total protein kinase B (*t*-Akt, 1: 2000), phosphorylated-Akt (*p*-Akt, 1: 1000) and β-actin (1: 2000), which were purchased from CST (Danvers, MA). After three washes with tris-buffered saline containing 0.05% Tween 20 (TBST), membranes were incubated with secondary horseradish peroxidase-conjugated antibody for 1 h at room temperature. Antigen-antibody complexes were then developed with an ECL kit (Amersham, Piscataway, NJ). The intensity of protein bands on blots were quantified by using Quantity one (Version 4.6.2, Bio-Rad, USA).

### RNA Isolation and Real-time Quantitative Polymerase Chain Reaction (RT-PCR)

Total RNA was isolated from kidney tissues using TRIzol reagent according to the manufacturer’s protocol (Invitrogen, Carlsbad, CA) and was quantified with a photometer Nanodrop 2000 (Thermo Scientific, San Jose, CA). The RNA samples were reverse transcribed into cDNA using a High-Capacity cDNA Reverse Transcription Kit (PE Applied Biosystems, Foster City, CA). The following primers were used for RT-PCR. ICAM-1: forward, 5′-GTGATGGCAGCCTCTTATGT-3′; and reverse, 5′-GGGCTTGTCCCTTGAGT TT-3′; PAI-1: forward, 5′-CTCCACAGCCTTTGTCATCT-3′; and reverse, 5′-ATTGTCTC TGTCGGGTTGTG-3′; TNF-α: forward, 5′-CTACCTTGTTGCCTCCTCTTT-3′; and reverse, 5′-GAGCAGAGGTTCAGTGATGTAG-3′; Nrf-2: forward, 5′-CTCCGTGGAGTCTTCCA TTTAC-3′; and reverse, 5′-GCACTATCTAGCTCCTCCATTTC-3′; SOD-1: forward, 5′-GGCAAAGGTGGAAATGAAGAAA-3′; and reverse, 5′-CTCAGACCACACAGGGAA TG-3′; NQO-1: forward, 5′-GAGAAGAGCCCTGATTGTACTG-3′; and reverse, 5′-ACC TCCCATCCTCTCTTCTT-3′; HO-1: forward, 5′-CTCCCTGTGTTTCCTTTCTCTC-3′; and reverse, 5′-CTGCTGGTTTCAAAGTTCAG-3′; β-actin: forward, 5′-AGGTATCCTGA CCCTGAAGTA-3′; and reverse, 5′-CACACGCAGCTCATTGTAGA-3′. RT-PCR was carried out in triplicate using the SYBR GREEN PCR master mix (Invitrogen, Carlsbad, CA) on a Stratagene MX3000p thermocycler (Agligent StrataGene, Santa Clara, CA). The amount of mRNA was calculated by the comparative CT method, which depends on the ratio of the amount of target genes to reference gene β-actin.

### Histological Examination and Immunohistochemical Staining

The fixed kidney tissues were cut into 3-mm-thick blocks and embedded in paraffin and cut into 4-μm slices. After de-paraffinization and rehydration, the sections were stained with hematoxylin and eosin (H&E) for general morphological examination, periodic acid Schiff (PAS) for evaluation of glomerulosclerosis, and Masson’s trichrome-staining for indication of interstitial expansion. Glomerulosclerosis examination was done by a pathologist in a blinded manner as described previous [30], [31]. Briefly, each slice randomly was selected five different fields containing at least fifty glomeruli, and the degree of glomerulosclerosis was divided into 4 grades from 0 to 4 according to the ratio of sclerosis lesions to each glomerulus (Grade 0, no changes; Grade 1, sclerotic area <1–25%; Grade 2, sclerotic area <26–50%; Grade 3, sclerotic area <51–75%; and Grade 4, sclerotic area <76–100%). The glomerulosclerosis index (GSI) was calculated with following formula: GSI = [(N_1_×1)+(N_2_×2)+(N_3_×3)+(N_4_×4)]/n _tot_, where n_ tot_ is the total number of glomeruli. Similarly, the extent of interstitial expansion was evaluated quantitatively by calculating tubular interstitial collagen-positive area (blue) for Masson’s trichrome-staining using the Image-Pro plus 6.0 software (Media Cybernetics, Silver Spring, Maryland, USA). Twenty consecutive glomeruli were examined for each section, and the averaged percentages of the collagen-positive lesions were obtained for each mouse.

For immunohistochemical staining, the sections were blocked with Superblock buffer (Pierce, Rockford, IL) for 30 min and incubated with the primary antibodies at a 1: 200 dilution overnight at 4°C. Followed by three washes with PBS, sections were incubated with biotin-labeled secondary antibody (Sigma) for 1 h at room temperature. And signal was detected with diaminobenzidine (DAB) and developed according to manufacturer’s instructions. Sections incubated with PBS in place of primary and second antibodies were used as blank controls.

### Statistical Analysis

Data were collected from eight or nine mice in each group, and the results are presented as means ± standard error of the mean (SEM). Statistical analyses were performed using one-way or two-way ANOVA: post-hoc multiple comparisons, followed by Bonferroni with the GraphPad Prism 5.0 (GraphPad Software, San Diego, CA). Statistical significance was detected at the 0.05 level.

## Results

### Diabetic Animal Model and LDR Effects on Hyperglycemia and Insulin Resistance in Type 2 Diabetic Mice

**Figure 1:**
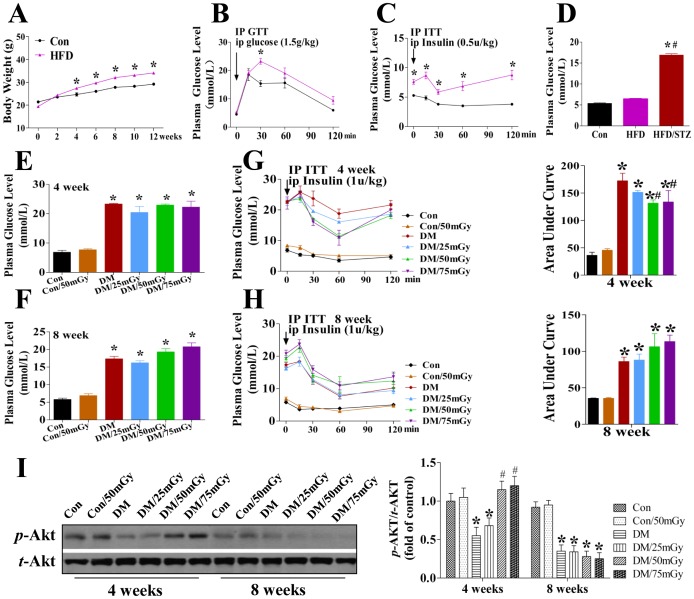
Induction of the type 2 diabetic mouse model and the effect of LDR on diabetes-induced hyperglycemia, insulin resistance and inactivation of Akt. The type 2 diabetic mouse model was established by a combination of a HFD and STZ treatment. C57BL/6J mice were fed with a HFD (40% of calories from fat) for 12 weeks to induce obesity (A), which exhibited abnormal blood glucose tolerance (B) and insulin resistance (C). After intraperitoneal injection of STZ (50 mg/kg), the type 2 diabetic mouse model was successfully induced with the characteristic of hyperglycemia (>12 mmol/L, D). The diabetic or control mice were irradiated with LDR at 25, 50, or 75 mGy over whole-body once every other day for the indicated times. The blood glucose levels and insulin tolerance at the 4-week time-point (E, G) and 8-week time-point (F, H) were examined. The *t*-Akt and *p*-Akt in kidneys were detected by western blotting assay (I). Data are presented as means ± SEM. n = 9 in diabetic group and n = 8 in each other group. *p<0.05 vs. the corresponding control group; #*p*<0.05 vs. the corresponding DM group. Con, control mice; Con/50 mGy, control mice treated with 50 mGy; DM, diabetic mice without LDR treatment; DM/25 mGy, diabetic mice with LDR at 25 mGy; DM/50 mGy, diabetic mice with LDR at 50 mGy; DM/75 mGy, diabetic mice with LDR at 75 mGy.

Since clinical type 2 diabetes is always attributed to obesity in humans, in the present study the mice were fed with a HFD followed by a single injection of STZ to mimic human type 2 diabetes. Compared with SD-fed mice, HFD-fed mice exhibited a significant increase in body weight after treatment for 4 weeks, and the difference gradually increased in a time-dependent manner (Fig. 1A). Meanwhile, the glucose tolerance test and insulin tolerance test revealed that mice fed with HFD for 12 weeks exhibited obvious abnormal glucose tolerance (Fig. 1B) and insulin resistance (Fig. 1C). And hyperglycemia was subsequently induced by a single injection of STZ (Fig. 1D). After successful induction of the type 2 diabetic model, the diabetic mice and age-matched nondiabetic mice were randomly divided into groups with or without LDR treatment. As shown in Fig. 1E, F, exposure to LDR at any dose for 4 or 8 weeks had no impact on hyperglycemia in the type 2 diabetic mice, but 50 and 75 mGy treatment for 4 weeks significantly relieved insulin resistance (Fig. 1G). A similar beneficial effect was not observed at the 8-week time-point (Fig. 1H). This finding was also confirmed by calculating the area under the curve of the insulin tolerance test.

Since Akt plays critical role in regulating insulin sensitivity by mediating insulin-stimulated glucose metabolic signaling, we also examined the expression and phosphorylation of renal Akt among groups to identify whether LDR-induced insulin sensitivity increase was related to Akt activation (Fig. 1I). Results showed that neither diabetes nor LDR affected the expression of renal Akt expression; however, diabetes significantly decreased the phosphorylation of renal Akt, which was significantly reversed by LDR treatment for 4 weeks at 50 or 75 mGy, but not at 25 mGy. Exposure to LDR for 8 weeks also did not significantly affect the diabetic effect on Akt phosphorylation levels (Fig. 1I). This result is consistent with the patterns of IP ITT results (Fig. 1G, H).

### The Effects of LDR on Type 2 Diabetes-induced Renal Hypertrophy and Dysfunction

As we know, the kidney is the target organ highly vulnerable to damage in type 2 diabetes. Therefore, we would investigate whether LDR can prevent diabetic-induced renal hypertrophy and dysfunction. We found diabetes significantly increased the kidney weight and decreased the body weight of the mice. However the tibia length of the mice in each group was unaffected. The ratio of kidney weight to tibia length strongly increased under diabetic condition. After exposure of the mice to LDR for 4 weeks, the magnitude of the ratio increase was slightly suppressed and the suppression became significantly once the LDR extended to 8 weeks (Table 1).

**Table 1:** Assessment of renal function in mice fed a standard diet or a high-fat diet with sham-radiation or radiation.

		Nondiabetic mice		Type 2 diabetic mice			
		Low dose radiation					
Parameters	weeks	0 mGy	50 mGy	0 mGy	25 mGy	50 mGy	75 mGy
Body weight (g)	4	31.10±	29.66±	27.58±	29.80±	29.53±	28.90±
		0.9	1.05	0.84*	0.9	0.78	0.85*
	8	31.38±	30.46±	28.38±	30.20±	29.33±	29.23±
		1.08	1.2	0.45*	0.82	0.7	1.00*
Kidney weight (g)	4	0.43±	0.39±	0.67±	0.59±	0.53±	0.51±
		0.01	0.03	0.05*	0.03*	0.01	0.02#
	8	0.44±	0.39±	0.66±	0.58±	0.52±	0.51±
		0.02	0.03	0.06*	0.02*	0.06#	0.04#
Urinary volume (mL/24 h)	4	1.10±	1.10±	10.52±	10.30±	10. 20±	9.75±
		0.12	0.14	0.7	0.78	0.45	0.68
	8	1.15±	1.00±	10.80±	10.35±	10.50±	10.30±
		0.13	0.13	0.81	0.58	0.6	0.82
Urinary protein (µg/day)	4	98.3±	92.9±	1048.9±	885.5±	879.8±	848.5±
		1.9	2.3	15.8*	11.5*#	12.6*#	15.3*#
	8	105.9±	107.1±	1134.0±	923.2±	995.8±	967.5±
		1.2	1.5	22.5*	12.8*#	15.4*#	20.7*#
Urinary mAlb (µg/day)	4	6.1±	6.8±	318.2±	297.2±	194.5±	203.9±
		0.4	0.4	13.2*	10.2*	11.7*#	16.4 *#
	8	6.7±	7.2±	382.1±	295.5±	336.4±	378.8±
		0.3	0.4	18.0*	15.5*#	13.6*#	14.4*
Urinary creatinine (µmol/day)	4	40.6±	41.5±	360.9±	387.6±	397.4±	385.7±
		1.6	1	13.6*	16.8*#	13.4*#	15.3*#
	8	41.7±	38.5±	376.9±	386.5±	381.6±	370.8±
		1.5	1.1	16.2*	15.7*	11.5*	14.9*

Notes: Data were presented as means ± SEM. n = 9 in diabetic group and n = 8 in each other group. mAlb, microalbumin;

*p<0.05 vs. the corresponding control group;

# *p*<0.05 vs. the corresponding DM group;

& *p*<0.05 vs. the corresponding DM/25 mGy group.

It is known that renal dysfunction manifests as an increase of the urinary volume, urinary protein and microalbumin (mAlb) and a decrease of urinary creatinine. We observed the above phenomenon in type 2 diabetic mice at both the 4- and 8-week time-points. Exposure of the mice to LDR for 4 weeks significantly prevented an increase of urinary protein and mAlb excretion. In contrast, urinary creatinine excretion was significantly increased after LDR treatment. However, the urinary volume was unaffected. In addition, the beneficial effects of LDR on diabetes induced renal dysfunction was largely diminished (Table 1).

### LDR Prevented Type 2 Diabetes-induced Pathological Changes and Glomerulosclerosis in the Kidney

**Figure 2:**
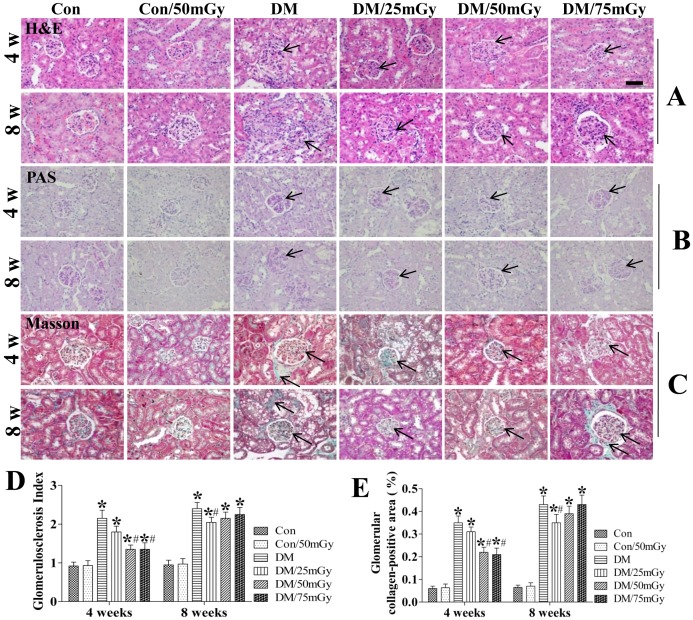
Exposure to LDR attenuated diabetes-induced histopathological changes in the kidneys of mice. Representative images of hematoxylin and eosin (H&E; panel A) staining, periodic acid-Schiff (PAS; panel B) staining, and Masson’s trichrome staining (Masson; panel C) for detection of renal pathological changes, glomerulosclerosis and collagen deposition, respectively. 400× magnification. The glomerulosclerosis and collagen accumulation were examined in PAS or Masson stained slices respectively (D, E) and quantified using Image-Pro plus 6.0 software. Data are presented as means ± SEM. n = 9 in diabetic group and n = 8 in each other group. *p<0.05 vs. the corresponding control group; #*p*<0.05 vs. the corresponding DM group.

Generally, renal dysfunction reflects pathological changes in the diabetic kidney. Compared with the control group, mice in the Con/50 mGy group displayed a normal structure of glomerulus and renal tubules by H&E examination (Fig. 2A). However, the diabetic kidneys showed obvious Bowman’s capsule adhesion, mesangial cell proliferation, mesangial matrix expansion and capillary collapse at the 4-week time-point. Simultaneously, renal tubular dilation and epithelial cell degeneration were also observed in the diabetic kidneys. Furthermore, there were some bubbles in the renal tubules, which were attributed to excessive lipid accumulation in the diabetic kidneys (Fig. 2A). Treatment with 50 mGy or 75 mGy for 4 weeks markedly attenuated the above pathological changes (Fig. 2A).

Meanwhile, kidney tissues were stained with PAS to detect glomerulosclerosis. As shown in (Fig. 2B and 2D). The glomerulosclerosis index was significantly increased in diabetic mice with a time-dependent manner. Exposure to LDR treatment for 4 weeks at 50 and 75 mGy, but not 25 mGy, greatly prevented glomerulosclerosis. However, the protective effect was attenuated once exposure of LDR extended to 8 weeks and only 25 mGy showed notable suppression on diabetes induced glomerulosclerosis.

Masson’s trichrome staining showed significant accumulation of collagen in the kidneys of diabetic mice at the 4-week time-point, indicating the development of interstitial fibrosis (Fig. 2C, E). The collagen accumulation and fibrosis were more evident in the kidneys of diabetic mice at the 8-week time-point. Exposure to LDR at doses of 50 and 75 mGy significantly prevented the above phenomenon in type 2 diabetic mice at the 4-week time-point (Fig. 2C, E). The anti-fibrotic effect of LDR became weaker as time progressed to the 8-week time-point and only 25 mGy induced remarkably positive effects on the pathological changes under diabetic conditions (Fig. 2C, E).

### LDR Prevented Systemic and Renal Dyslipidemia in Type 2 Diabetic Mice

Dyslipidemia is regarded as a main generator of type 2 diabetes-induced renal damage. Thus, a mechanistic study was carried out to investigate whether LDR can induce beneficial effects on the abnormal lipid profiles in type 2 diabetic mice. We found that under physiological conditions, there was no change in the lipid profiles in Con/50 mGy group (Table 2). In contrast, type 2 diabetes significantly increased plasma TG levels at the 4-week time-point, which was further enhanced at the 8-week time-point (Table 2). Although a dose of 25 mGy did not, LDR at 50 and 75 mGy for 4 weeks remarkably prevented increased plasma TG levels in diabetic mice (Table 2). However, the attenuation effect of LDR at these two doses vanished at the 8-week time-point (Table 2). In contrast, 25 mGy significantly lowered plasma TG under diabetic conditions at the 8-week time-point (Table 2). In addition, we also found that type 2 diabetes strongly increased the total CHO and LDL levels in the plasma at both the 4- and 8-week time-points. Exposure to LDR at any dose remarkably induced a decrease in the plasma LDL levels at the 4-week time-point (Table 2). However, at the 8-week time-point, only LDR at 25 mGy showed a preventive effect on plasma LDL levels in type 2 diabetes. The similar lowering effect due to LDR was not found on total plasma CHO levels at either time-point (Table 2). Furthermore, the results also showed that type 2 diabetes at the 4-week time-point did not, but at the 8-week time-point significantly decreased plasma HDL levels. In contrast, LDR at 50 and 75 mGy remarkably increased plasma HDL levels at both the 4- and 8-week time-point; while 25 mGy showed the similar increasing effect on plasma HDL levels only observed at the 8-week time-point (Table 2).

**Table 2:** Plasma and renal lipid metabolic parameters in mice fed a standard diet or a high-fat diet with sham-radiation or radiation.

		Nondiabetic mice		Type 2 diabetic mice			
		Low dose radiation					
Parameters	weeks	0 mGy	50 mGy	0 mGy	25 mGy	50 mGy	75 mGy
Plasma TG (mmol/L)	4	1.39±	1.25±	4.62±	4.11±	3.26±	3.37±
		0.12	0.14	0.19*	0.22*	0.24*#	0.18*#
	8	1.40±	1.20±	6.16±	4.72±	5.56±	5.70±
		0.13	0.12	0.28*	0.35*#	0.62*&	0.44*&
Plasma CHO (mmol/L)	4	3.12±	3.09±	4.60±	4.12±	4.32±	4.62±
		0.17	0.29	0.45*	0.30*	0.52*	0.43*
	8	3.24±	3.25±	4.86±	4.82±	4.78±	4.90±
		0.21	0.19	0.52*	0.48*	0.64*	0.45*
Plasma HDL (mmol/L)	4	1.79±	1.75±	1.78±	1.93±	2. 46±	2.49±
		0.14	0.20	0.19	0.24	0.36*#	0.20*#
	8	1.81±	1.88±	1.60±	2.45±	2.48±	2.23±
		0.18	0.22	0.17*	0.15*#	0.21*#	0.29*#
Plasma LDL (mmol/L)	4	0.29±	0.28±	0.46±	0.33±	0.35±	0.38±
		0.03	0.02	0.05*	0.05#	0.03#	0.03#
	8	0.31±	0.32±	0.53±	0.40±	0.52±	0.53±
		0.02	0.04	0.04*	0.03*	0.05*&	0.05*&
Renal TG (mg/g of tissue)	4	1.84±	1.78±	8.56±	7.65±	5.60±	5.45±
		0.10	0.10	0.95*	0.66*	0.45*#	0.58*#
	8	1.85±	1.90±	10.50±	8.86±	9.77±	10.64±
		0.20	0.14	0.70*	0.63*#	0.92*&	0.66*&
Renal FFA (mg/g of tissue)	4	7.85±	7.68±	14.65±	14.06±	10.26±	10.35±
		0.27	0.30	1.00*	0.88*	0.64*#	0.75*#
	8	7.95±	8.12±	17.62±	15.24±	16.78±	17.85±
		0.20	0.40	0.82*	1.05*	0.86*	0.95*

Notes: Data were presented as means ± SEM. n = 9 in diabetic group and n = 8 in each other group. TG, Triglyceride; FFA, free fatty acid; CHO, cholesterol; HDL, high-density lipoprotein; LDL, low-density lipoprotein.

*p<0.05 vs. the corresponding control group;

#*p*<0.05 vs. the corresponding DM group;

& *p*<0.05 vs. the corresponding DM/25 mGy group.

Consistent with the plasma lipid profile examination in the kidney, increased renal TG and FFA levels were also observed in diabetic mice at both the 4- and 8-week time-points. LDR at 50 and 75 mGy, but not at 25 mGy, remarkably inhibited renal TG and FFA level increases in type 2 diabetic mice at the 4-week time-point (Table 2). The attenuation effect of LDR at these two doses vanished and only 25 mGy slightly prevented but significantly diabetes-induced renal TG increase at the 8-week time-point (Table 2).

### LDR Attenuated Renal Inflammation in Type 2 Diabetic Mice

**Figure 3:**
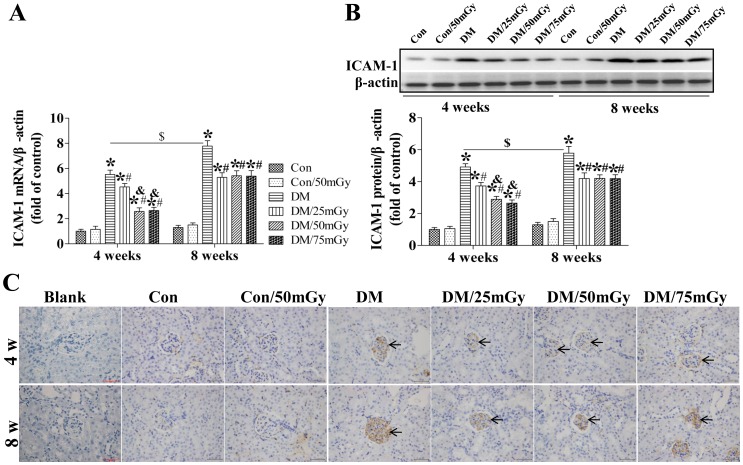
Effect of LDR on renal ICAM-1 levels in type 2 diabetic mice. Renal tissues from different groups were collected at the indicated times for measuring ICAM-1 expression at the mRNA (A) and protein (B) levels with RT-PCR and western blotting, respectively. (C) The location of ICAM-1 in the kidney was detected by immunohistochemical staining, at 400× magnification. Data are presented as means ± SEM. n = 9 in diabetic group and n = 8 in each other group. *p<0.05 vs. the corresponding control group; #*p*<0.05 vs. the corresponding DM group; &, *p*<0.05 vs. the corresponding DM/25 mGy; $, *p*<0.05 vs. DM group at the 4-week time-point.

Dyslipidemia in type 2 diabetes always leads to lipotoxicity in the kidney, which can induce renal damage associated with the severe inflammation characterized by the release of multiple inflammatory factors. To define the effects of LDR on diabetes-induced renal inflammation, renal expression of ICAM-1 mRNA and protein was measured by RT-PCR (Fig. 3A) and western blotting assays (Fig. 3B), respectively. Diabetes induced strong increases in renal ICAM-1 at both the mRNA and protein levels at the 4-week time-point, which were further enhanced at the 8-week time-point. By immunohistochemical staining, diabetes-increased ICAM-1 was found to be expressed predominantly in the glomeruli (Fig. 3C), which was not observed in blank control group (no primary and secondary antibody, Fig. 3C). Multiple exposures of control mice to 50 mGy had no impact on renal ICAM-1 levels but significantly attenuated diabetes-induced ICAM-1 levels after treatment for 4 weeks (Fig. 3A, B). Simultaneously, compared with 25 mGy, LDR at 50 and 75 mGy further attenuated renal ICAM-1 levels in diabetic kidney (Fig. 3A, B). The anti-inflammatory effect of LDR became weaker at the 8-week time-point, although the preventive effect still existed (Fig. 3A, B).

**Figure 4:**
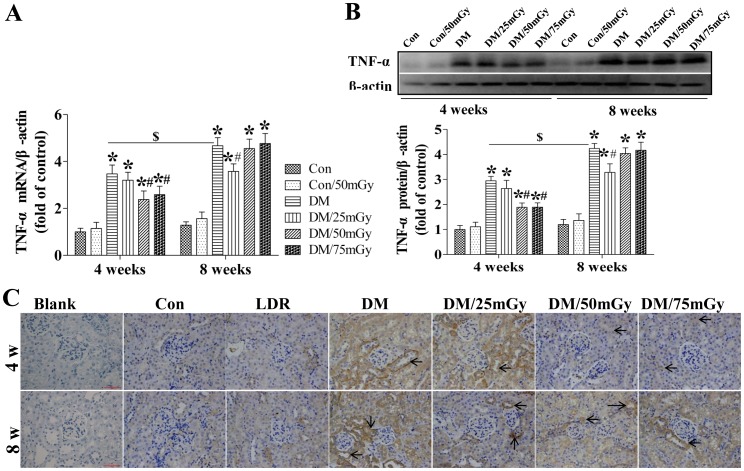
Effect of LDR on renal TNF-α levels in type 2 diabetic mice. Renal tissues from different groups were collected at the indicated times for measuring TNF-α expression at the mRNA (A) and protein (B) levels with RT-PCR and western blotting, respectively. (C) The location of TNF-α in the kidney was detected by immunohistochemical staining, at 400× magnification. Data are presented as means ± SEM. n = 9 in diabetic group and n = 8 in each other group. *p<0.05 vs. the corresponding control group; #*p*<0.05 vs. the corresponding DM group; $, *p*<0.05 vs. DM group at the 4-week time-point.

Renal TNF-α expression was also found to be significantly increased in diabetic groups at the mRNA (Fig. 4A) and protein (Fig. 4B) levels in a time-dependent manner. The localization of increased TNF-α expression was found predominantly in tubules and less in the glomerulus (Fig. 4C). The positive staining of TNF-α was increased in diabetic group, which was much less in control group and was unobservable in blank control group (Fig. 4C). Under physiological conditions, control mice exposure to 50 mGy did not change the renal TNF-α level, which were significantly attenuated by LDR treatment at 50 and 75 mGy for 4 weeks in the diabetic kidneys (Fig. 4A, B). However, the anti-inflammatory effect of LDR at these two doses vanished at the 8-week time-point. Meanwhile, only 25 mGy induced an attenuation effect on renal TNF-α expression at both the mRNA and protein levels (Fig. 4A, B).

**Figure 5:**
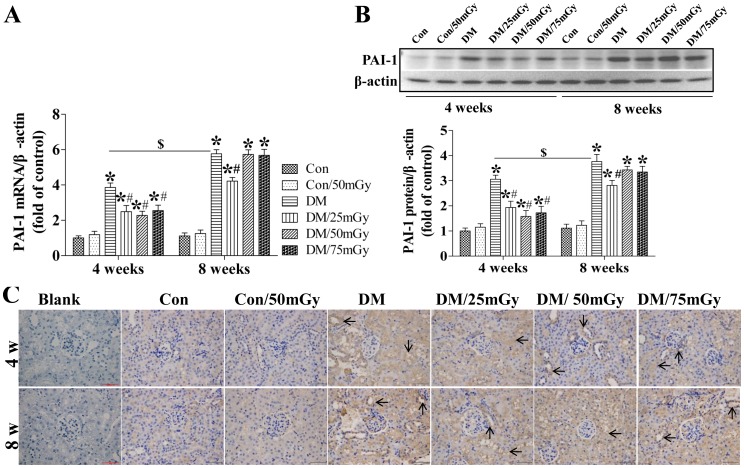
Effect of LDR on renal PAI-1 levels in type 2 diabetic mice. Renal tissues from different groups were collected at the indicated times for measuring PAI-1 expression at the mRNA (A) and protein (B) levels with RT-PCR and western blotting, respectively. (C) The location of PAI-1 in the kidney was detected by immunohistochemical staining, at 400× magnification. Data are presented as means ± SEM. n = 9 in diabetic group and n = 8 in each other group. *p<0.05 vs. the corresponding control group; #*p*<0.05 vs. the corresponding DM group; $, *p*<0.05 vs. DM group at the 4-week time-point.

The proinflammatory cytokine PAI-1 is also profibrotic given its inhibition of the conversion of plasminogen to plasmin [32], [33]. Moreover, in the present study, we found that exposure to LDR significantly prevented type 2 diabetes-induced inflammation and fibrosis. Therefore, we next determined whether the beneficial effect of LDR was associated with attenuation of renal PAI-1 expression at both the mRNA and protein levels. Real-time PCR and western blotting assays revealed that type 2 diabetes significantly up-regulated renal PAI-1 levels at the 4-week time-point, which were further strengthened at the 8-week time-point (Fig. 5A, B). Immunohistochemical staining confirmed our finding that diabetes-induced PAI-1 expression mainly located in the tubules which was less in the kidneys of nondiabetic mice and unobservable in blank control (Fig. 5C). Exposure to LDR at all doses remarkably attenuated renal PAI-1 levels at the 4-week time-point in diabetic mice. Furthermore, LDR at 50 and 75 mGy showed a greater attenuation effect than at 25 mGy; although the difference did not reach a level of significance (Fig. 5A, B). However, the beneficial effect of LDR at 50 and 75 mGy vanished at the 8-week time-point and only 25 mGy induced remarkable inhibition of the diabetes-caused renal PAI-1 expression increase (Fig. 5A, B).

### LDR Prevented Renal Oxidative Stress in Type 2 Diabetic Mice

**Figure 6:**
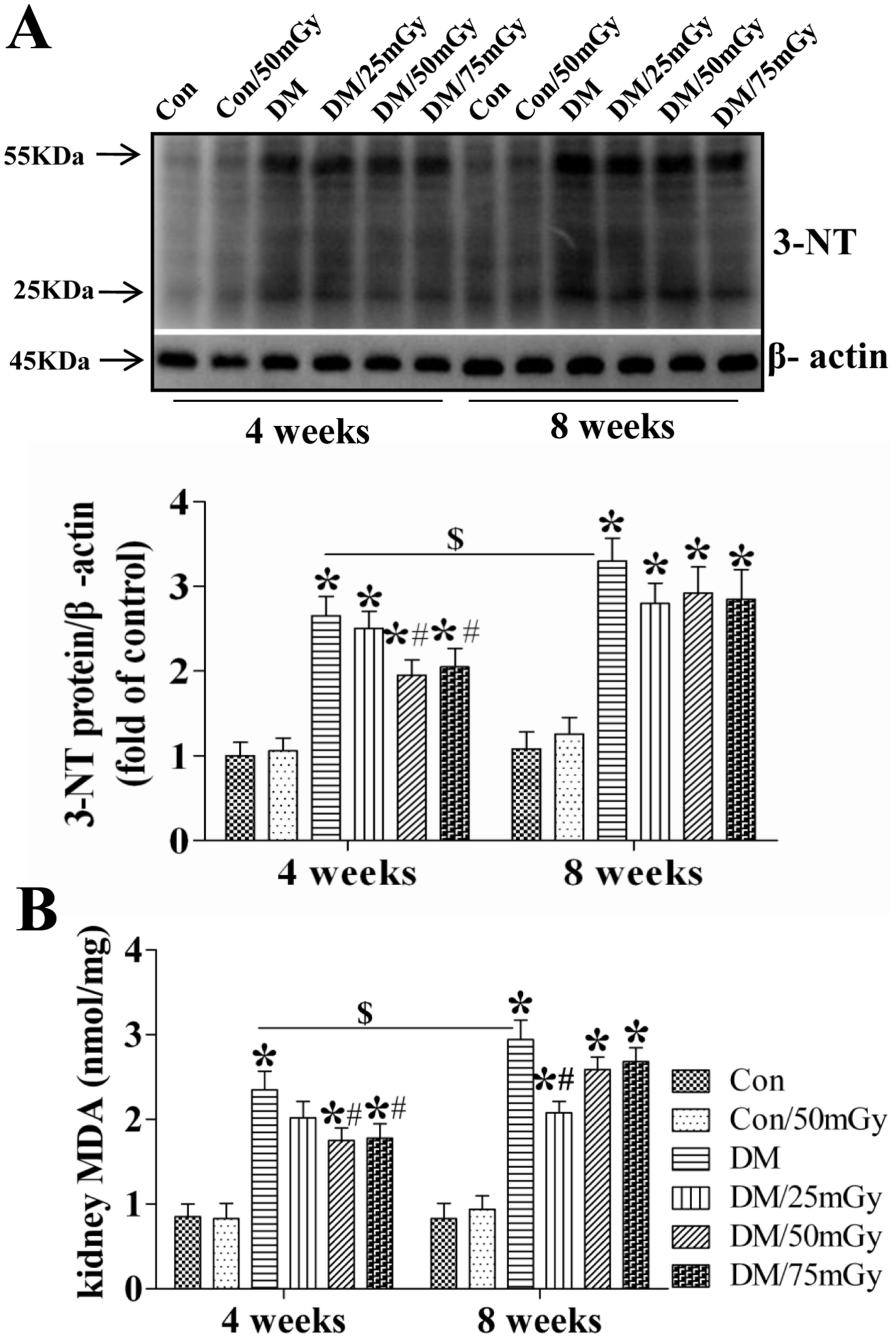
Effects of LDR on renal oxidative damage in type 2 diabetic mice. After control and diabetic mice with and without multiple exposures to LDR for 4 and 8 weeks, renal oxidative damage was examined by western blotting assays for the expression of 3-NT as an index of protein nitration (A, B) and MDA by ELISA (C). Data are presented as means ± SEM. n = 9 in diabetic group and n = 8 in each other group. *p<0.05 vs. the corresponding control group; #*p*<0.05 vs. the corresponding DM group; $, *p*<0.05 vs. DM group at the 4-week time-point.

Oxidative stress is a common consequence of type 2 diabetes closely associated with lipotoxicity. It is also considered as the main pathogenesis of diabetic renal damage. Hence, we further identified the effect of LDR on lipotoxicity-induced oxidative damage measured by 3-NT as an index of nitrosative damage (Fig. 6A) and MDA as a classic oxidative damage marker (Fig. 6B). The results showed that both renal 3-NT and MDA had similar changing patterns among groups. Type 2 diabetes significantly increased their levels in the kidney at the 4-week time-point, and they were further enhanced at the 8-week time-point. LDR at 25 mGy did not, but at 50 and 75 mGy significantly attenuated renal 3-NT and MDA levels at the 4-week time-point under diabetic conditions (Fig. 6). A similar preventive effect of LDR was not found on the 3-NT level at the 8-week time-point; however, LDR at 25 mGy, rather than at 50 and 75, remarkably attenuated the renal MDA level at the same time-point (Fig. 6).

### LDR Prevented Diabetic Downregulation of Renal Nuclear Factor E2-related Factor-2 (Nrf-2) Expression and Function

**Figure 7:**
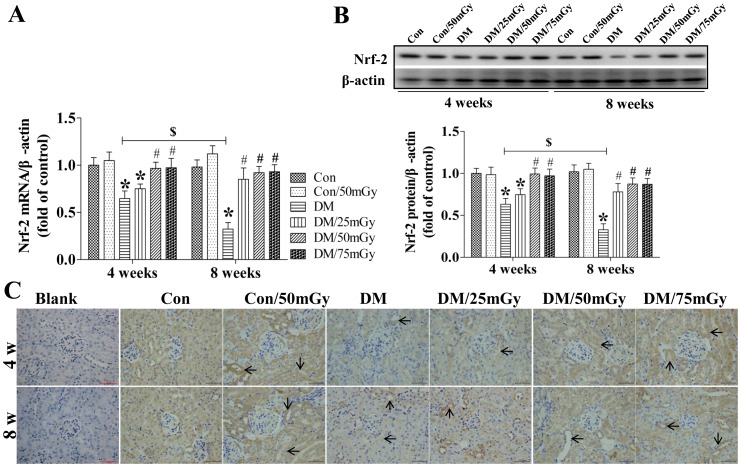
Effect of LDR on renal Nrf-2 levels in type 2 diabetic mice. Renal tissues from different groups were collected at the indicated times for measuring Nrf-2 expression at the mRNA (A) and protein (B) levels with RT-PCR and western blotting, respectively. (C) The location of Nrf-2 in the kidney was detected by immunohistochemical staining, at 400× magnification. Data are presented as means ± SEM. n = 9 in diabetic group and n = 8 in each other group. *p<0.05 vs. the corresponding control group; #*p*<0.05 vs. the corresponding DM group; $, *p*<0.05 vs. DM mice at the 4-week time-point.

Nrf-2 is a key transcription factor that regulates intracellular redox balance and is a sensor of oxidative stress [34]. Next, we determined whether LDR-induced renal protection against oxidative damage was associated with up-regulation of renal Nrf-2 levels. The results showed that the expression of Nrf-2 at both the mRNA and protein levels significantly decreased in the kidneys of diabetic mice at the 4-week time-point, and the expression levels were further decreased at the 8-week time-point (Fig. 7A, B). Multiple exposures of diabetic mice to LDR at 25 mGy for 4 weeks did not, but 50 or 75 mGy almost completely prevented diabetic inhibition of renal Nrf-2 levels. Although exposure to LDR still up-regulated renal Nrf-2 levels after treatment for 8 weeks, the effect was weaker (Fig. 7A, B).

Immunohistochemical staining revealed that Nrf-2 expression was predominantly localized in the tubules and less in the glomerulus (Fig. 7C). However, no positive staining was observed in blank control group. The intensity of Nrf-2 staining was significantly reduced in the kidneys of diabetic mice, which was greatly reversed after LDR treatment for 4 weeks (Fig. 7C).

**Figure 8:**
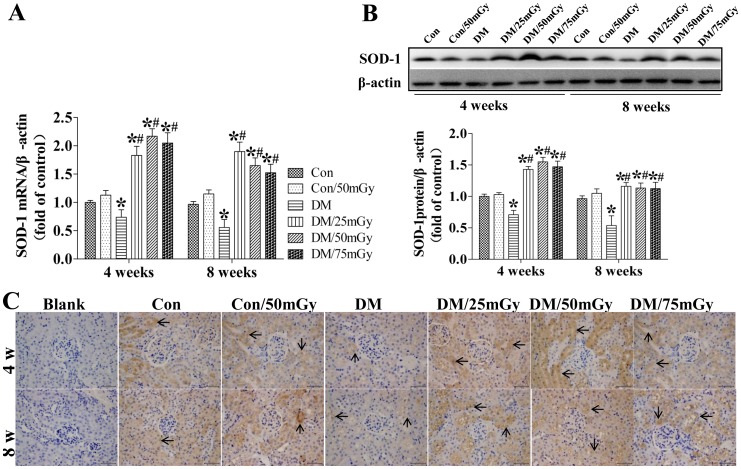
Effect of LDR on renal SOD-1 levels in type 2 diabetic mice. Renal tissues from different groups were collected at the indicated times for measuring SOD-1 expression at the mRNA (A) and protein (B) levels with RT-PCR and western blotting, respectively. (C) The location of SOD-1 in the kidney was detected by immunohistochemical staining, at 400× magnification. Data are presented as means ± SEM. n = 9 in diabetic group and n = 8 in each other group. *p<0.05 vs. the corresponding control group; #*p*<0.05 vs. the corresponding DM group; $, p<0.05 vs. DM group at the 4-week time-point.

Since Nrf-2 is a transcription factor that positively regulates the expression of several downstream genes playing an important role in the prevention of oxidative stress and damage, we examined several of its downstream genes to functionally evaluate Nrf-2 in the diabetic kidneys with and without LDR treatment. Real-time PCR and western blotting assays disclosed that renal SOD-1 expression was significantly decreased in the diabetic group compared with the control group at the 4-week time-point and further decreased at the 8-week time-point (Fig. 8A, B). However, the decrease was completely reversed by exposure to LDR at all doses at both 4 and 8 weeks (Fig. 8A, B). Immunohistochemical staining confirmed our finding that exposure to LDR at all doses significantly preserved renal SOD-1 expression, located mainly in the tubules, in type 2 diabetic mice (Fig. 8C). And no positive staining was observed in blank control group.

**Figure 9:**
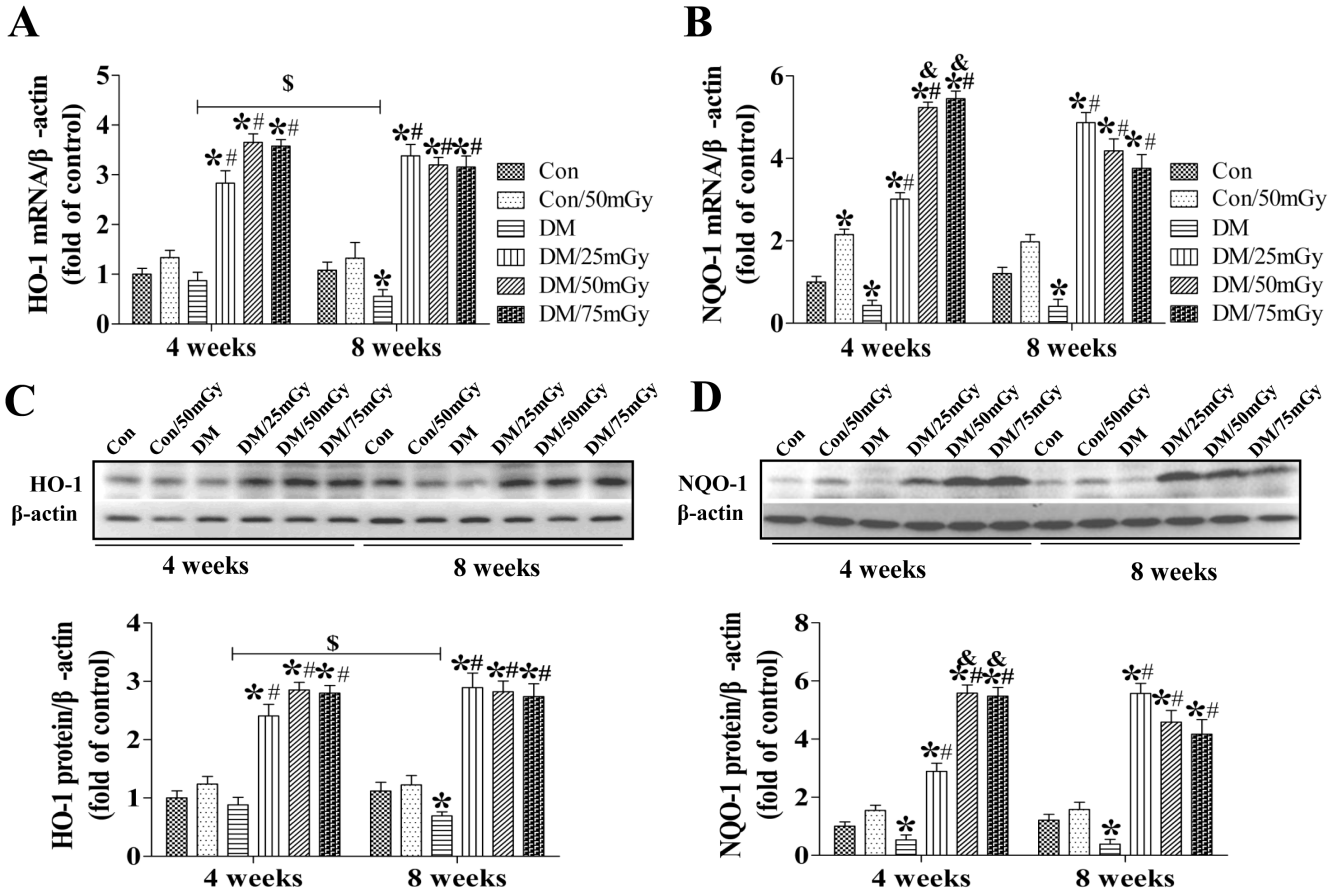
Effect of LDR on renal HO-1 and NQO-1 levels in type 2 diabetic mice. Renal tissues from different groups were collected at the indicated times for measuring HO-1 expression (A, C) and NQO-1 expression (B, D) levels at the mRNA and protein with RT-PCR and western blotting, respectively. Data are presented as means ± SEM. n = 9 in diabetic group and n = 8 in each other group. *p<0.05 vs. the corresponding control group; #*p*<0.05 vs. the corresponding DM group; $, *p*<0.05 vs. DM group at the 4-week time-point.

In addition, we also examined other downstream targets of Nrf-2, HO-1 and NQO-1, which showed similar changing patterns as SOD-1 among groups. Renal HO-1 and NQO-1 levels were significantly decreased under diabetic conditions at both the 4- and 8-week time-points (Fig. 9). Exposure to LDR for both 4 and 8 weeks completely preserved the renal expression of both anti-oxidants. Although the beneficial effects were even among all doses of LDR at the 8-week time-point, 50 and 75 mGy further enhanced the protection compared with 25 mGy at the 4-week time-point.

## Discussion

The study is the first one to demonstrate that multiple exposures to LDR, especially at 50 and 75 mGy for 4 weeks, significantly attenuated type 2 diabetes-induced DKD attributed to not only alleviation of systemic and renal dyslipidemia, but also suppression of the subsequent lipotoxicity via relieving insulin resistance, renal inflammation and oxidative damage.

The kidney is the main target organ that is attacked under conditions of type 2 diabetes, which leads to severe renal damage characterized by induction of renal dysfunction, fibrosis and glomerulosclerosis, finally causing DN [3]–[5]. Although the pathogeneses of type 2 diabetes-induced renal damage are multiple and complicated, dyslipidemia and subsequent lipotoxicity play important roles in this pathological process and accelerate kidney injury [20], [35], [36]. Dyslipidemia is defined as abnormal lipid profiles characterized by an increase of plasma and tissue TG, LDL and total CHO levels, but a decrease of HDL levels [10], [11]. Disturbances in lipoprotein metabolism contribute to atherogenic diathesis and possibly to progression of renal disease and impaired energy metabolism in DKD [12]. Diabetes-related dyslipidemia always leads to excessive lipid accumulation in the kidney, which causes severe renal damage through enhancement of insulin resistance, inflammation and oxidative stress [9], [37], [38]. In addition, insulin resistance induces the release of adipocytokines and relaxation of the afferent arteriole, resulting in glomerular hyperfiltration, angiogenesis, mesangial cell proliferation and renal damage [17], [18]. The inflammatory response has been considered as one of the major mechanisms by which diabetic lipotoxicity causes renal oxidative injury, structural changes and dysfunction through the release of multiple inflammatory factors [39]–[41]. Moreover, lipotoxicity also induces excess lipid peroxidation products that stimulate transforming growth factor-β, thereby inducing oxidative stress by production of ROS and then causing renal function damage, which may contribute to the progression of DKD [19], [20]. Therefore, finding a proper way to simultaneously prevent dyslipidemia and the subsequent lipotoxicity-induced insulin resistance, inflammation and oxidative stress might be an efficient candidate for DKD therapy.

Ionizing radiation is commonly considered to be harmful, which usually leads to DNA damage, cytotoxicity and tumorigenesis [42]–[45]. However, increasing evidence has demonstrated that exposure to radiation at a relatively low level resists radiation- or chemical-caused damage due to the protective mechanisms of stimulating cellular metabolic activities, enhancing immunity and increasing anti-oxidative action [46]–[49]. Additionally, it has been reported that LDR has the potential to modify the progression of chronic renal failure in rats [24], [50]. Furthermore, our previous studies found that LDR significantly prevents type 1 diabetes-induced renal damage through attenuation of inflammation and oxidative stress [23], [26]. Meanwhile, another advantage of LDR is its noninvasive characteristics, which cannot increase the working load of the kidney. Therefore, in the present study, we investigated whether LDR had the potential to prevent type 2 diabetes-induced renal damage and whether the protection was associated with attenuation of diabetes-induced dyslipidemia and lipotoxicity.

In order to test our hypothesis, the first mission for us was to establish a human-like type 2 diabetic mouse model. Although several genetically modified animal models are available to study type 2 diabetes, the patterns of disease initiation and development do not appear to be closely analogous to the clinical situation in humans [28], [51]. Moreover, the observations derived from these highly inbred genetic strains may not always be satisfactorily extended to the human population as a whole because of the large heterogeneity in the latter. In addition, these animals are expensive and are not easily available for investigative purposes as well as regular screening experiments [28], [52]. In humans, despite hereditary reasons, most type 2 diabetes cases are initiated by obesity. Therefore, we mimicked human-like type 2 diabetes by HFD/STZ strategy in C57BL/6J mice for which the type 2 diabetic mouse model was induced via HFD treatment for 12 weeks, followed by a single low-dose STZ injection of 50 mg/kg.

Once the type 2 diabetic model was successfully established, the diabetic or nondiabetic mice were exposed to LDR at 25, 50 or 75 mGy for 4 or 8 weeks. Then, the next question we have to answer is whether LDR can prevent type 2 diabetes-induced renal damage. Normally, the renal hypertrophy was determined by the ratio of kidney weight to body weight under the premise of constant body weight. But in our study the body weight varied among groups which will cause inaccurate conclusion. Therefore we used tibia length to replace the body weight, which was proximately even in each group. The results showed the ratio of kidney weight to tibia length was increased in diabetic group, which is a sign of renal hypertrophy. In addition, there was an increase of the urinary protein, mAlb excretion and a decrease of urinary creatinine excretion. Morphological studies also revealed that type 2 diabetes significantly induced renal pathological changes, including obvious Bowman’s capsule adhesion, mesangial cell proliferation, mesangial matrix expansion, capillary wall thickness, capillary collapse, glomerulosclerosis and fibrosis. Meanwhile, abnormal lipid accumulation was also obvious in the kidney tissue of type 2 diabetic mice. However, most of the pathological changes induced by type 2 diabetes were prevented by exposure to LDR for 4 weeks. Although the beneficial effects of LDR were still observed at the 8-week time-point, the efficiency became weaker. Our findings implied that besides the single dose, the accumulated dose also plays a critical role in LDR-induced hormesis and beneficial effects on type 2 diabetes-induced renal damage. Despite the fact that each dose of radiation was equal in the groups treated for 4 or 8 weeks, the accumulated dose for 8 weeks was too high to further enhance the beneficial effects on diabetic renal damage compared with the 4-week time-point. After comprehensive evaluation of the renal protection among the three doses of LDR, we found that 50 and 75 mGy were more efficient than 25 mGy at the 4-week time-point. However, the above beneficial phenomenon was attenuated at the 8-week time-point; only LDR at 25 mGy showed great renal protection against type 2 diabetes at the 8-week time-point. The proper explanation might be that the accumulated dose of 50 mGy for 4 weeks and 25 mGy for 8 weeks were the same. Thus, both doses of LDR exhibited similar protection on diabetic renal damage.

To date, we identified that LDR prevented type 2 diabetes-induced renal damage characterized by suppression of kidney hypertrophic response, attenuation of renal dysfunction and pathological changes; so, the protective mechanism of LDR remains to be elucidated. In our study, we found that LDR-induced renal protection against type 2 diabetes was independent of hypoglycemic effects but associated with improving insulin sensitivity, which might be attributed to activation of the Akt signaling pathway. Since insulin-induced glucose regulation was mainly mediated by the Akt signaling pathway, impairment of the Akt signaling pathway always leads to insulin resistance. In the literature, several studies have used *in vitro* models to show that LDR is able to stimulate cell survival signaling such as Akt phosphorylation [48], [53]. Our previous study also determined that exposure to LDR significantly up-regulated the activation of the renal Akt signaling pathway in type 1 diabetic mice. Moreover, the Akt signaling pathway also plays an important role in preventing diabetes-induced renal cell death [54]. Similar anti-apoptotic effects of Akt in response to various pathogenic stimuli have also been extensively reported [55], [56]. Therefore, we assumed that stimulation of the Akt signaling pathway by LDR in diabetic kidneys may be one of the possible mechanisms by which LDR prevents the development of DN.

Furthermore, strong evidence has demonstrated that besides hyperglycemia, lipotoxicity is another main pathogenesis of type 2 diabetes-induced insulin resistance by increasing the inhibition of serine phosphorylation of the key mediators of insulin receptor signaling [13]–[15]. Accordingly, we hypothesized that the renal protection of LDR might be related to the attenuation of lipotoxicity. Since lipotoxicity is the consequence of dyslipidemia, we next determined the systemic and renal lipid profiles. The results disclosed that type 2 diabetes-induced dyslipidemia in both the plasma and kidney was characterized by an increase of CHO, TG and LDL levels and a decrease of HDL levels. However, some of the abnormal lipid profiles were remarkably reversed by exposure to LDR, especially at 50 and 75 mGy. Although the lipid metabolic regulating effect was still observed at the 8-week time-point, the efficiency was getting weaker due to the high accumulated dose.

Dyslipidemia-induced lipotoxicity always leads to severe inflammation and oxidative stress, which also accelerates renal damage under diabetic conditions. Real-time PCR and western blotting assays revealed that type 2 diabetes significantly up-regulated the expression levels of inflammatory factors (ICAM-1, TNF-α and PAI-1) and nitrosative damage markers (3-NT and MDA) in the kidney. Exposure to LDR, especially at 50 and 75 mGy, for 4 weeks greatly prevented diabetes-induced inflammation and oxidative stress. Although LDR at 25 mGy induced less of an effect on anti-inflammatory and anti-oxidative stress at the 4-week time-point, the beneficial effect became significant at the 8-week time-point. This finding further confirmed that the accumulated dose is critical for LDR-induced renal protection against inflammation and oxidative stress induced by type 2 diabetes. In the present study, LDR prevented most of the renal pathological changes only with the total dose ranging from 700 mGy (50 mGy for 4 weeks) to 1035 mGy (75 mGy for 4 weeks). A dose that is either too low or too high will reduce the protective effect of LDR.

Finally, we explored the mechanism of LDR-induced anti-oxidative stress. Our previous study has indicated that LDR protection of rat testes from diabetes-induced oxidative damage occurred along with an up-regulation of testicular superoxide dismutase levels [57]. Recently, we also reported that LDR-induced up-regulation of multiple renal antioxidant levels was mediated by Nrf-2 expression in the kidney [26]. Nrf-2 is a key transcription factor that regulates the intracellular redox balance and is a sensor of oxidative stress. SOD-1, NQO-1 and HO-1 are all well-studied Nrf-2 target genes that are up-regulated through the antioxidant response regulatory element in response to oxidative stress [26], [29], [58]. In the present study, we found that type 2 diabetes significantly down-regulated the expression of renal Nrf-2 and the downstream targets SOD-1, HO-1 and NQO-1, which were remarkably reversed by exposure to LDR, especially at 50 or 75 mGy for both 4 and 8 weeks. This study answered our question that LDR protects the kidney from type 2 diabetes-induced oxidative damage associated with up-regulation of Nrf-2 and the downstream target oxidants. It is interesting that the antioxidant expression in each DM/LDR group was much higher than in Con, Con/50 mGy or DM group. Why does this phenomenon appear? We suspected that under normal condition, oxidative stress is just kept at low level and unharmful to the kidney. However, under diabetic condition, renal oxidative stress significantly increased attributed to up-regulation of ROS production and down-regulation of the anti-oxidants expressions [26], [59]–[64]. LDR induced increase of antioxidants is not only to compensate the decrease of renal anti-oxidants expression induced by diabetes [26], [65], but also to neutralize the ROS production in diabetic kidney [66]–[69]. Therefore, the expressions of multiple anti-oxidants in the kidney were significantly higher than in Con or DM groups.

## Conclusion

In summary, we showed for the first time that exposure of mice to LDR significantly prevented type 2 diabetes-induced renal damage through attenuation of renal hypertrophy, dysfunction and pathological changes. The mechanistic study demonstrated that the protection was independent of blood glucose level reduction but was attributed to the suppression of dyslipidemia and the subsequent lipotoxicity-induced insulin resistance, inflammation and oxidative stress. The beneficial effects of LDR on renal damage were associated with activation of the Akt signaling pathway and up-regulation of Nrf-2 expression and function. In the present study, we also found that both the single and accumulated doses play a critical role in LDR-induced renal protection; either too low or too high of a dose will reduce the efficiency. Although potential long-term mild toxic effects should be considered for diabetic patients who may develop severe DN earlier than the occurrence of the late adverse effects of LDR. Furthermore, given that whole body exposure to LDR is so easy and convenient to perform, this method may provide a novel therapeutic approach for diabetic patients in the near future.

## References

[1] Burrows NR, Li Y, Williams DE (2008) Racial and ethnic differences in trends of end-stage renal disease: United States, 1995 to 2005. Adv Chronic Kidney Dis 15: 147–152. 18334239 10.1053/j.ackd.2008.01.002

[2] Iseki K (2008) Chronic kidney disease in Japan. Intern Med 47: 681–689. 18421182 10.2169/internalmedicine.47.0906

[3] Meguro S, Tomita M, Kabeya Y, Katsuki T, Oikawa Y, et al (2012) Factors Associated with the Decline of Kidney Function Differ among eGFR Strata in Subjects with Type 2 Diabetes Mellitus. Int J Endocrinol 2012: 687867. 23316229 10.1155/2012/687867 PMC3536318

[4] Heerspink HJ, de Zeeuw D (2011) The kidney in type 2 diabetes therapy. Rev Diabet Stud 8: 392–402. 22262076 10.1900/RDS.2011.8.392 PMC3280673

[5] Franceschini N, Shara NM, Wang H, Voruganti VS, Laston S, et al (2012) The association of genetic variants of type 2 diabetes with kidney function. Kidney Int 82: 220–225. 22513821 10.1038/ki.2012.107 PMC3664521

[6] Toblli JE, Ferrini MG, Cao G, Vernet D, Angerosa M, et al (2009) Antifibrotic effects of pioglitazone on the kidney in a rat model of type 2 diabetes mellitus. Nephrol Dial Transplant 24: 2384–2391. 19297362 10.1093/ndt/gfp103 PMC2727296

[7] McKay GJ, Savage DA, Patterson CC, Lewis G, McKnight AJ, et al (2013) Association analysis of dyslipidemia-related genes in diabetic nephropathy. PLoS One 8: e58472. 23555584 10.1371/journal.pone.0058472 PMC3608831

[8] Jisieike-Onuigbo NN, Unuigbe EI, Kalu OA, Oguejiofor CO, Onuigbo PC (2011) Prevalence of dyslipidemia among adult diabetic patients with overt diabetic nephropathy in Anambra state south-east Nigeria. Niger J Clin Pract 14: 171–175. 21860134 10.4103/1119-3077.84009

[9] Murea M, Freedman BI, Parks JS, Antinozzi PA, Elbein SC, et al (2010) Lipotoxicity in diabetic nephropathy: the potential role of fatty acid oxidation. Clin J Am Soc Nephrol 5: 2373–2379. 21051750 10.2215/CJN.08160910

[10] Bonnet F, Cooper ME (2000) Potential influence of lipids in diabetic nephropathy: insights from experimental data and clinical studies. Diabetes Metab 26: 254–264. 11011217

[11] Hirano T (1999) Lipoprotein abnormalities in diabetic nephropathy. Kidney Int Suppl 71: S22–24. 10.1046/j.1523-1755.1999.07106.x 10412730

[12] Bobulescu IA (2010) Renal lipid metabolism and lipotoxicity. Curr Opin Nephrol Hypertens 19: 393–402. 20489613 10.1097/MNH.0b013e32833aa4ac PMC3080272

[13] Harjai KJ (1999) Potential new cardiovascular risk factors: left ventricular hypertrophy, homocysteine, lipoprotein(a), triglycerides, oxidative stress, and fibrinogen. Ann Intern Med 131: 376–386. 10475891 10.7326/0003-4819-131-5-199909070-00009

[14] Petersen KF, Shulman GI (2006) Etiology of insulin resistance. Am J Med 119: S10–16. 16563942 10.1016/j.amjmed.2006.01.009 PMC2995525

[15] Qatanani M, Lazar MA (2007) Mechanisms of obesity-associated insulin resistance: many choices on the menu. Genes Dev 21: 1443–1455. 17575046 10.1101/gad.1550907

[16] Gum RJ, Gaede LL, Koterski SL, Heindel M, Clampit JE, et al (2003) Reduction of protein tyrosine phosphatase 1B increases insulin-dependent signaling in ob/ob mice. Diabetes 52: 21–28. 12502489 10.2337/diabetes.52.1.21

[17] Miller AW, Dimitropoulou C, Han G, White RE, Busija DW, et al (2001) Epoxyeicosatrienoic acid-induced relaxation is impaired in insulin resistance. Am J Physiol Heart Circ Physiol 281: H1524–1531. 11557540 10.1152/ajpheart.2001.281.4.H1524

[18] Piqueras L, Reynolds AR, Hodivala-Dilke KM, Alfranca A, Redondo JM, et al (2007) Activation of PPARbeta/delta induces endothelial cell proliferation and angiogenesis. Arterioscler Thromb Vasc Biol 27: 63–69. 17068288 10.1161/01.ATV.0000250972.83623.61

[19] Yang RL, Shi YH, Hao G, Li W, Le GW (2008) Increasing Oxidative Stress with Progressive Hyperlipidemia in Human: Relation between Malondialdehyde and Atherogenic Index. J Clin Biochem Nutr 43: 154–158. 19015749 10.3164/jcbn.2008044 PMC2581765

[20] Rutledge JC, Ng KF, Aung HH, Wilson DW (2010) Role of triglyceride-rich lipoproteins in diabetic nephropathy. Nat Rev Nephrol 6: 361–370. 20440276 10.1038/nrneph.2010.59

[21] Pradhan AD, Manson JE, Rifai N, Buring JE, Ridker PM (2001) C-reactive protein, interleukin 6, and risk of developing type 2 diabetes mellitus. JAMA 286: 327–334. 11466099 10.1001/jama.286.3.327

[22] Skrha J (2003) Pathogenesis of angiopathy in diabetes. Acta Diabetol 40 Suppl 2S324–329. 14704862 10.1007/s00592-003-0113-z

[23] Zhang C, Tan Y, Guo W, Li C, Ji S, et al (2009) Attenuation of diabetes-induced renal dysfunction by multiple exposures to low-dose radiation is associated with the suppression of systemic and renal inflammation. Am J Physiol Endocrinol Metab 297: E1366–1377. 19789291 10.1152/ajpendo.00478.2009

[24] Aunapuu M, Pechter U, Gerskevits E, Marjamagi MM, Suuroja S, et al (2004) Low-dose radiation modifies the progression of chronic renal failure. Ann Anat 186: 277–282. 15255305 10.1016/S0940-9602(04)80017-7

[25] van Kleef EM, Zurcher C, Oussoren YG, Te Poele JA, van der Valk MA, et al (2000) Long-term effects of total-body irradiation on the kidney of Rhesus monkeys. Int J Radiat Biol 76: 641–648. 10866286 10.1080/095530000138303

[26] Xing X, Zhang C, Shao M, Tong Q, Zhang G, et al (2012) Low-dose radiation activates Akt and Nrf2 in the kidney of diabetic mice: a potential mechanism to prevent diabetic nephropathy. Oxid Med Cell Longev 2012: 291087. 23227273 10.1155/2012/291087 PMC3514845

[27] Gilbert ER, Fu Z, Liu D (2011) Development of a nongenetic mouse model of type 2 diabetes. Exp Diabetes Res 2011: 416254. 22164157 10.1155/2011/416254 PMC3226533

[28] Srinivasan K, Viswanad B, Asrat L, Kaul CL, Ramarao P (2005) Combination of high-fat diet-fed and low-dose streptozotocin-treated rat: a model for type 2 diabetes and pharmacological screening. Pharmacol Res 52: 313–320. 15979893 10.1016/j.phrs.2005.05.004

[29] Zhao Y, Tan Y, Dai J, Li B, Guo L, et al (2011) Exacerbation of diabetes-induced testicular apoptosis by zinc deficiency is most likely associated with oxidative stress, p38 MAPK activation, and p53 activation in mice. Toxicol Lett 200: 100–106. 21078376 10.1016/j.toxlet.2010.11.001

[30] Ji H, Pesce C, Zheng W, Kim J, Zhang Y, et al (2005) Sex differences in renal injury and nitric oxide production in renal wrap hypertension. Am J Physiol Heart Circ Physiol 288: H43–47. 15319201 10.1152/ajpheart.00630.2004

[31] Song Y, Li C, Cai L (2004) Fluvastatin prevents nephropathy likely through suppression of connective tissue growth factor-mediated extracellular matrix accumulation. Exp Mol Pathol 76: 66–75. 14738871 10.1016/j.yexmp.2003.08.002

[32] Zhao W, Ahokas RA, Weber KT, Sun Y (2006) ANG II-induced cardiac molecular and cellular events: role of aldosterone. Am J Physiol Heart Circ Physiol 291: H336–343. 16489102 10.1152/ajpheart.01307.2005

[33] Marney AM, Brown NJ (2007) Aldosterone and end-organ damage. Clin Sci (Lond) 113: 267–278. 17683282 10.1042/CS20070123

[34] Adair LS (2008) Child and adolescent obesity: epidemiology and developmental perspectives. Physiol Behav 94: 8–16. 18191968 10.1016/j.physbeh.2007.11.016

[35] Athyros VG, Mitsiou EK, Tziomalos K, Karagiannis A, Mikhailidis DP (2010) Impact of managing atherogenic dyslipidemia on cardiovascular outcome across different stages of diabetic nephropathy. Expert Opin Pharmacother 11: 723–730. 20210681 10.1517/14656560903575654

[36] Kim MY, Lim JH, Youn HH, Hong YA, Yang KS, et al (2013) Resveratrol prevents renal lipotoxicity and inhibits mesangial cell glucotoxicity in a manner dependent on the AMPK-SIRT1-PGC1alpha axis in db/db mice. Diabetologia 56: 204–217. 23090186 10.1007/s00125-012-2747-2

[37] Wahba IM, Mak RH (2007) Obesity and obesity-initiated metabolic syndrome: mechanistic links to chronic kidney disease. Clin J Am Soc Nephrol 2: 550–562. 17699463 10.2215/CJN.04071206

[38] Chung HW, Lim JH, Kim MY, Shin SJ, Chung S, et al (2012) High-fat diet-induced renal cell apoptosis and oxidative stress in spontaneously hypertensive rat are ameliorated by fenofibrate through the PPARalpha-FoxO3a-PGC-1alpha pathway. Nephrol Dial Transplant 27: 2213–2225. 22076434 10.1093/ndt/gfr613

[39] Lin J, Glynn RJ, Rifai N, Manson JE, Ridker PM, et al (2008) Inflammation and progressive nephropathy in type 1 diabetes in the diabetes control and complications trial. Diabetes Care 31: 2338–2343. 18796620 10.2337/dc08-0277 PMC2584192

[40] Navarro-Gonzalez JF, Mora-Fernandez C (2008) The role of inflammatory cytokines in diabetic nephropathy. J Am Soc Nephrol 19: 433–442. 18256353 10.1681/ASN.2007091048

[41] Rivero A, Mora C, Muros M, Garcia J, Herrera H, et al (2009) Pathogenic perspectives for the role of inflammation in diabetic nephropathy. Clin Sci (Lond) 116: 479–492. 19200057 10.1042/CS20080394

[42] Pinar B, Lara PC, Lloret M, Bordon E, Nunez MI, et al (2007) Radiation-induced DNA damage as a predictor of long-term toxicity in locally advanced breast cancer patients treated with high-dose hyperfractionated radical radiotherapy. Radiat Res 168: 415–422. 17903032 10.1667/RR0746.1

[43] Hellman B, Brodin D, Andersson M, Dahlman-Wright K, Isacsson U, et al (2005) Radiation-induced DNA-damage and gene expression profiles in human lung cancer cells with different radiosensitivity. Exp Oncol 27: 102–107. 15995626

[44] Popanda O, Ebbeler R, Twardella D, Helmbold I, Gotzes F, et al (2003) Radiation-induced DNA damage and repair in lymphocytes from breast cancer patients and their correlation with acute skin reactions to radiotherapy. Int J Radiat Oncol Biol Phys 55: 1216–1225. 12654430 10.1016/s0360-3016(02)04415-2

[45] Palyvoda O, Mukalov I, Polanska J, Wygoda A, Drobot L, et al (2002) Radiation-induced DNA damage and its repair in lymphocytes of patients with head and neck cancer and healthy donors. Anticancer Res 22: 1721–1725. 12168860

[46] Cai L (1999) Research of the adaptive response induced by low-dose radiation: where have we been and where should we go? Hum Exp Toxicol 18: 419–425. 10454070 10.1191/096032799678840291

[47] de Toledo SM, Asaad N, Venkatachalam P, Li L, Howell RW, et al (2006) Adaptive responses to low-dose/low-dose-rate gamma rays in normal human fibroblasts: the role of growth architecture and oxidative metabolism. Radiat Res 166: 849–857. 17149977 10.1667/RR0640.1

[48] Kim CS, Kim JK, Nam SY, Yang KH, Jeong M, et al (2007) Low-dose radiation stimulates the proliferation of normal human lung fibroblasts via a transient activation of Raf and Akt. Mol Cells 24: 424–430. 18182859

[49] Liu G, Gong P, Bernstein LR, Bi Y, Gong S, et al (2007) Apoptotic cell death induced by low-dose radiation in male germ cells: hormesis and adaptation. Crit Rev Toxicol 37: 587–605. 17674213 10.1080/10408440701493061

[50] Pathak CM, Avti PK, Kumar S, Khanduja KL, Sharma SC (2007) Whole body exposure to low-dose gamma radiation promotes kidney antioxidant status in Balb/c mice. J Radiat Res 48: 113–120. 17339750 10.1269/jrr.06063

[51] Srinivasan K, Ramarao P (2007) Animal models in type 2 diabetes research: an overview. Indian J Med Res 125: 451–472. 17496368

[52] Luo J, Quan J, Tsai J, Hobensack CK, Sullivan C, et al (1998) Nongenetic mouse models of non-insulin-dependent diabetes mellitus. Metabolism 47: 663–668. 9627363 10.1016/s0026-0495(98)90027-0

[53] Gaipl US, Meister S, Lodermann B, Rodel F, Fietkau R, et al (2009) Activation-induced cell death and total Akt content of granulocytes show a biphasic course after low-dose radiation. Autoimmunity 42: 340–342. 19811295 10.1080/08916930902831233

[54] Rane MJ, Song Y, Jin S, Barati MT, Wu R, et al (2010) Interplay between Akt and p38 MAPK pathways in the regulation of renal tubular cell apoptosis associated with diabetic nephropathy. Am J Physiol Renal Physiol 298: F49–61. 19726550 10.1152/ajprenal.00032.2009 PMC2806120

[55] Lee S, Chanoit G, McIntosh R, Zvara DA, Xu Z (2009) Molecular mechanism underlying Akt activation in zinc-induced cardioprotection. Am J Physiol Heart Circ Physiol 297: H569–575. 19525380 10.1152/ajpheart.00293.2009 PMC2724202

[56] Matheny RW Jr, Adamo ML (2010) PI3K p110 alpha and p110 beta have differential effects on Akt activation and protection against oxidative stress-induced apoptosis in myoblasts. Cell Death Differ 17: 677–688. 19834495 10.1038/cdd.2009.150 PMC2839024

[57] Zhao H, Xu S, Wang Z, Li Y, Guo W, et al (2010) Repetitive exposures to low-dose X-rays attenuate testicular apoptotic cell death in streptozotocin-induced diabetes rats. Toxicol Lett 192: 356–364. 19931367 10.1016/j.toxlet.2009.11.011

[58] Tan Y, Ichikawa T, Li J, Si Q, Yang H, et al (2011) Diabetic downregulation of Nrf2 activity via ERK contributes to oxidative stress-induced insulin resistance in cardiac cells in vitro and in vivo. Diabetes 60: 625–633. 21270272 10.2337/db10-1164 PMC3028364

[59] Forbes JM, Coughlan MT, Cooper ME (2008) Oxidative stress as a major culprit in kidney disease in diabetes. Diabetes 57: 1446–1454. 18511445 10.2337/db08-0057

[60] Bagby SP (2007) Diabetic nephropathy and proximal tubule ROS: challenging our glomerulocentricity. Kidney Int 71: 1199–1202. 17554351 10.1038/sj.ki.5002286

[61] Tan AL, Forbes JM, Cooper ME (2007) AGE, RAGE, and ROS in diabetic nephropathy. Semin Nephrol 27: 130–143. 17418682 10.1016/j.semnephrol.2007.01.006

[62] Soetikno V, Watanabe K, Sari FR, Harima M, Thandavarayan RA, et al (2011) Curcumin attenuates diabetic nephropathy by inhibiting PKC-alpha and PKC-beta1 activity in streptozotocin-induced type I diabetic rats. Mol Nutr Food Res 55: 1655–1665. 22045654 10.1002/mnfr.201100080

[63] Li B, Liu S, Miao L, Cai L (2012) Prevention of diabetic complications by activation of Nrf2: diabetic cardiomyopathy and nephropathy. Exp Diabetes Res 2012: 216512. 22645602 10.1155/2012/216512 PMC3356887

[64] Cui W, Li B, Bai Y, Miao X, Chen Q, et al (2013) Potential role for Nrf2 activation in the therapeutic effect of MG132 on diabetic nephropathy in OVE26 diabetic mice. Am J Physiol Endocrinol Metab 304: E87–99. 23132297 10.1152/ajpendo.00430.2012

[65] Calabrese EJ (2013) Low doses of radiation can enhance insect lifespans. Biogerontology 14: 365–381. 23793937 10.1007/s10522-013-9436-5

[66] Blokhina O, Virolainen E, Fagerstedt KV (2003) Antioxidants, oxidative damage and oxygen deprivation stress: a review. Ann Bot 91 Spec No: 179–194. 10.1093/aob/mcf118 PMC4244988 12509339

[67] San Miguel SM, Opperman LA, Allen EP, Svoboda KK (2011) Reactive oxygen species and antioxidant defense mechanisms in the oral cavity: a literature review. Compend Contin Educ Dent 32: E10–15. 23738797

[68] Karihtala P, Soini Y (2007) Reactive oxygen species and antioxidant mechanisms in human tissues and their relation to malignancies. APMIS 115: 81–103. 17295675 10.1111/j.1600-0463.2007.apm_514.x

[69] Mantovani G, Maccio A, Madeddu C, Mura L, Massa E, et al (2003) Reactive oxygen species, antioxidant mechanisms, and serum cytokine levels in cancer patients: impact of an antioxidant treatment. J Environ Pathol Toxicol Oncol 22: 17–28. 12678402 10.1615/jenvpathtoxoncol.v22.i1.20

