# Expression of Concern: Multiple Low-Dose Radiation Prevents Type 2 Diabetes-Induced Renal Damage through Attenuation of Dyslipidemia and Insulin Resistance and Subsequent Renal Inflammation and Oxidative Stress

**DOI:** 10.1371/journal.pone.0327042

**Published:** 2025-06-30

**Authors:** The PLOS One Editors

After this article [1] was published, concerns were raised regarding results presented in Figs 4-5 and 7-8.

**Fig 4:**
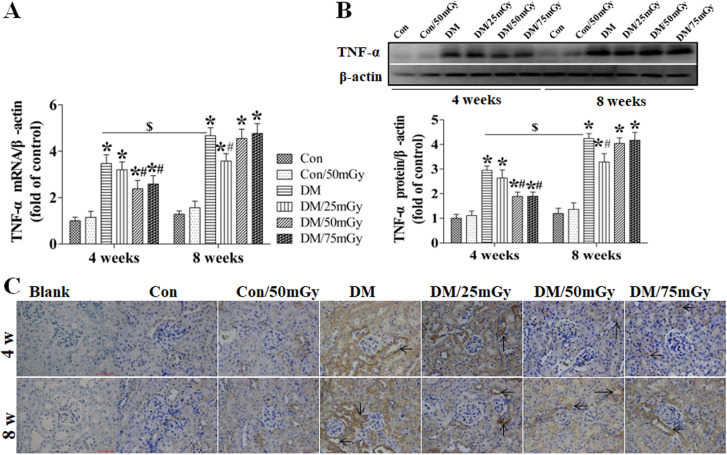
Effect of LDR on renal TNF- **α** **levels in type 2 diabetic mice.** Renal tissues from different groups were collected at the indicated times for measuring TNF-α expression at the mRNA (A) and protein (B) levels with RT-PCR and western blotting, respectively. **(C)** The location of TNF-α in the kidney was detected by immunohistochemical staining, at 400 × magnification. Data are presented as means ± SEM. n = 9 in diabetic group and n = 8 in each other group. *p < 0.05 vs. the corresponding control group; #*p* < 0.05 vs. the corresponding DM group; $, *p* < 0.05 vs. DM group at the 4-week time-point.

**Fig 5:**
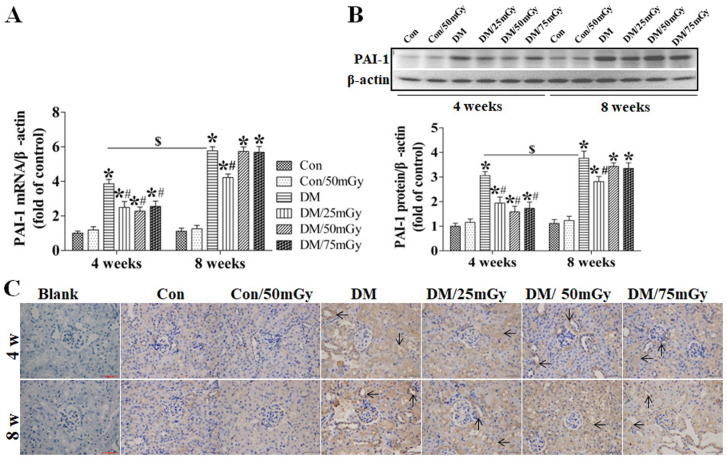
Effect of LDR on renal PAI-1 levels in type 2 diabetic mice. Renal tissues from different groups were collected at the indicated times for measuring PAI-1 expression at the mRNA (A) and protein (B) levels with RT-PCR and western blotting, respectively. **(C)** The location of PAI-1 in the kidney was detected by immunohistochemical staining, at 400 × magnification. Data are presented as means ± SEM. n = 9 in diabetic group and n = 8 in each other group. *p < 0.05 vs. the corresponding control group; #*p* < 0.05 vs. the corresponding DM group; $, *p* < 0.05 vs. DM group at the 4-week time-point.

**Fig 7:**
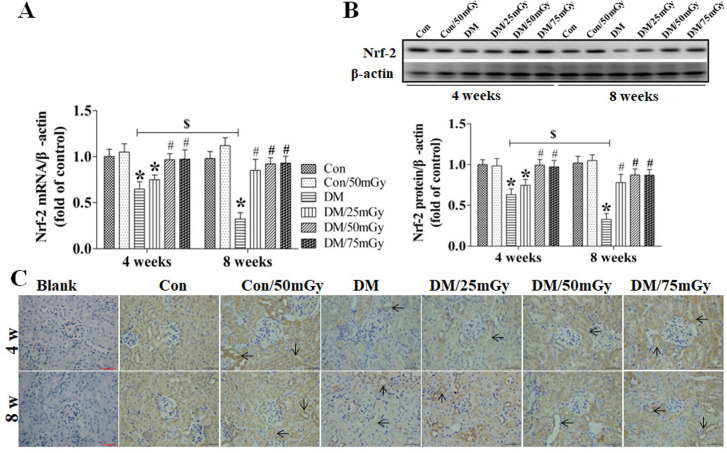
Effect of LDR on renal Nrf-2 levels in type 2 diabetic mice. Renal tissues from different groups were collected at the indicated times for measuring Nrf-2 expression at the mRNA (A) and protein (B) levels with RT-PCR and western blotting, respectively. **(C)** The location of Nrf-2 in the kidney was detected by immunohistochemical staining, at 400 × magnification. Data are presented as means ± SEM. n = 9 in diabetic group and n = 8 in each other group. *p < 0.05 vs. the corresponding control group; #*p* < 0.05 vs. the corresponding DM group; $, *p* < 0.05 vs. DM mice at the 4-week time-point.

**Fig 8:**
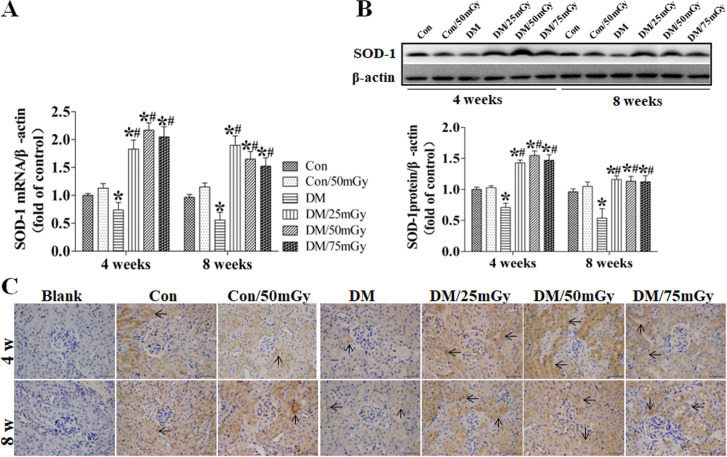
Effect of LDR on renal SOD-1 levels in type 2 diabetic mice. Renal tissues from different groups were collected at the indicated times for measuring SOD-1 expression at the mRNA (A) and protein (B) levels with RT-PCR and western blotting, respectively. **(C)** The location of SOD-1 in the kidney was detected by immunohistochemical staining, at 400 × magnification. Data are presented as means ± SEM. n = 9 in diabetic group and n = 8 in each other group. *p < 0.05 vs. the corresponding control group; #*p* < 0.05 vs. the corresponding DM group; $, p < 0.05 vs. DM group at the 4-week time-point.

Specifically:

• The following panels appear to partially overlap despite representing different experimental conditions:
  - ◦ Fig 4C DM 4w and DM/25mGy 4w
  - ◦ Fig 5C DM 8w and DM/75mGy 8w
  - ◦ Fig 7C Con/50mGy 4w and Con/50mGy 8w
• The Fig 8C Con/50mGy 4w and DM/75mGy 4w panels appear similar
• The method of euthanasia used for the mice in this study was not described.

The corresponding author CZ stated that errors were made in the preparation of Figs 4, 5, 7, and 8. Specifically, the DM/25mGy 4w panel in Fig 4C, the DM/75mGy 8w panel in Fig 5C, the Con/50mGy 8w panel in Fig 7C and the Con/50mGy 4w panel in Fig 8C are incorrect. They provided updated versions of these Figs in which the correct images are shown and the LDR label in Fig 4C has been updated to Con/50mGy. The original image data underlying these figures are presented in the S7, S9-10, S12-13, and S15-16 Files.

Regarding the method of euthanasia, the corresponding author CZ provided the following response: “After the mice were fully anesthetized to unconsciousness, blood samples were collected from the retro-orbital venous plexus. Subsequently, the mice were humanely euthanized by careful and rapid cervical dislocation to minimize their suffering.”.

In reviewing the quantitative data for Fig 1 (S1 File), it was noted that the number of mice in each treatment group for Figs 1A, 1B, 1C, and 1H differed from the number of mice reported in the figure legend for Fig 1 of 8 to 9 mice per group. The corresponding author CZ clarified that the 8–9 mice in the figure legend referred to Figs 1E and 1F only. Regarding Fig 1A, they stated that the Con group initially comprised 32 mice, while the HFD group (high-fat diet group) consisted of 108 mice. However, when the actual measurements were recorded, they stated that 24 mice were selected in the Con group, and 52 mice were selected in the HFD group. They also stated that the mice were selected for measurement using a cage-based sampling strategy by randomly selecting cages each containing 4–6 mice and then repeatedly measuring the selected cages according to the time points. The corresponding author CZ stated that in Figs 1B, 1C and 1H, the number of mice tested in each treatment group was between 3–6 due to the constraints of testing multiple mice within the time points of these experiments. A member of the *PLOS One* Editorial Board reviewed this response, and stated they had no concerns for the number of mice used in Figs 1A, 1B, 1C, and 1H.

During editorial follow-up it was also noted that within the Fig 3B ICAM-1 and Fig 8B SOD-1 panels, lanes 1−6 appear similar, but not identical, to lanes 7−12. Regarding Fig 3B ICAM-1 and Fig 8B SOD-1, the corresponding author CZ stated that the original uncropped image data underlying these results are no longer available.

The available underlying data for Figs 1-5 and Figs 7-9 are provided in the S1-S19 Files.

Due to the extent of the above listed image concerns, PLOS remains concerned regarding the reliability of the published data. The *PLOS One* Editors issue this Expression of Concern to notify readers of the above issues, and to relay the available information and data provided.

## Supporting information

S1 File Available quantitative data underlying Fig 1. (ZIP)

S2 File Image data underlying Fig 2A – folder 1. (ZIP)

S3 File Image data underlying Fig 2A – folder 2. (ZIP)

S4 File Image data underlying Fig 2A – folder 3. (ZIP)

S5 File Available data provided for Fig 3B. (ZIP)

S6 File Available data provided for Fig 4B. (ZIP)

S7 File Image data underlying Fig 4C. (ZIP)

S8 File Available data provided for Fig 5B. (ZIP)

S9 File Image data underlying Fig 5C – folder 1. (ZIP)

S10 File Image data underlying Fig 5C – folder 2. (ZIP)

S11 File Available data provided for Fig 7B. (ZIP)

S12 File Image data underlying Fig 7C – folder 1. (ZIP)

S13 File Image data underlying Fig 7C – folder 2. (ZIP)

S14 File Available data provided for Fig 8B SOD-1 blot. (TIF)

S15 File Image data underlying Fig 8C – folder 1. (ZIP)

S16 File Image data underlying Fig 8C – folder 2. (ZIP)

S17 File Available data provided for Figs 9C and 9D. (ZIP)

S18 File Quantitative data underlying the qPCR results Figs 3, 4, 5, 7, 8. (XLS)

S19 File Quantitative data underlying the western blot results Figs 3, 4, 5, 7, 8. (XLS)
