# Supplementary figures and images for: Expression of Concern: Multiple Low-Dose Radiation Prevents Type 2 Diabetes-Induced Renal Damage through Attenuation of Dyslipidemia and Insulin Resistance and Subsequent Renal Inflammation and Oxidative Stress (part 1 of 3)
Source: PLoS One. 2025 Jun 30;20(6):e0327042. doi: 10.1371/journal.pone.0327042 (PMC12208481; doi:10.1371/journal.pone.0327042)

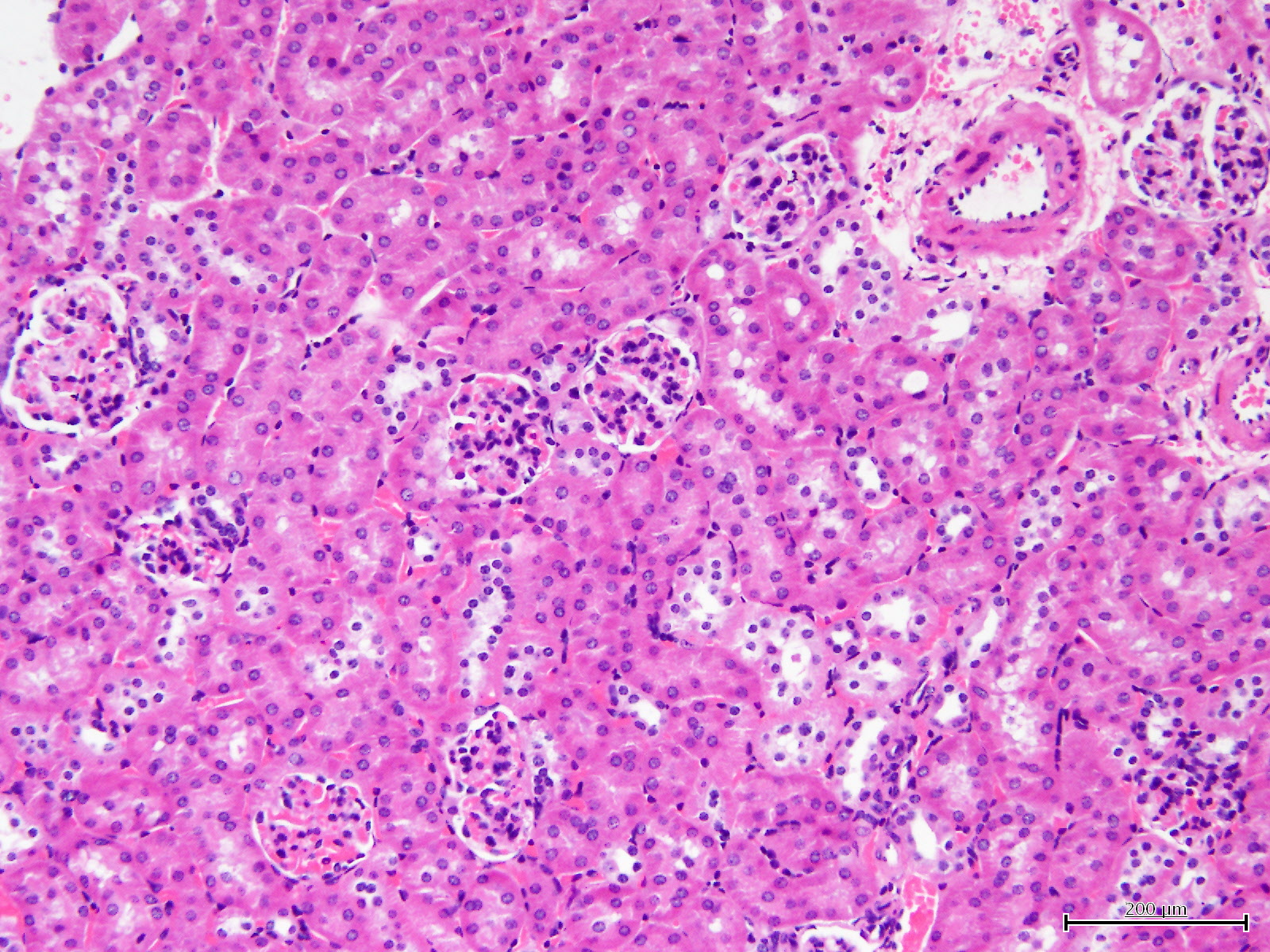

Supplement: S2 File — (ZIP) [file pone.0327042.s002.zip › HE-4w Con/HE-4w Con-1.JPG]

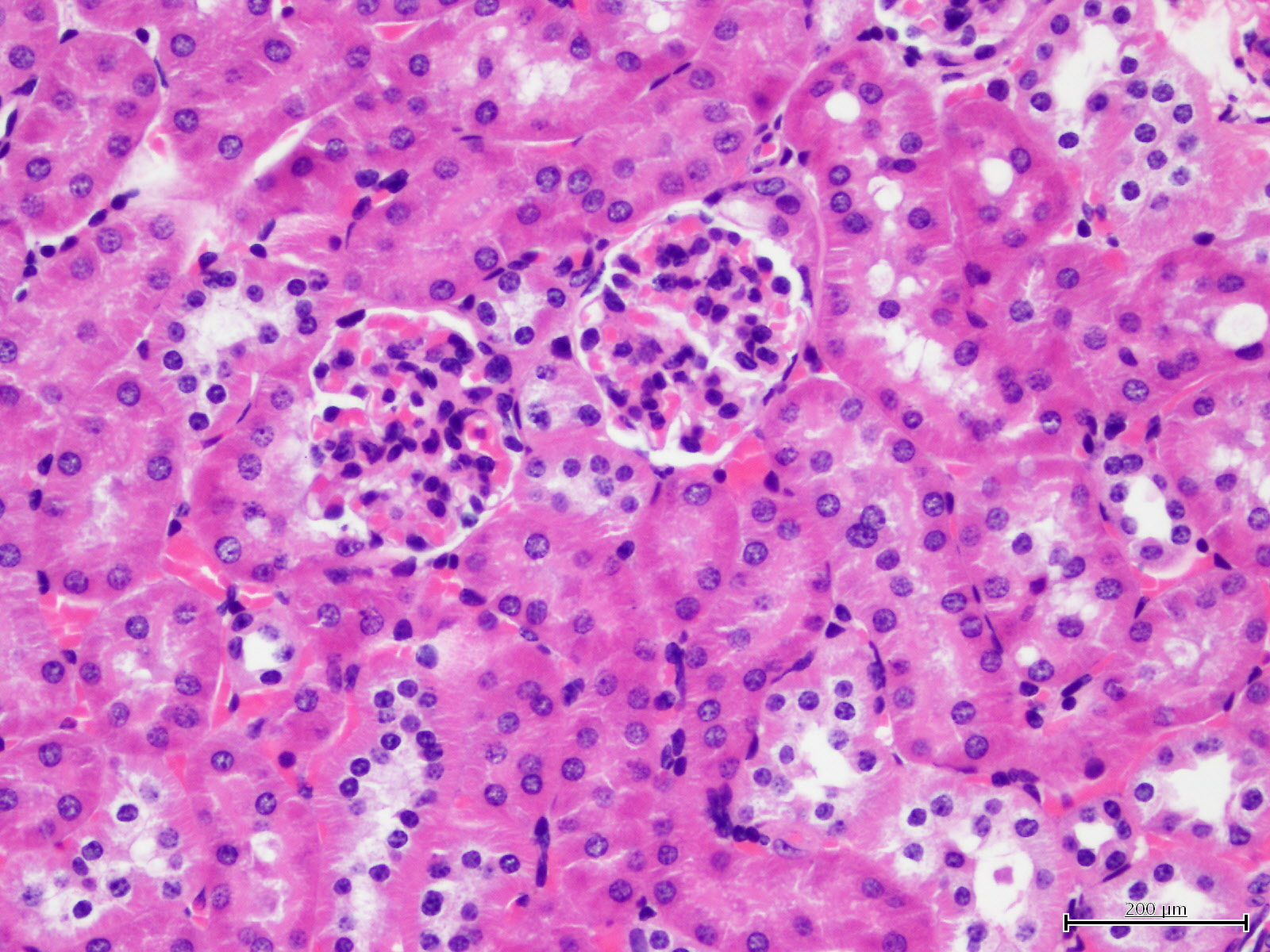

Supplement: S2 File — (ZIP) [file pone.0327042.s002.zip › HE-4w Con/HE-4w Con-2.JPG]

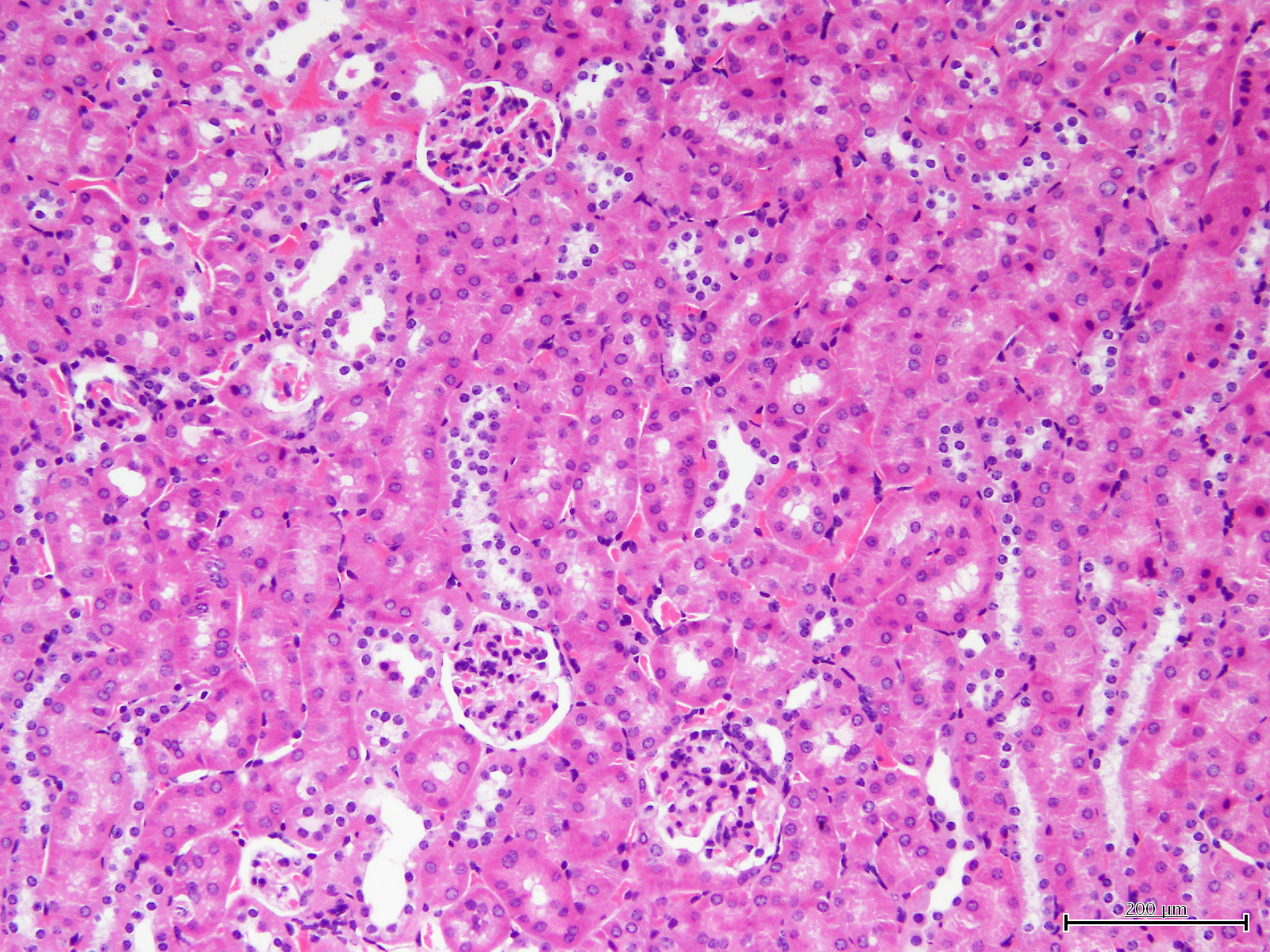

Supplement: S2 File — (ZIP) [file pone.0327042.s002.zip › HE-4w Con/HE-4w Con-3.JPG]

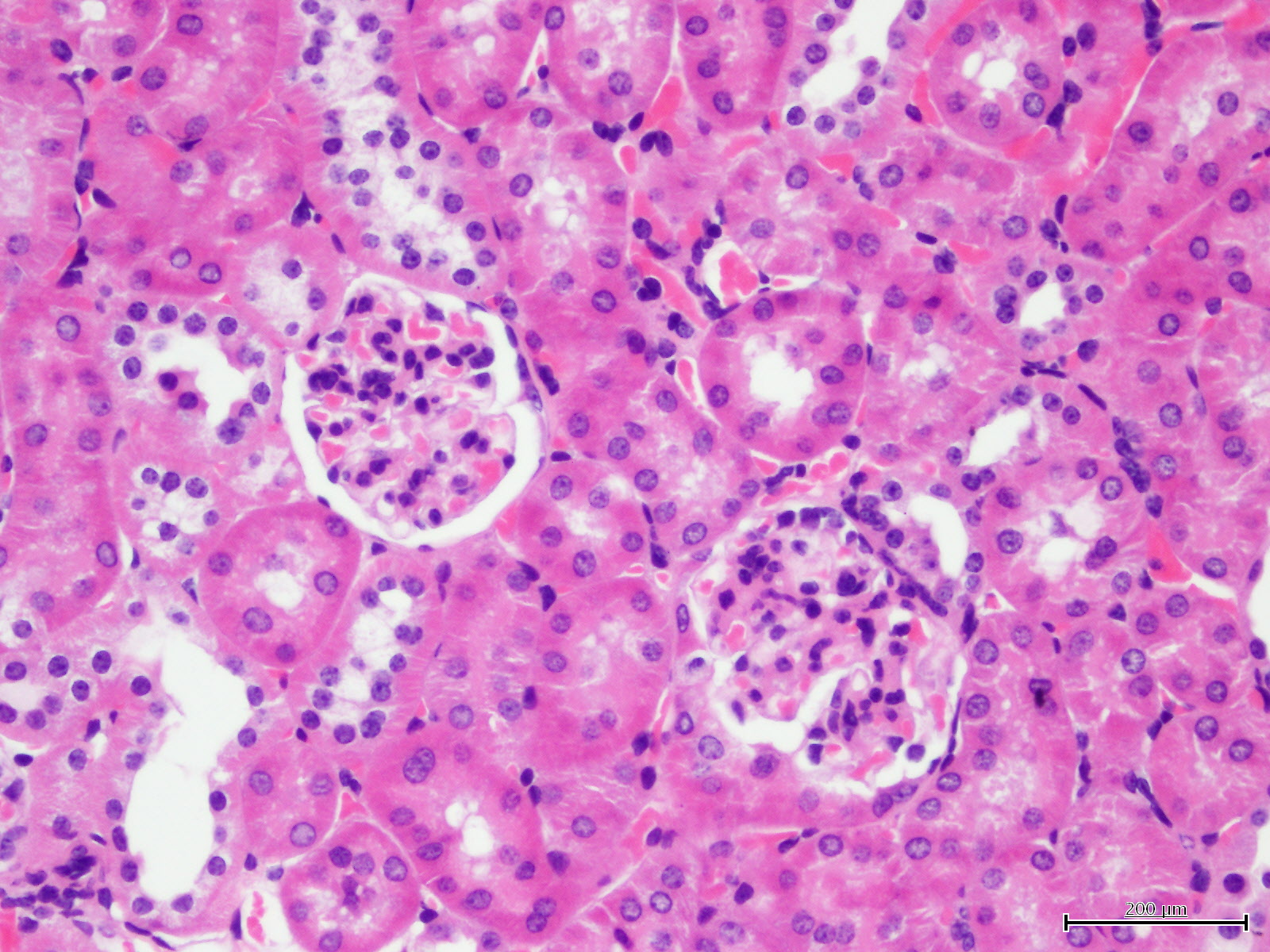

Supplement: S2 File — (ZIP) [file pone.0327042.s002.zip › HE-4w Con/HE-4w Con-4 (Used publication).JPG]

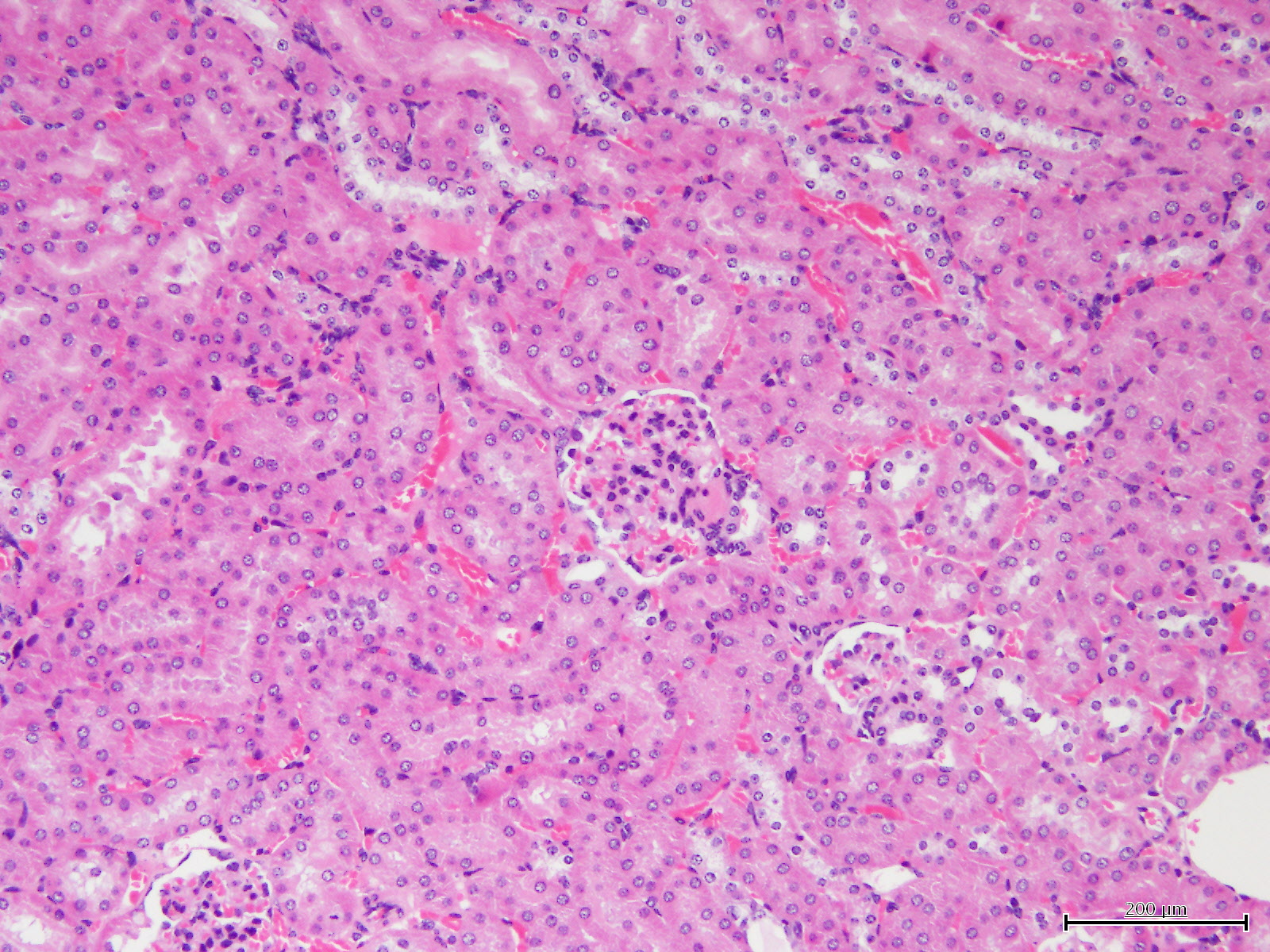

Supplement: S2 File — (ZIP) [file pone.0327042.s002.zip › HE-4w Con 50mGy/4w Con 50mGy-1 20x.JPG]

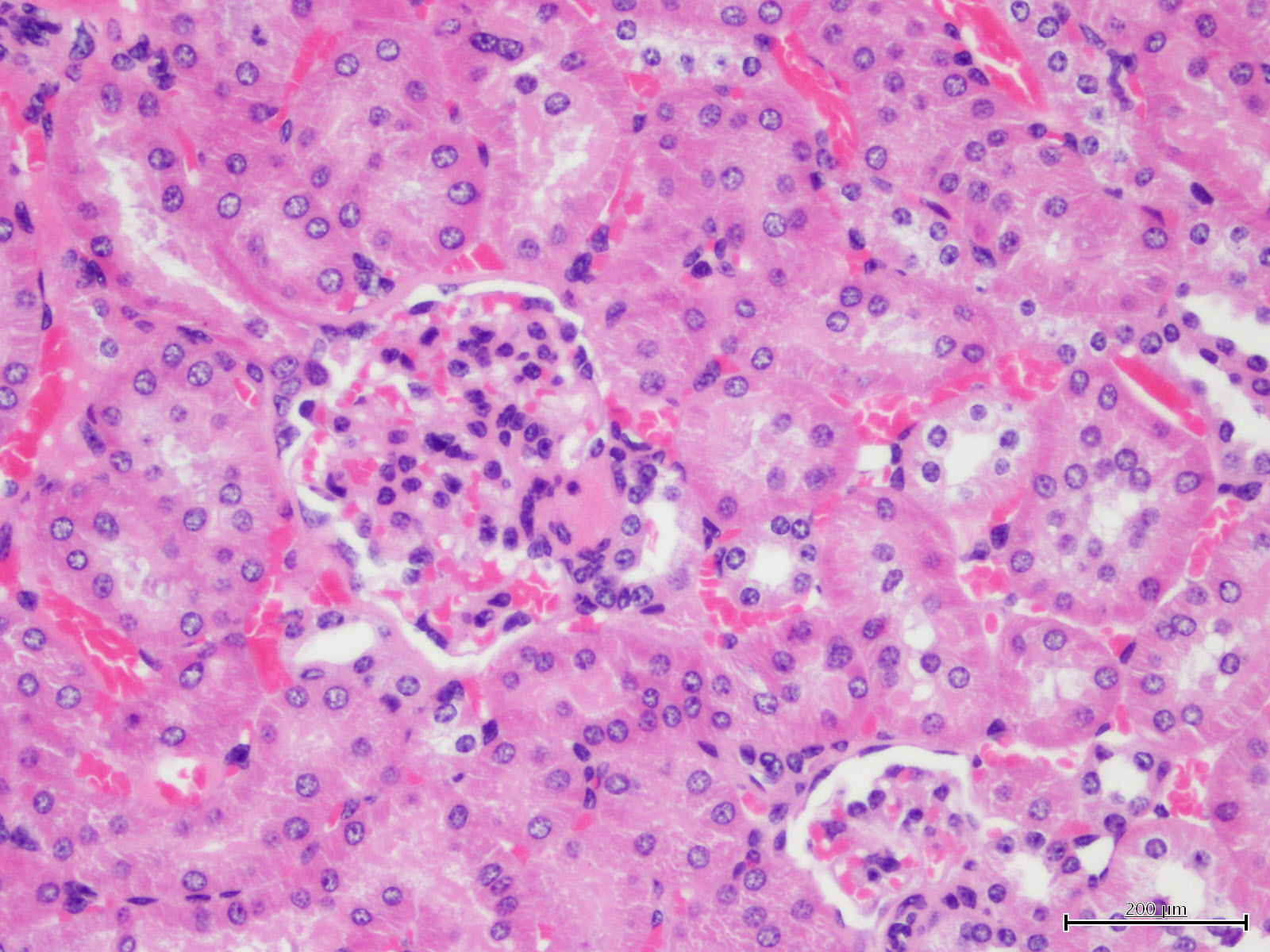

Supplement: S2 File — (ZIP) [file pone.0327042.s002.zip › HE-4w Con 50mGy/4w Con 50mGy-1.JPG]

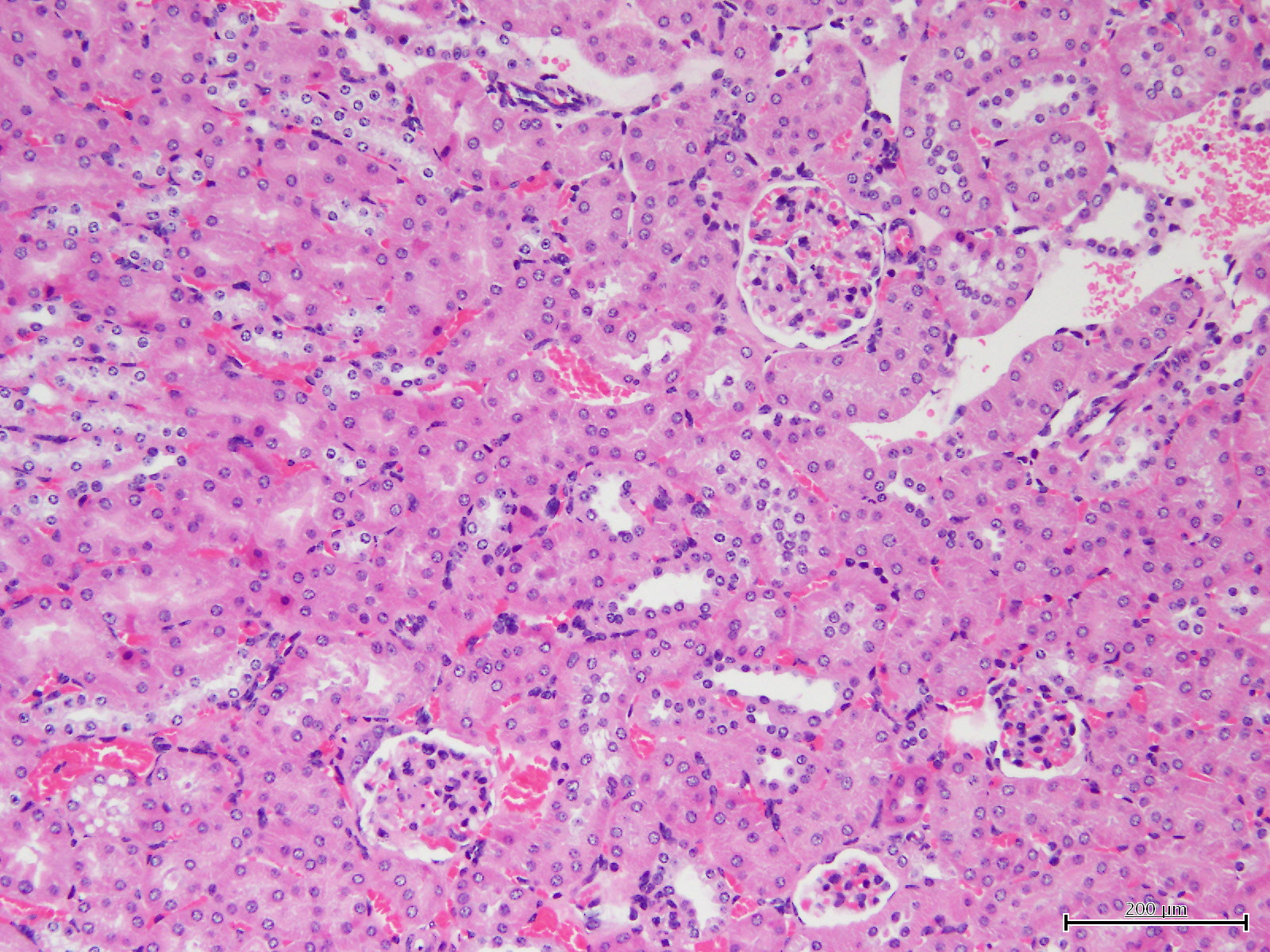

Supplement: S2 File — (ZIP) [file pone.0327042.s002.zip › HE-4w Con 50mGy/4w Con 50mGy-2 20x.JPG]

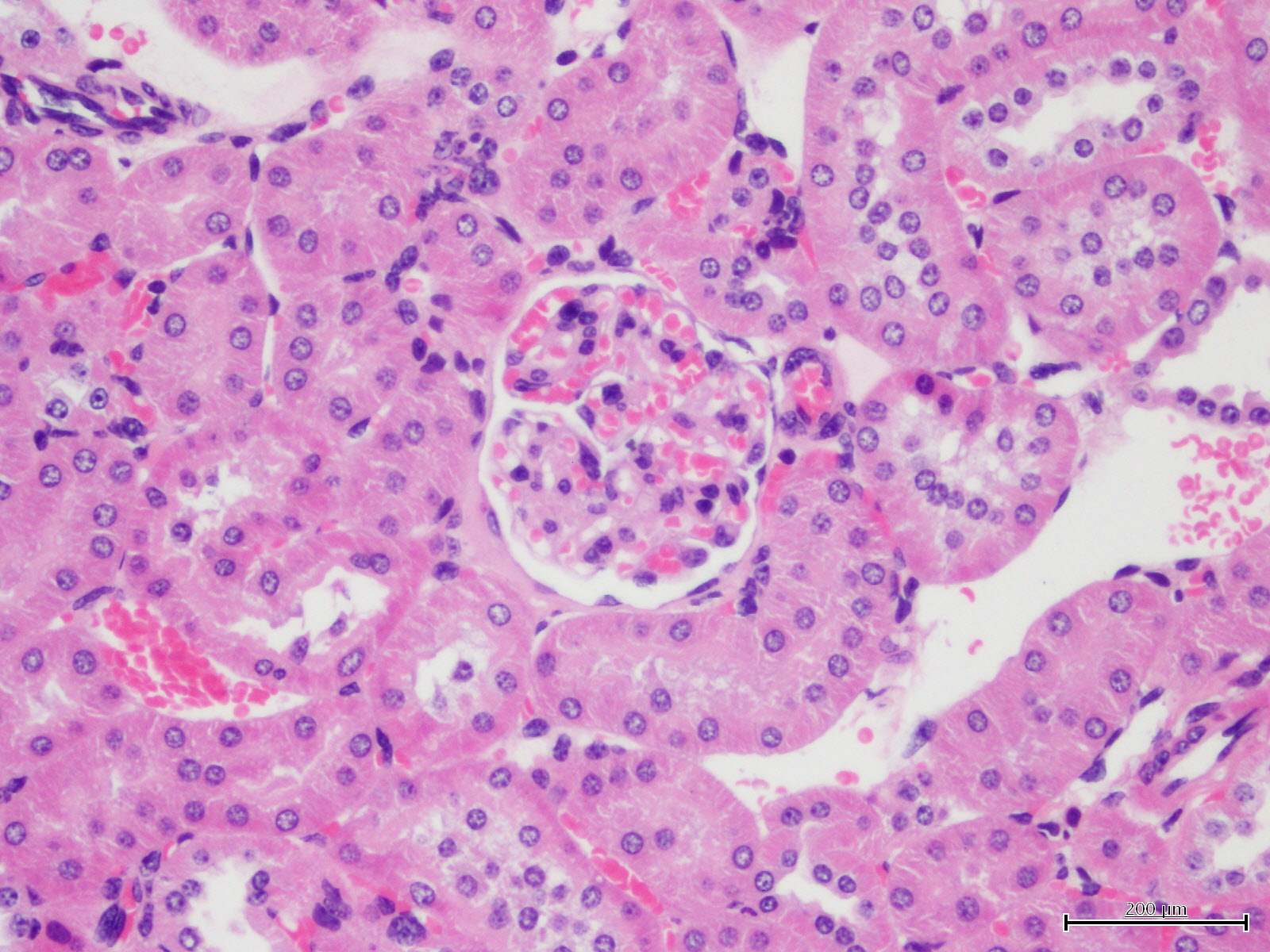

Supplement: S2 File — (ZIP) [file pone.0327042.s002.zip › HE-4w Con 50mGy/4w Con 50mGy-2.JPG]

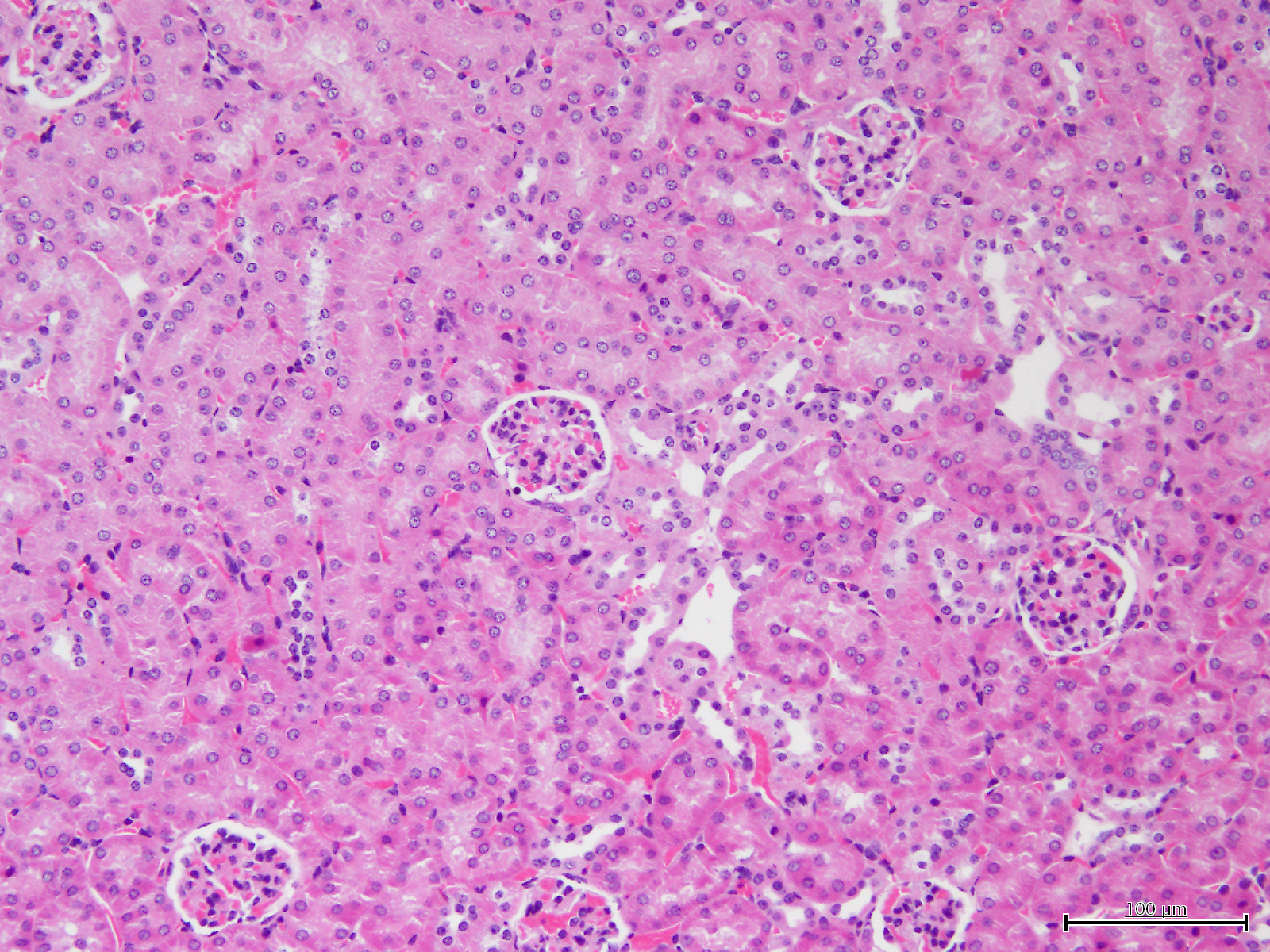

Supplement: S2 File — (ZIP) [file pone.0327042.s002.zip › HE-4w Con 50mGy/4w Con 50mGy-3 20x.TIF]

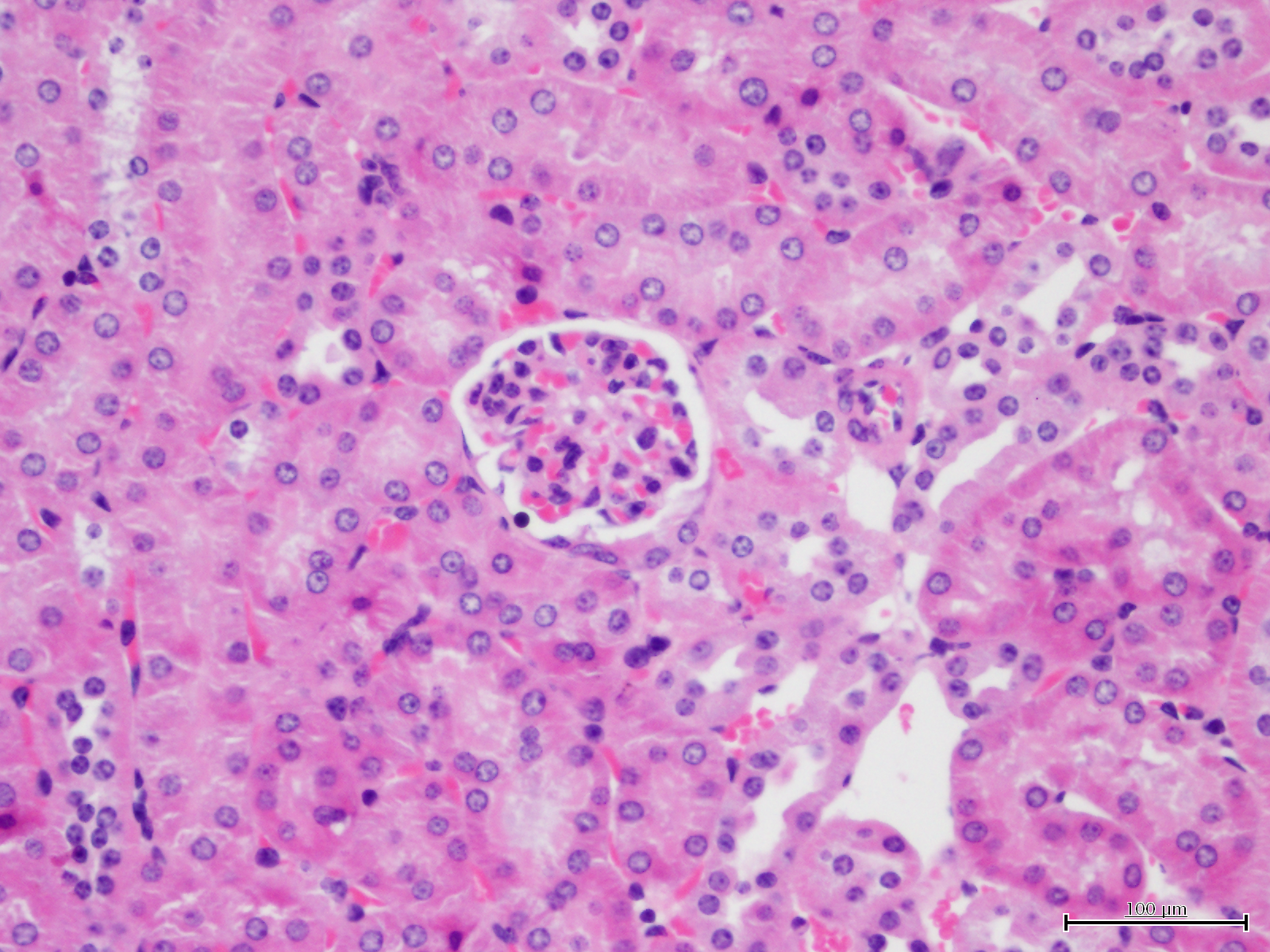

Supplement: S2 File — (ZIP) [file pone.0327042.s002.zip › HE-4w Con 50mGy/4w Con 50mGy-3.TIF]

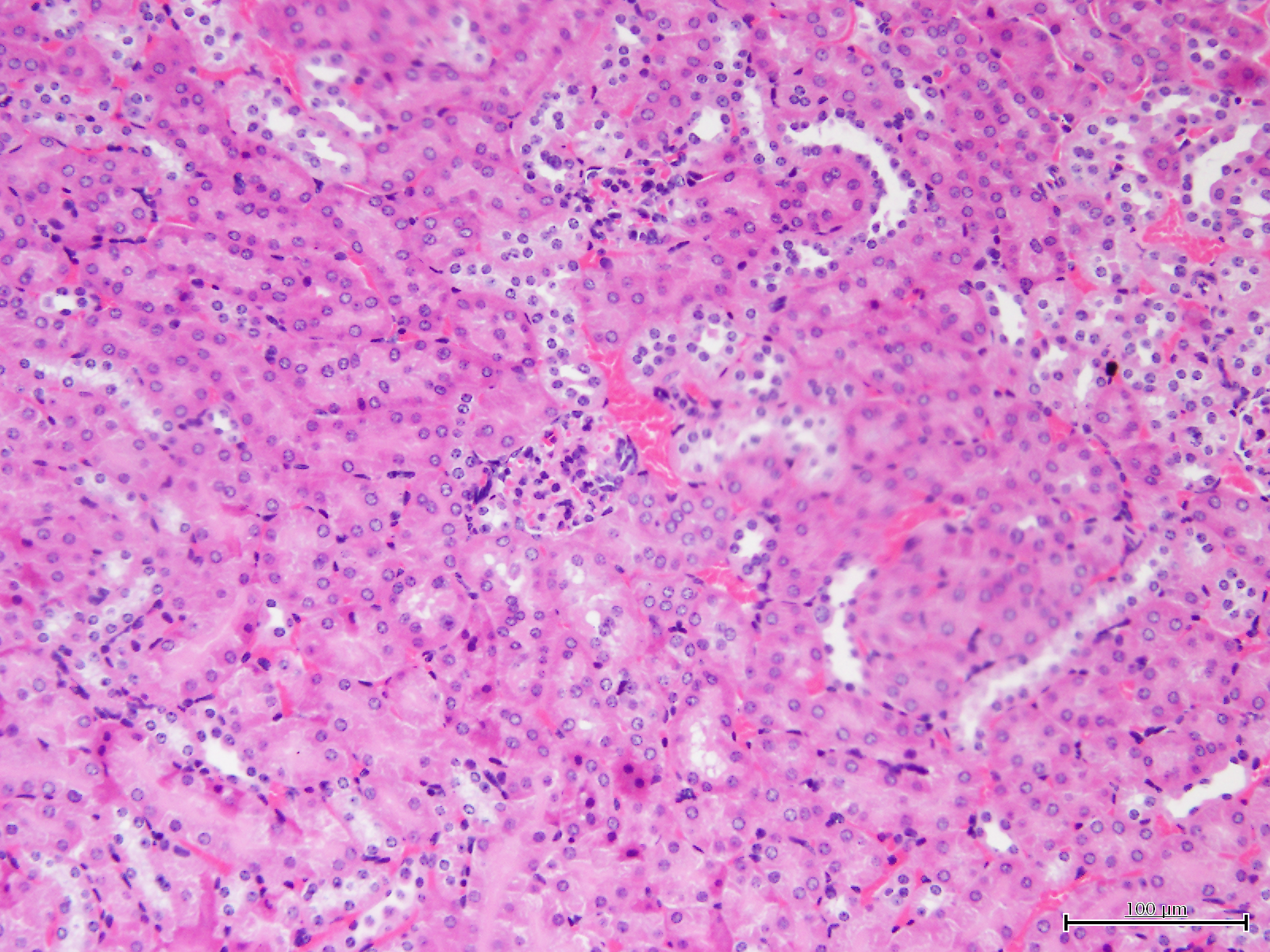

Supplement: S2 File — (ZIP) [file pone.0327042.s002.zip › HE-4w Con 50mGy/4w Con 50mGy-4 20x.TIF]

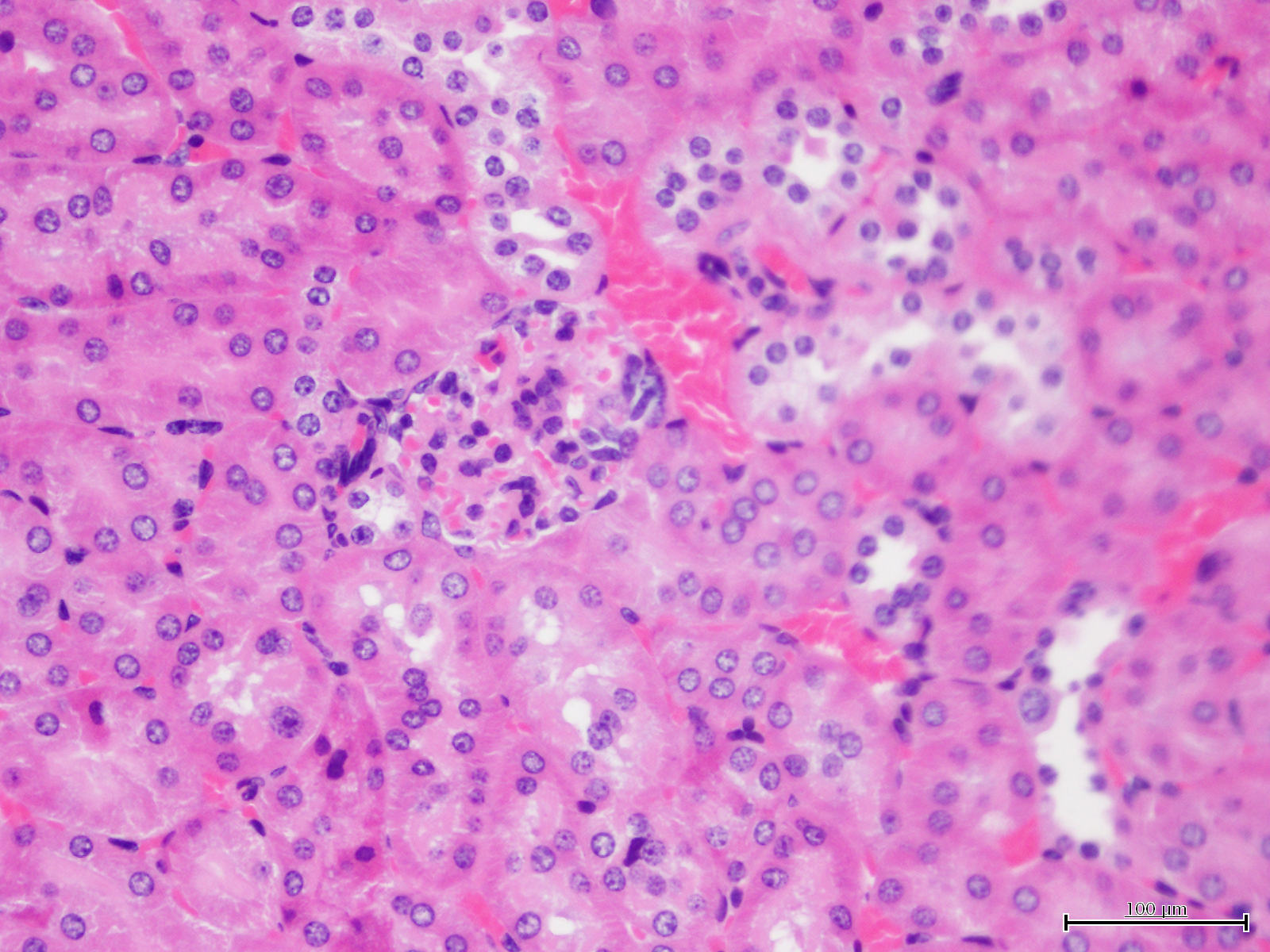

Supplement: S2 File — (ZIP) [file pone.0327042.s002.zip › HE-4w Con 50mGy/4w Con 50mGy-4.TIF]

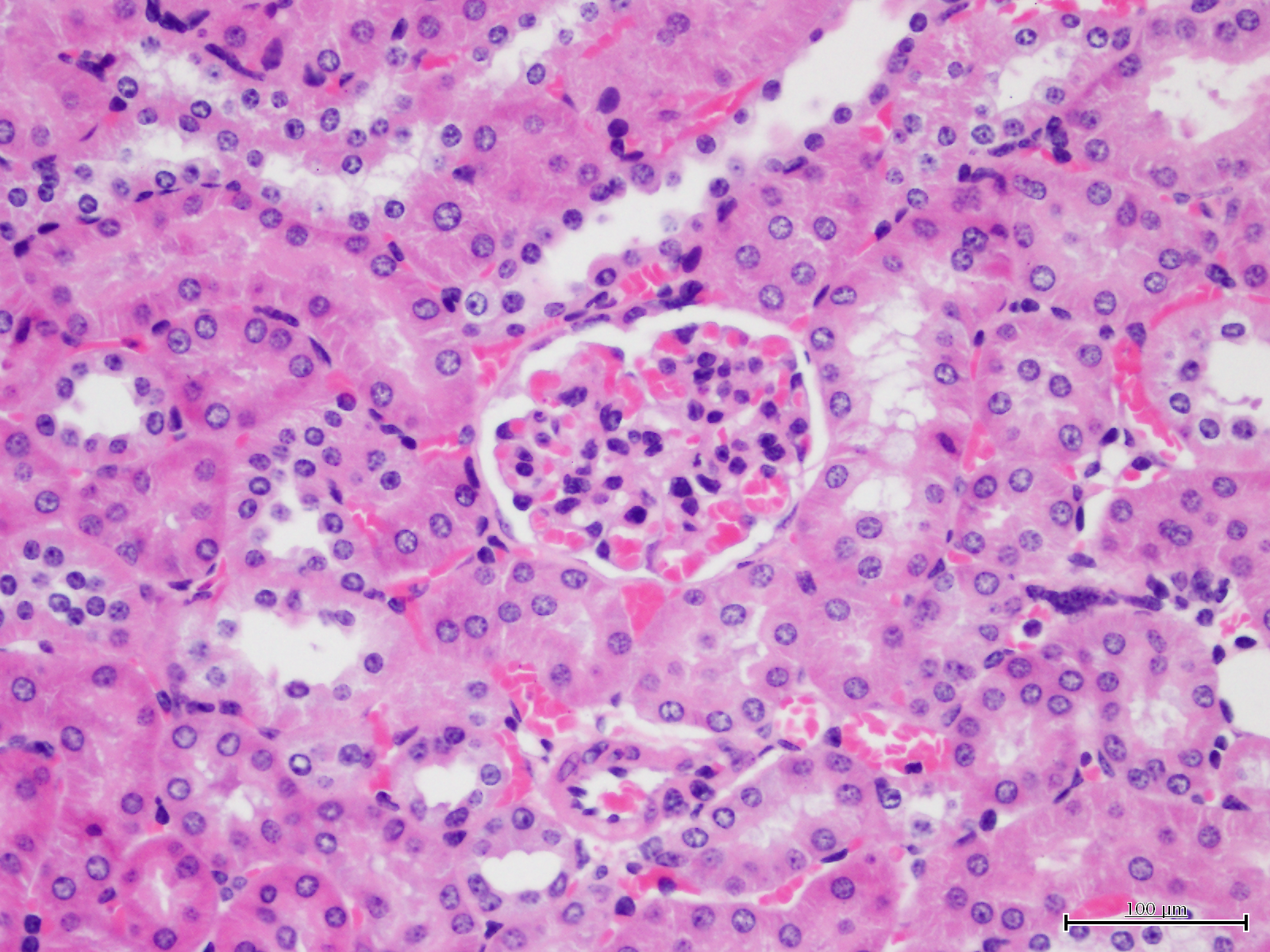

Supplement: S2 File — (ZIP) [file pone.0327042.s002.zip › HE-4w Con 50mGy/4w Con 50mGy-6 (Used publication).TIF]

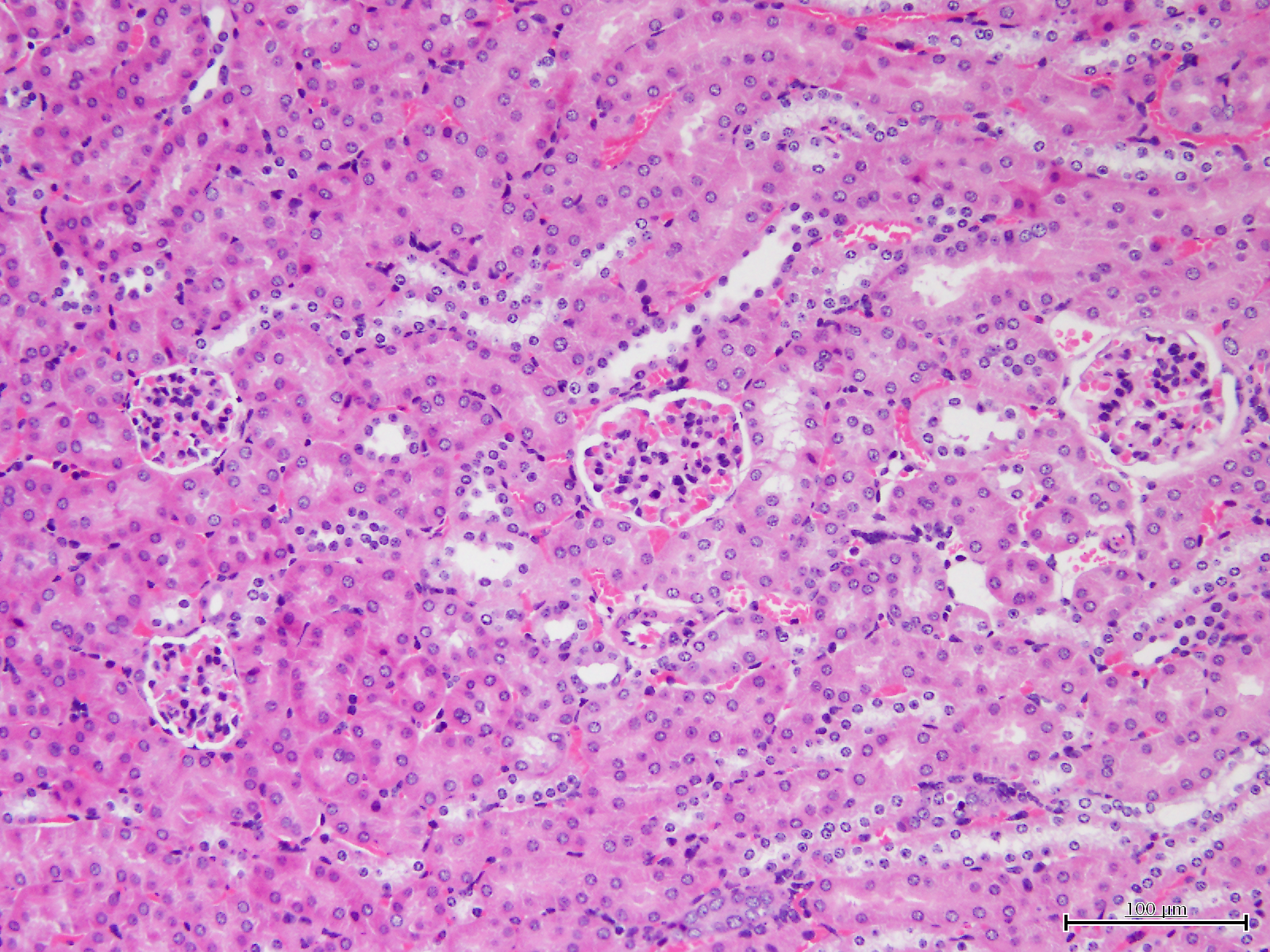

Supplement: S2 File — (ZIP) [file pone.0327042.s002.zip › HE-4w Con 50mGy/4w Con 50mGy-6 20x.TIF]

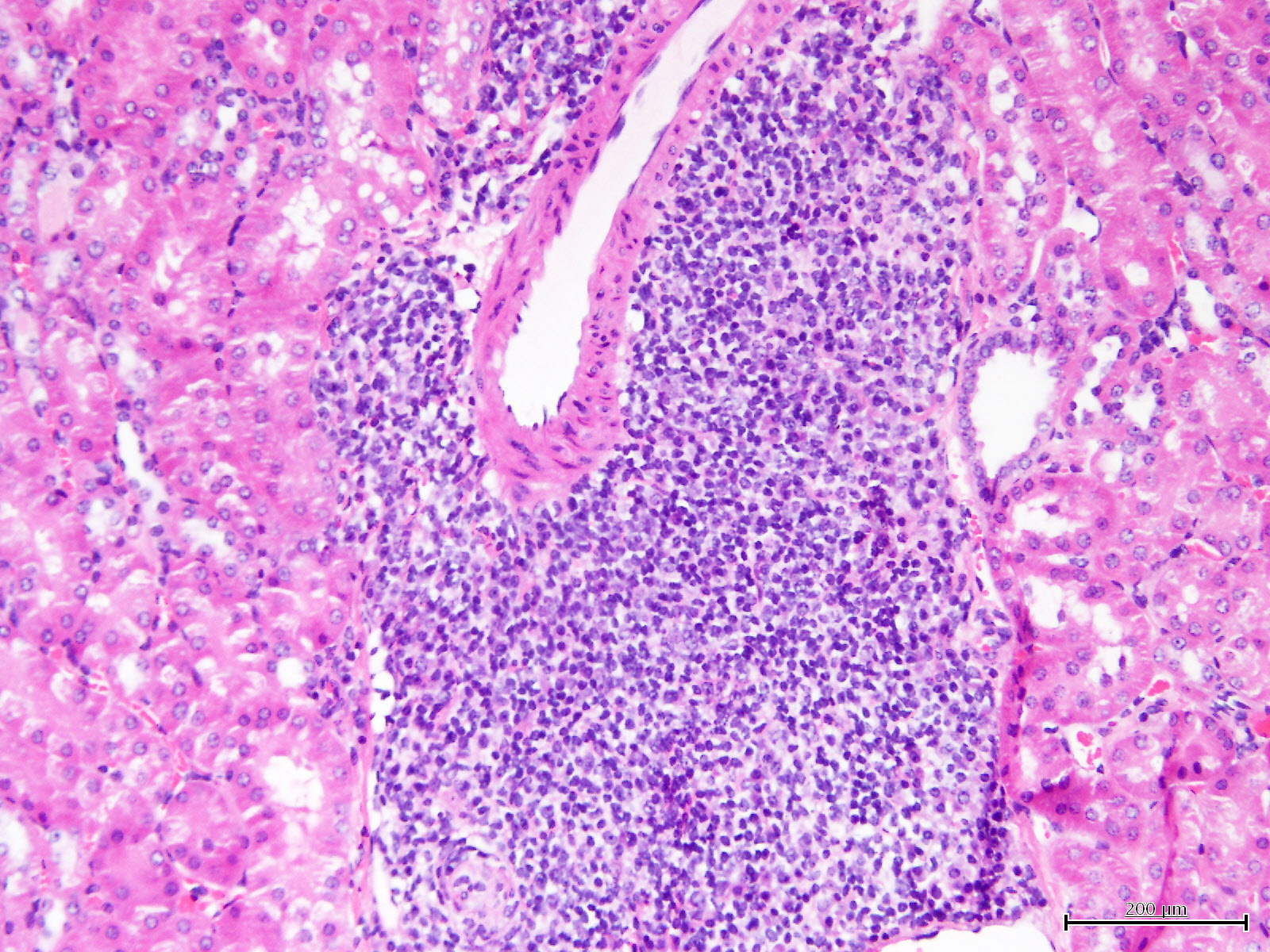

Supplement: S2 File — (ZIP) [file pone.0327042.s002.zip › HE-4w DM/HE 4w DM-1 10X.JPG]

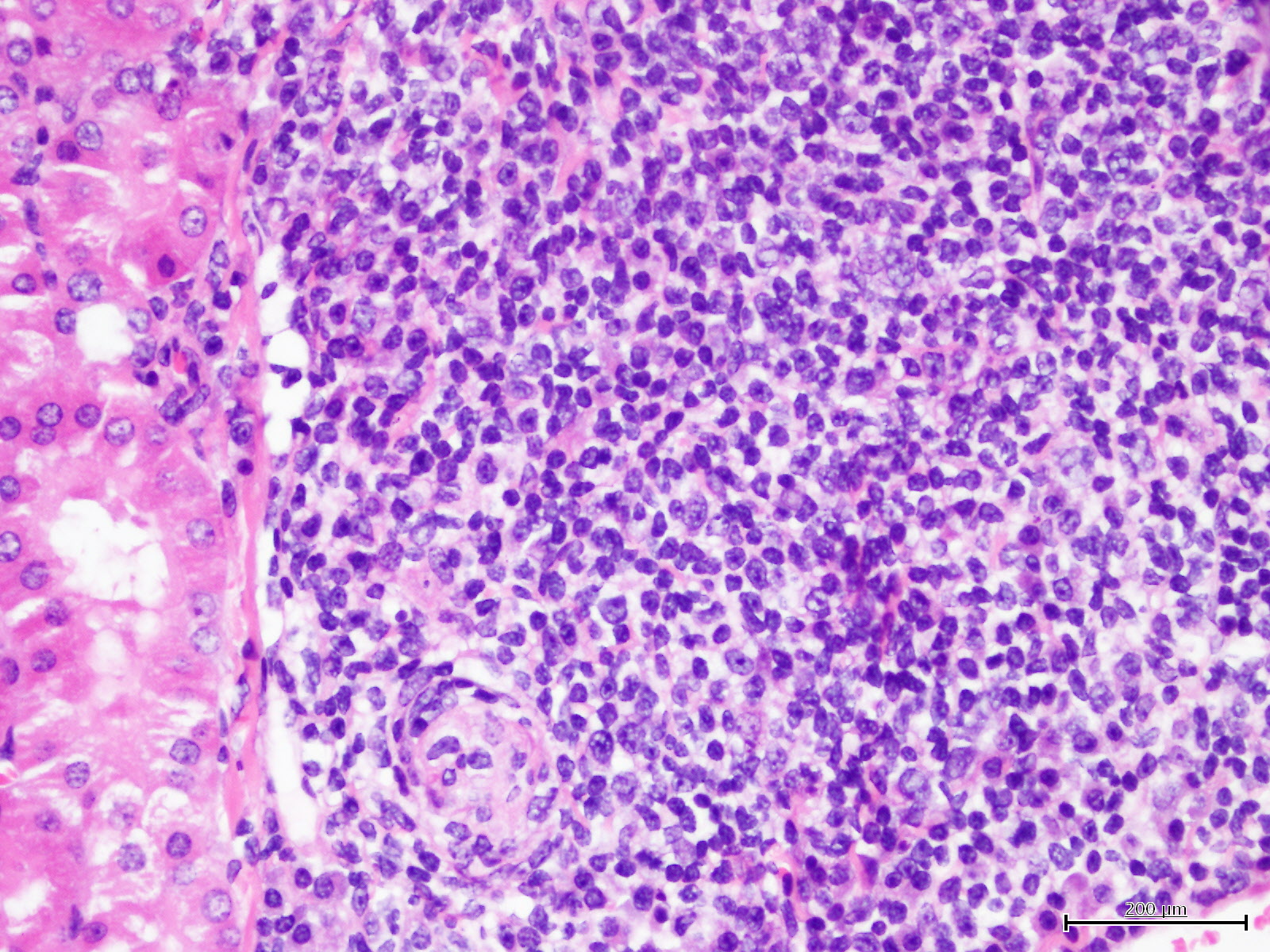

Supplement: S2 File — (ZIP) [file pone.0327042.s002.zip › HE-4w DM/HE 4w DM-1.JPG]

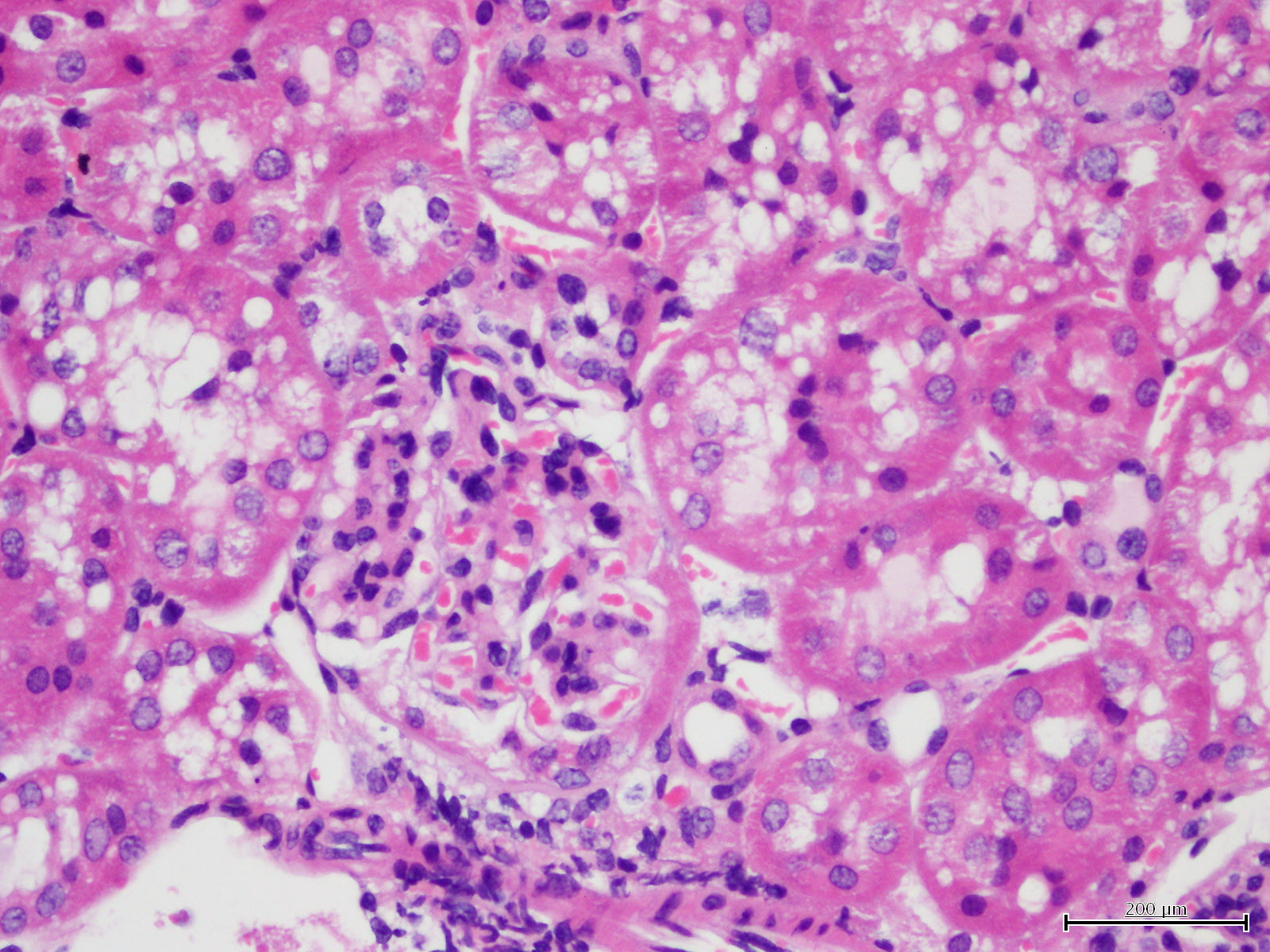

Supplement: S2 File — (ZIP) [file pone.0327042.s002.zip › HE-4w DM/HE 4w DM-2(Used publication).JPG]

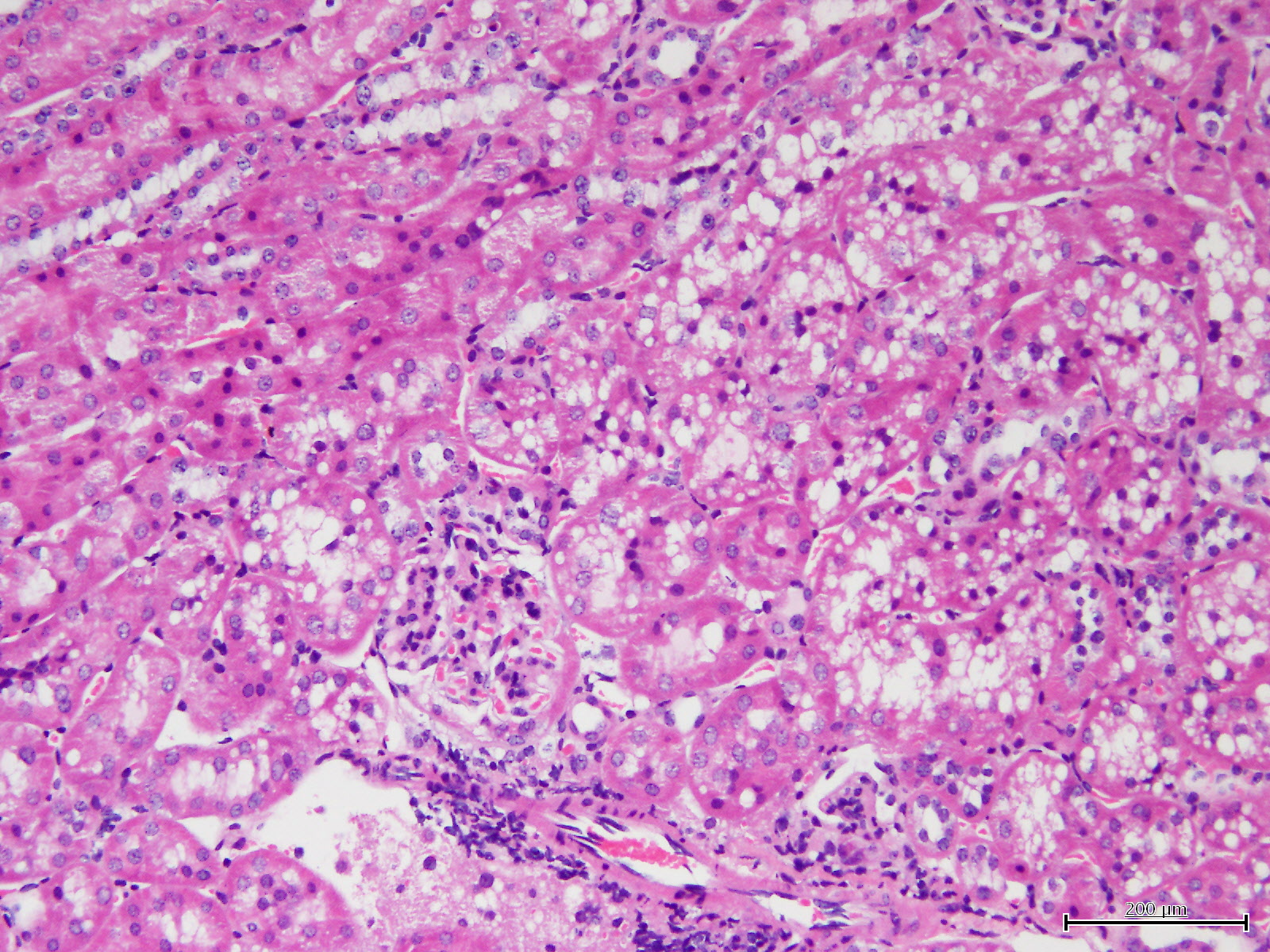

Supplement: S2 File — (ZIP) [file pone.0327042.s002.zip › HE-4w DM/HE 4w DM-2.JPG]

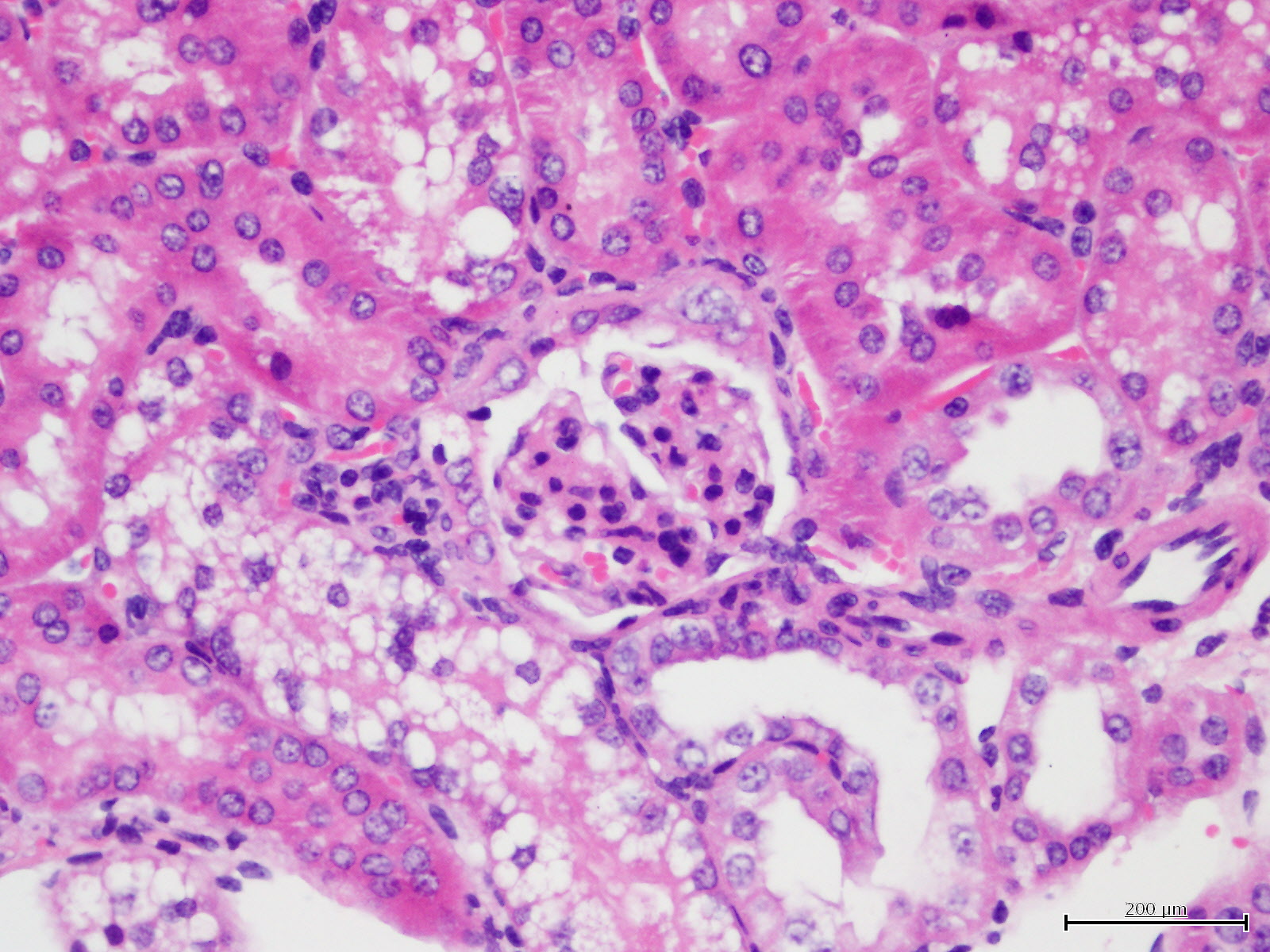

Supplement: S2 File — (ZIP) [file pone.0327042.s002.zip › HE-4w DM/HE 4w DM-3.JPG]

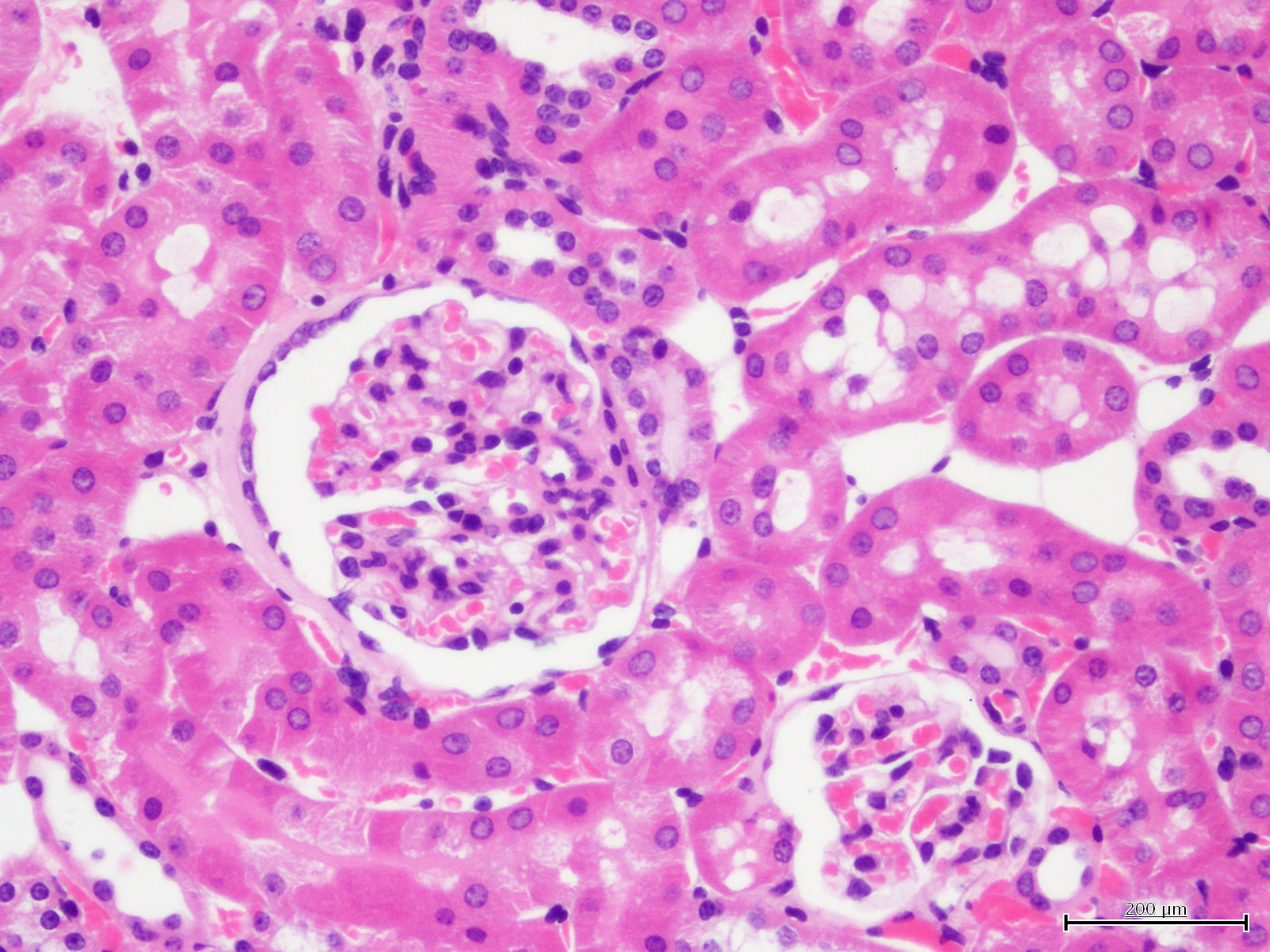

Supplement: S2 File — (ZIP) [file pone.0327042.s002.zip › HE-4w DM/HE 4w DM-4.JPG]

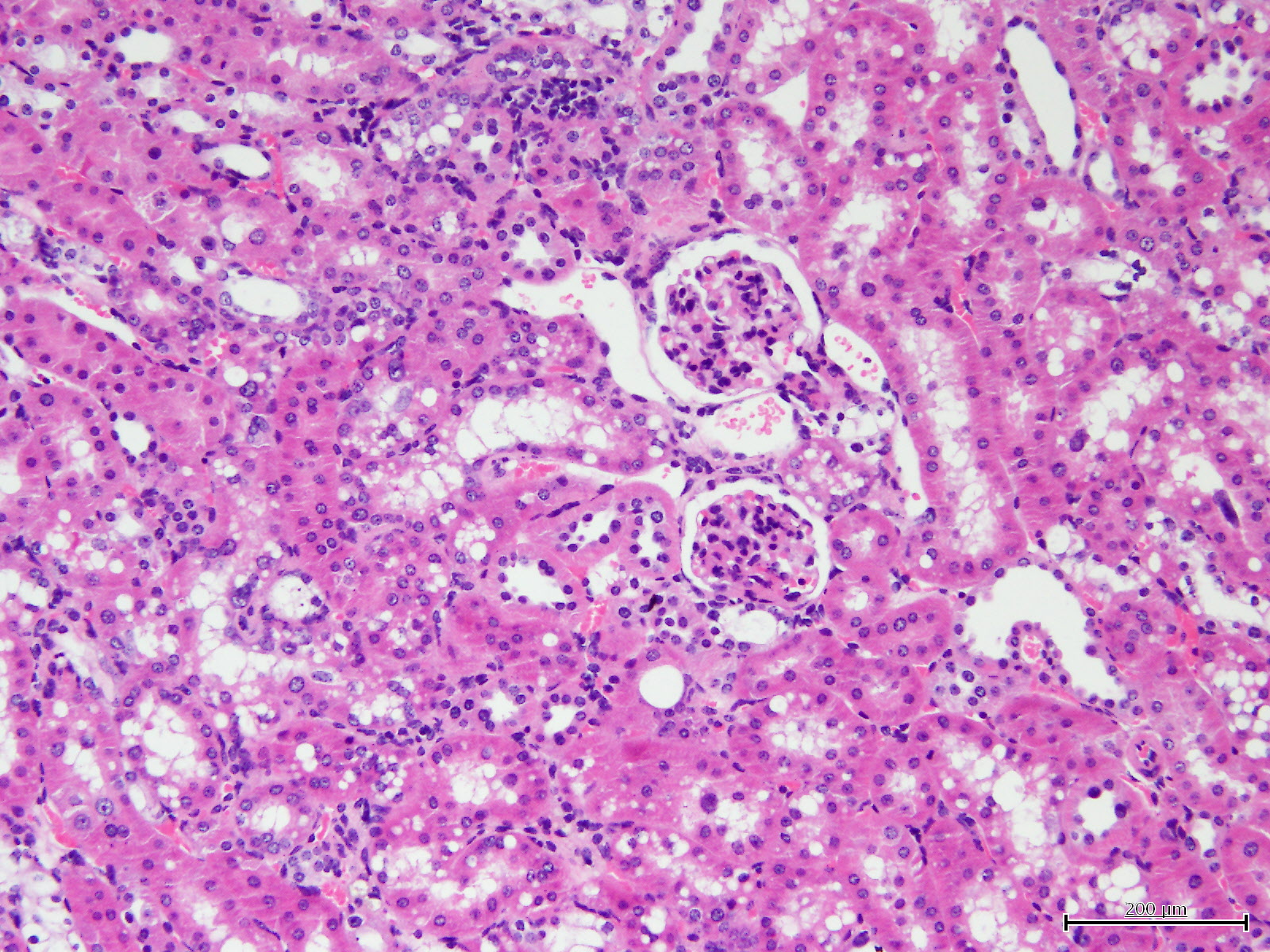

Supplement: S2 File — (ZIP) [file pone.0327042.s002.zip › HE-4w DM 25mGy/4w DM 25mGy-1 20x.JPG]

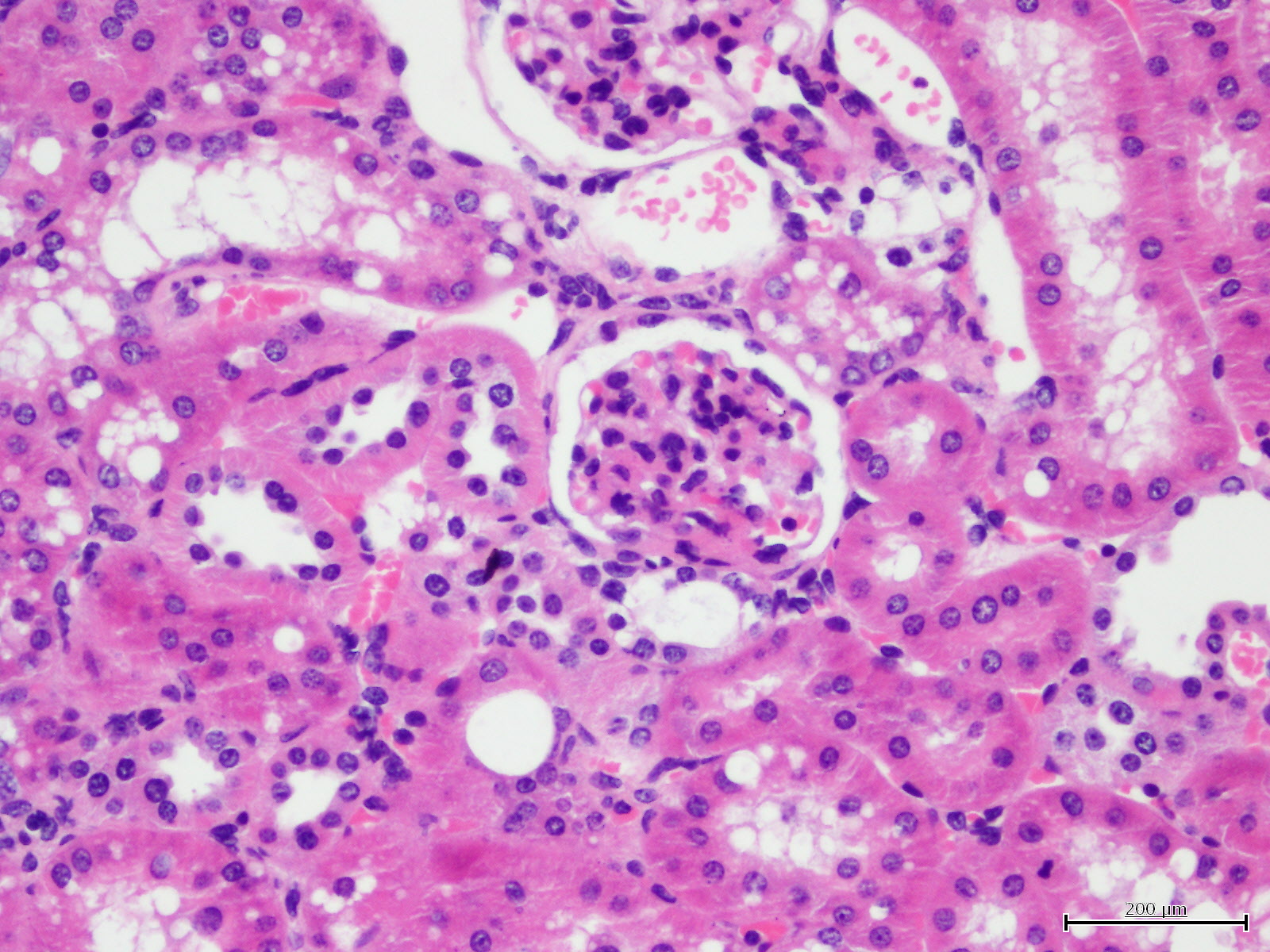

Supplement: S2 File — (ZIP) [file pone.0327042.s002.zip › HE-4w DM 25mGy/4w DM 25mGy-1.JPG]

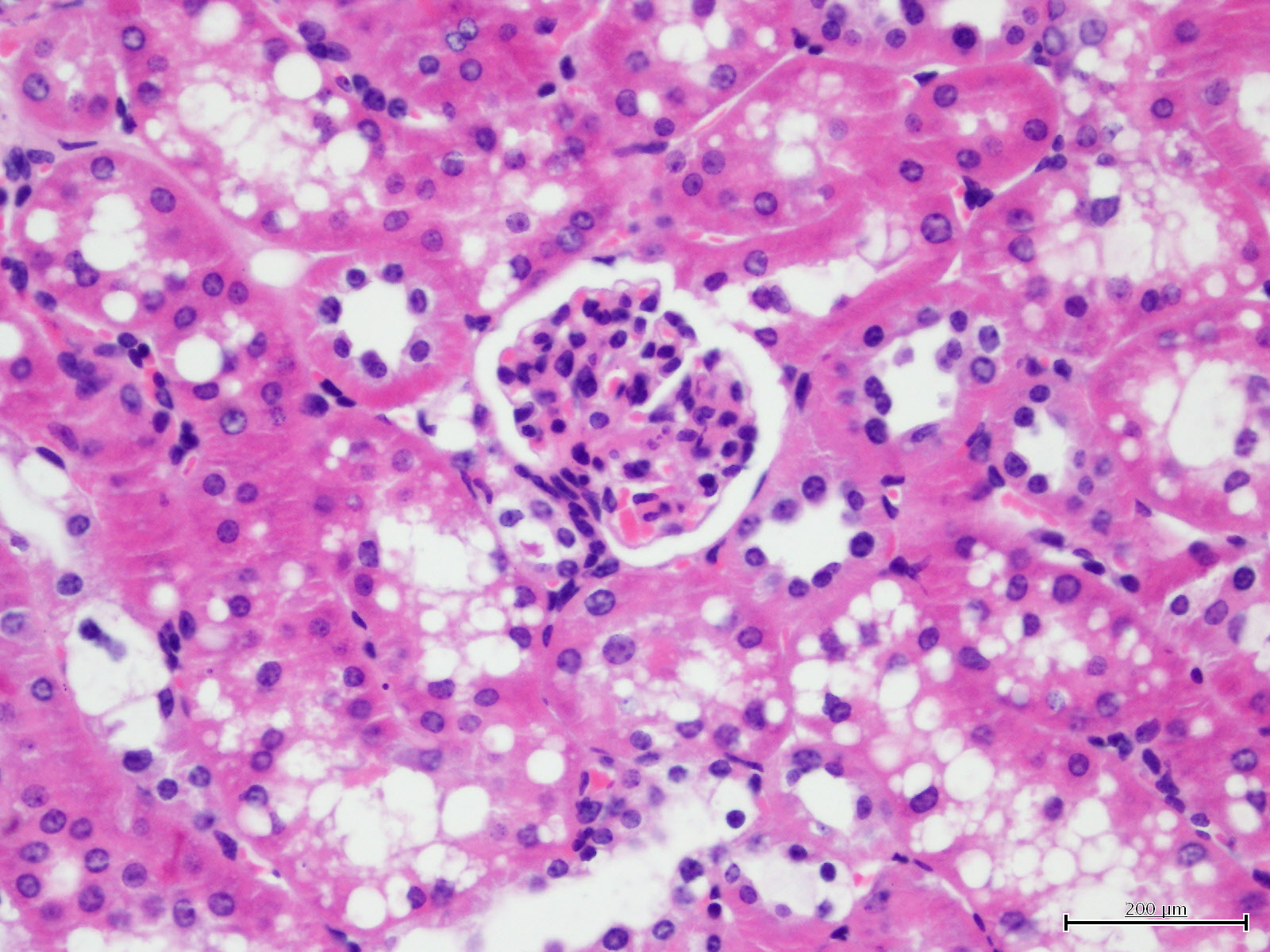

Supplement: S2 File — (ZIP) [file pone.0327042.s002.zip › HE-4w DM 25mGy/4w DM 25mGy-2.JPG]

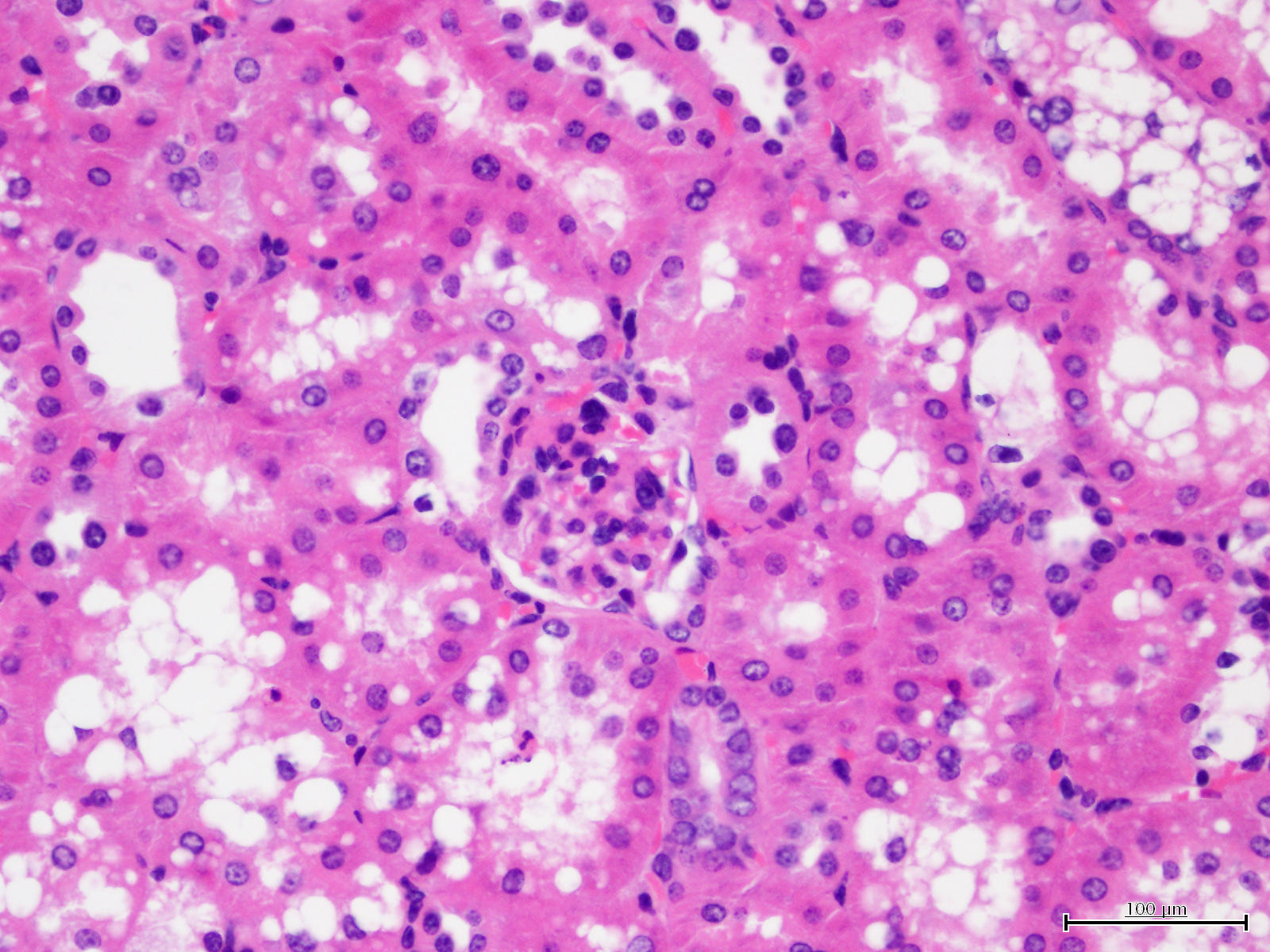

Supplement: S2 File — (ZIP) [file pone.0327042.s002.zip › HE-4w DM 25mGy/4w DM 25mGy-3.TIF]

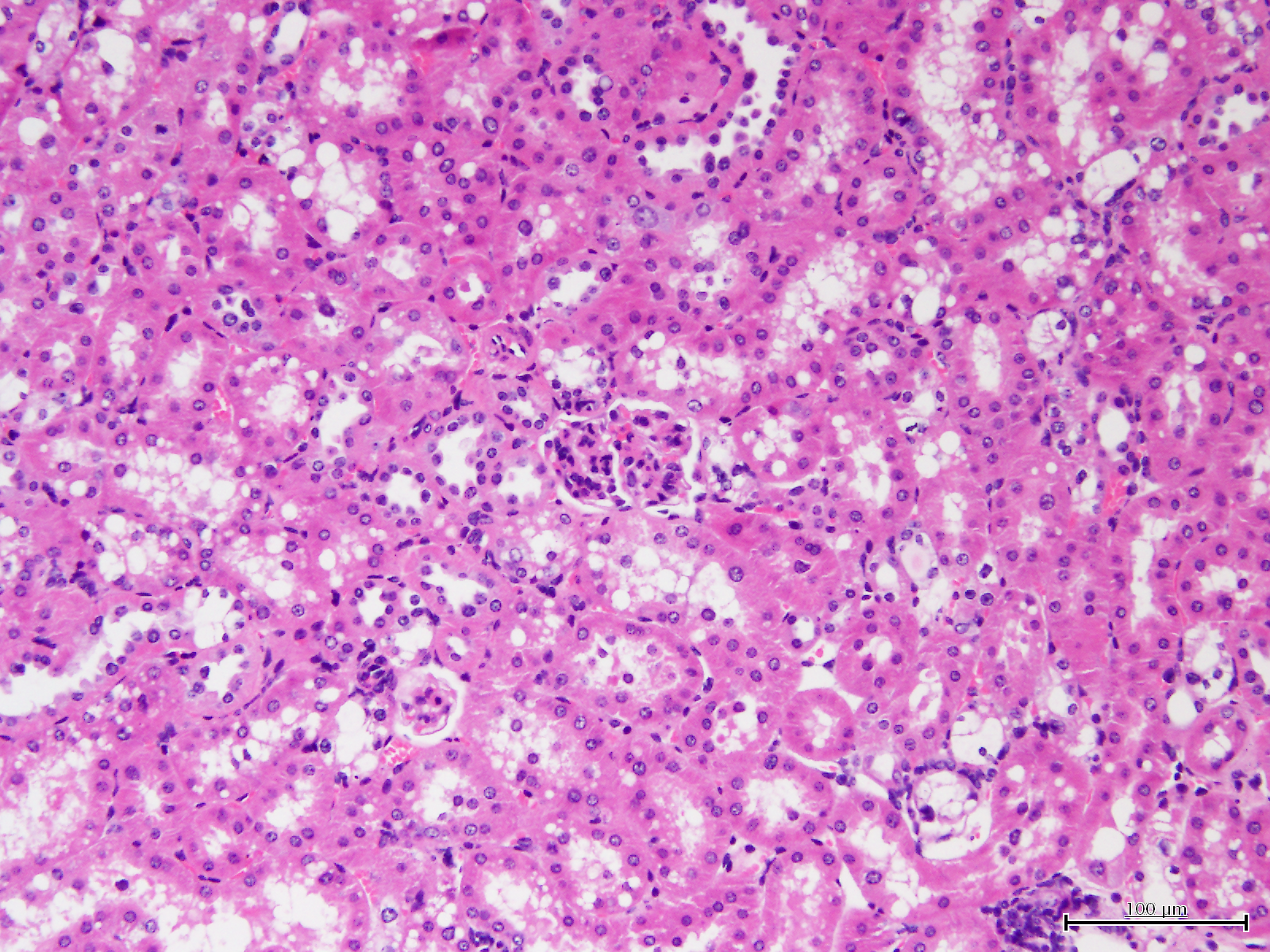

Supplement: S2 File — (ZIP) [file pone.0327042.s002.zip › HE-4w DM 25mGy/4w DM 25mGy-4 20x.TIF]

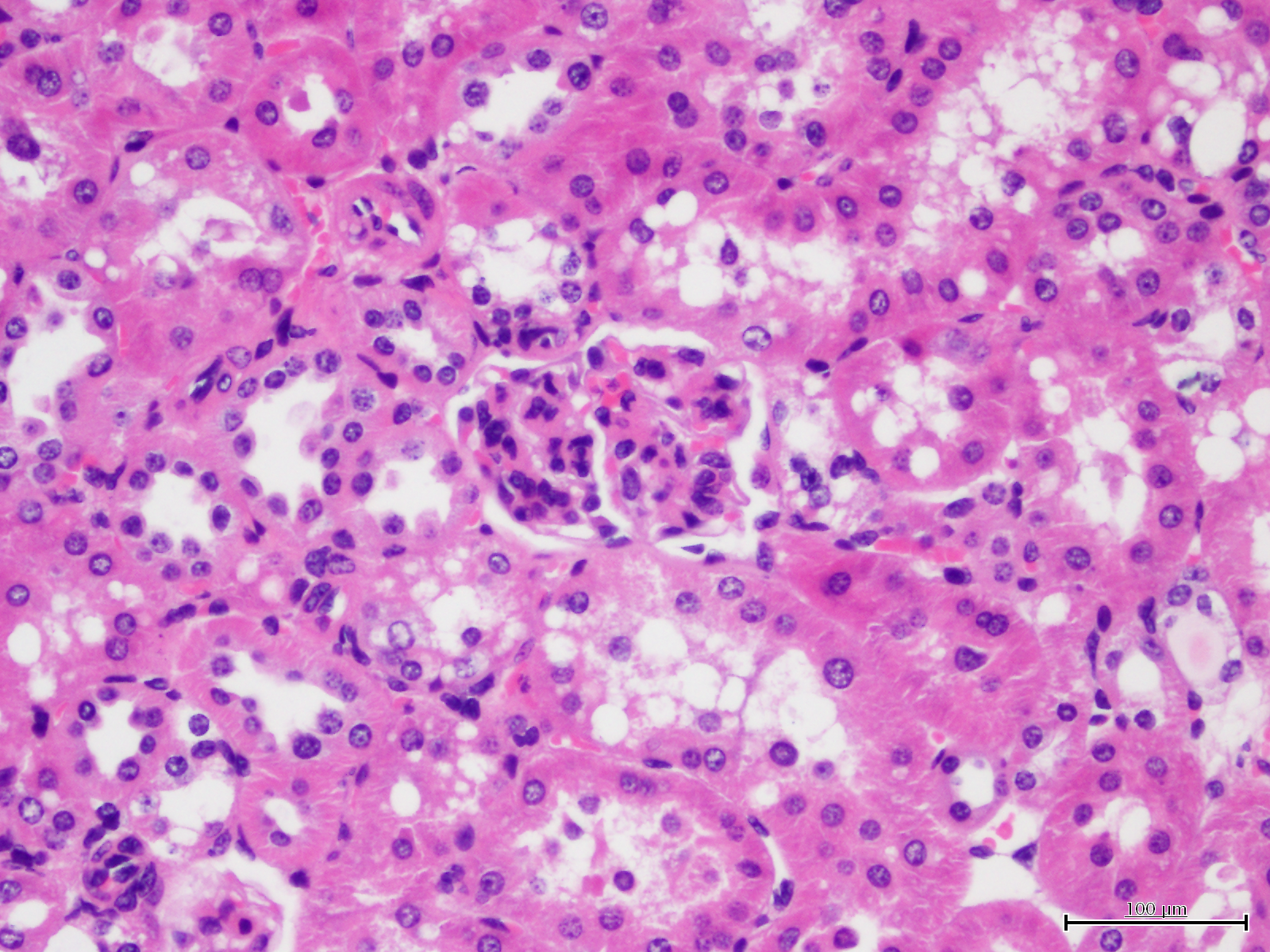

Supplement: S2 File — (ZIP) [file pone.0327042.s002.zip › HE-4w DM 25mGy/4w DM 25mGy-4.TIF]

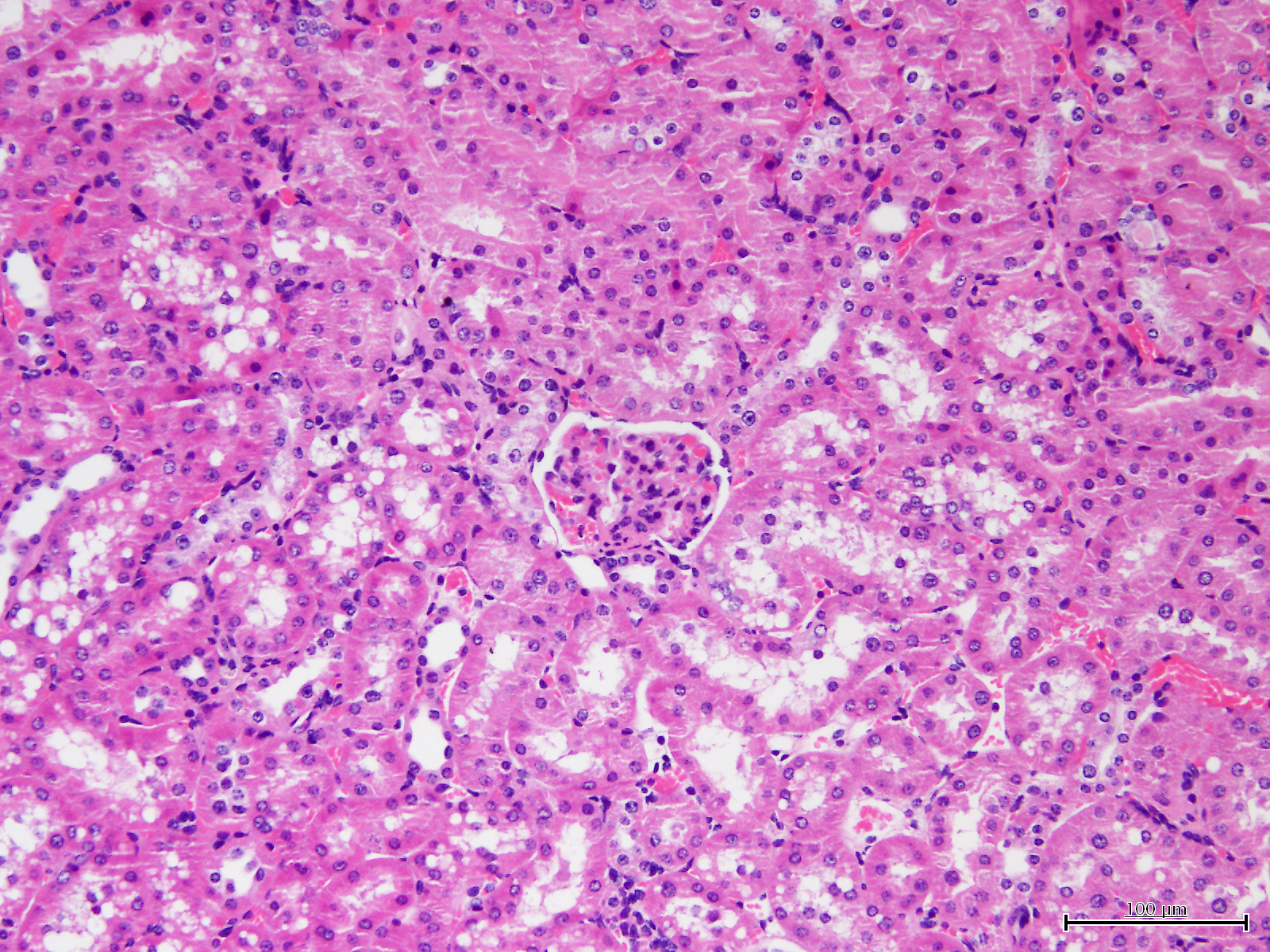

Supplement: S2 File — (ZIP) [file pone.0327042.s002.zip › HE-4w DM 25mGy/4w DM 25mGy-5 20x.TIF]

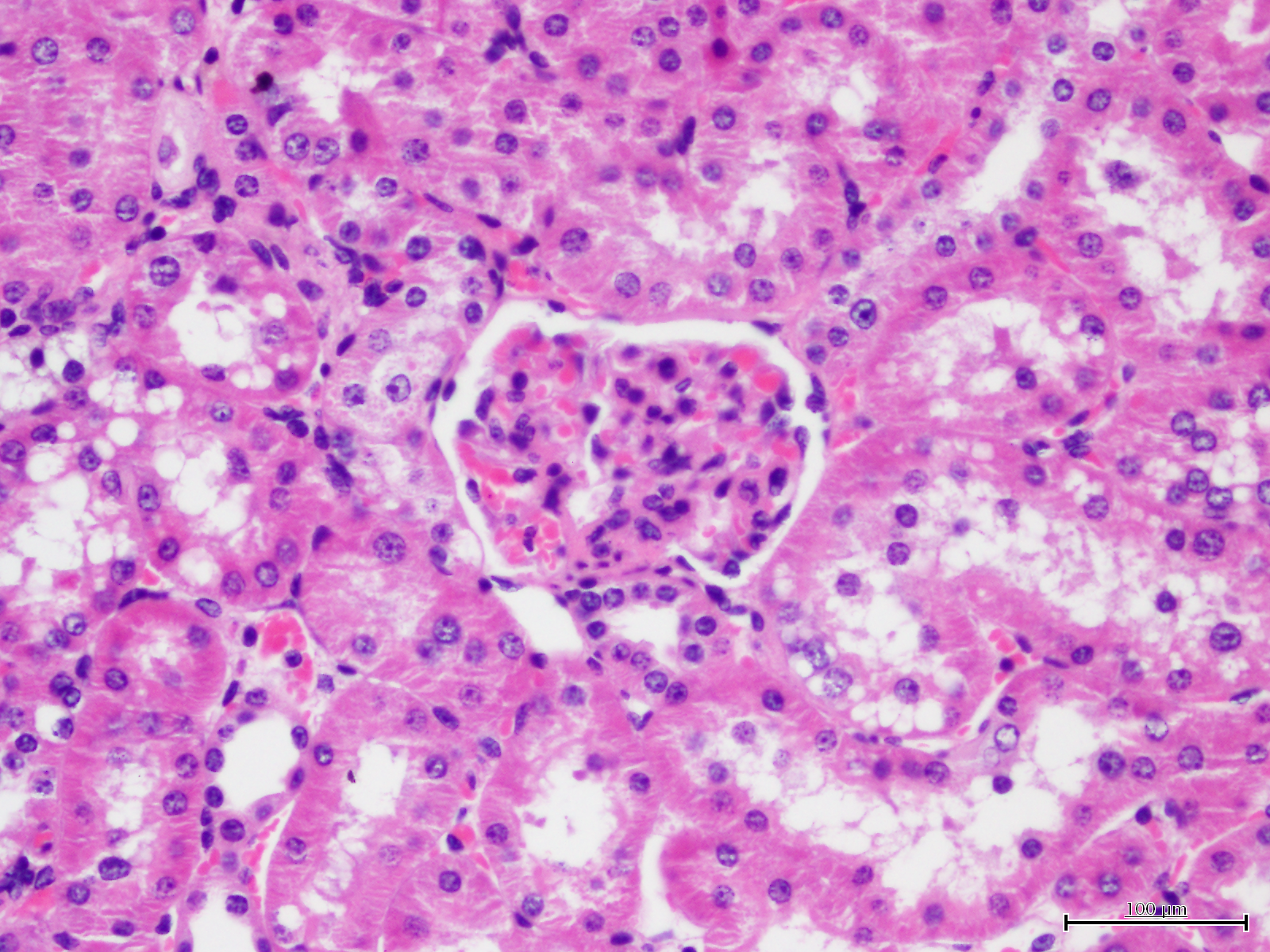

Supplement: S2 File — (ZIP) [file pone.0327042.s002.zip › HE-4w DM 25mGy/4w DM 25mGy-5.TIF]

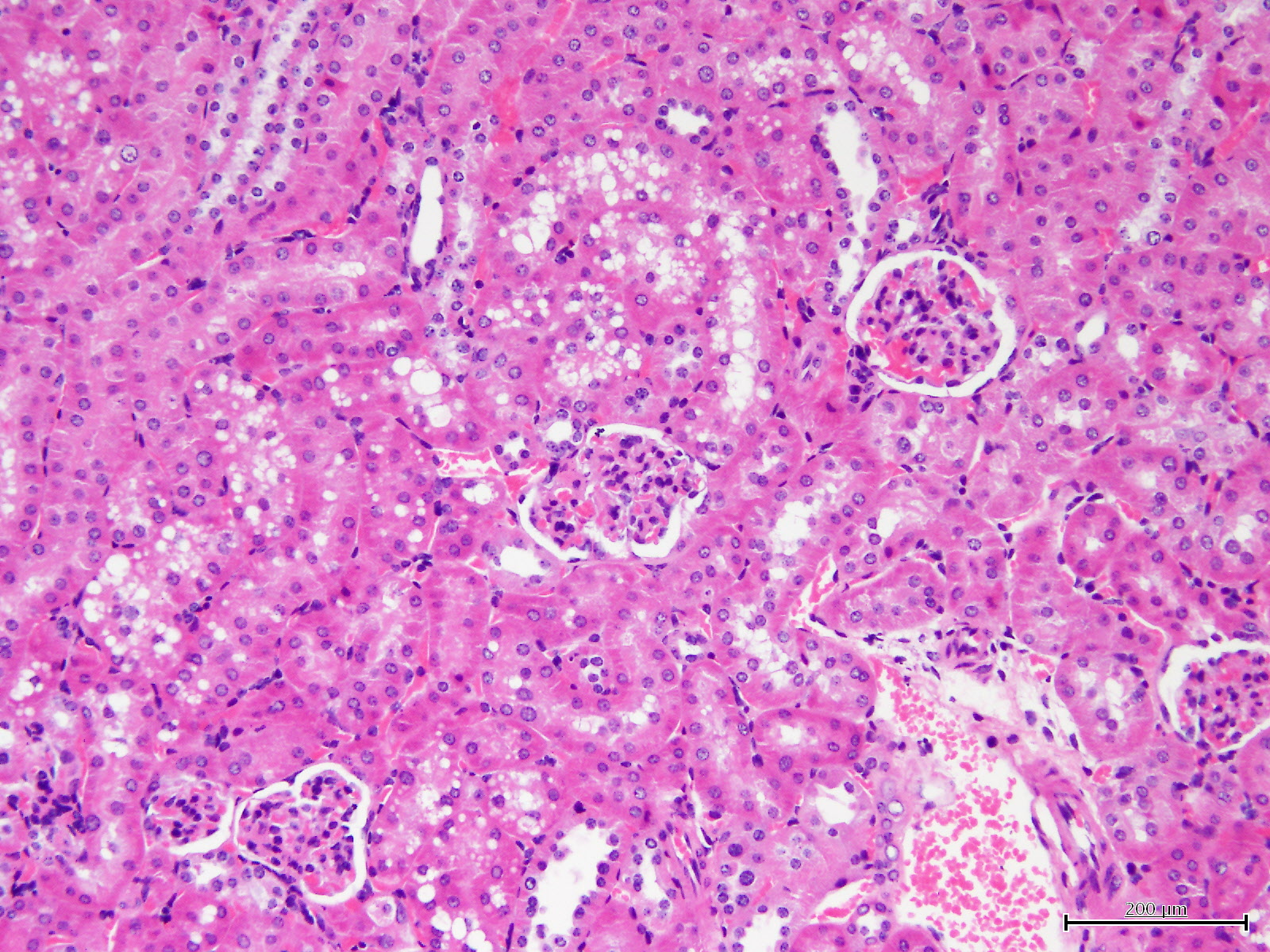

Supplement: S2 File — (ZIP) [file pone.0327042.s002.zip › HE-4w DM 25mGy/4w DM 25mGy-6 20x.JPG]

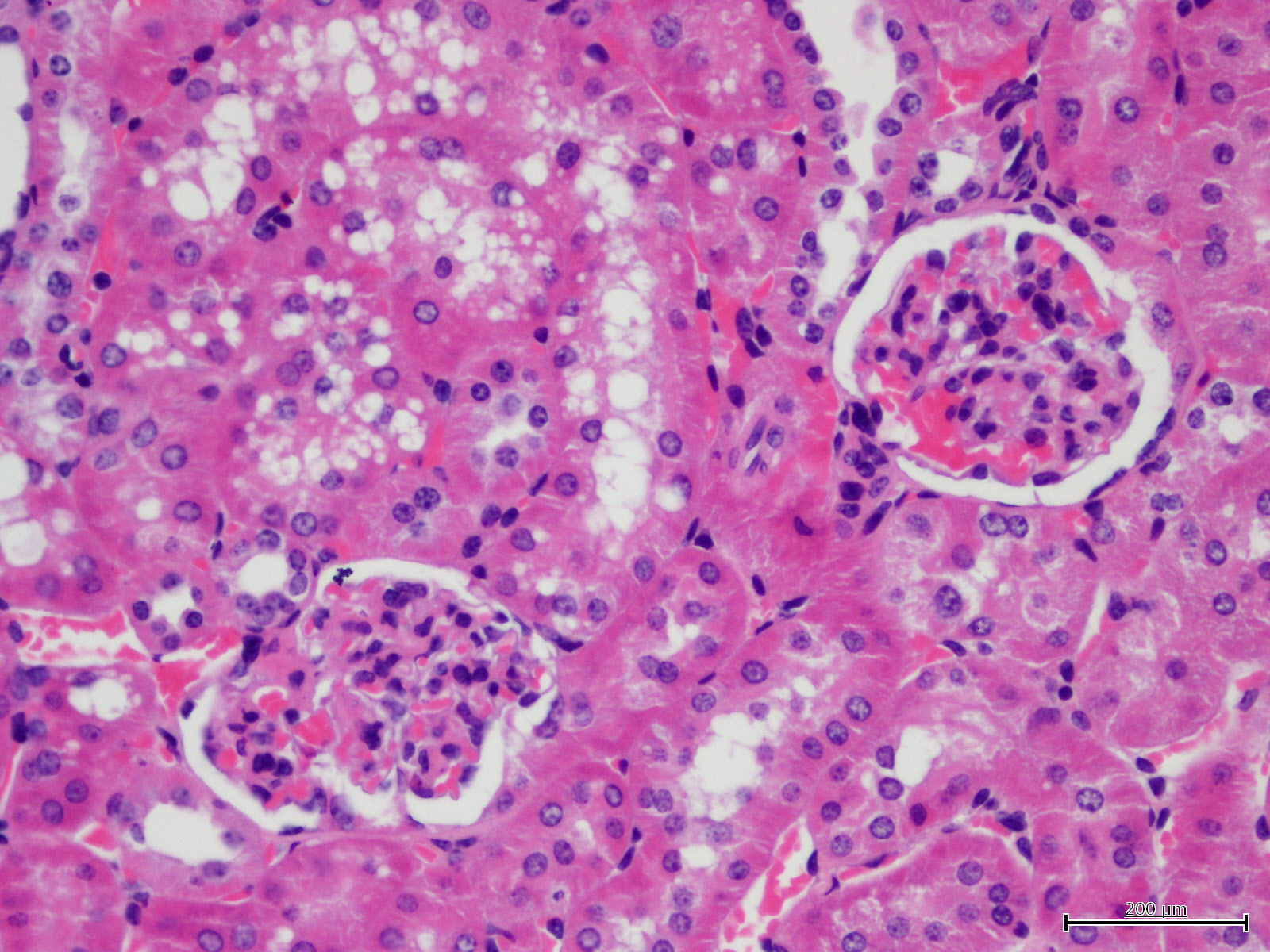

Supplement: S2 File — (ZIP) [file pone.0327042.s002.zip › HE-4w DM 25mGy/4w DM 25mGy-6(Uesd publication).JPG]

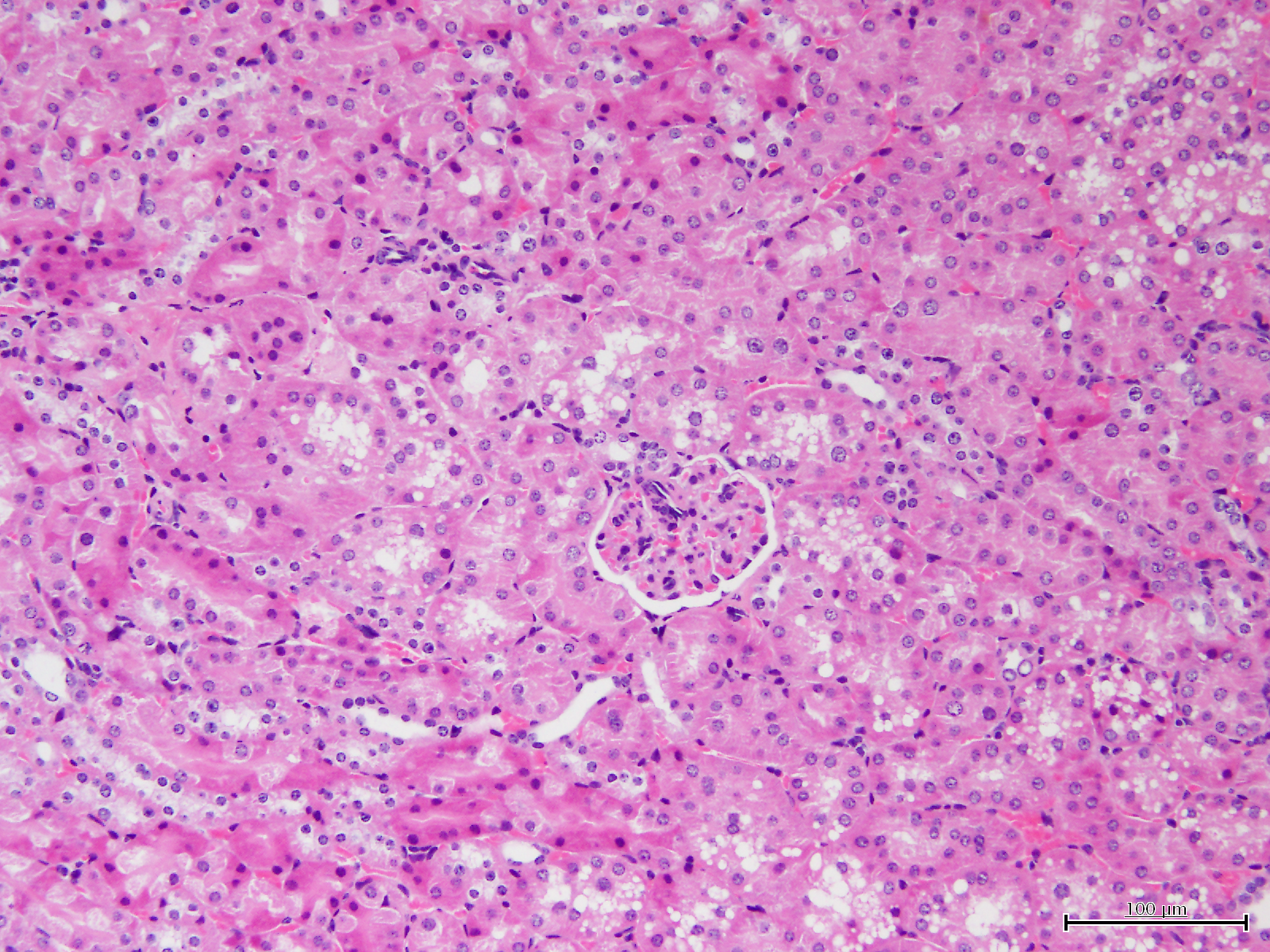

Supplement: S3 File — (ZIP) [file pone.0327042.s003.zip › S3 File - Image data underlying Fig 2A folder 2/HE-4w DM 50mGy/4w DM 50mGy-1 20x.TIF]

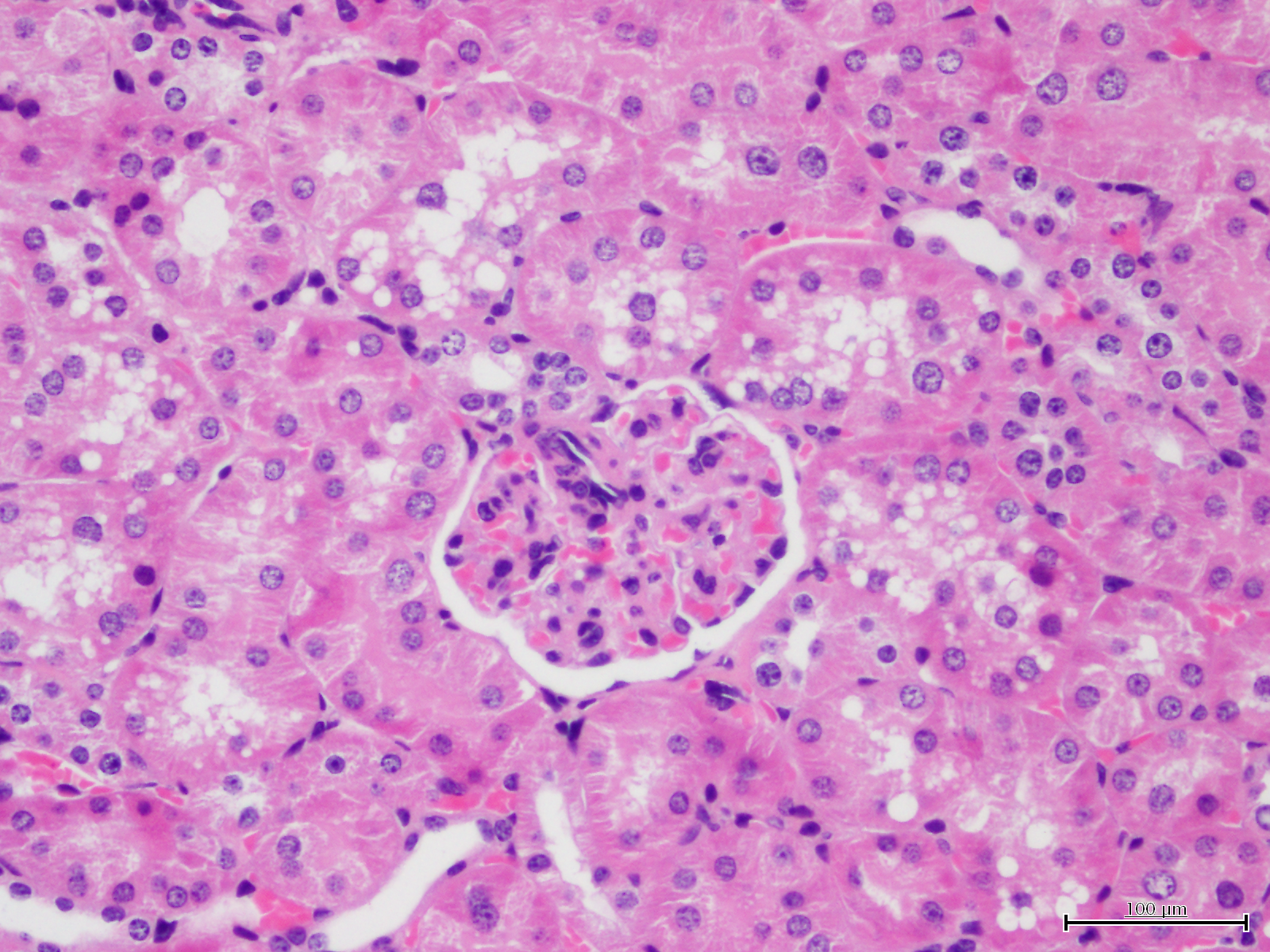

Supplement: S3 File — (ZIP) [file pone.0327042.s003.zip › S3 File - Image data underlying Fig 2A folder 2/HE-4w DM 50mGy/4w DM 50mGy-1.TIF]

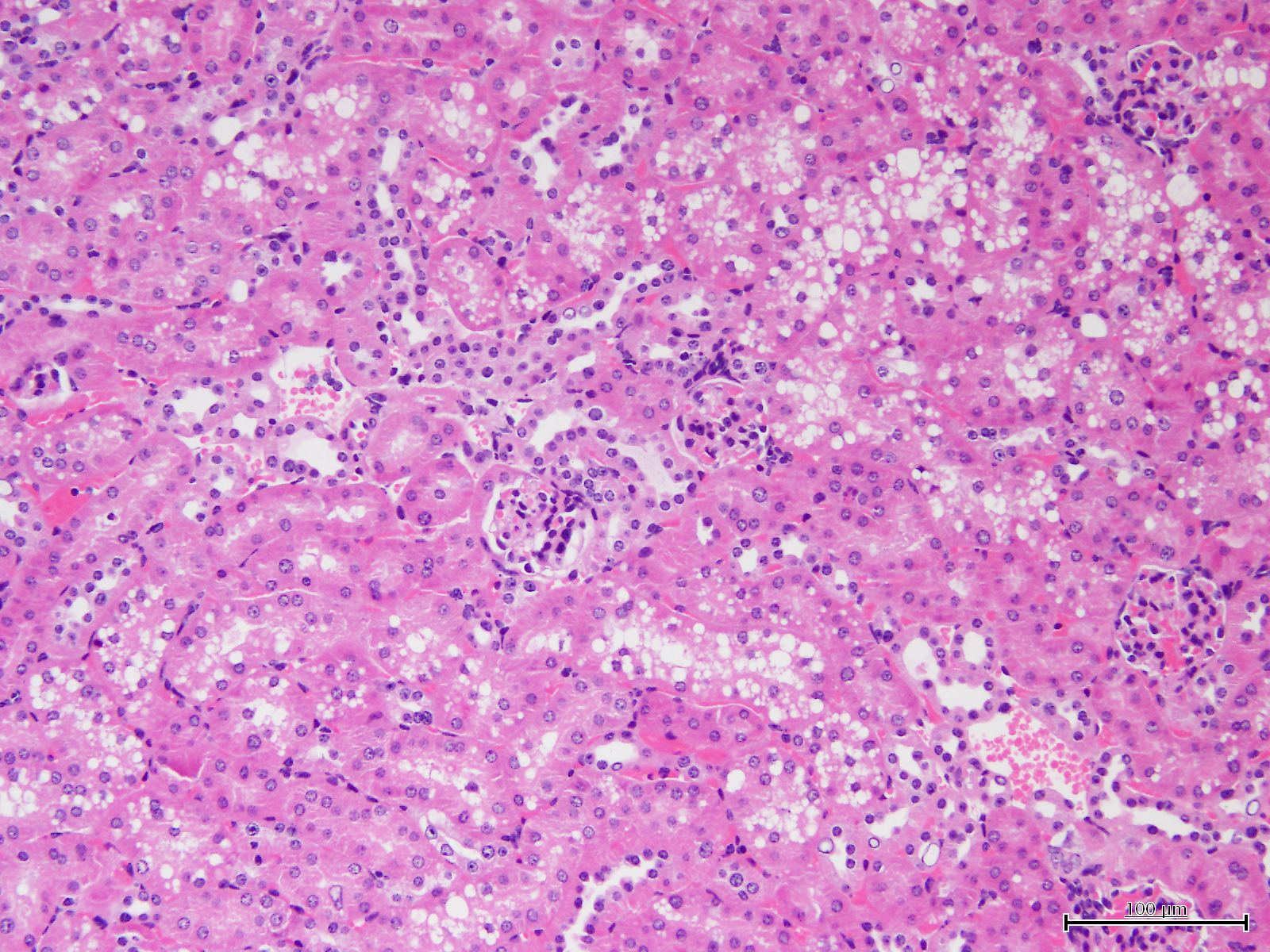

Supplement: S3 File — (ZIP) [file pone.0327042.s003.zip › S3 File - Image data underlying Fig 2A folder 2/HE-4w DM 50mGy/4w DM 50mGy-2 20x.TIF]

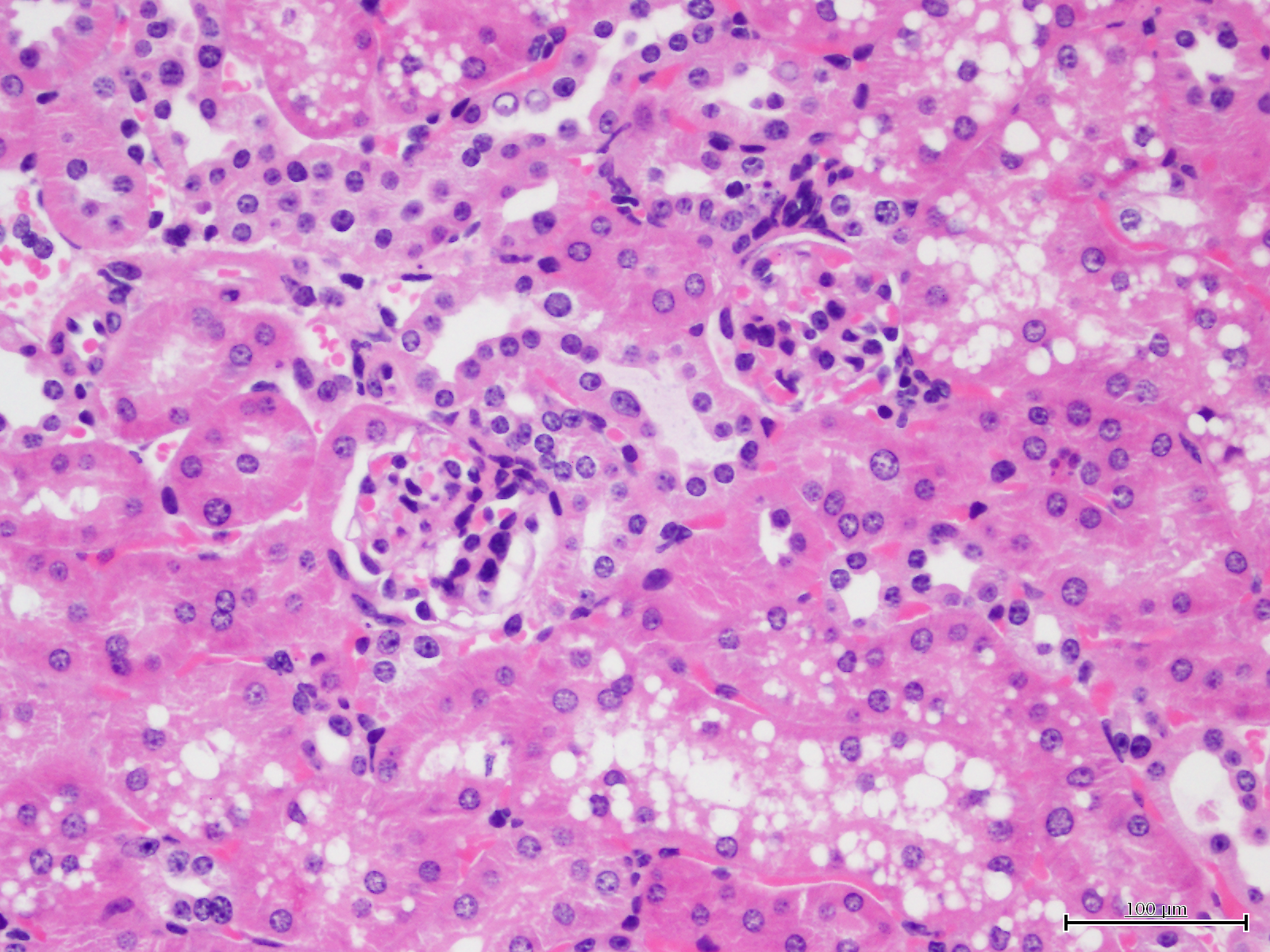

Supplement: S3 File — (ZIP) [file pone.0327042.s003.zip › S3 File - Image data underlying Fig 2A folder 2/HE-4w DM 50mGy/4w DM 50mGy-2.TIF]

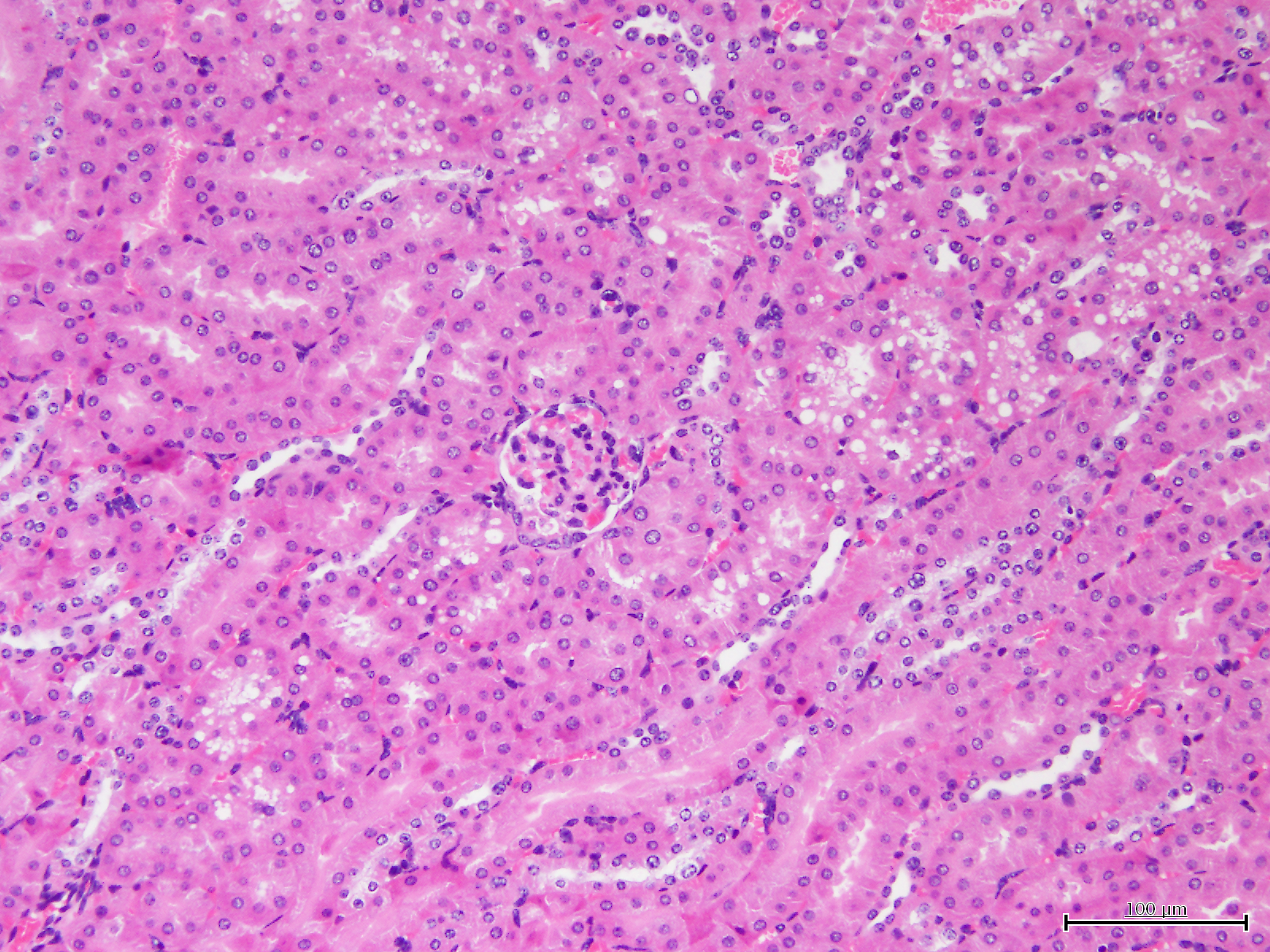

Supplement: S3 File — (ZIP) [file pone.0327042.s003.zip › S3 File - Image data underlying Fig 2A folder 2/HE-4w DM 50mGy/4w DM 50mGy-3 20x.TIF]

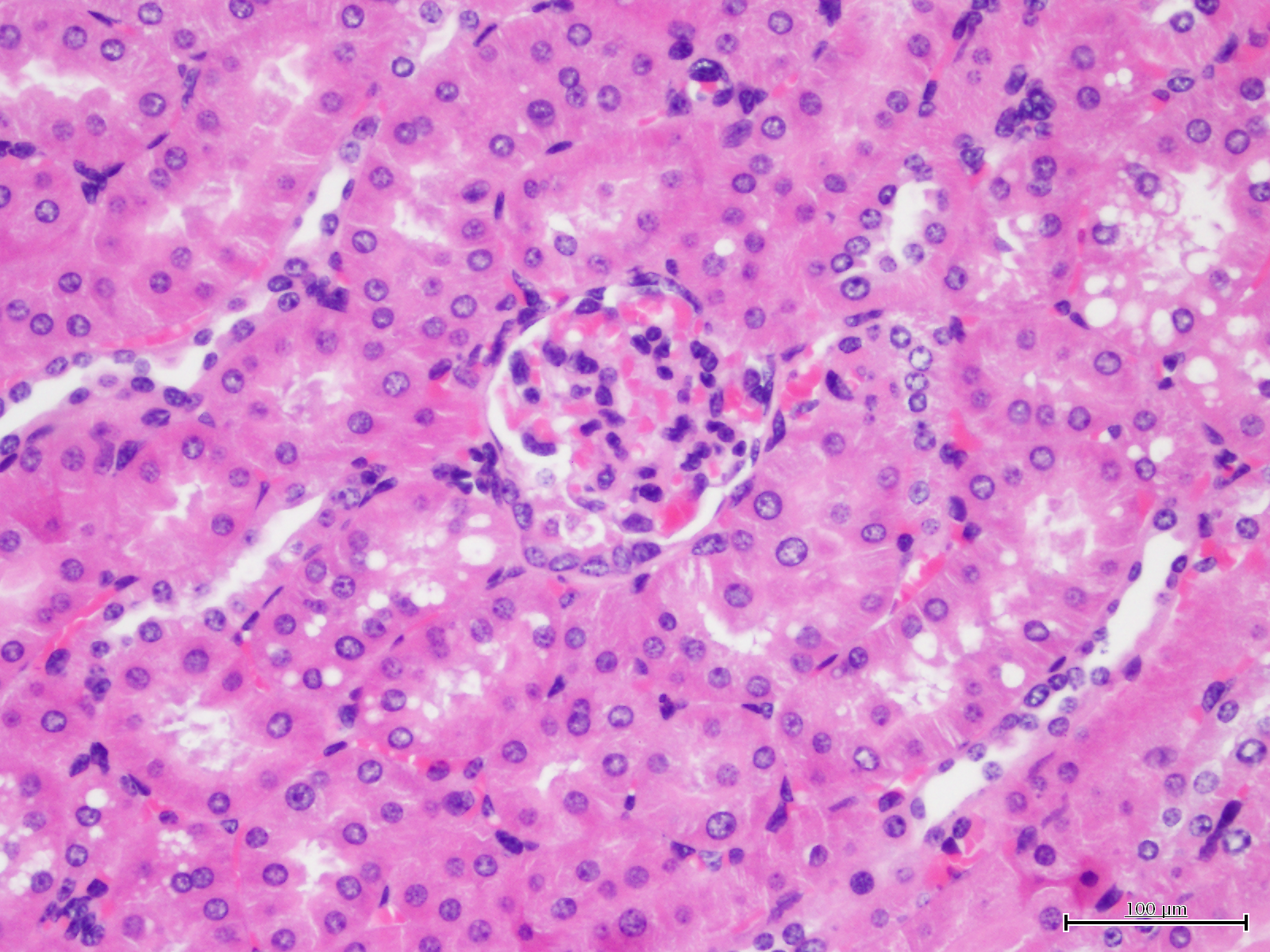

Supplement: S3 File — (ZIP) [file pone.0327042.s003.zip › S3 File - Image data underlying Fig 2A folder 2/HE-4w DM 50mGy/4w DM 50mGy-3.TIF]

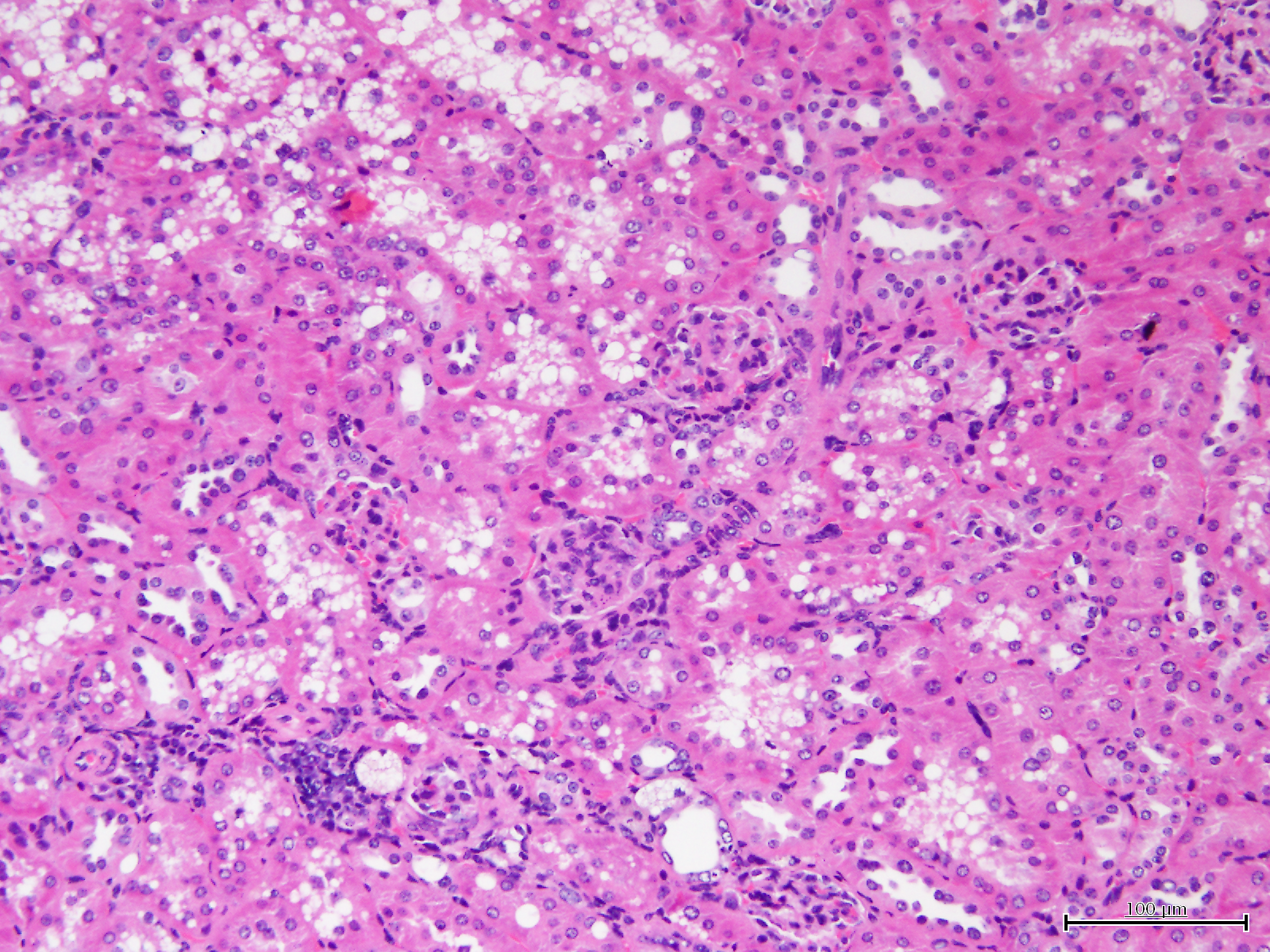

Supplement: S3 File — (ZIP) [file pone.0327042.s003.zip › S3 File - Image data underlying Fig 2A folder 2/HE-4w DM 50mGy/4w DM 50mGy-4 20x.TIF]

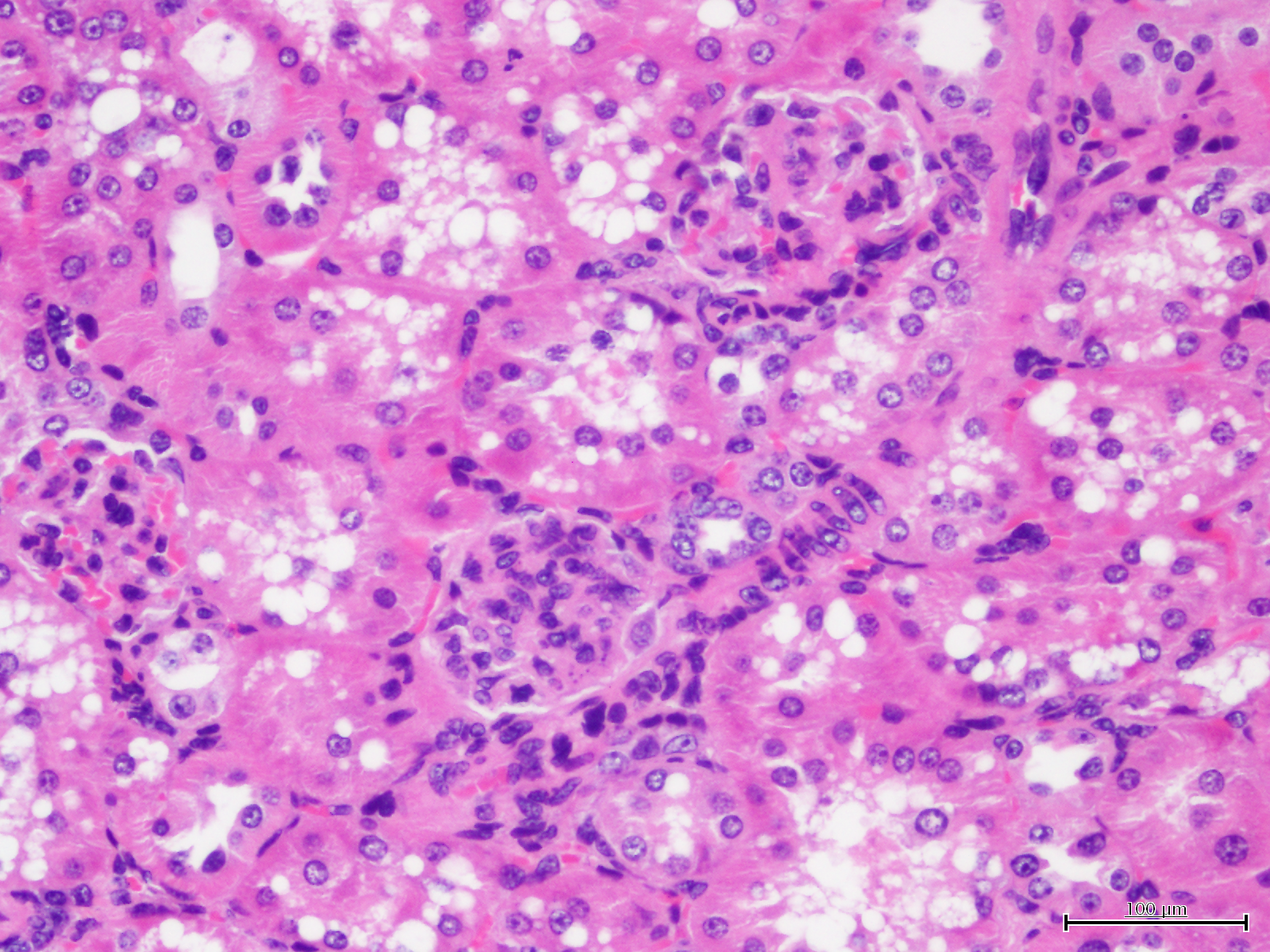

Supplement: S3 File — (ZIP) [file pone.0327042.s003.zip › S3 File - Image data underlying Fig 2A folder 2/HE-4w DM 50mGy/4w DM 50mGy-4.TIF]

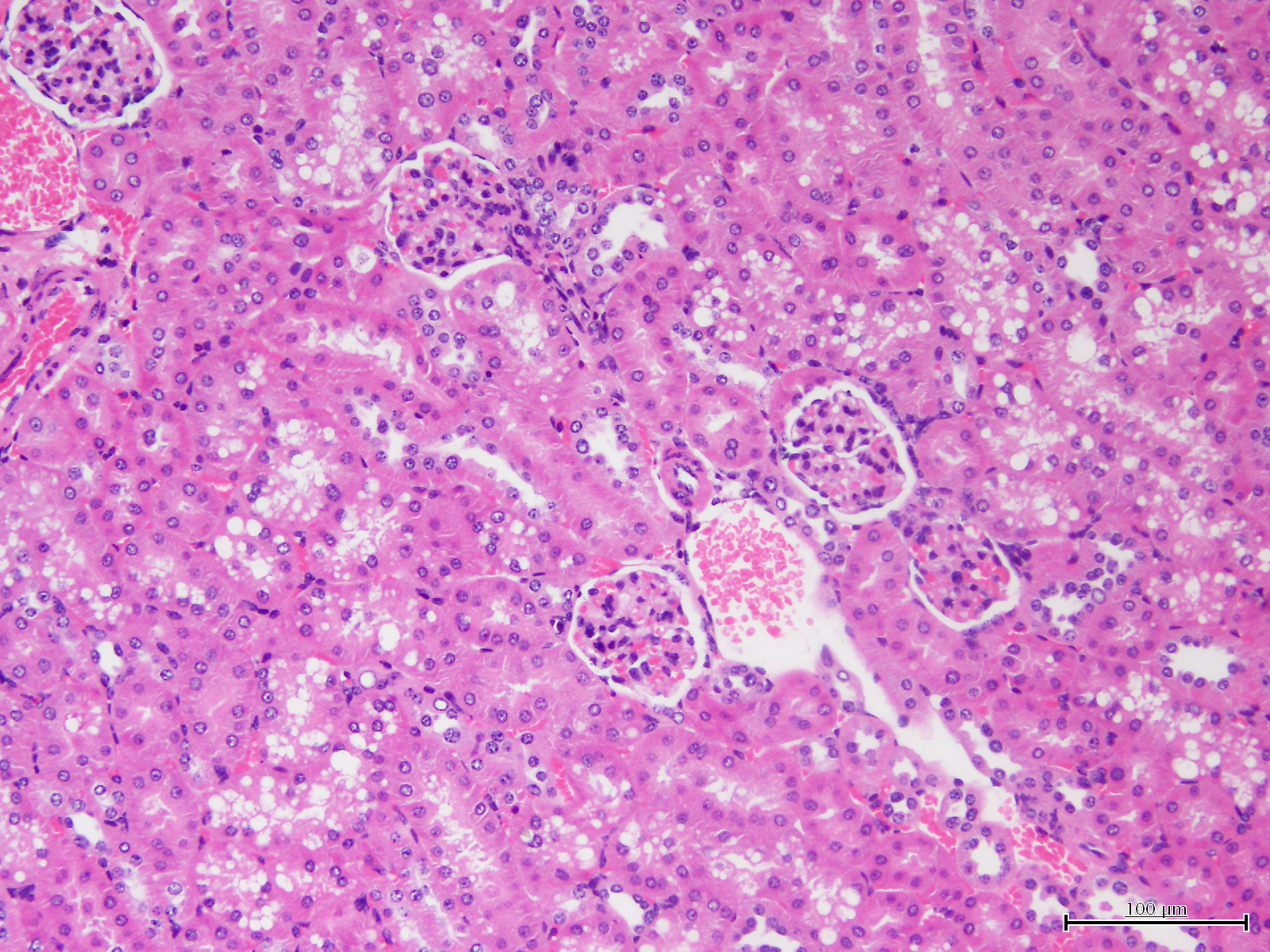

Supplement: S3 File — (ZIP) [file pone.0327042.s003.zip › S3 File - Image data underlying Fig 2A folder 2/HE-4w DM 50mGy/4w DM 50mGy-5.TIF]

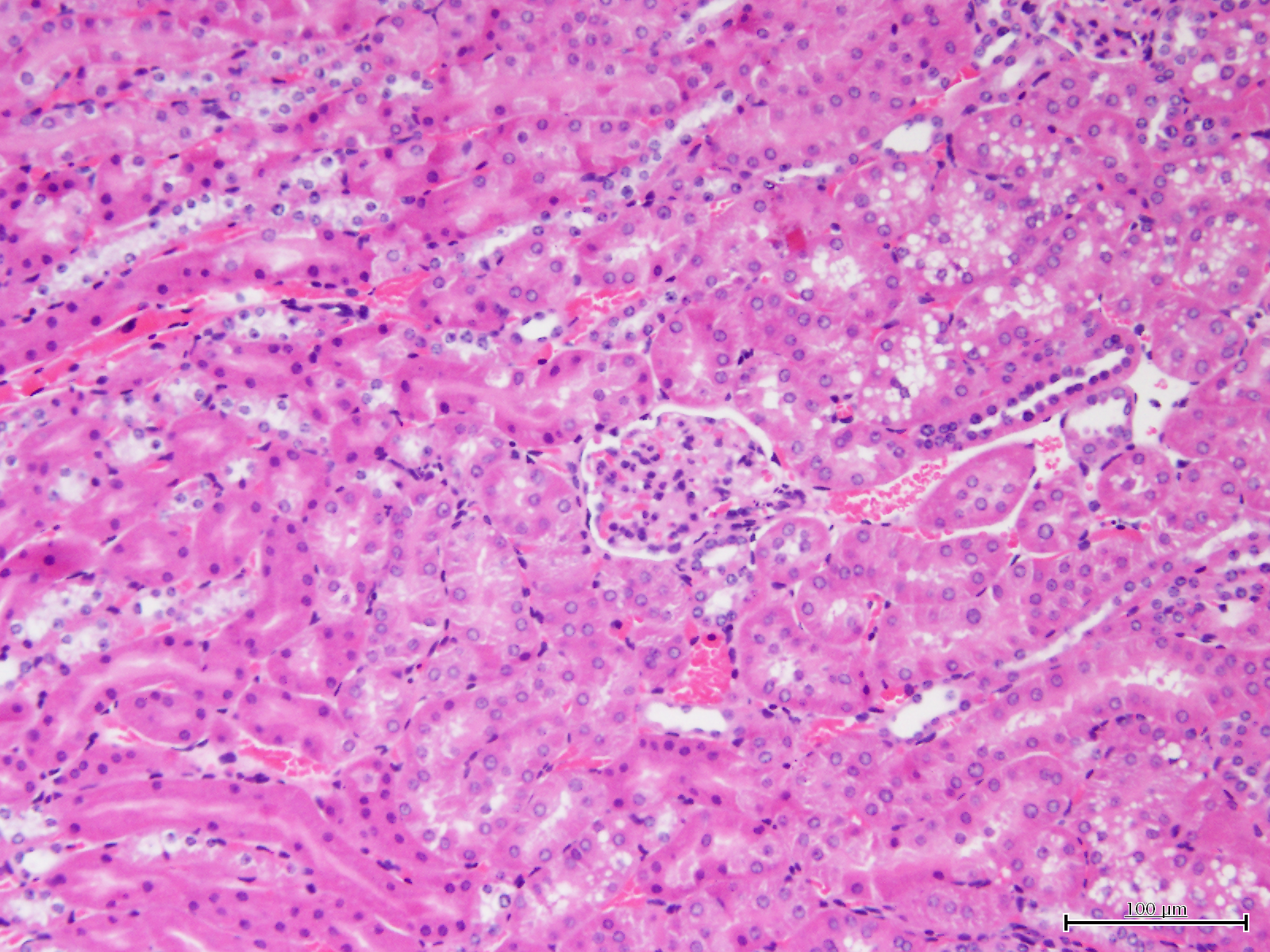

Supplement: S3 File — (ZIP) [file pone.0327042.s003.zip › S3 File - Image data underlying Fig 2A folder 2/HE-4w DM 50mGy/4w DM 50mGy-6 20X.TIF]

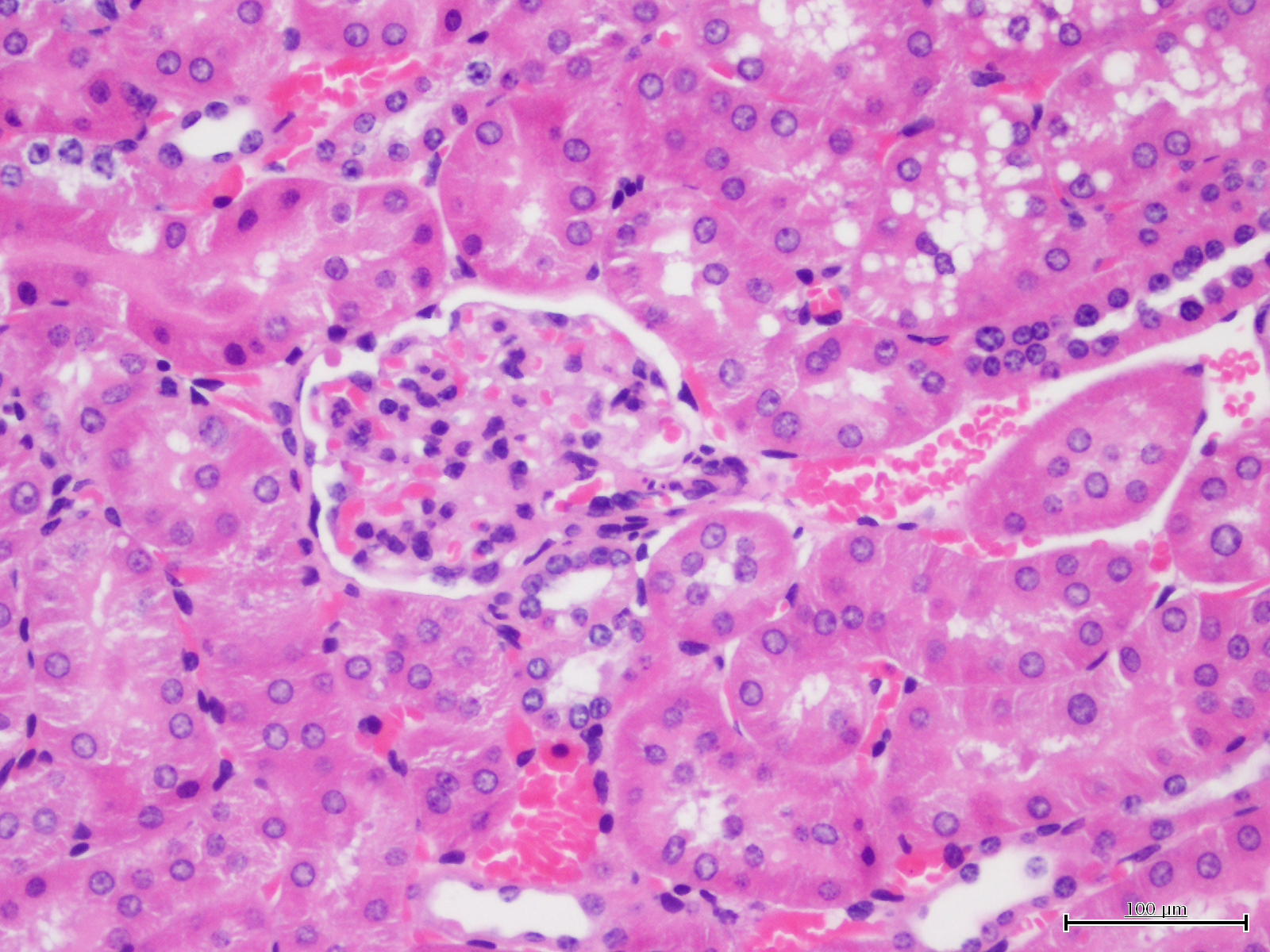

Supplement: S3 File — (ZIP) [file pone.0327042.s003.zip › S3 File - Image data underlying Fig 2A folder 2/HE-4w DM 50mGy/4w DM 50mGy-6(Used publication).TIF]

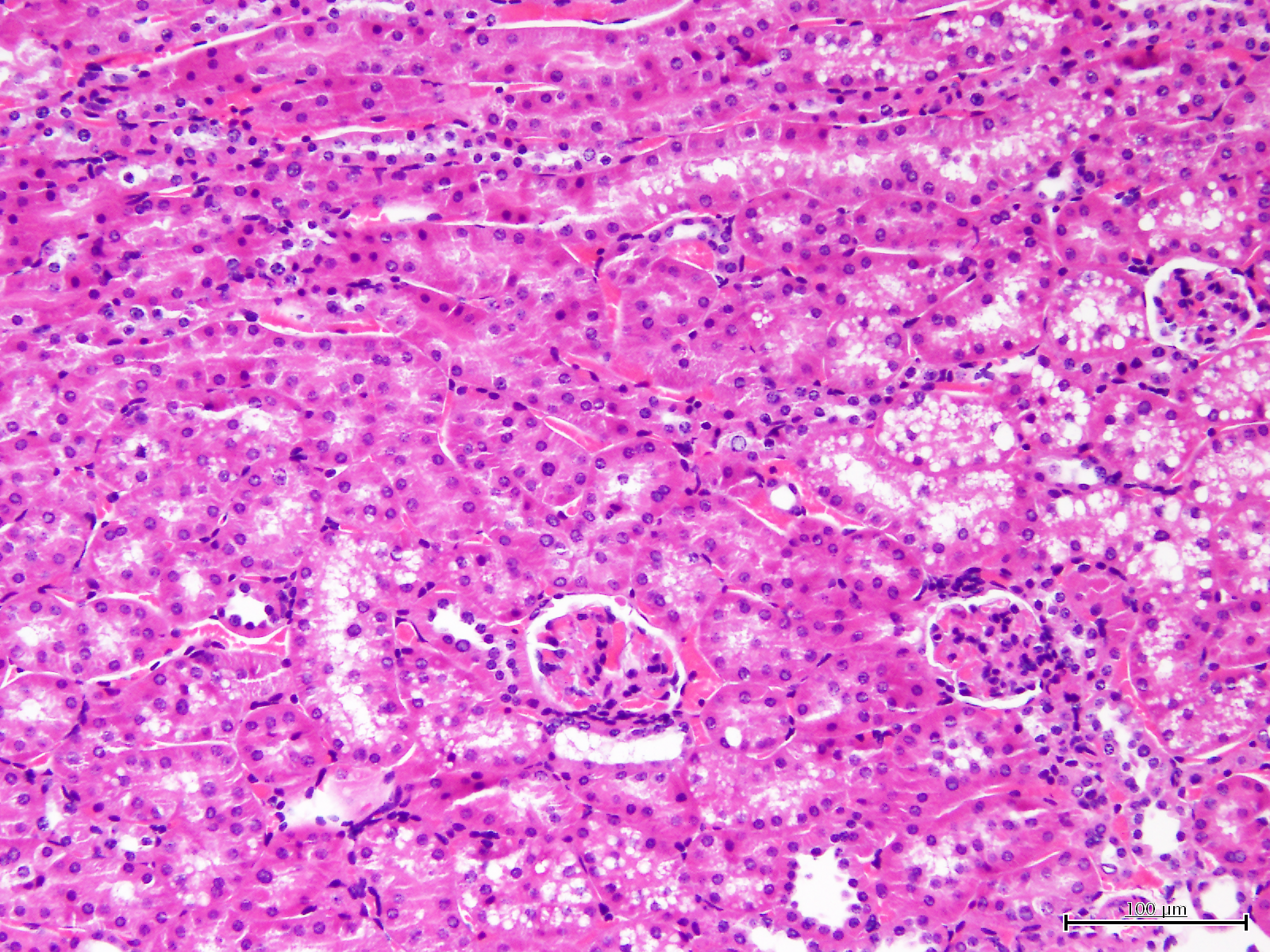

Supplement: S3 File — (ZIP) [file pone.0327042.s003.zip › S3 File - Image data underlying Fig 2A folder 2/HE-4w DM 75mGy/4w DM 75mGy-1 20x.TIF]

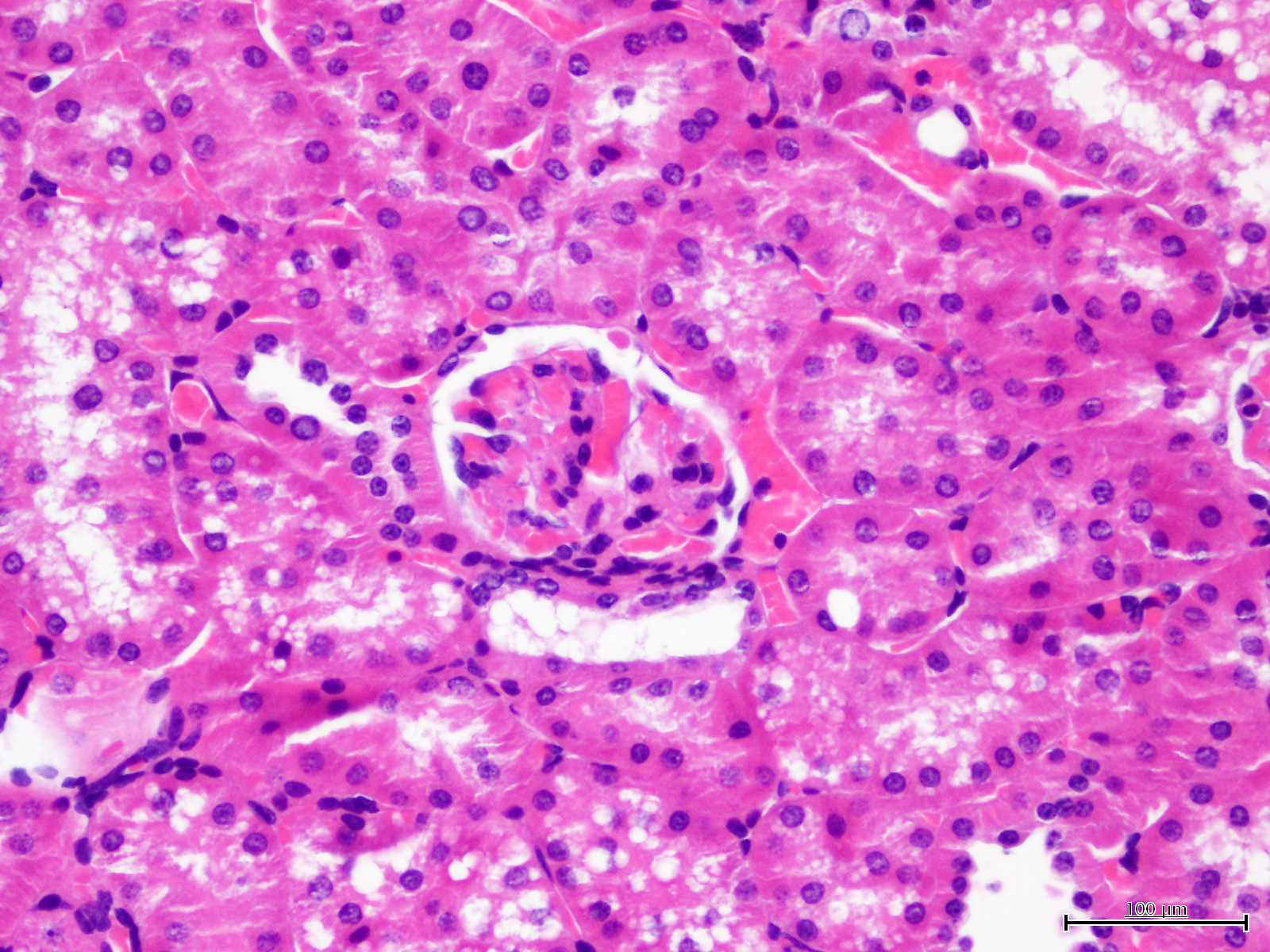

Supplement: S3 File — (ZIP) [file pone.0327042.s003.zip › S3 File - Image data underlying Fig 2A folder 2/HE-4w DM 75mGy/4w DM 75mGy-1.TIF]

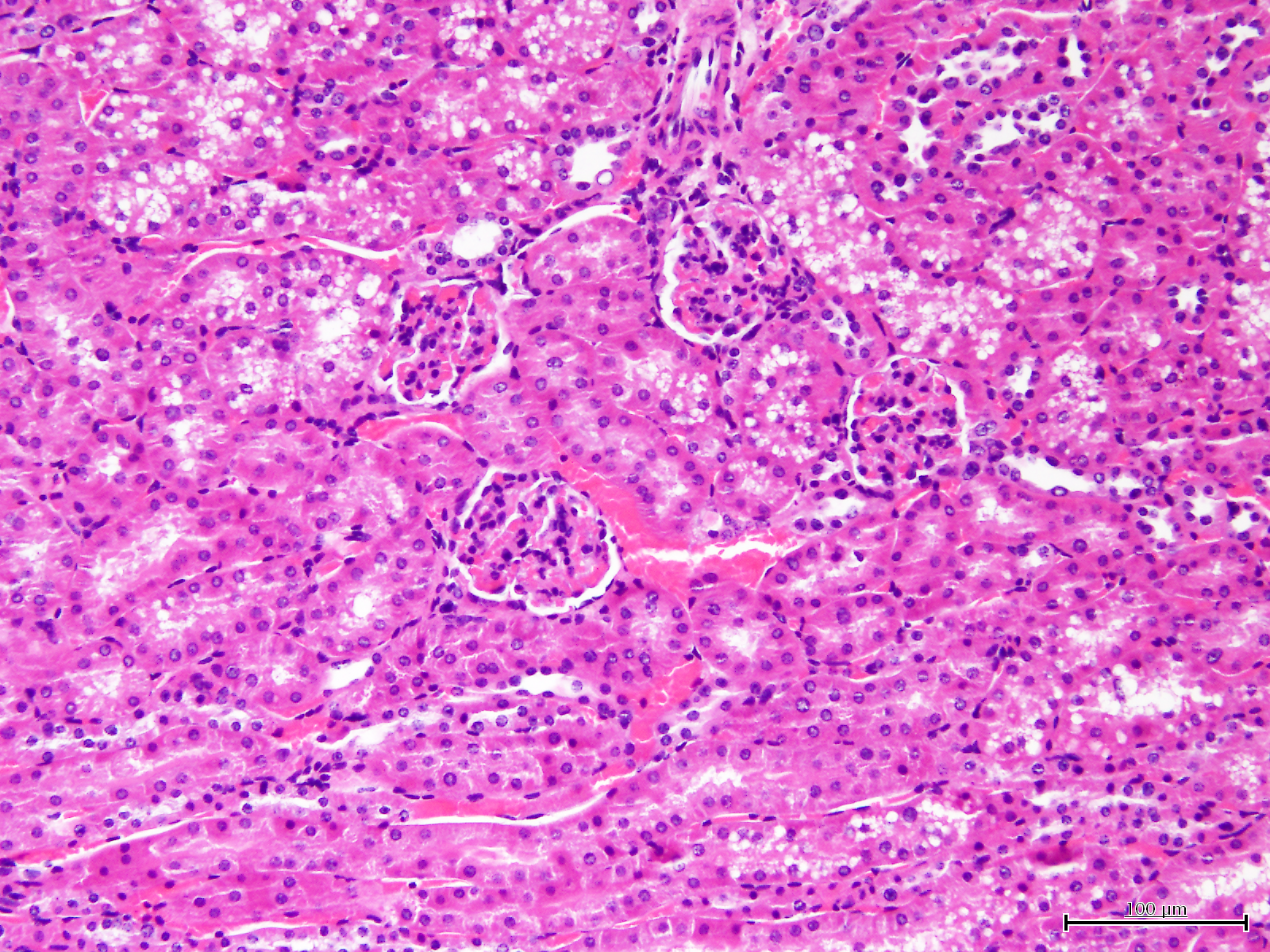

Supplement: S3 File — (ZIP) [file pone.0327042.s003.zip › S3 File - Image data underlying Fig 2A folder 2/HE-4w DM 75mGy/4w DM 75mGy-2 20x.TIF]

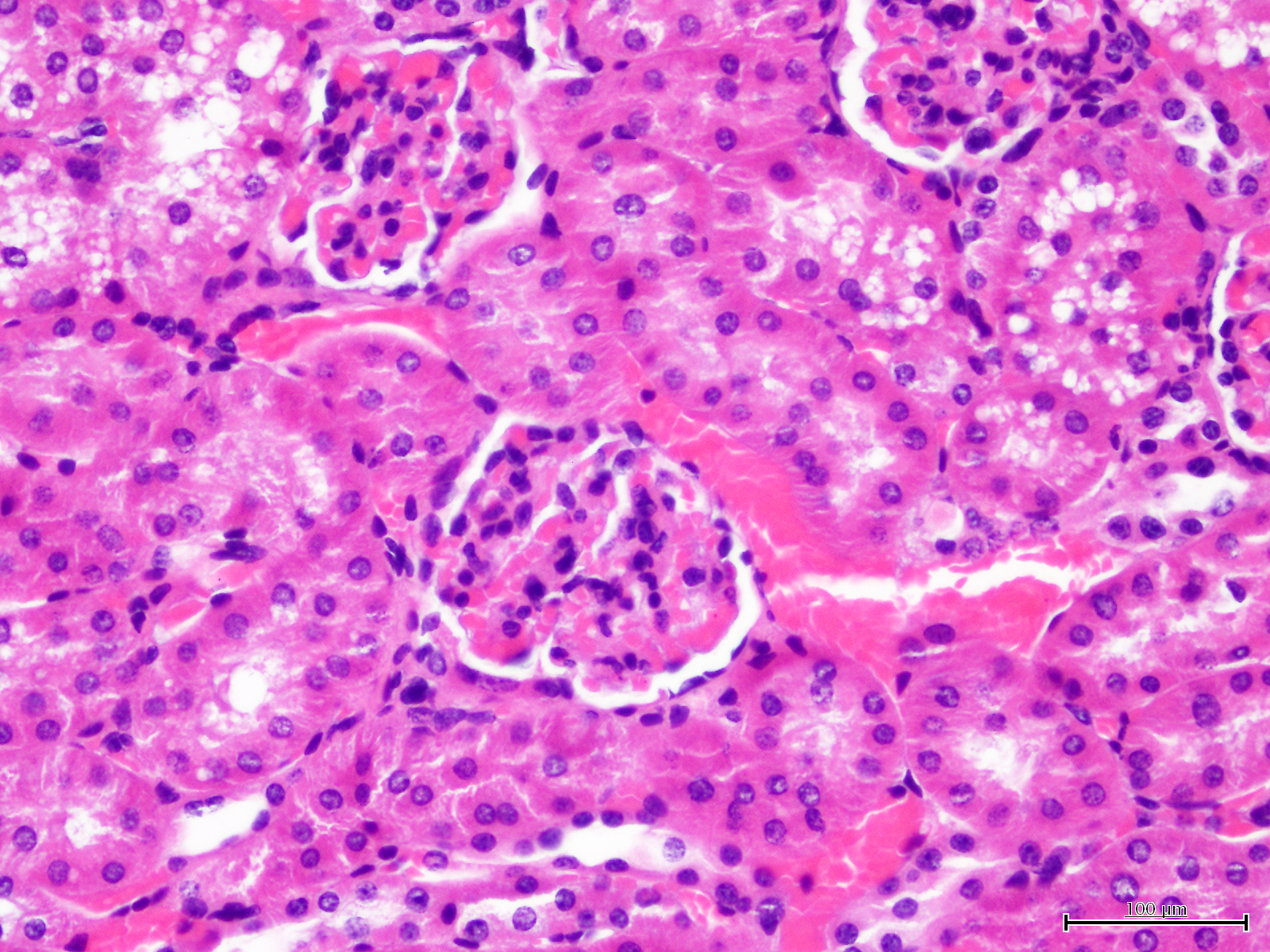

Supplement: S3 File — (ZIP) [file pone.0327042.s003.zip › S3 File - Image data underlying Fig 2A folder 2/HE-4w DM 75mGy/4w DM 75mGy-2.TIF]

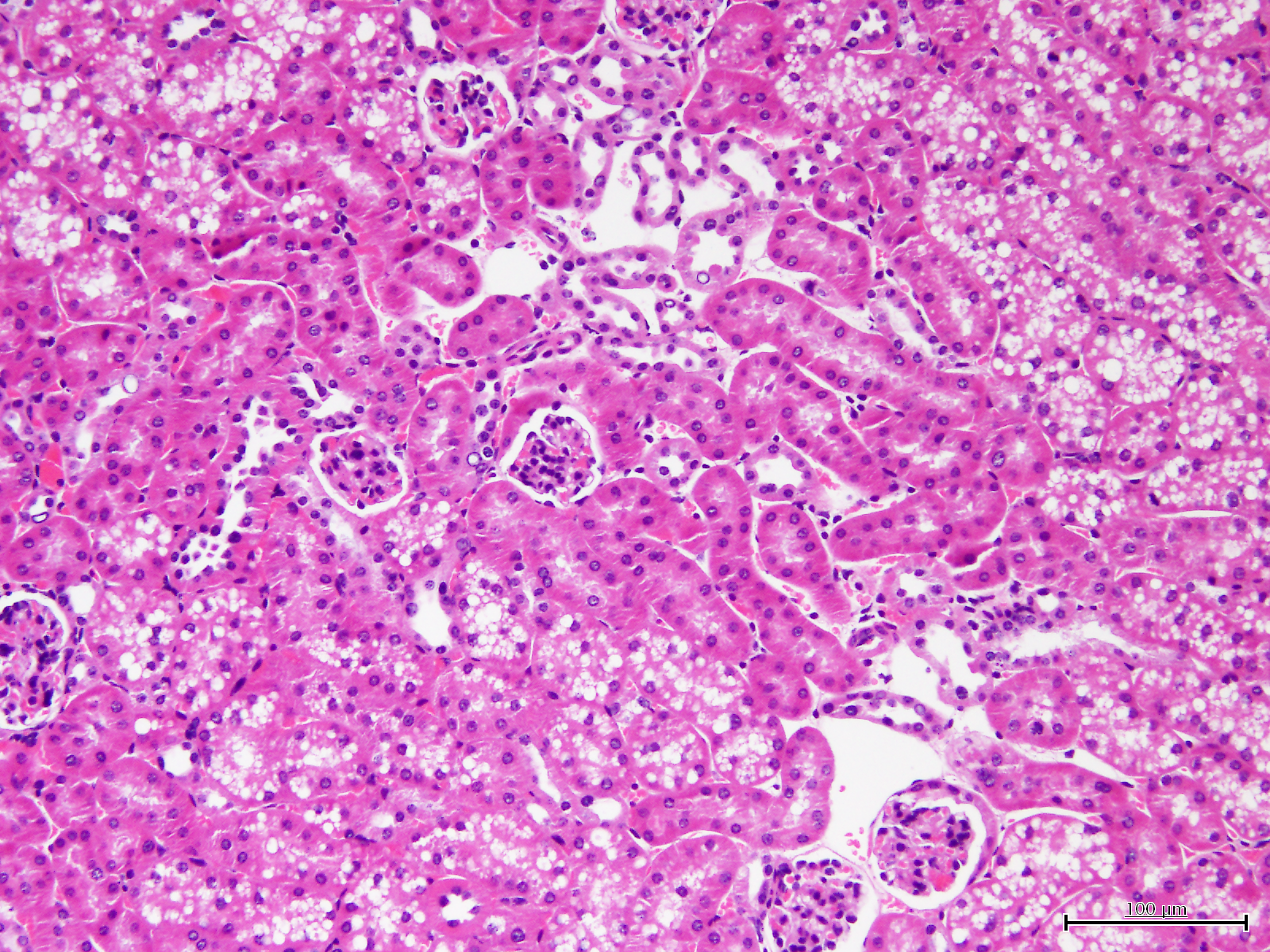

Supplement: S3 File — (ZIP) [file pone.0327042.s003.zip › S3 File - Image data underlying Fig 2A folder 2/HE-4w DM 75mGy/4w DM 75mGy-3 20x.TIF]

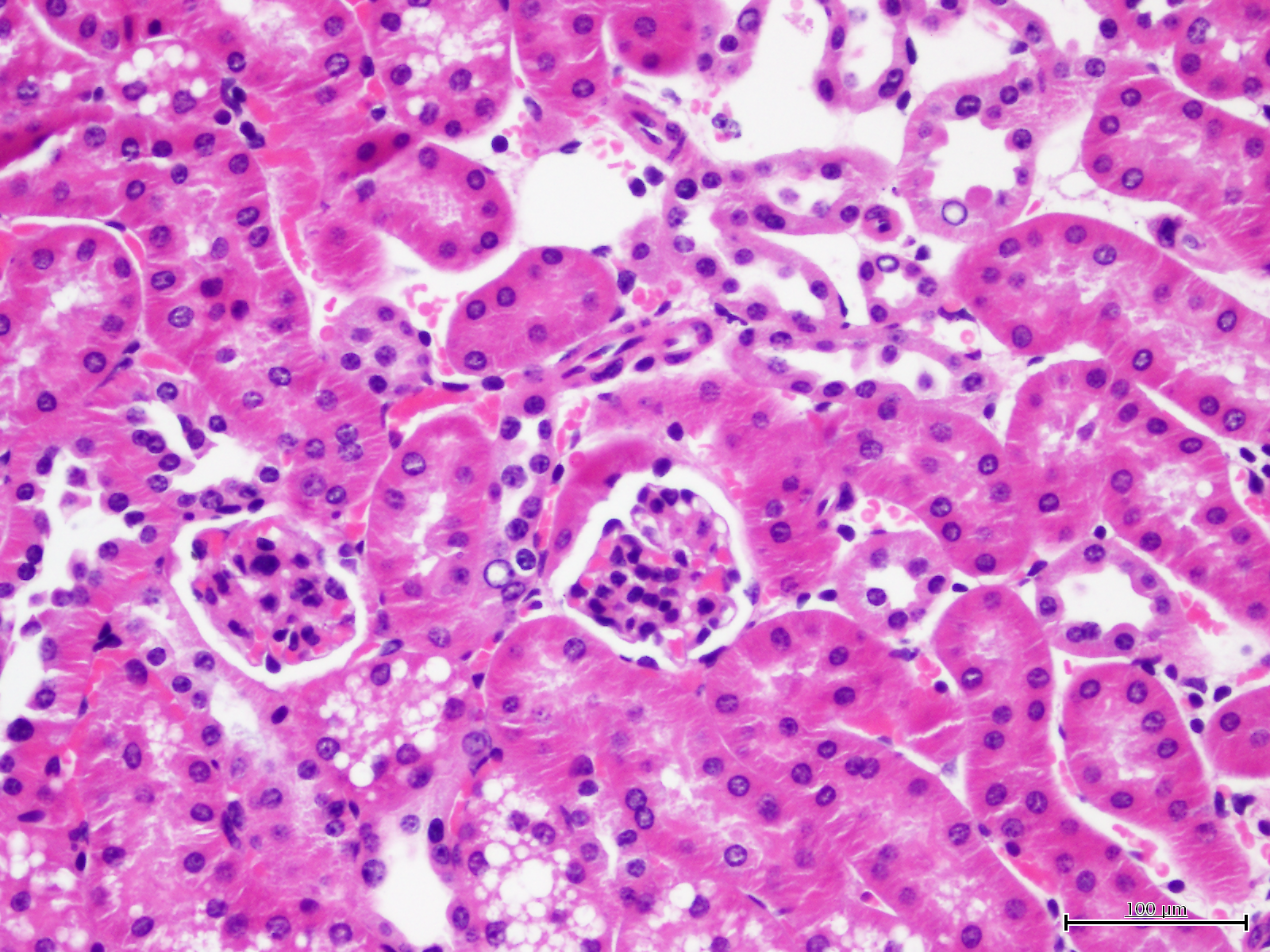

Supplement: S3 File — (ZIP) [file pone.0327042.s003.zip › S3 File - Image data underlying Fig 2A folder 2/HE-4w DM 75mGy/4w DM 75mGy-3.TIF]

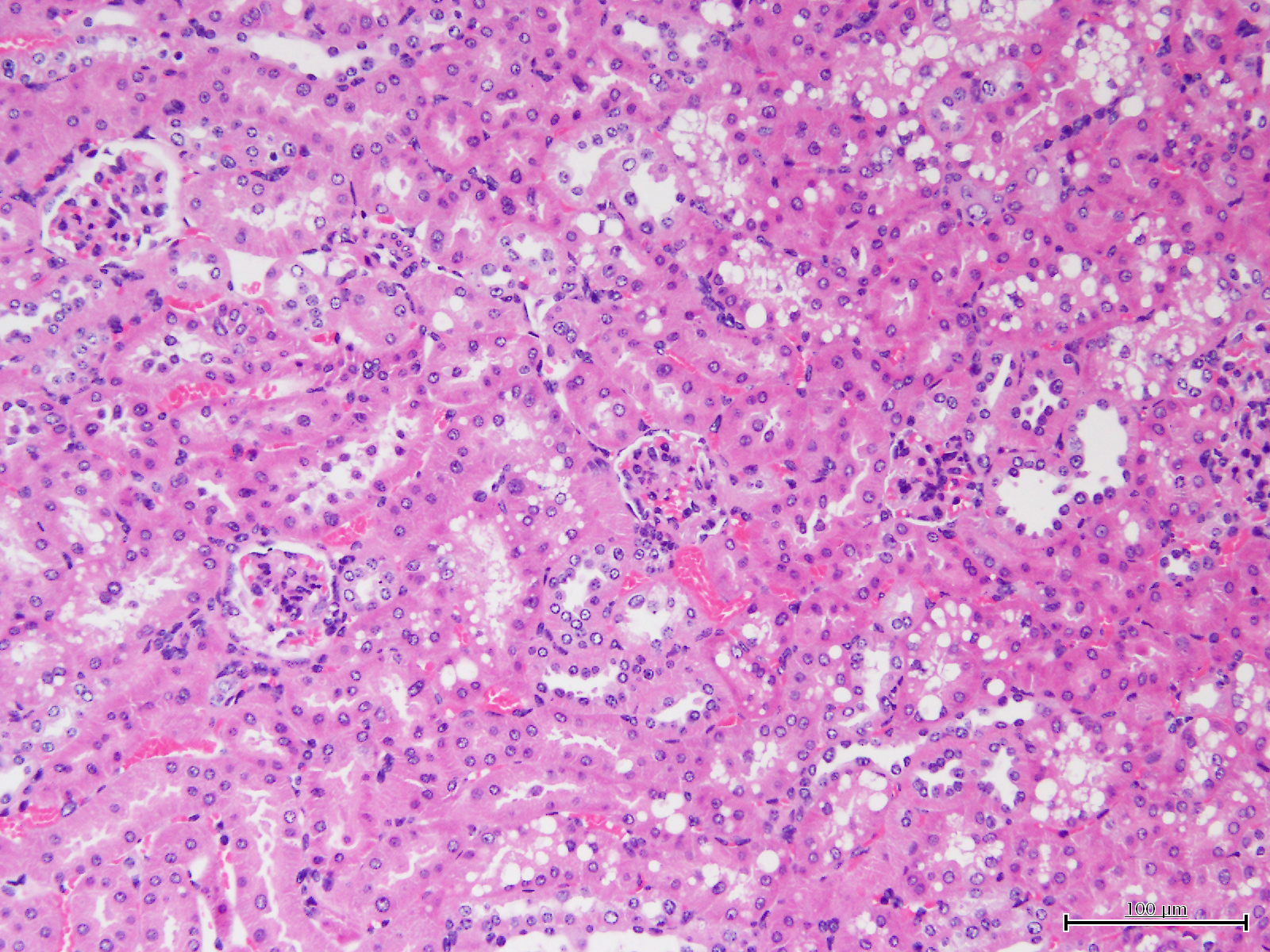

Supplement: S3 File — (ZIP) [file pone.0327042.s003.zip › S3 File - Image data underlying Fig 2A folder 2/HE-4w DM 75mGy/4w DM 75mGy-5 20x.TIF]

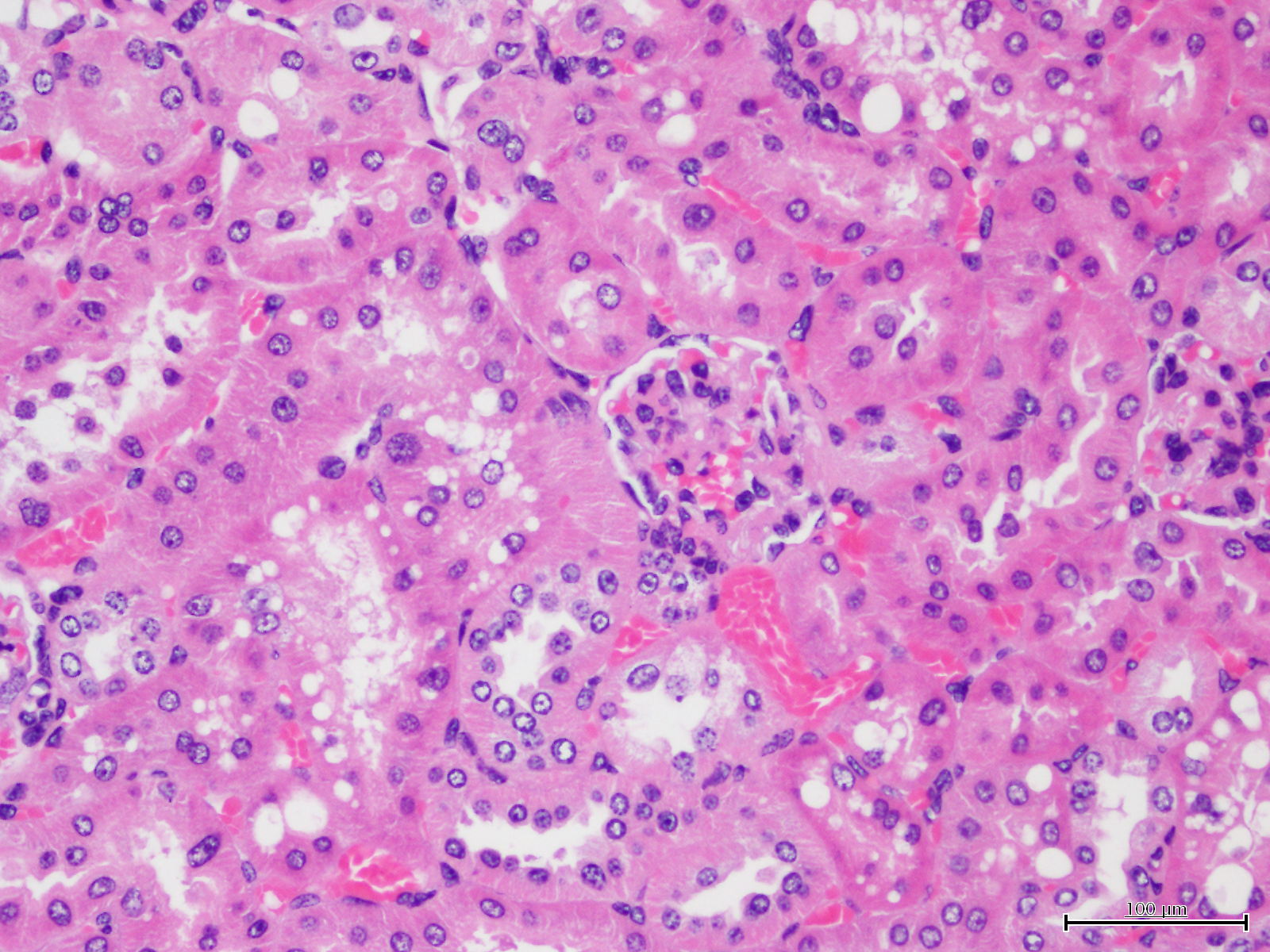

Supplement: S3 File — (ZIP) [file pone.0327042.s003.zip › S3 File - Image data underlying Fig 2A folder 2/HE-4w DM 75mGy/4w DM 75mGy-5.TIF]

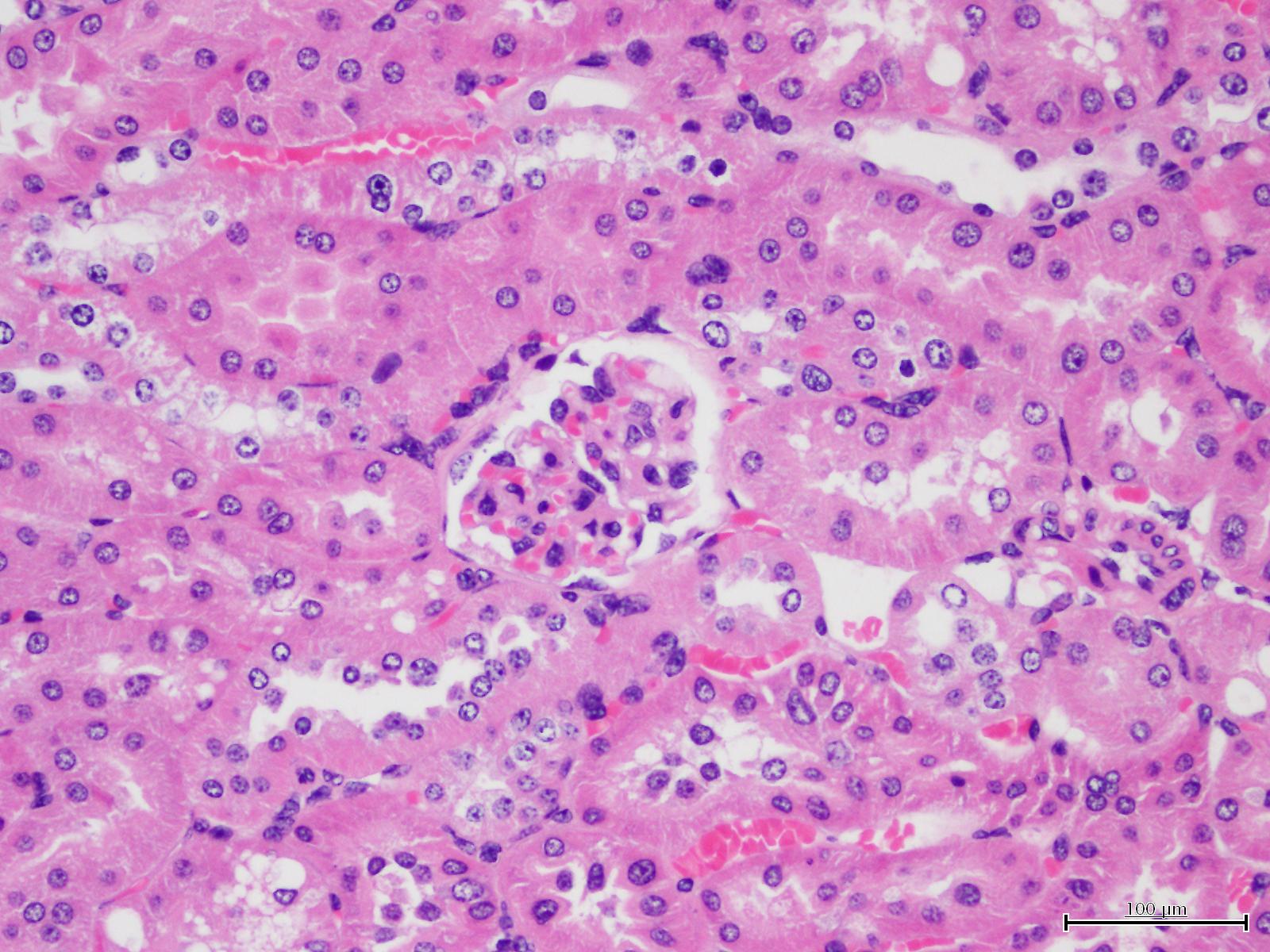

Supplement: S3 File — (ZIP) [file pone.0327042.s003.zip › S3 File - Image data underlying Fig 2A folder 2/HE-4w DM 75mGy/4w DM 75mGy-8 (Used publication).TIF]

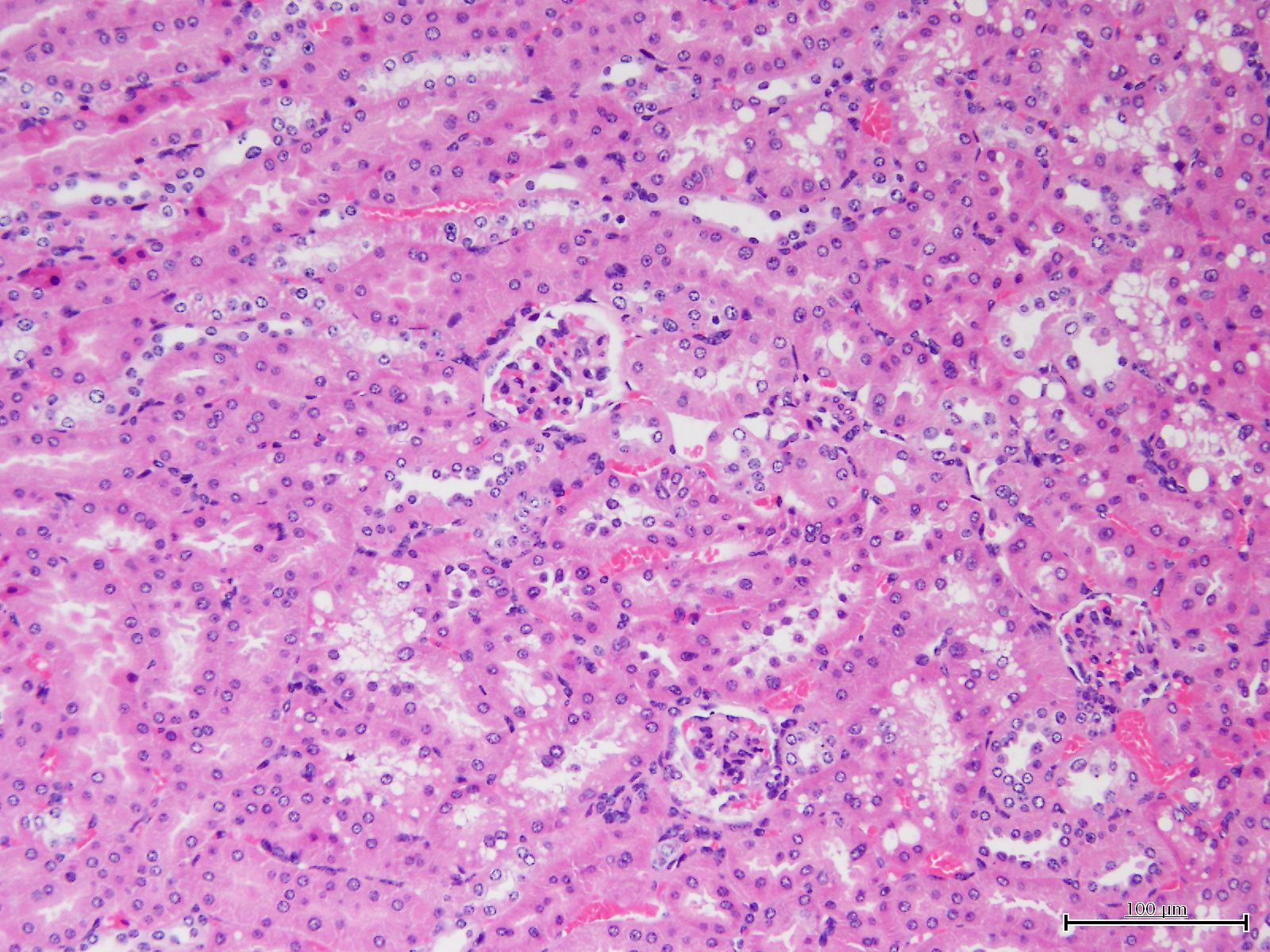

Supplement: S3 File — (ZIP) [file pone.0327042.s003.zip › S3 File - Image data underlying Fig 2A folder 2/HE-4w DM 75mGy/4w DM 75mGy-8 20x.TIF]

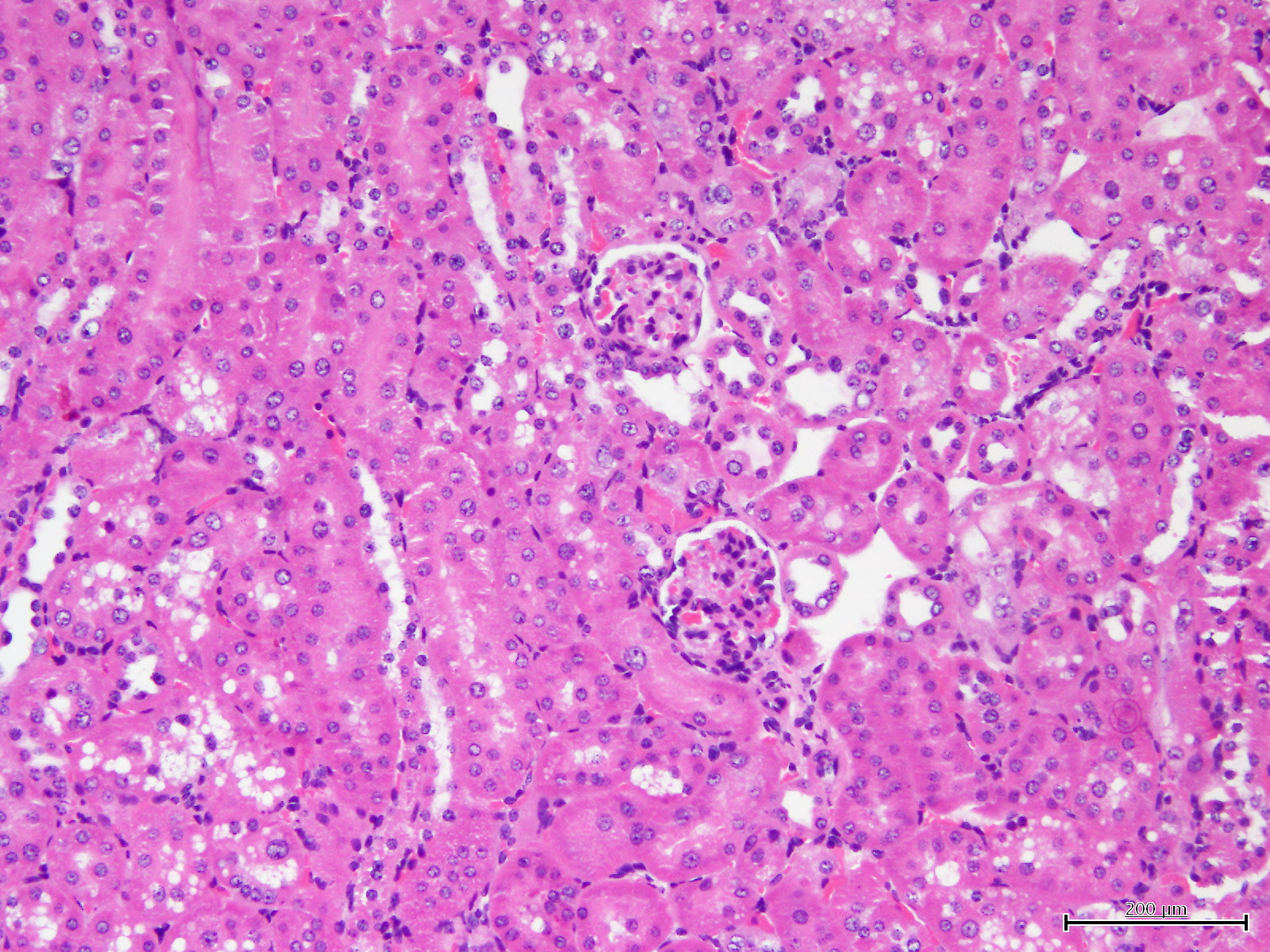

Supplement: S4 File — (ZIP) [file pone.0327042.s004.zip › HE-8w 25mGy DM/8w 25mGy DM-2 20x.JPG]

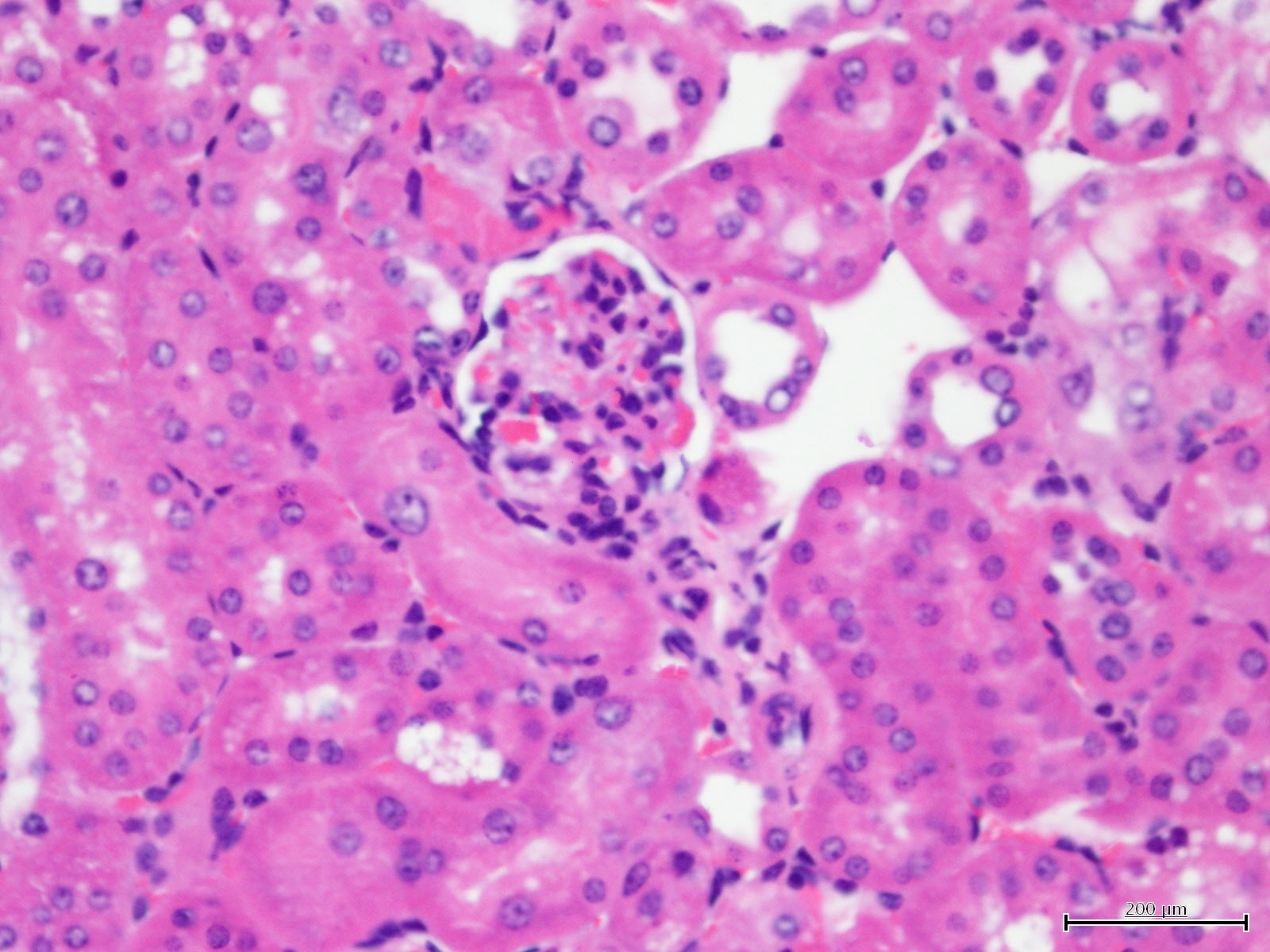

Supplement: S4 File — (ZIP) [file pone.0327042.s004.zip › HE-8w 25mGy DM/8w 25mGy DM-2.JPG]

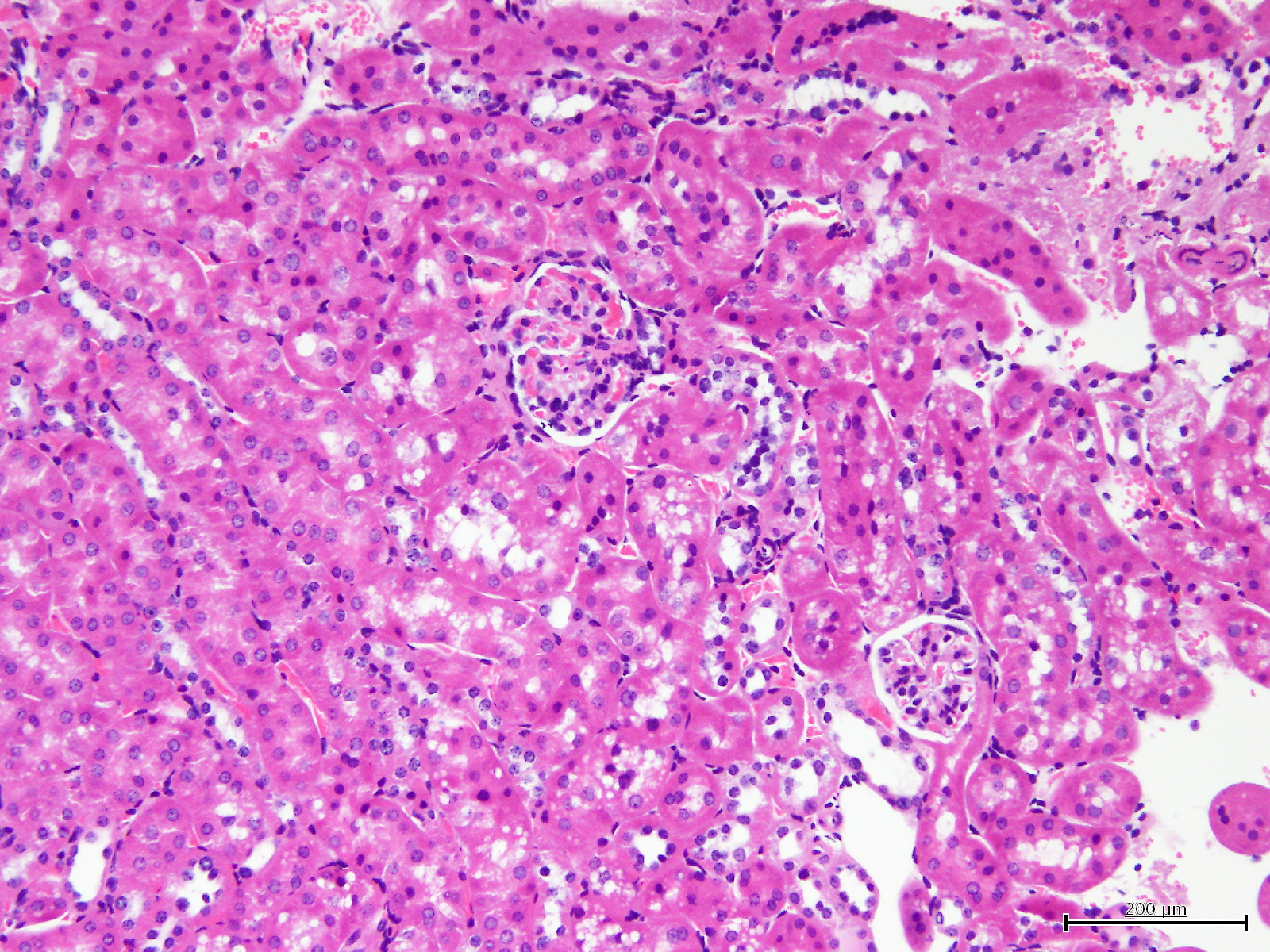

Supplement: S4 File — (ZIP) [file pone.0327042.s004.zip › HE-8w 25mGy DM/8w 25mGy DM-4 20x.JPG]

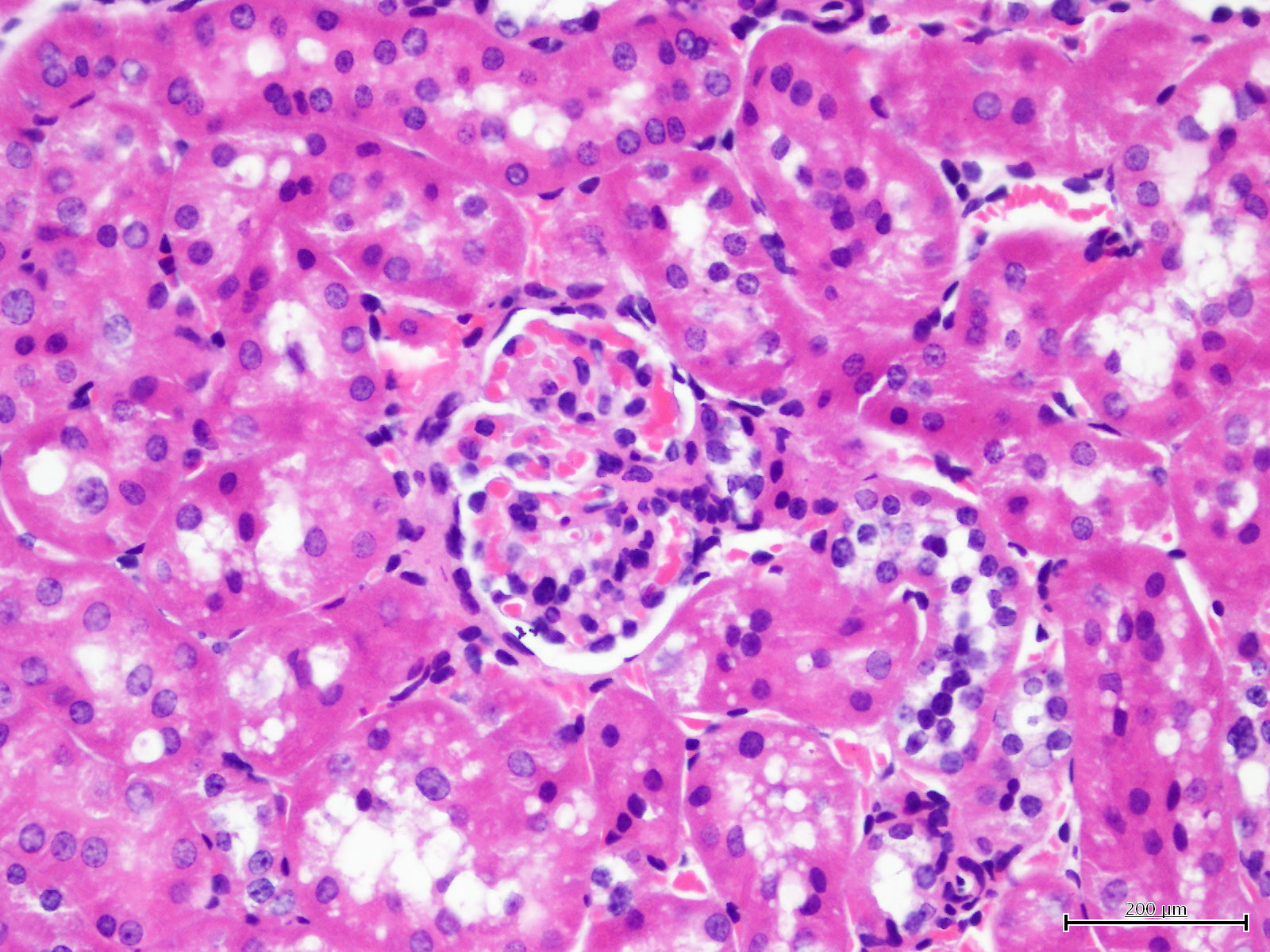

Supplement: S4 File — (ZIP) [file pone.0327042.s004.zip › HE-8w 25mGy DM/8w 25mGy DM-4(Used publication).JPG]

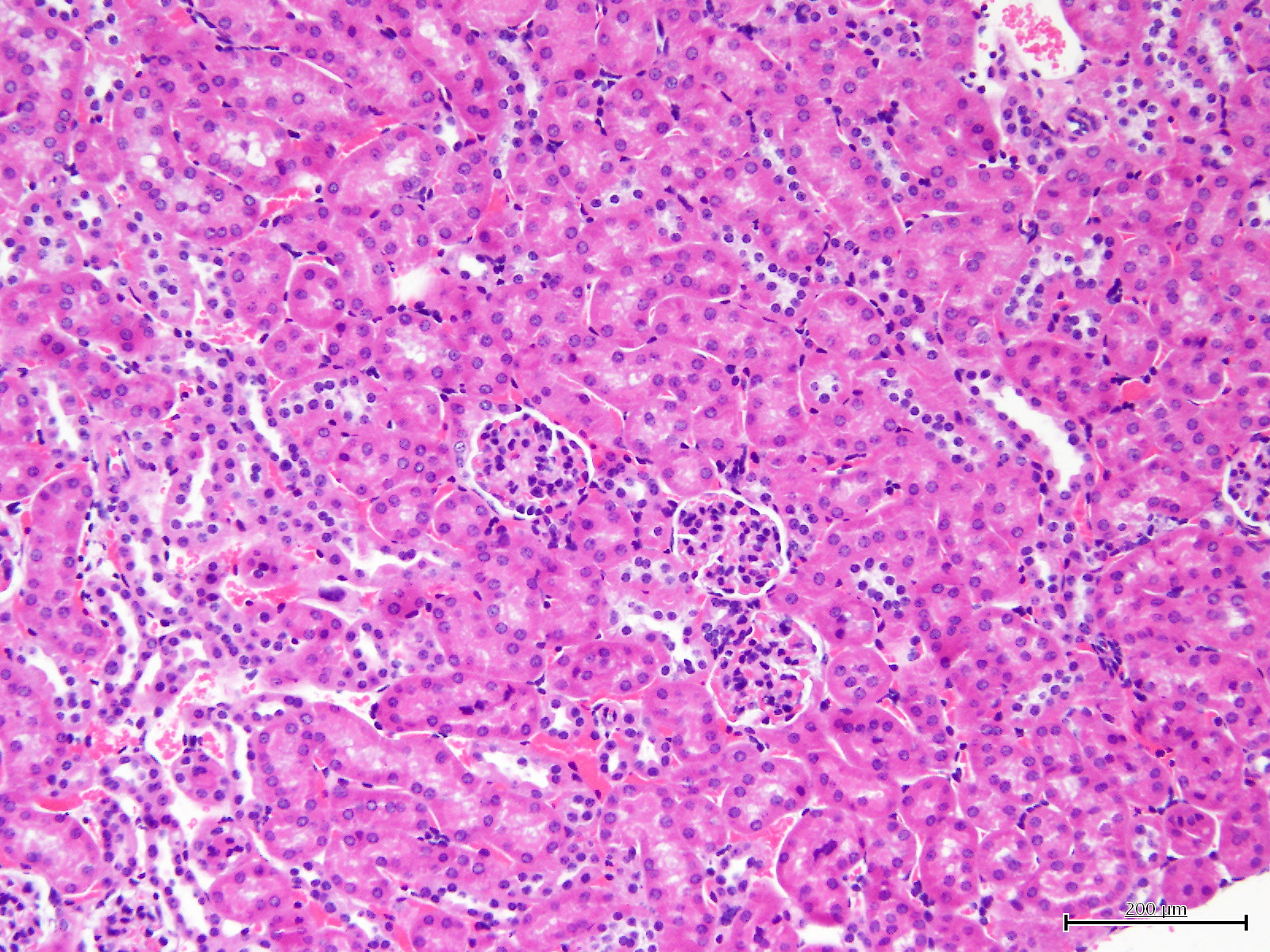

Supplement: S4 File — (ZIP) [file pone.0327042.s004.zip › HE-8w 50mGy Con/8w 50mGy Con-1 20x.JPG]

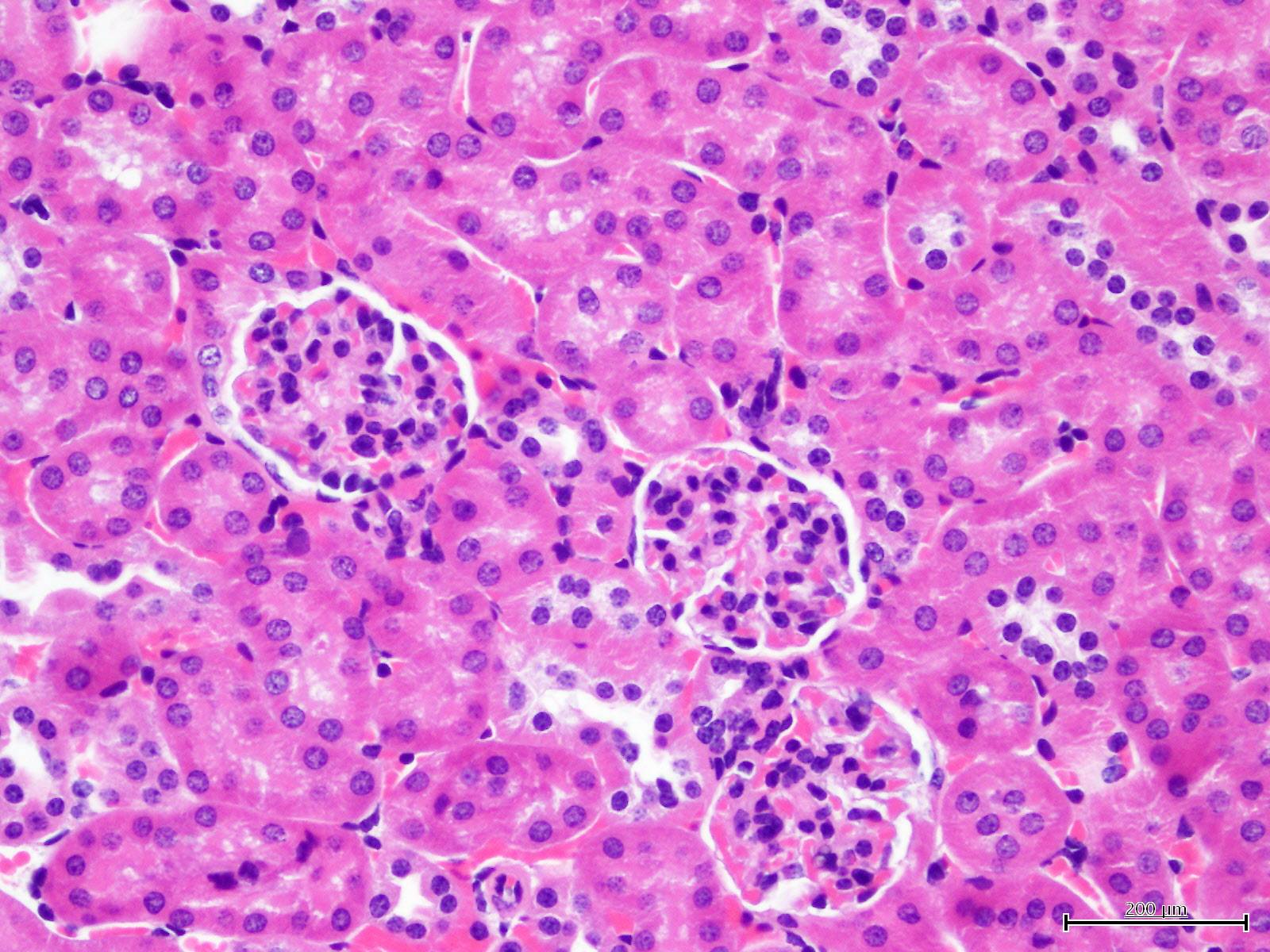

Supplement: S4 File — (ZIP) [file pone.0327042.s004.zip › HE-8w 50mGy Con/8w 50mGy Con-1.JPG]

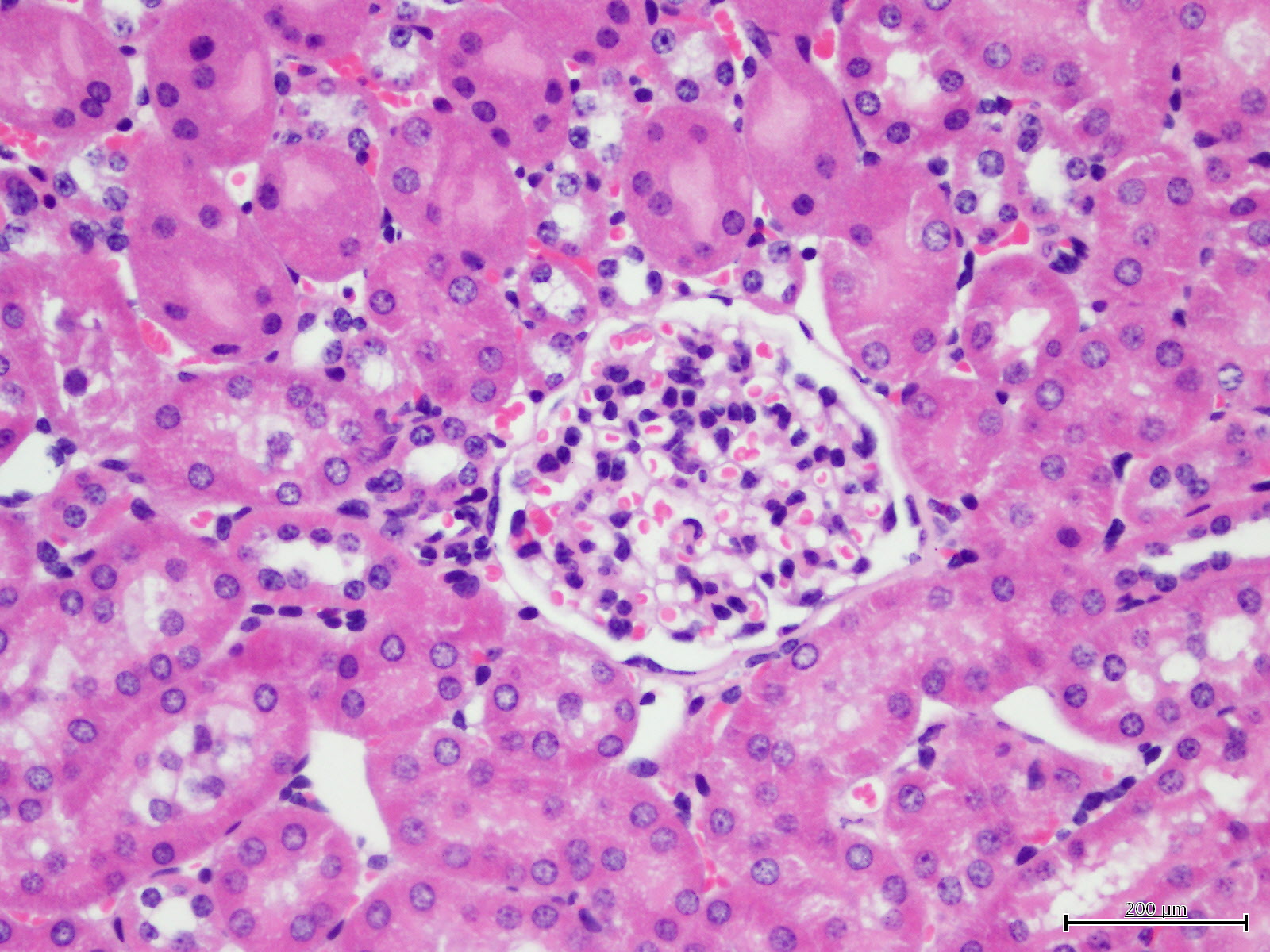

Supplement: S4 File — (ZIP) [file pone.0327042.s004.zip › HE-8w 50mGy Con/8w 50mGy Con-4 (Used publication).JPG]

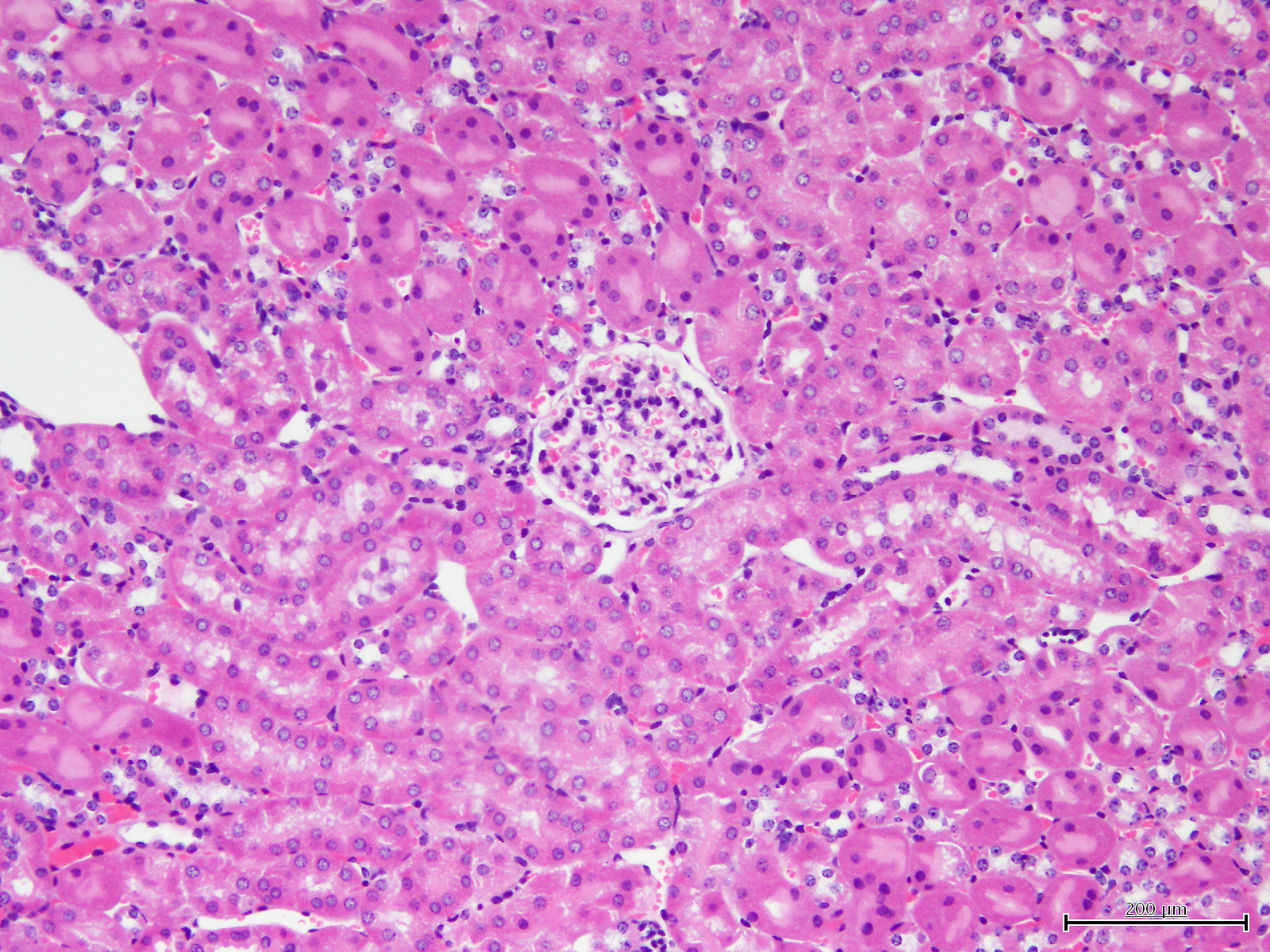

Supplement: S4 File — (ZIP) [file pone.0327042.s004.zip › HE-8w 50mGy Con/8w 50mGy Con-4.JPG]

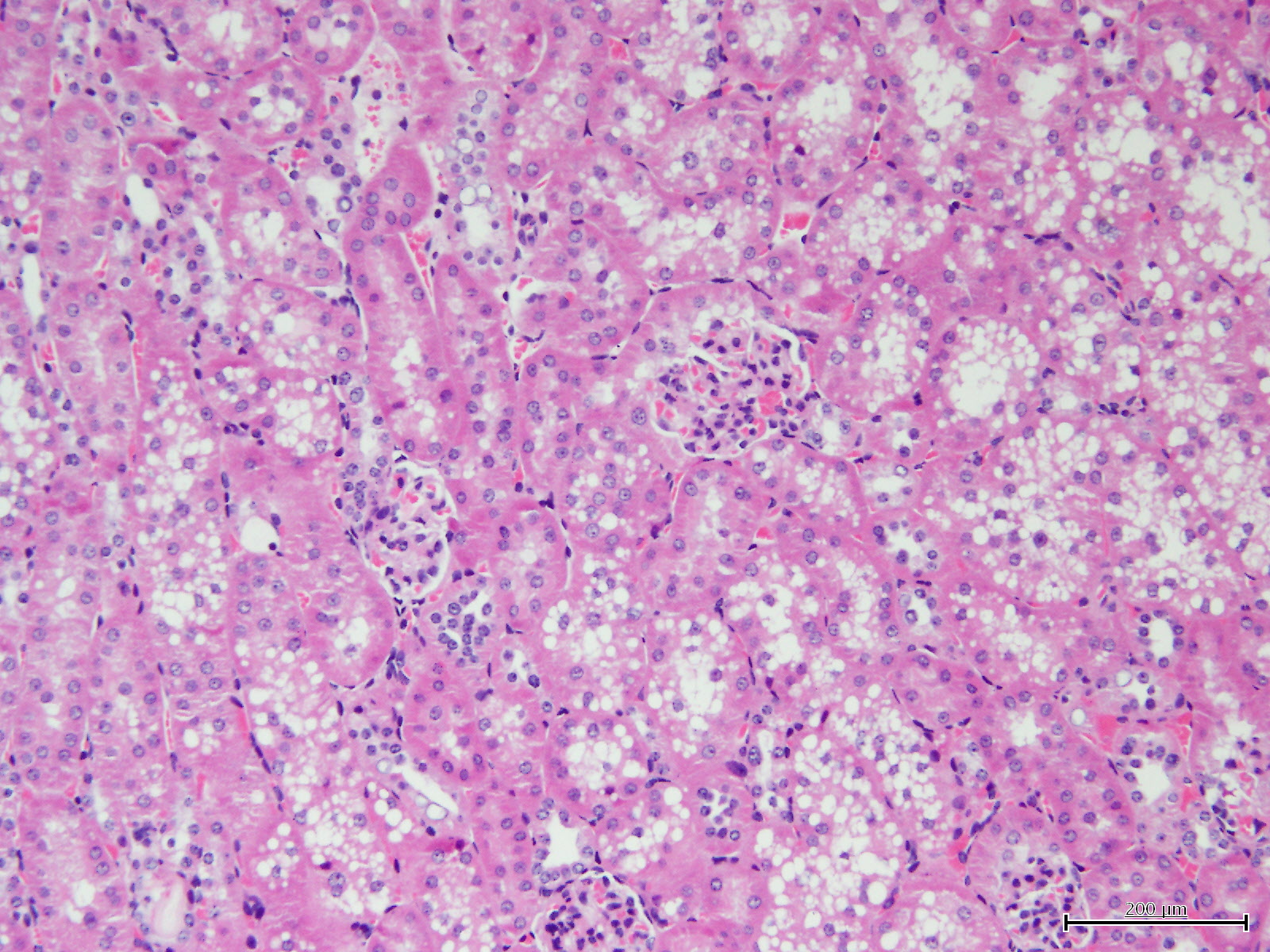

Supplement: S4 File — (ZIP) [file pone.0327042.s004.zip › HE-8w 50mGy DM/8w 50mGy DM-1 20x.JPG]

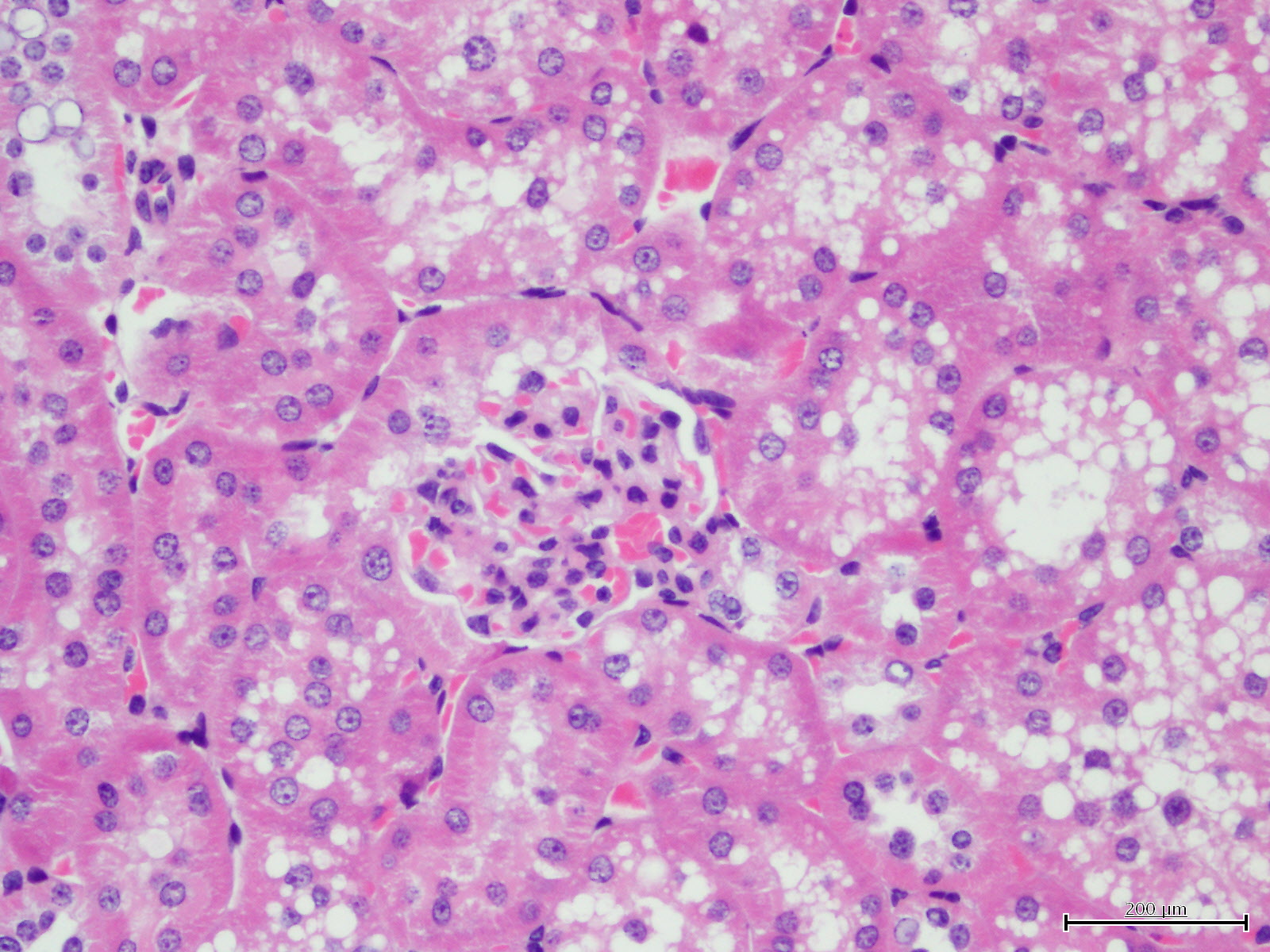

Supplement: S4 File — (ZIP) [file pone.0327042.s004.zip › HE-8w 50mGy DM/8w 50mGy DM-2.JPG]

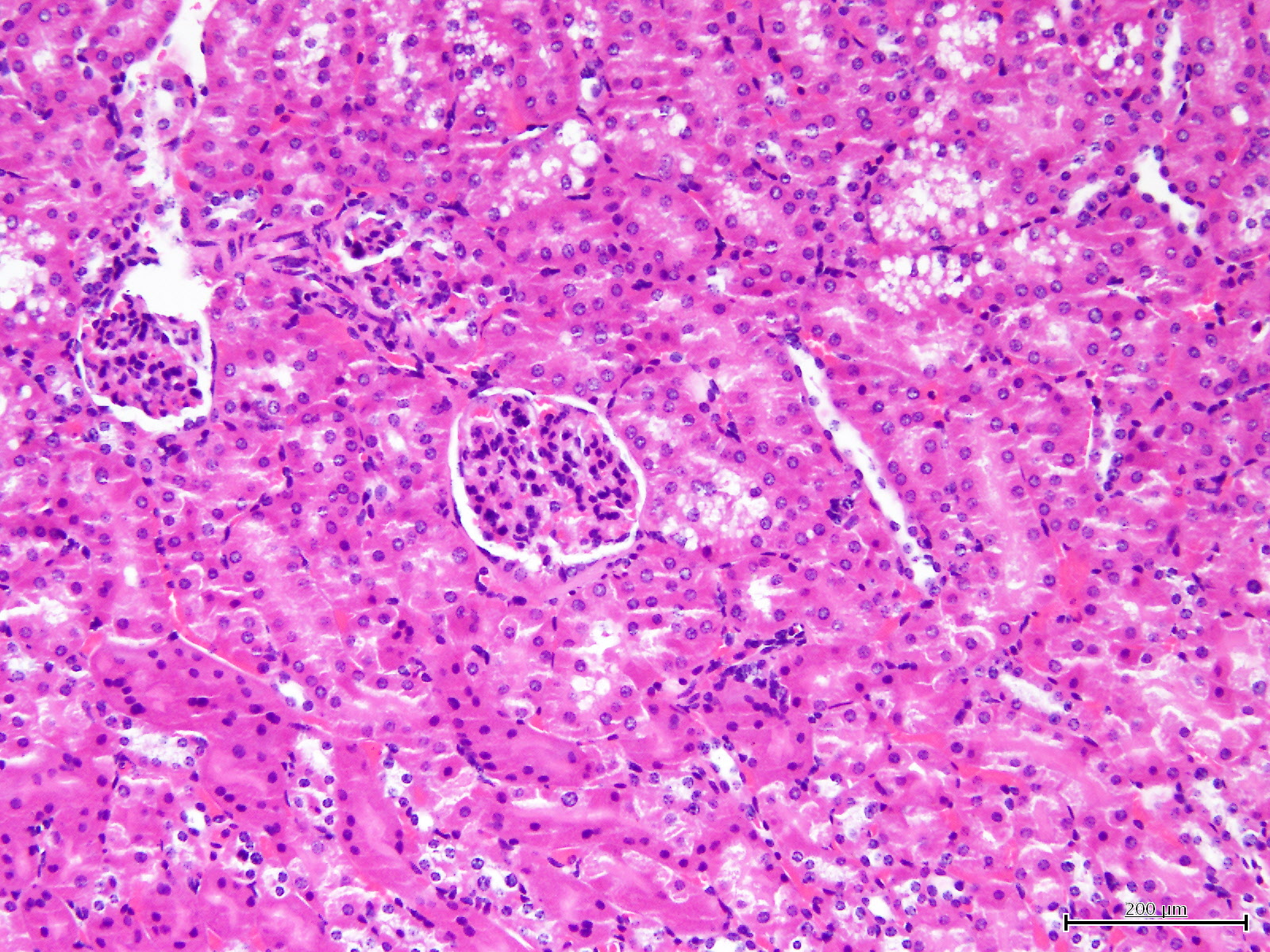

Supplement: S4 File — (ZIP) [file pone.0327042.s004.zip › HE-8w 50mGy DM/8w 50mGy DM-3 20x.JPG]

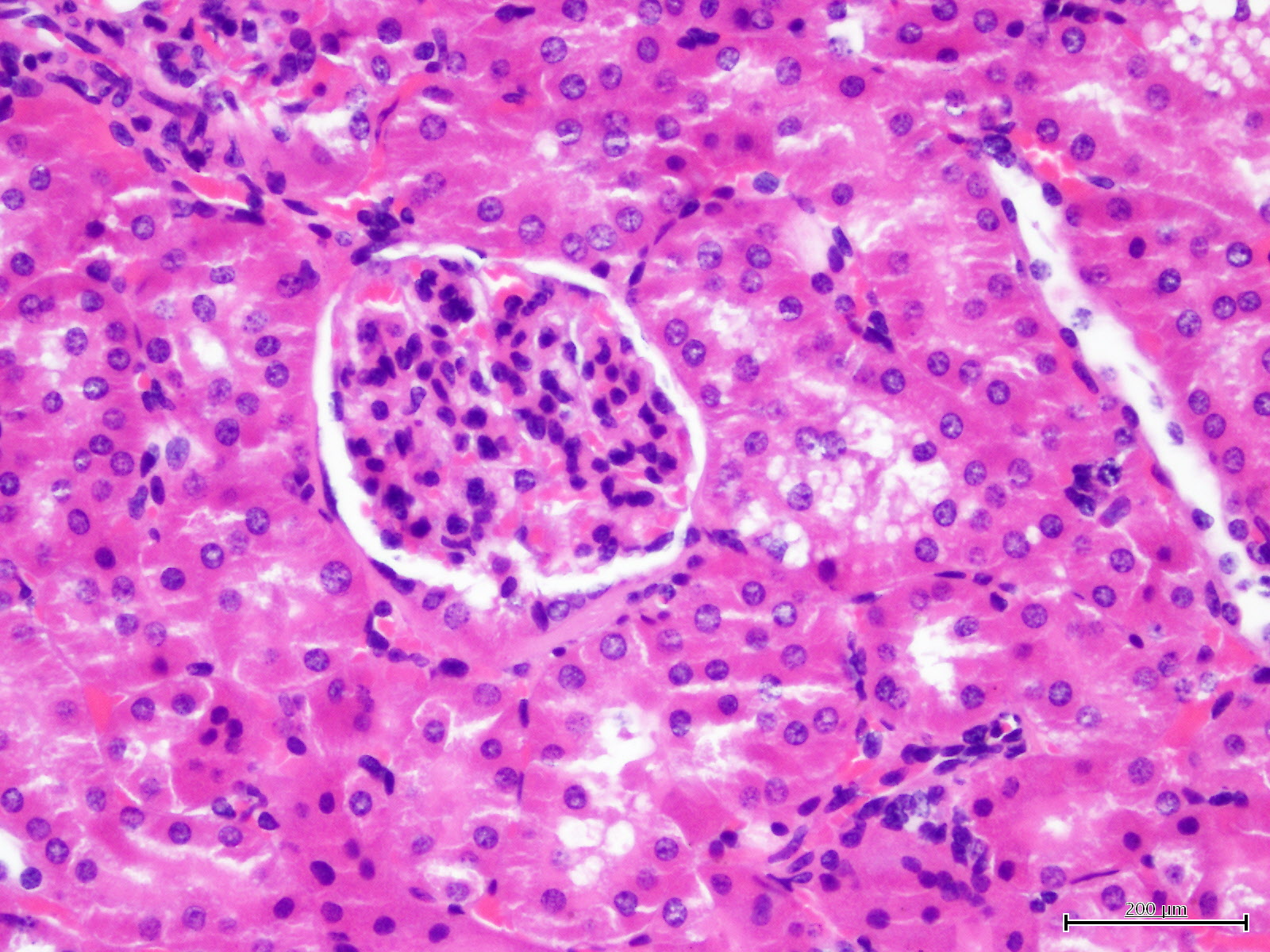

Supplement: S4 File — (ZIP) [file pone.0327042.s004.zip › HE-8w 50mGy DM/8w 50mGy DM-3.JPG]

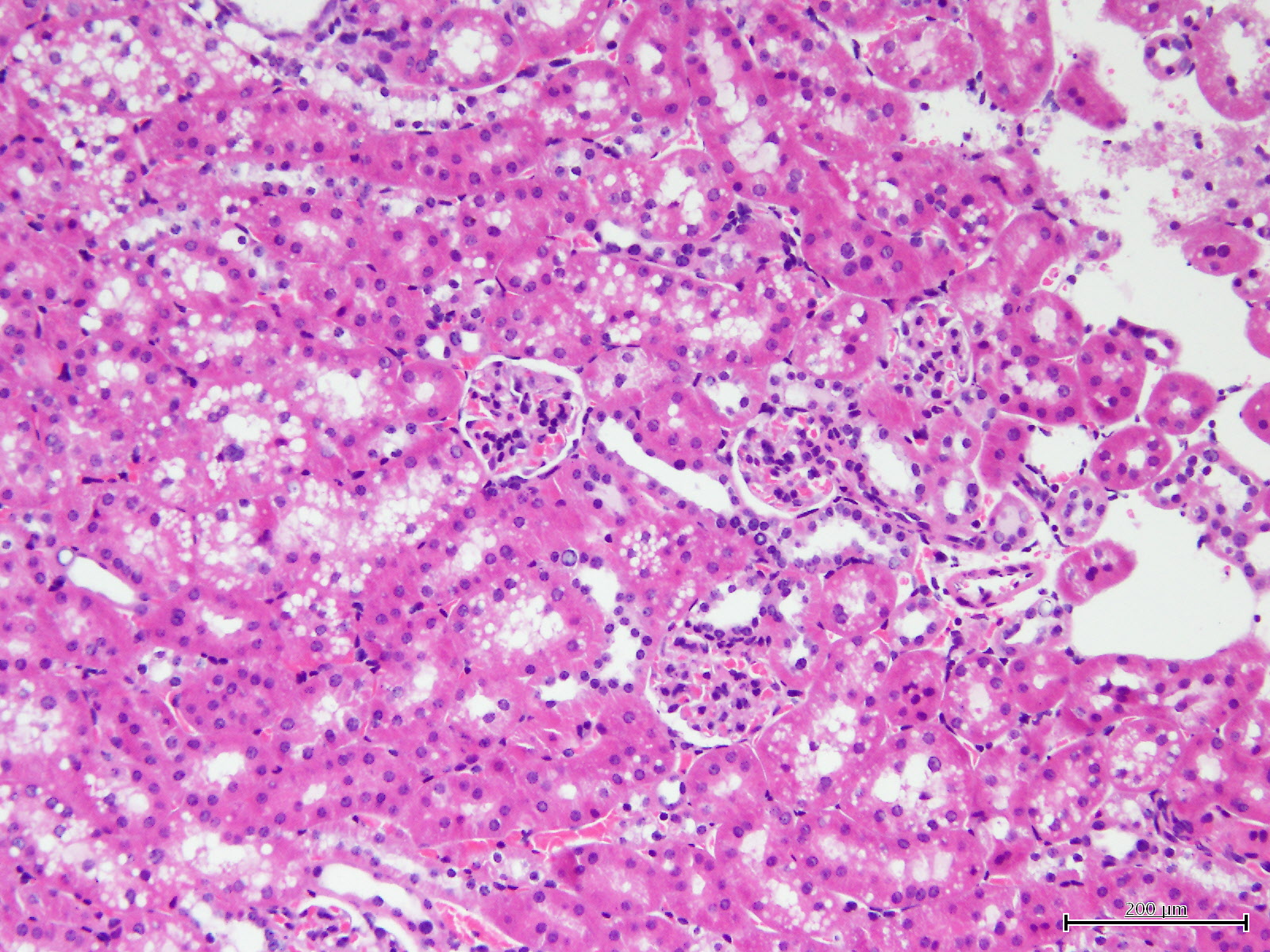

Supplement: S4 File — (ZIP) [file pone.0327042.s004.zip › HE-8w 50mGy DM/8w 50mGy DM-5 20x.JPG]

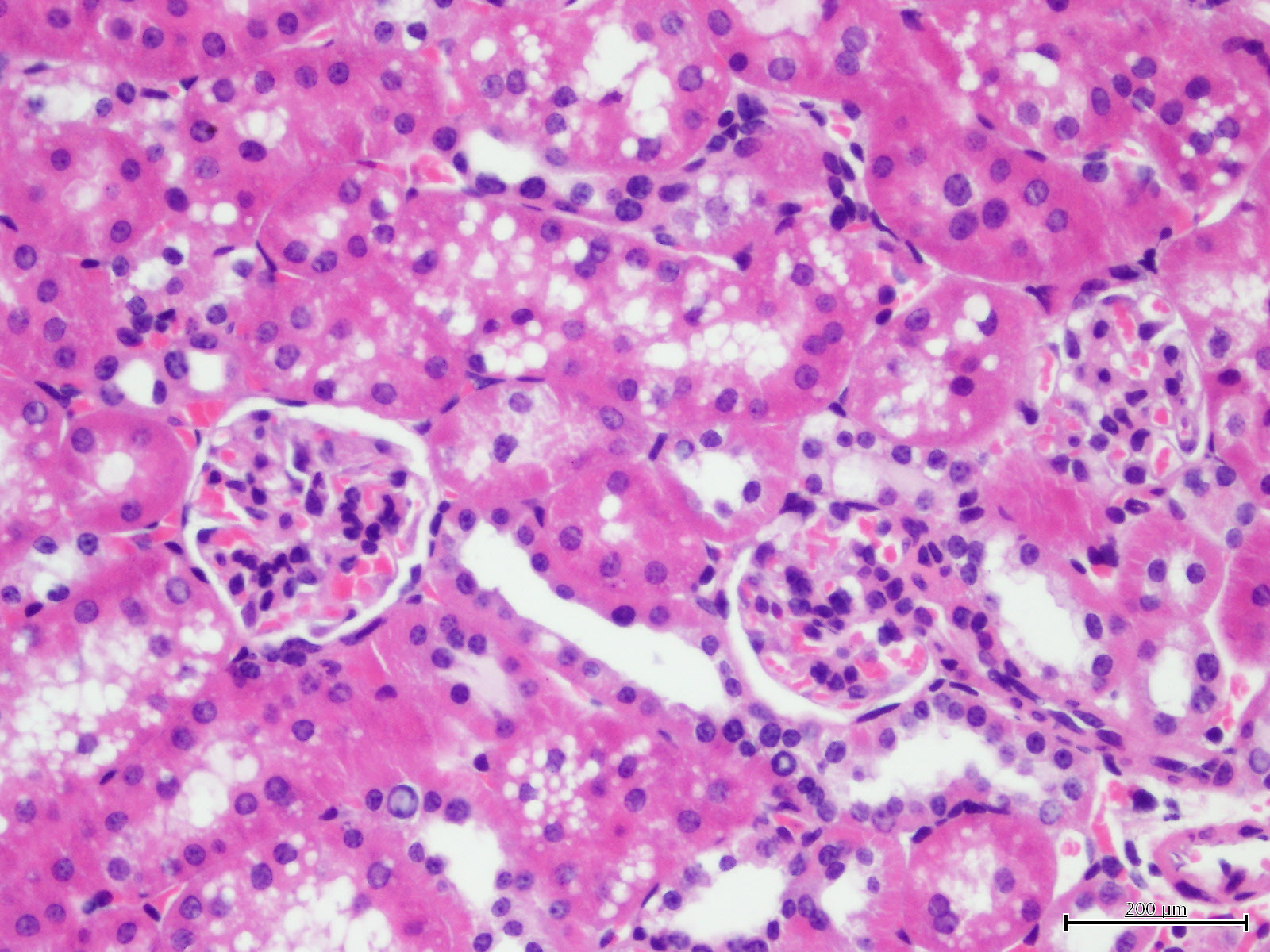

Supplement: S4 File — (ZIP) [file pone.0327042.s004.zip › HE-8w 50mGy DM/8w 50mGy DM-5.JPG]

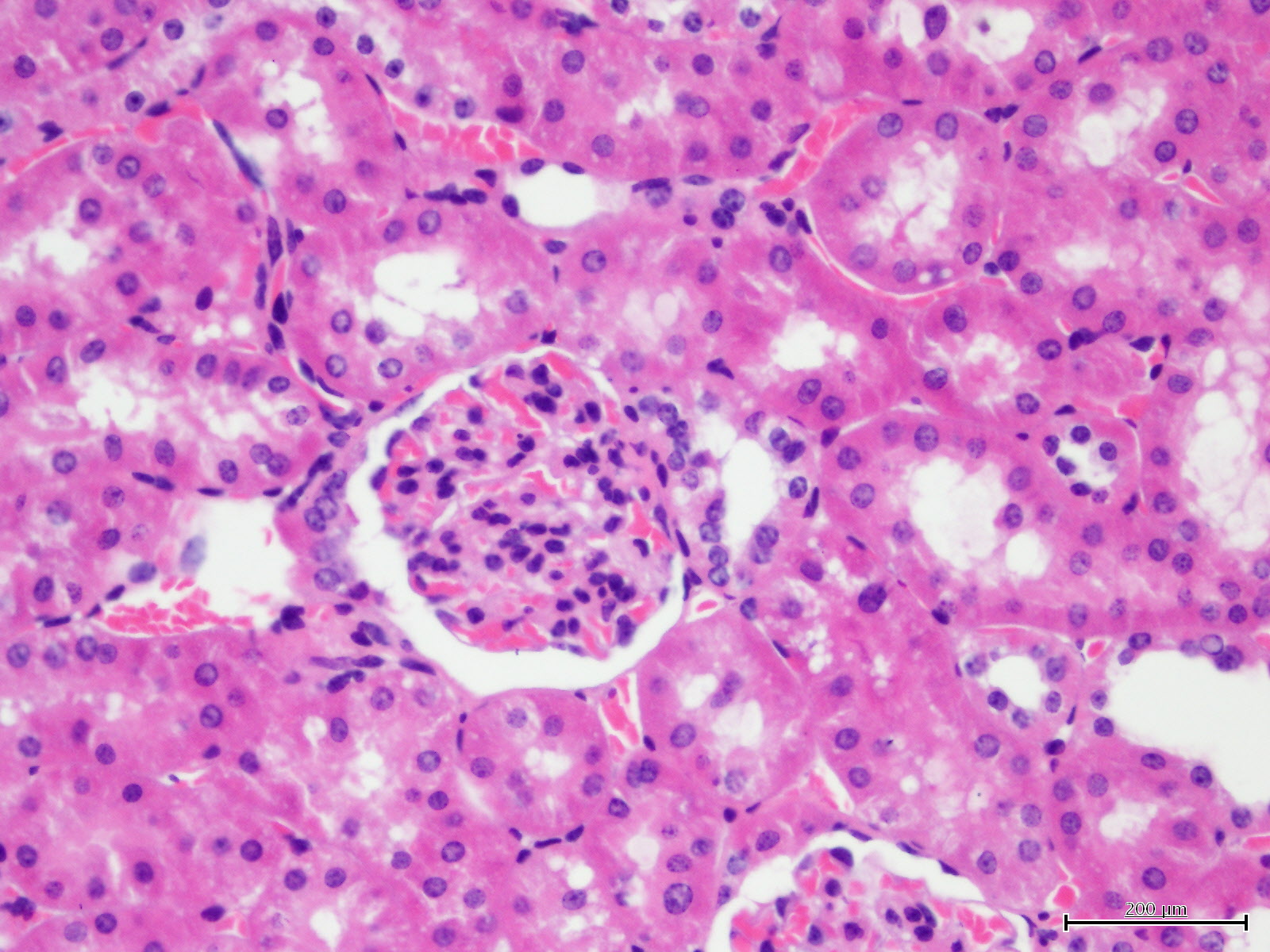

Supplement: S4 File — (ZIP) [file pone.0327042.s004.zip › HE-8w 50mGy DM/8w 50mGy DM-6 (Used publication).JPG]

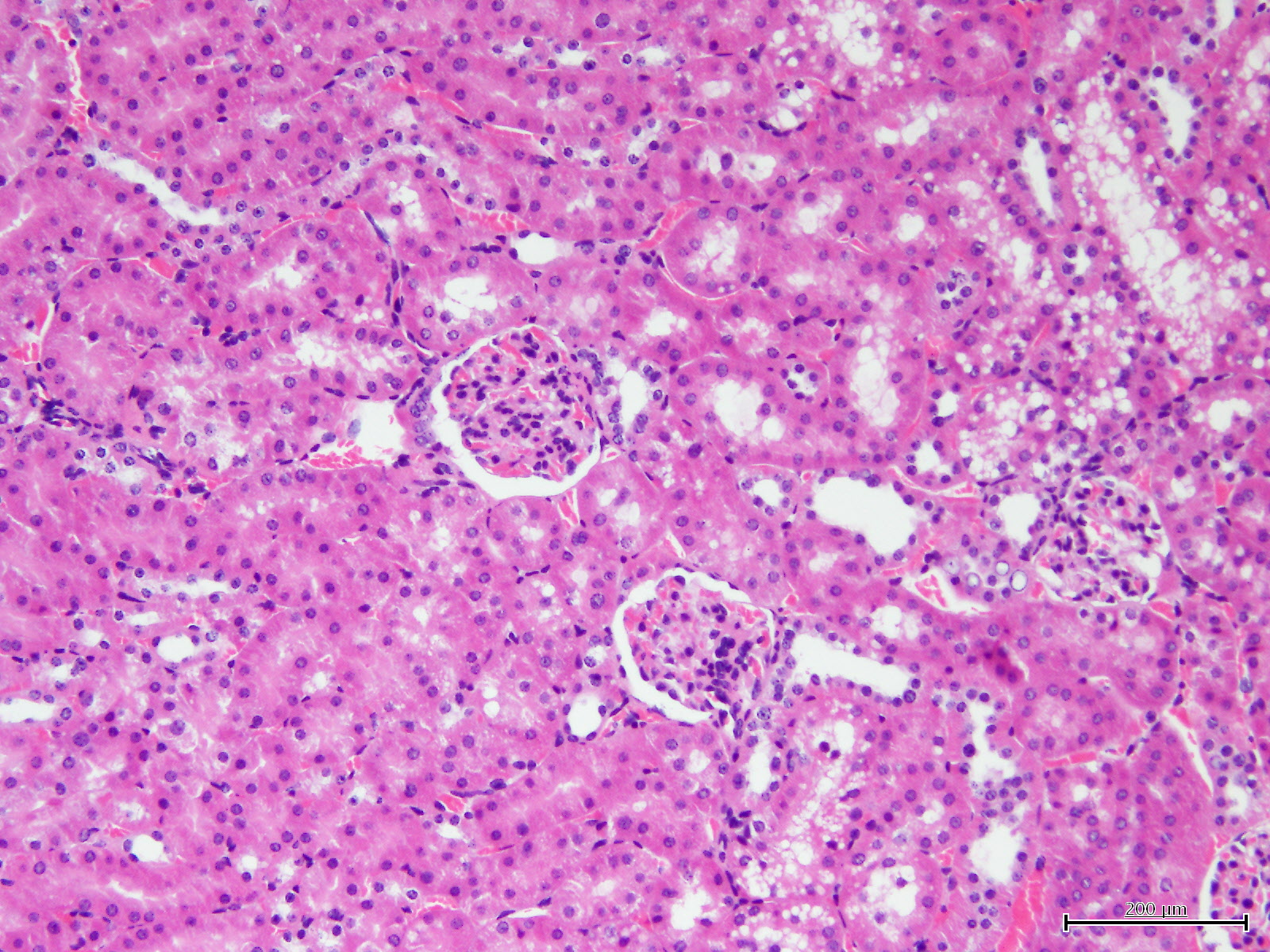

Supplement: S4 File — (ZIP) [file pone.0327042.s004.zip › HE-8w 50mGy DM/8w 50mGy DM-6 20x.JPG]

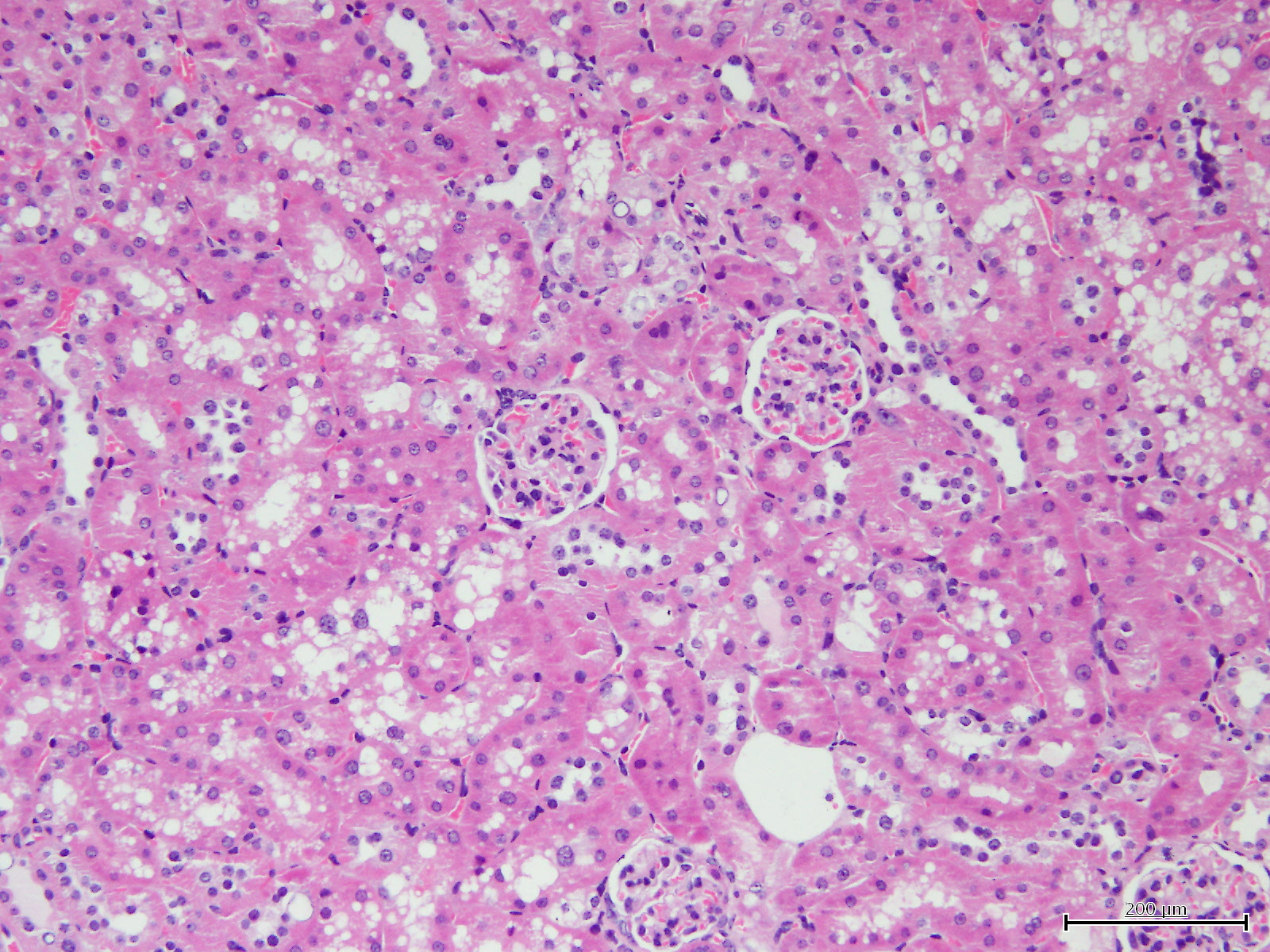

Supplement: S4 File — (ZIP) [file pone.0327042.s004.zip › HE-8w 75mGy DM/8w 75mGy DM-1 20x.JPG]

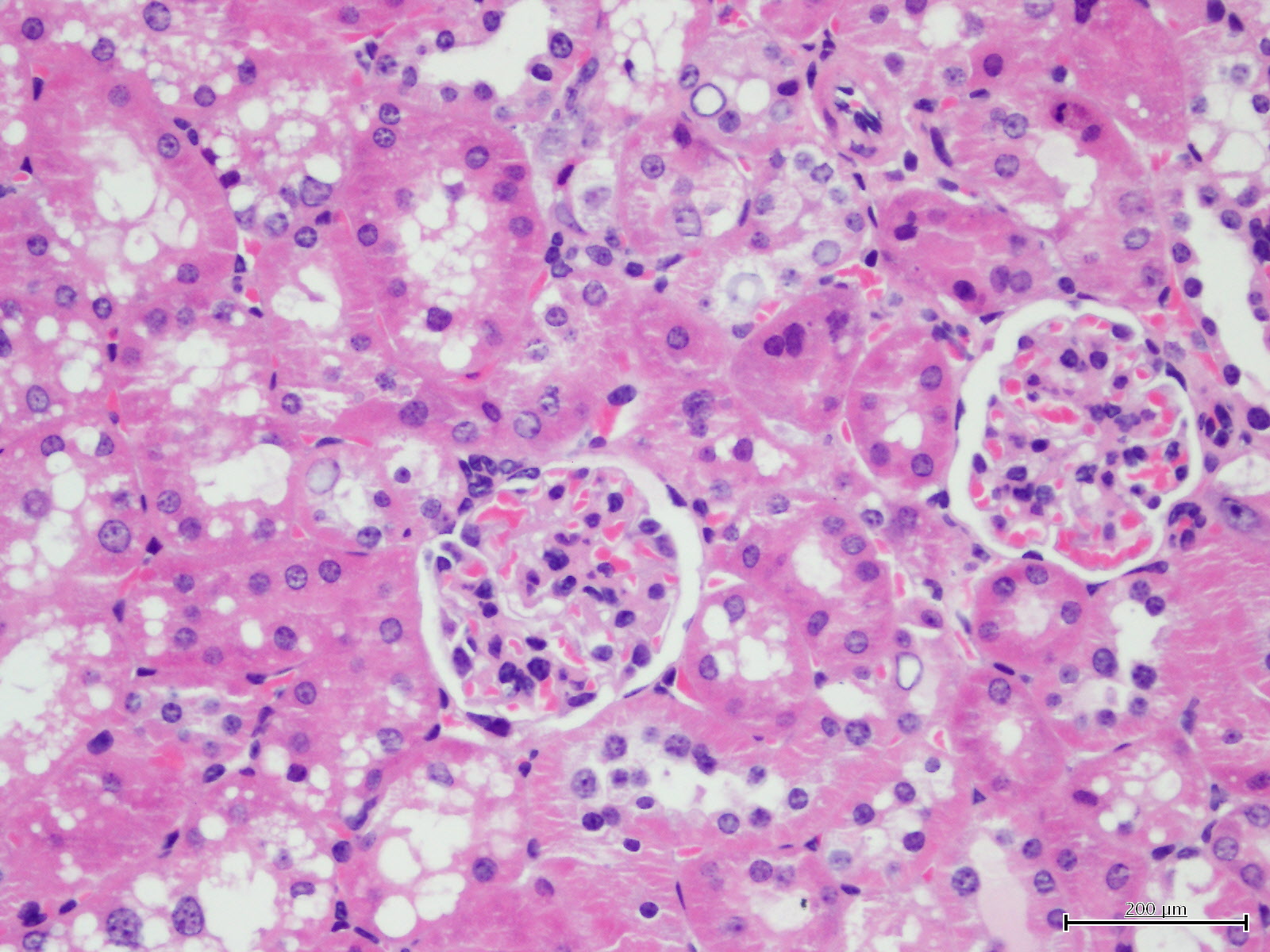

Supplement: S4 File — (ZIP) [file pone.0327042.s004.zip › HE-8w 75mGy DM/8w 75mGy DM-1.JPG]

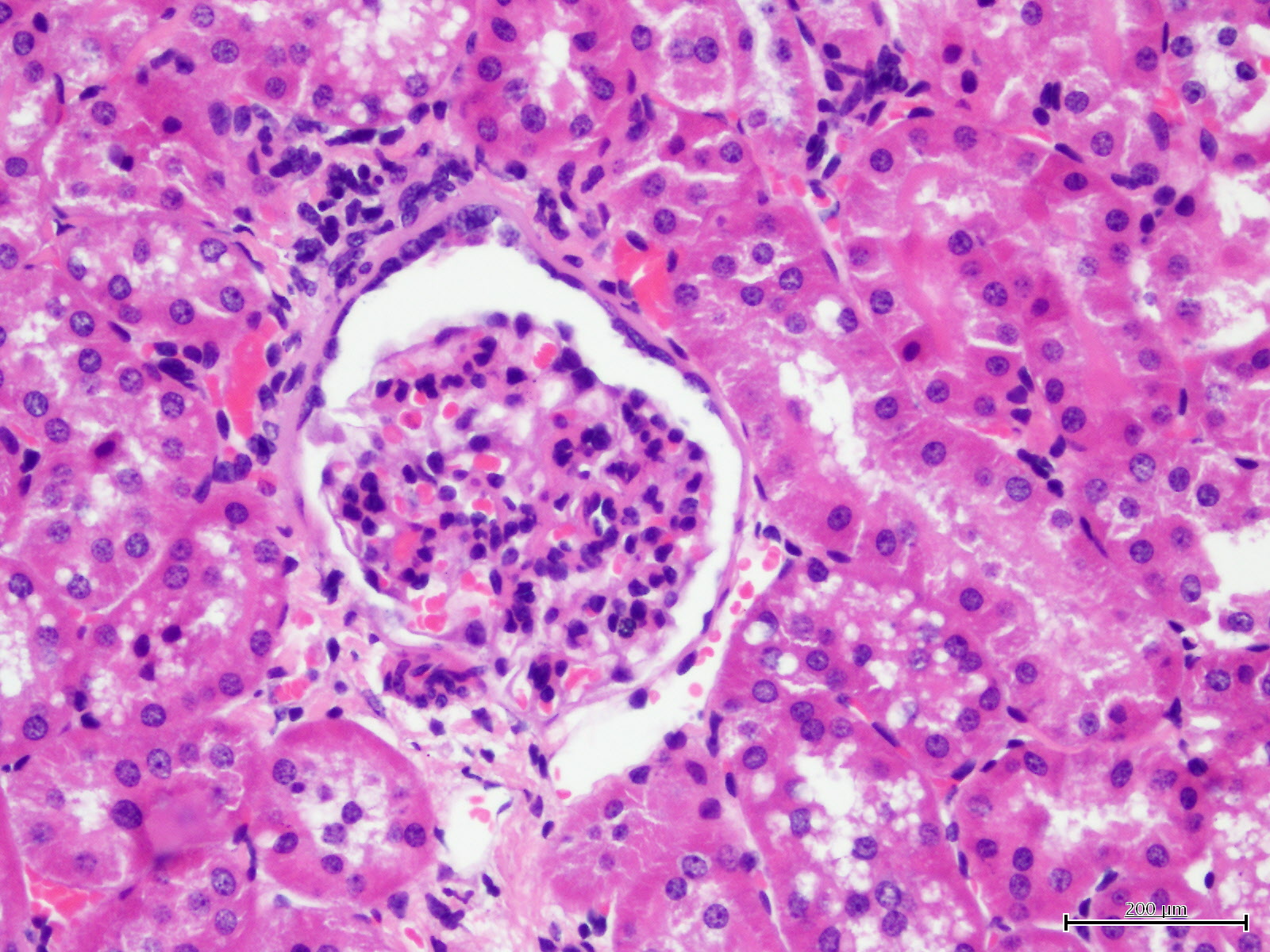

Supplement: S4 File — (ZIP) [file pone.0327042.s004.zip › HE-8w 75mGy DM/8w 75mGy DM-4 (Used publication).JPG]

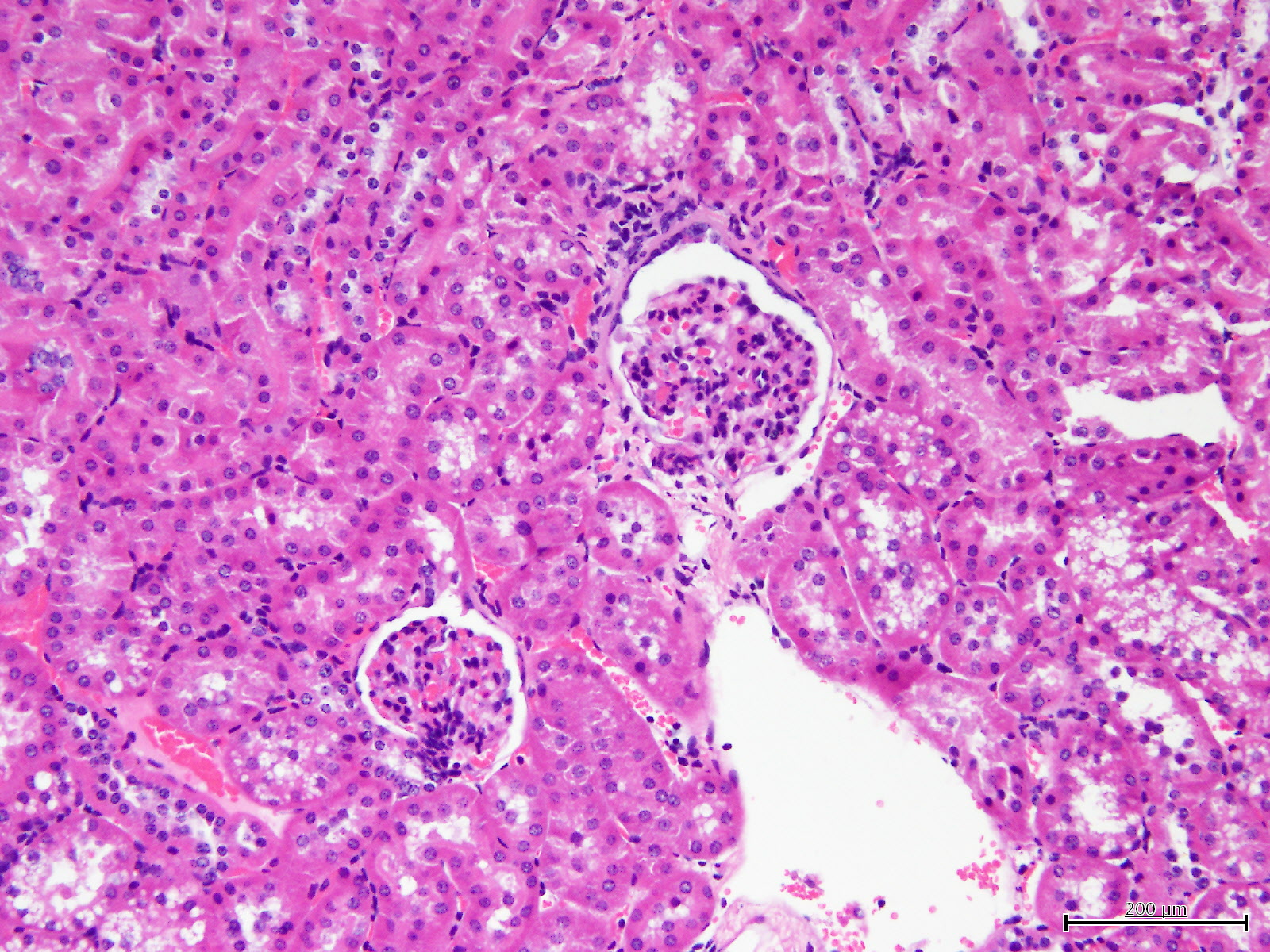

Supplement: S4 File — (ZIP) [file pone.0327042.s004.zip › HE-8w 75mGy DM/8w 75mGy DM-4.JPG]

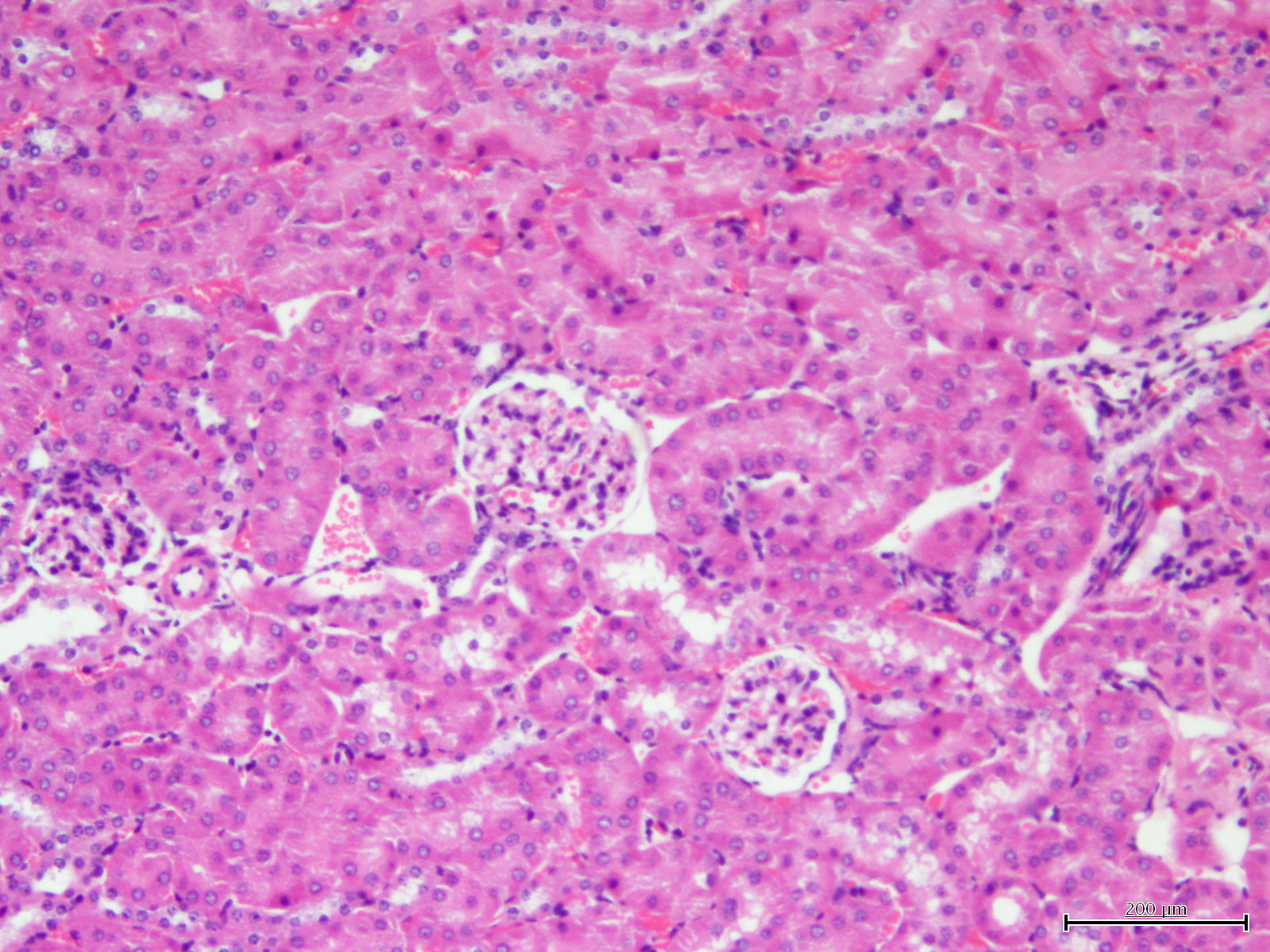

Supplement: S4 File — (ZIP) [file pone.0327042.s004.zip › HE-8w Con/8w Con-1 20X.JPG]

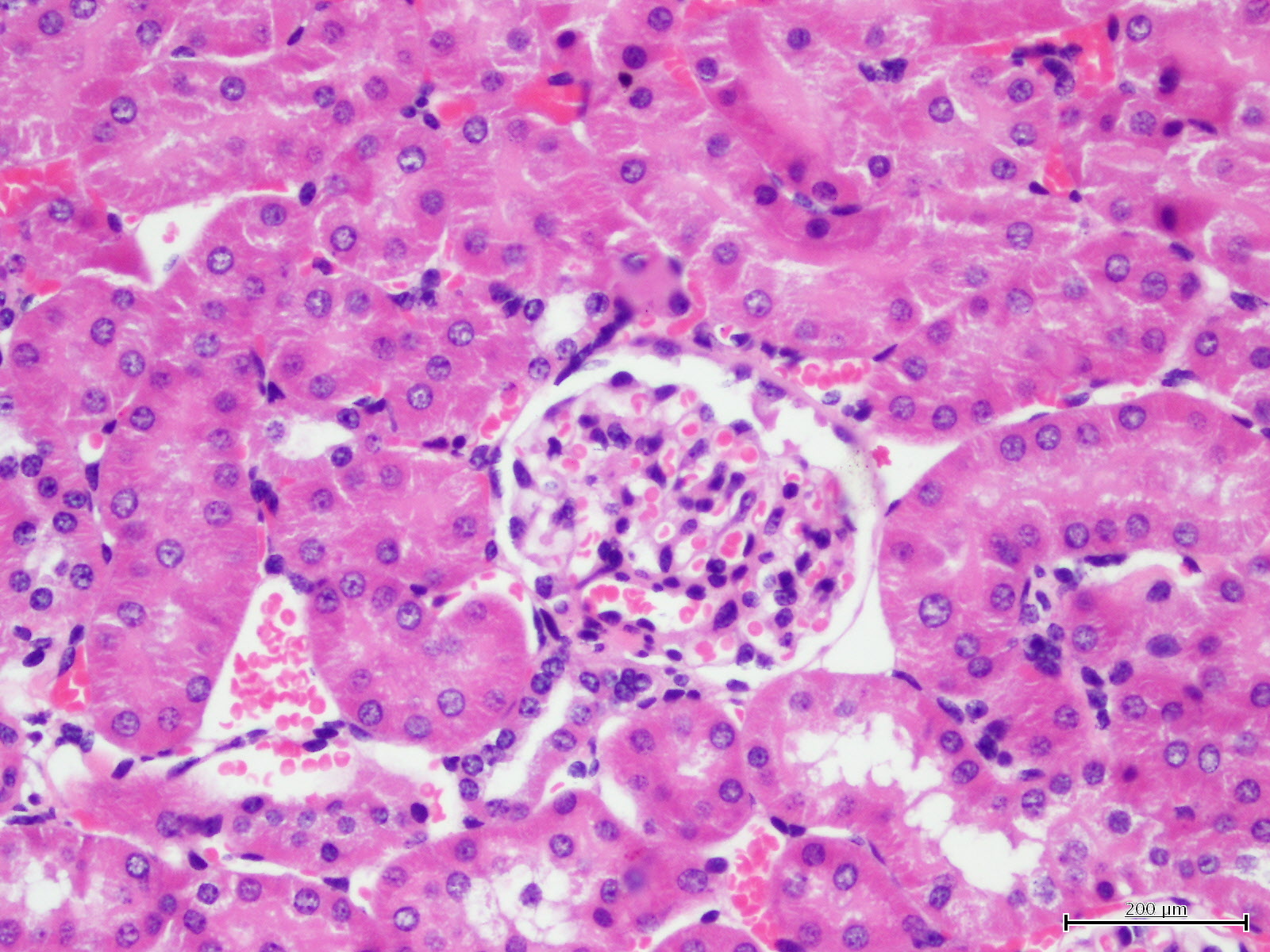

Supplement: S4 File — (ZIP) [file pone.0327042.s004.zip › HE-8w Con/8w Con-1.JPG]

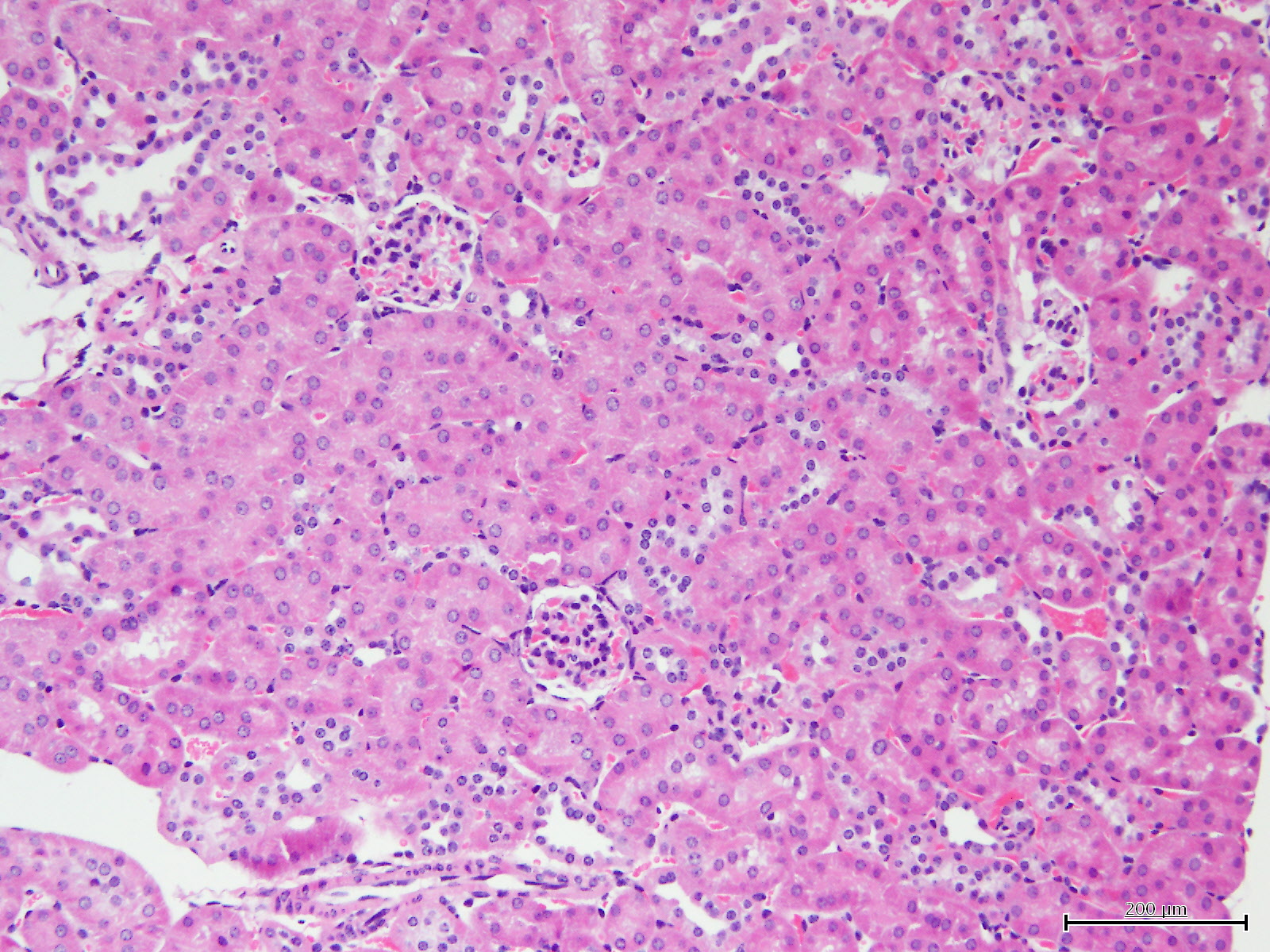

Supplement: S4 File — (ZIP) [file pone.0327042.s004.zip › HE-8w Con/8w Con-3.JPG]

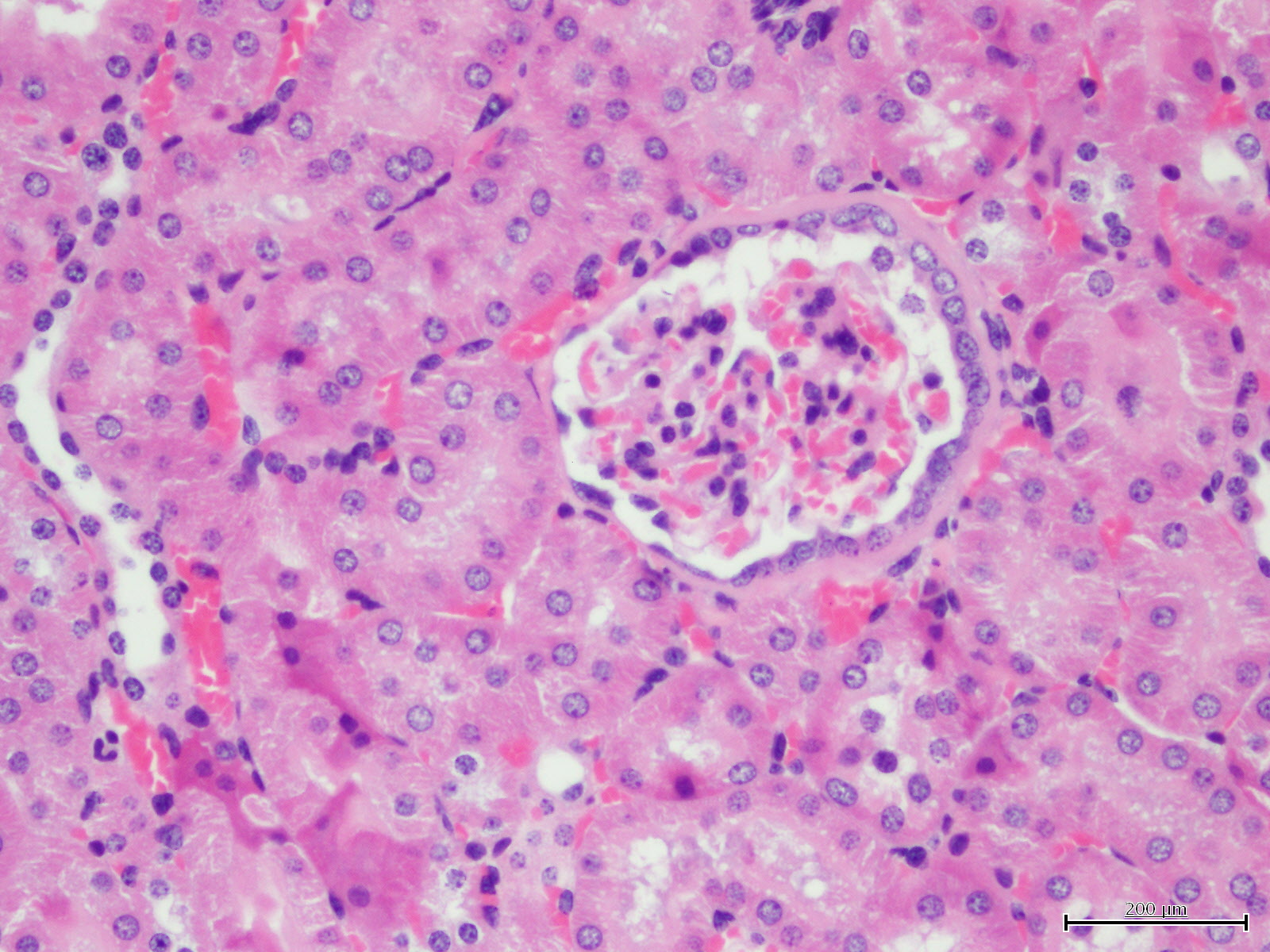

Supplement: S4 File — (ZIP) [file pone.0327042.s004.zip › HE-8w Con/8w Con-4 (Used publication).JPG]

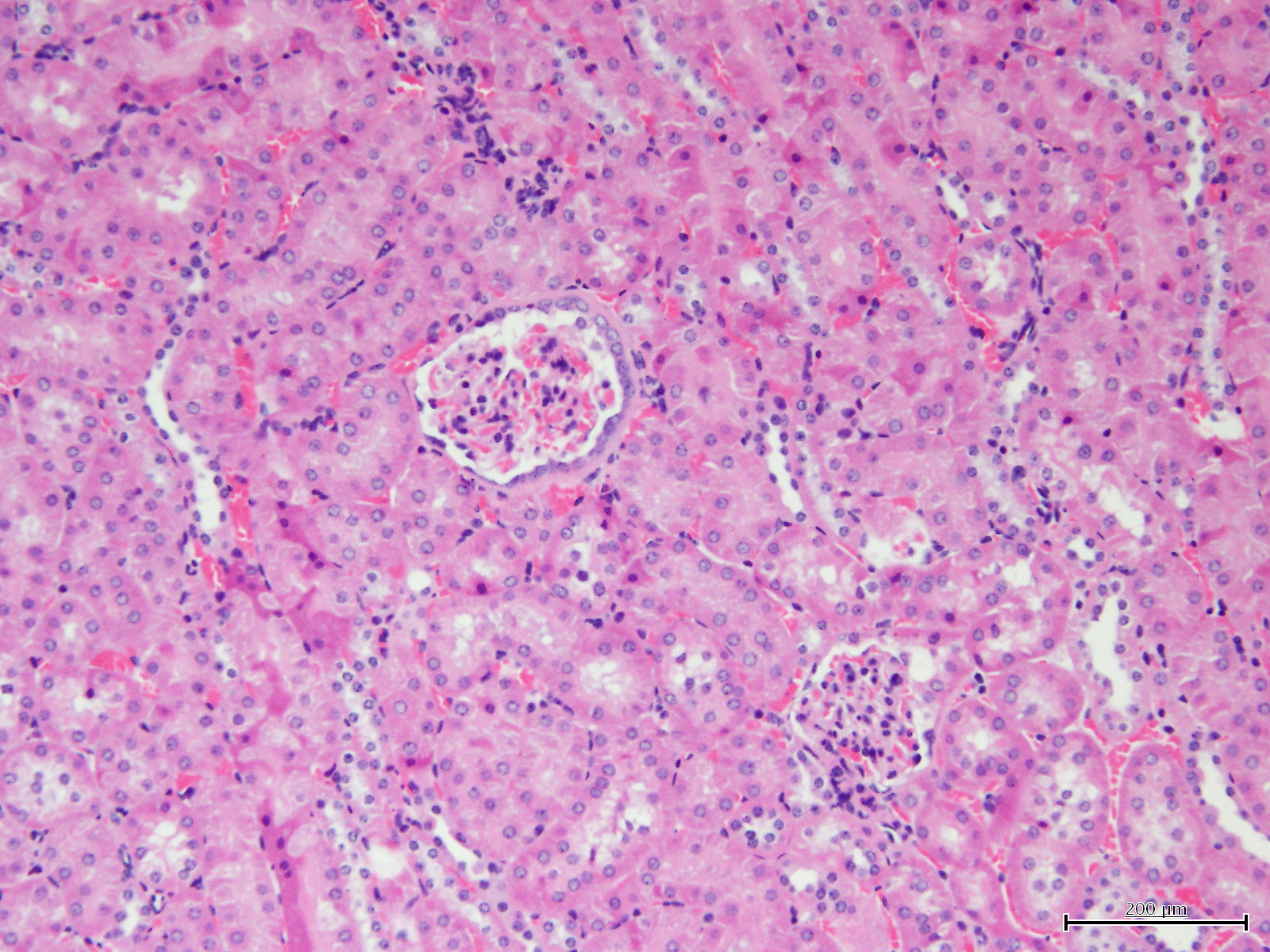

Supplement: S4 File — (ZIP) [file pone.0327042.s004.zip › HE-8w Con/8w Con-4 20X.JPG]

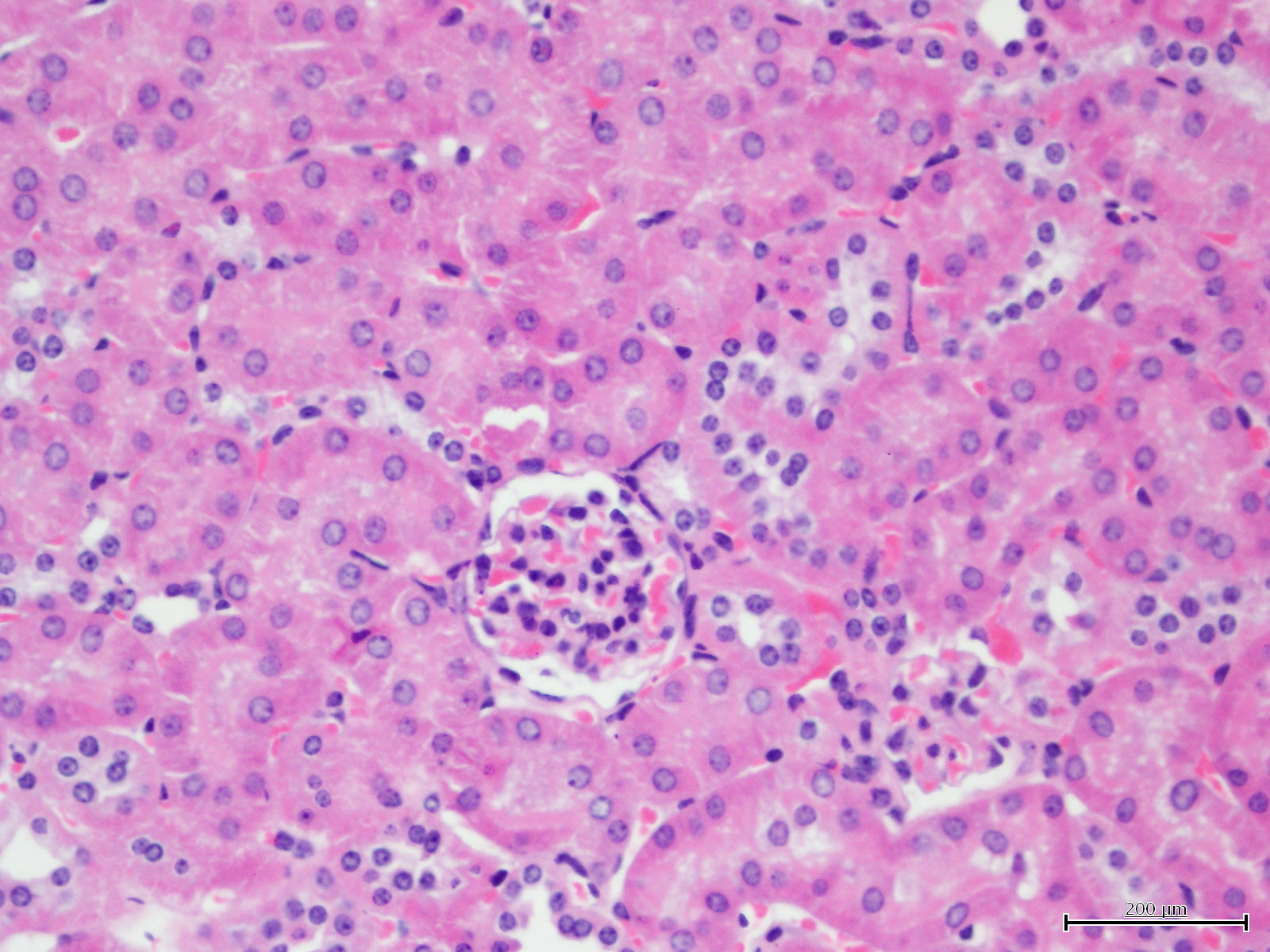

Supplement: S4 File — (ZIP) [file pone.0327042.s004.zip › HE-8w Con/8w Con-4.JPG]

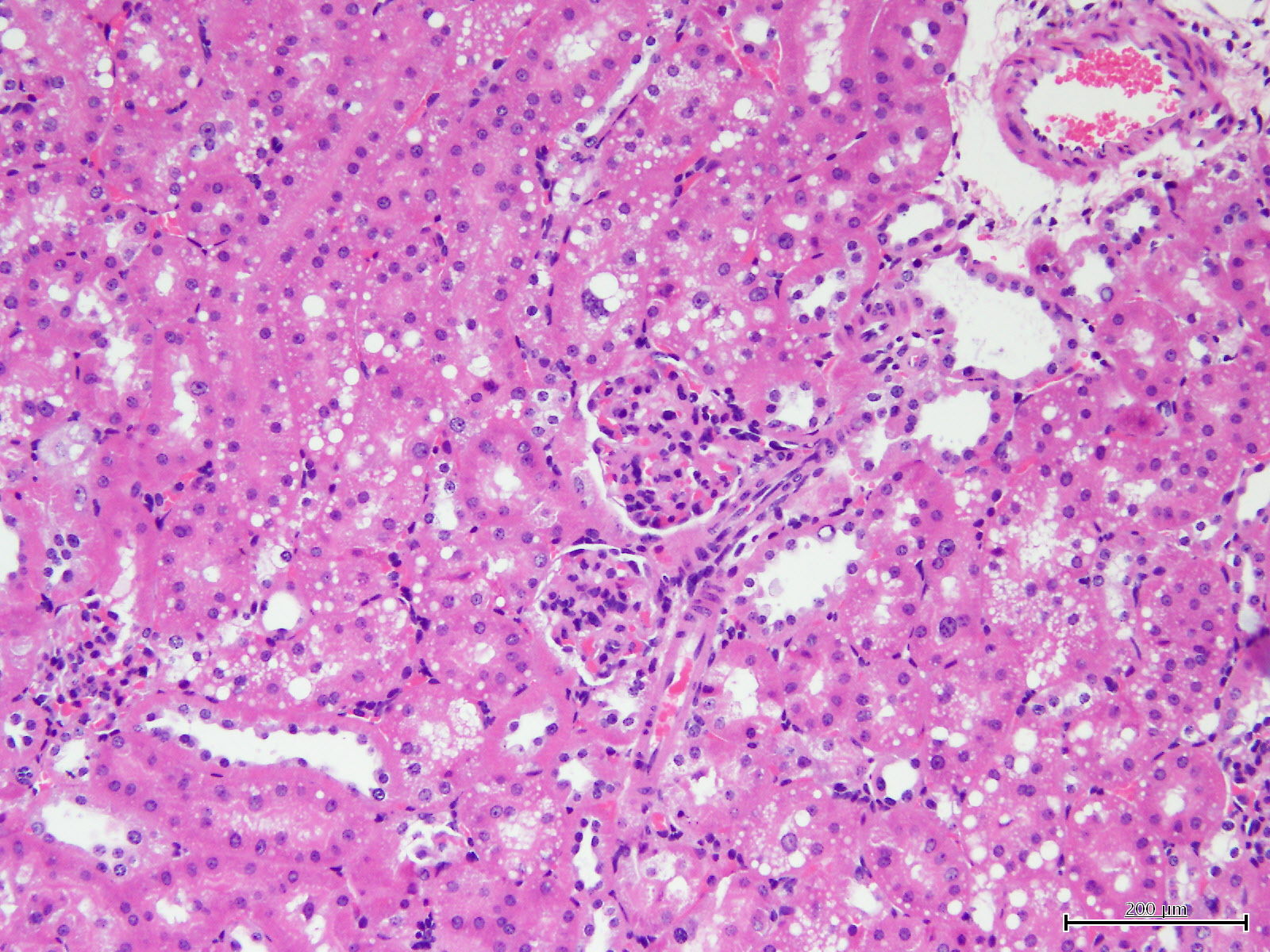

Supplement: S4 File — (ZIP) [file pone.0327042.s004.zip › HE-8w DM/HE-8w DM-1 20X.JPG]

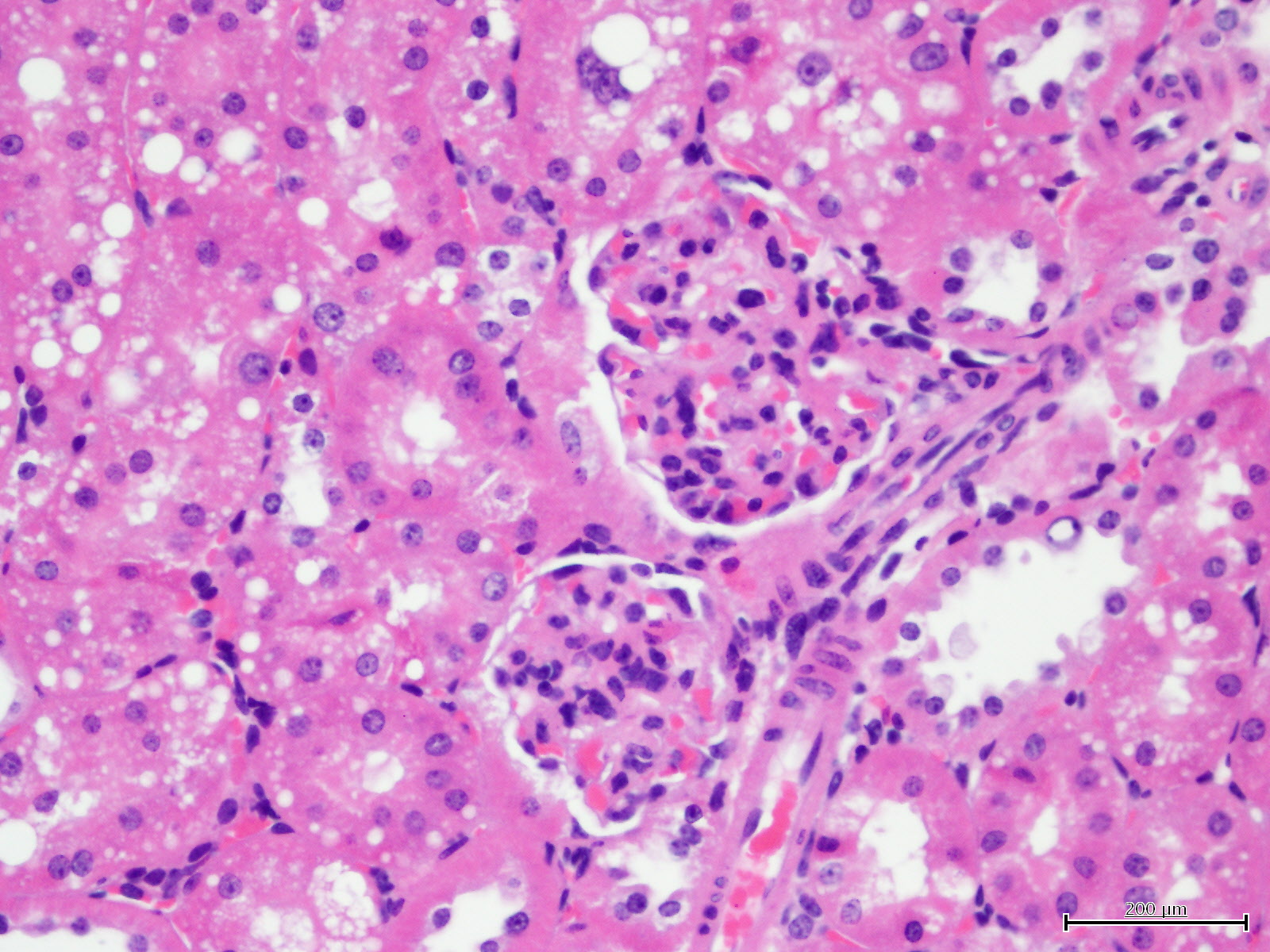

Supplement: S4 File — (ZIP) [file pone.0327042.s004.zip › HE-8w DM/HE-8w DM-1.JPG]

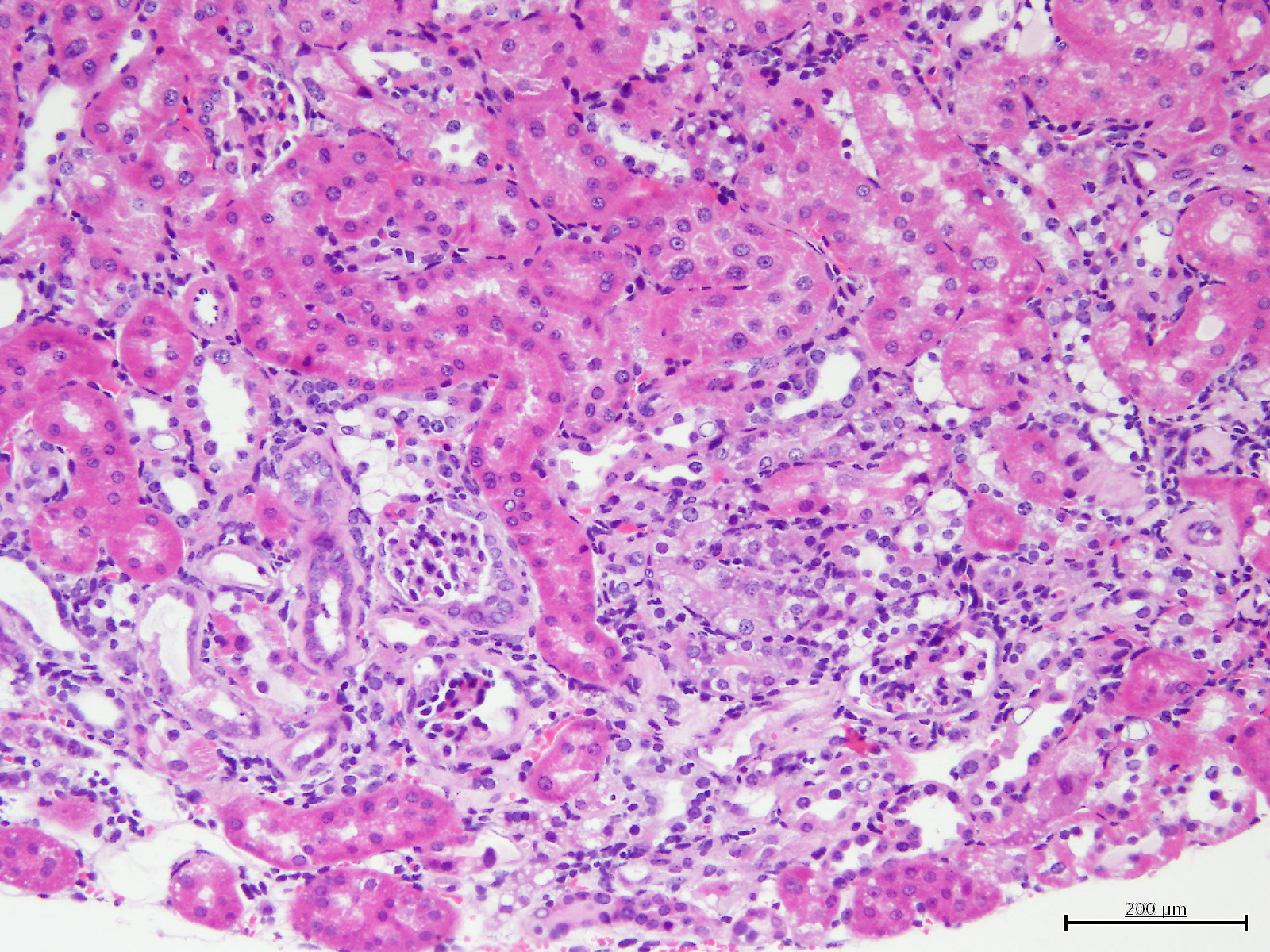

Supplement: S4 File — (ZIP) [file pone.0327042.s004.zip › HE-8w DM/HE-8w DM-2 20X.JPG]

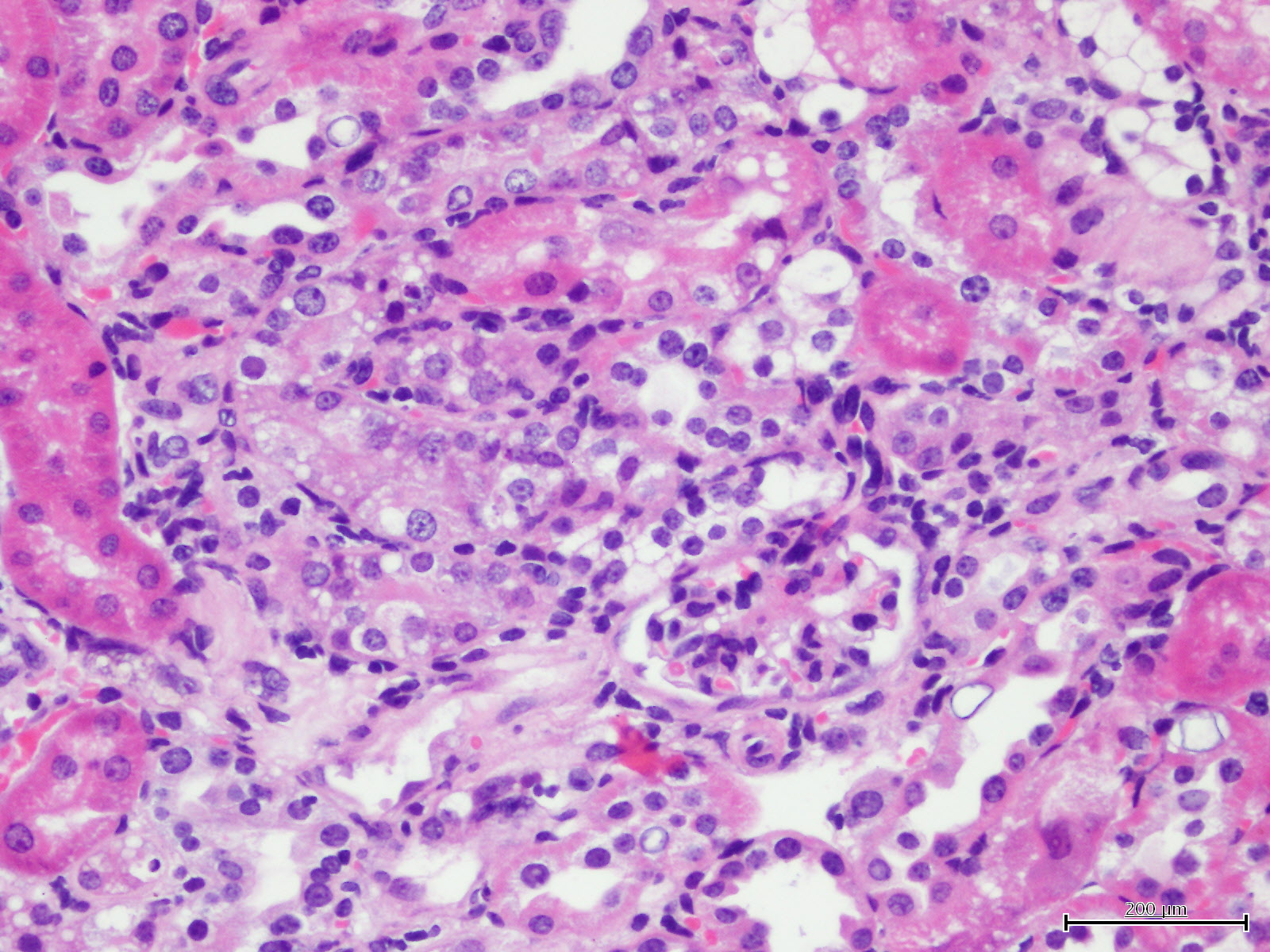

Supplement: S4 File — (ZIP) [file pone.0327042.s004.zip › HE-8w DM/HE-8w DM-2(Used publication).JPG]

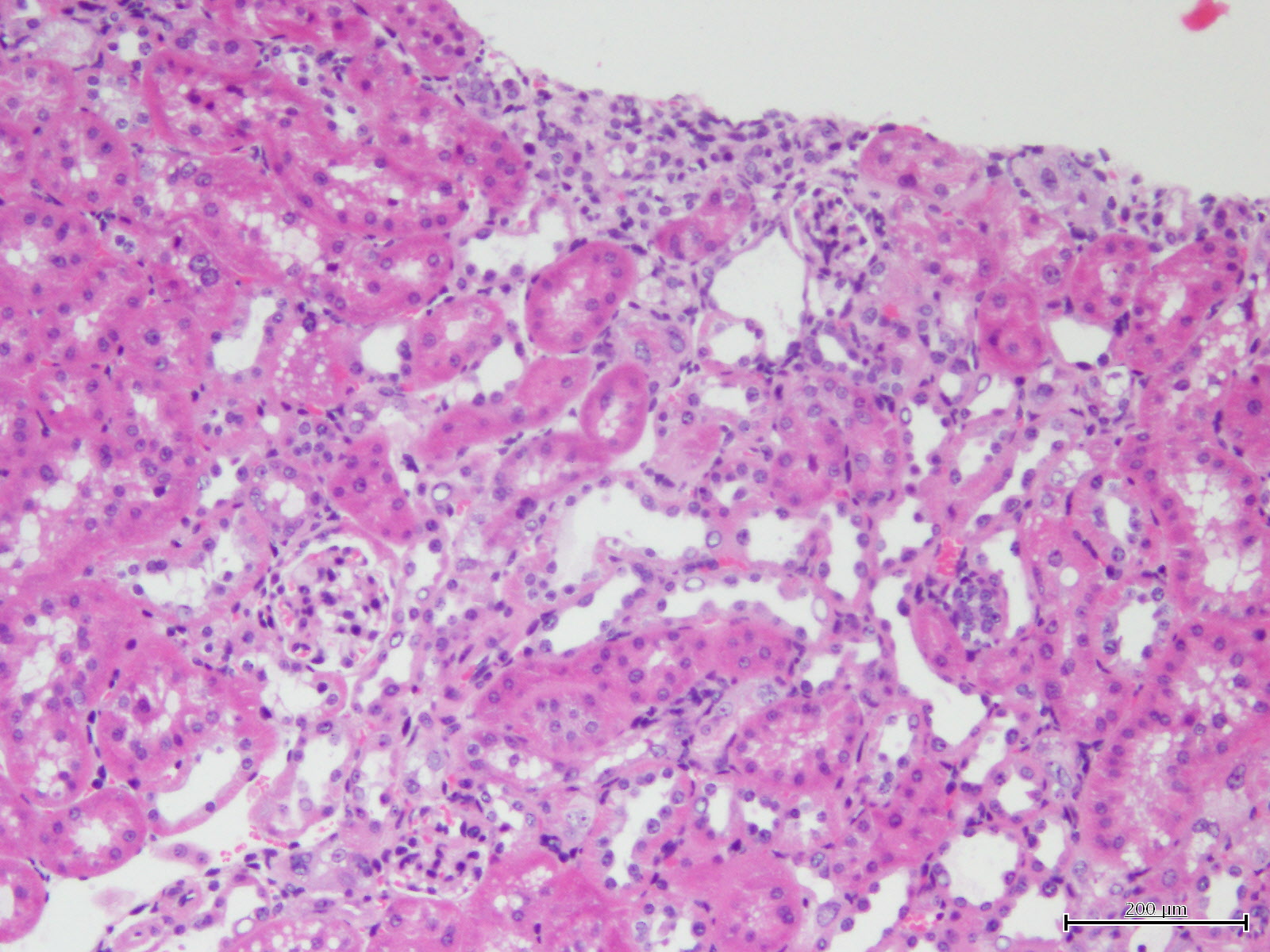

Supplement: S4 File — (ZIP) [file pone.0327042.s004.zip › HE-8w DM/HE-8w DM-3 20X.JPG]

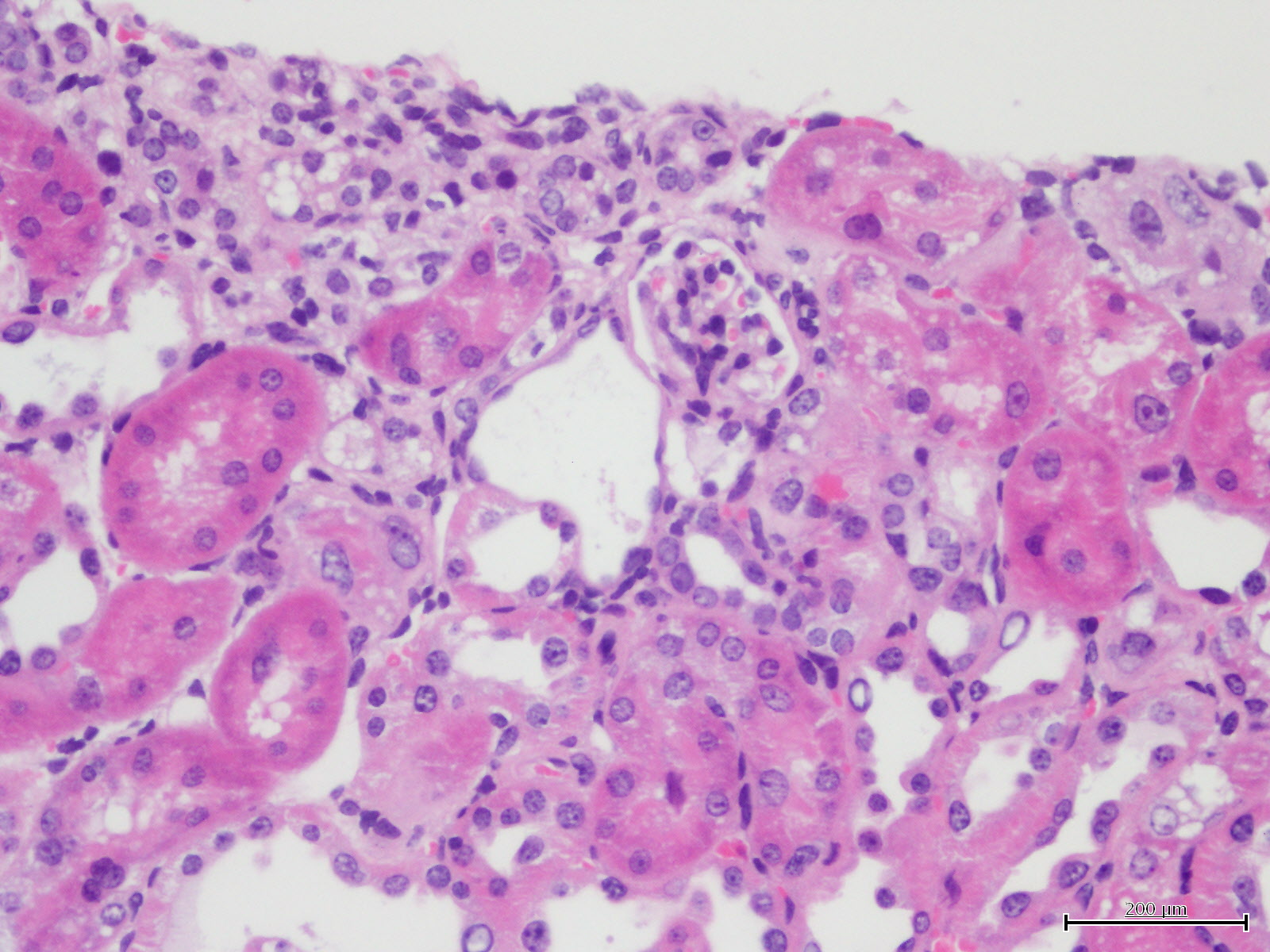

Supplement: S4 File — (ZIP) [file pone.0327042.s004.zip › HE-8w DM/HE-8w DM-3.JPG]

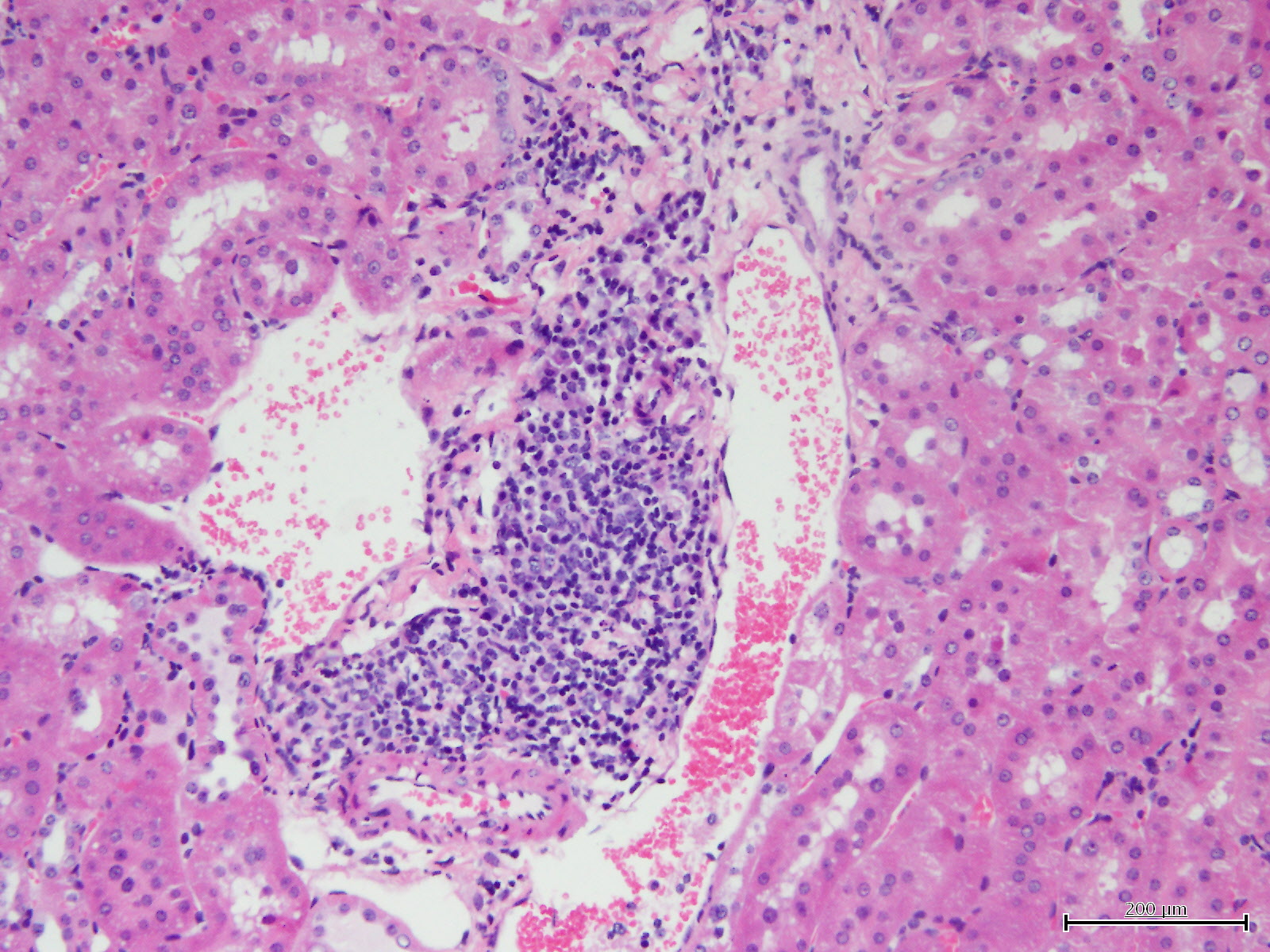

Supplement: S4 File — (ZIP) [file pone.0327042.s004.zip › HE-8w DM/HE-8w DM-4 20x.JPG]

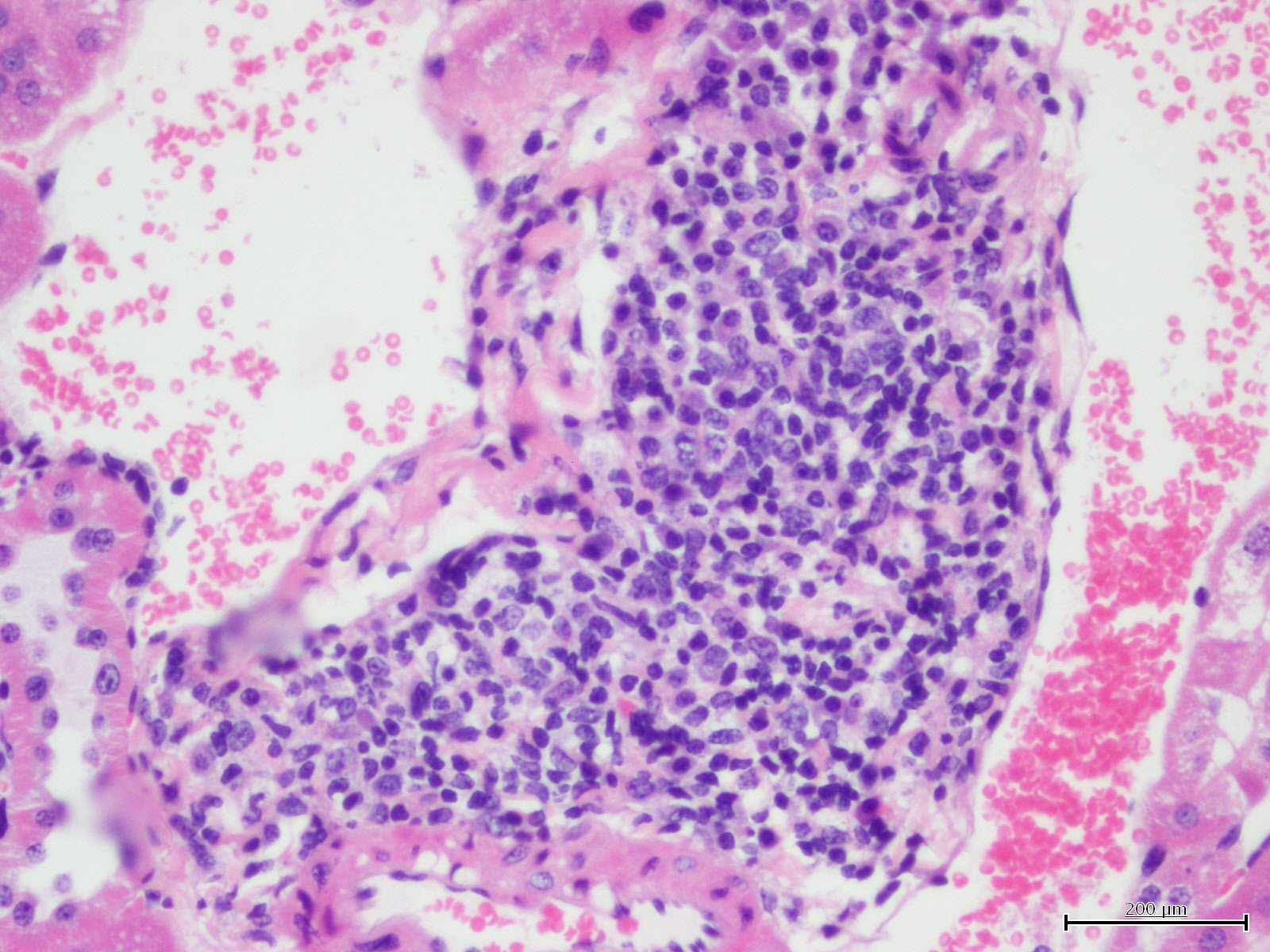

Supplement: S4 File — (ZIP) [file pone.0327042.s004.zip › HE-8w DM/HE-8w DM-4.JPG]

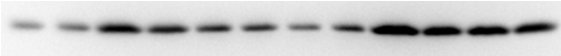

Supplement: S5 File — (ZIP) [file pone.0327042.s005.zip › Fig 3B_ICAM-1 blot.TIF]

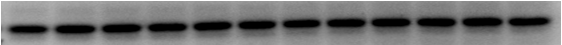

Supplement: S5 File — (ZIP) [file pone.0327042.s005.zip › actin ICAM1.TIF]

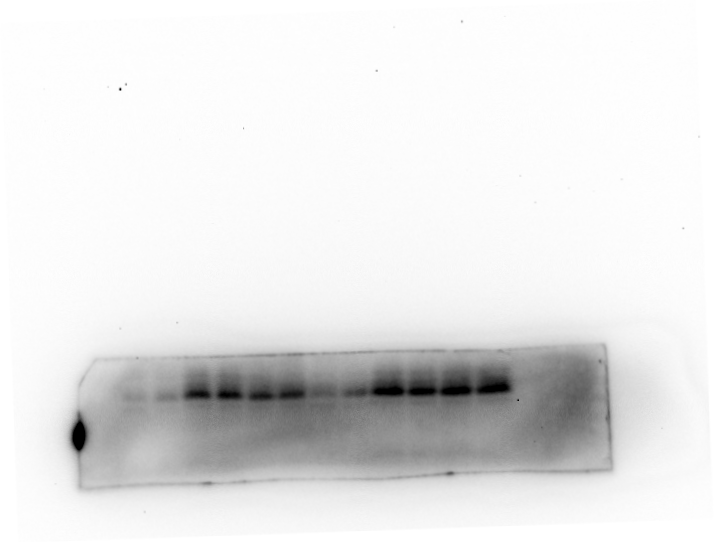

Supplement: S6 File — (ZIP) [file pone.0327042.s006.zip › Fig 4B_TNFα blot.tif]

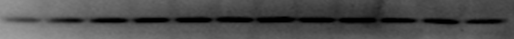

Supplement: S6 File — (ZIP) [file pone.0327042.s006.zip › actin TNF .tif]

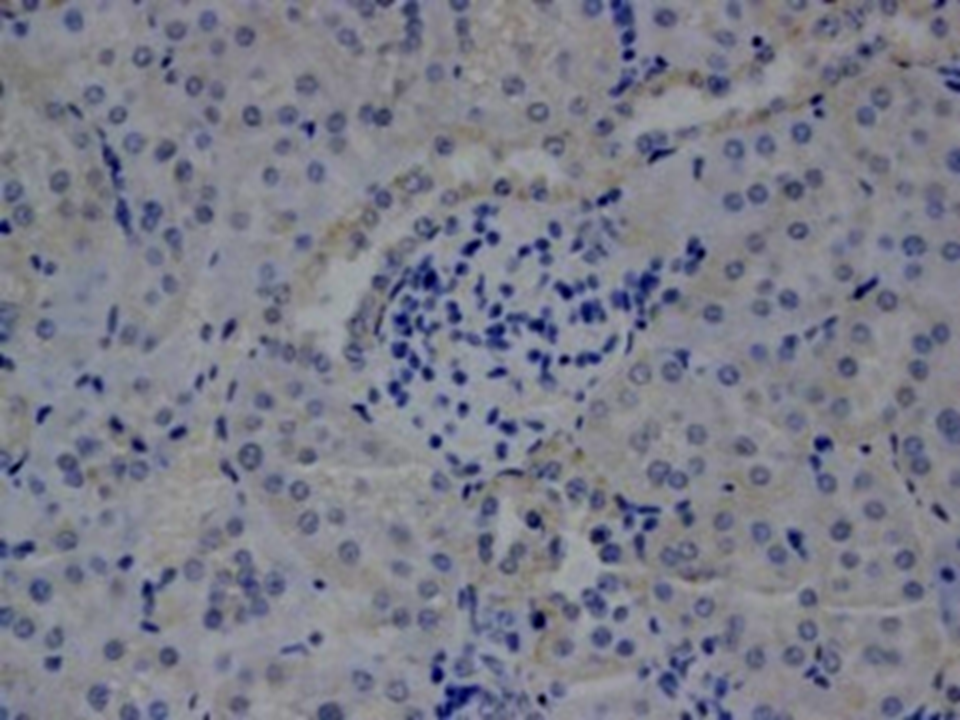

Supplement: S7 File — (ZIP) [file pone.0327042.s007.zip › TNFα IHC Original images/Con-1 4w (Used publication).tif]

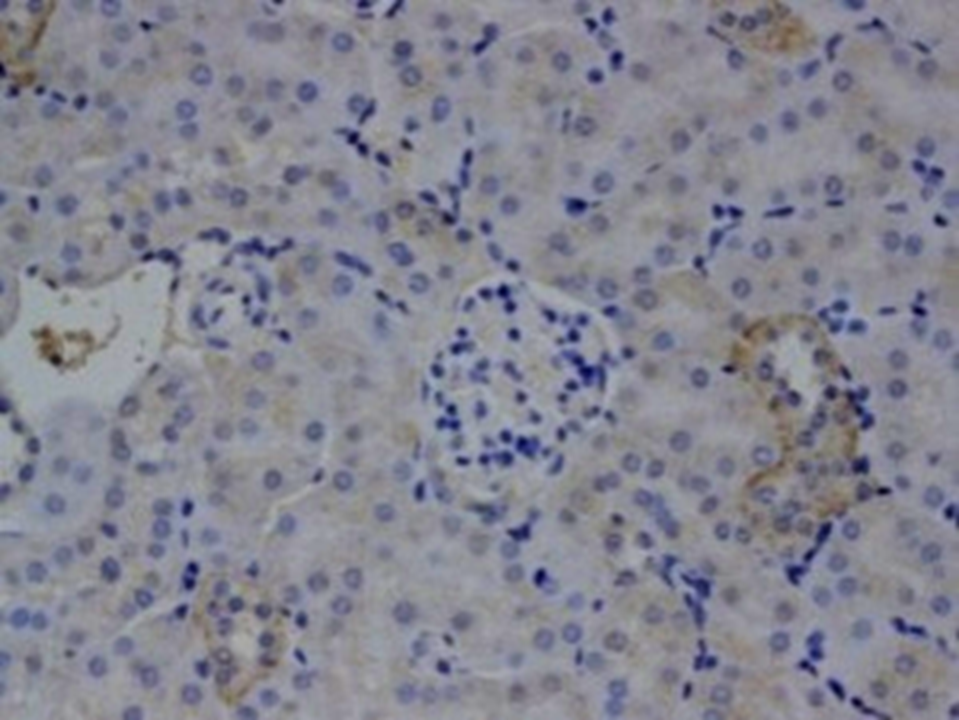

Supplement: S7 File — (ZIP) [file pone.0327042.s007.zip › TNFα IHC Original images/Con-1 50mGy 4w(Used publication).tif]

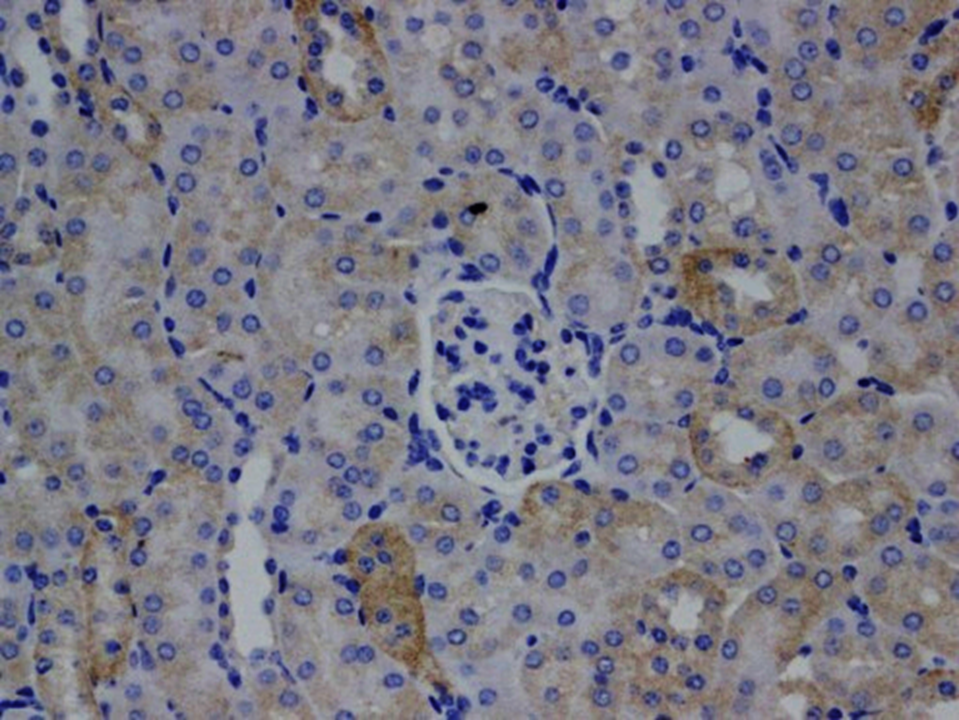

Supplement: S7 File — (ZIP) [file pone.0327042.s007.zip › TNFα IHC Original images/Con-1 50mGy 8w(Used publication).tif]

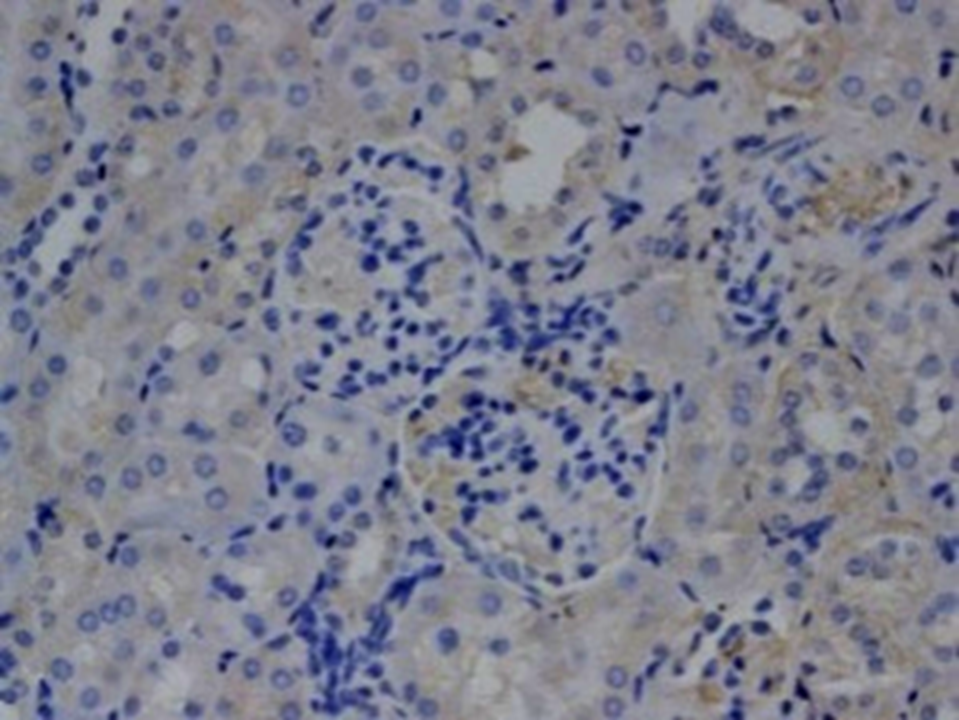

Supplement: S7 File — (ZIP) [file pone.0327042.s007.zip › TNFα IHC Original images/Con-1 8w(Used publication).tif]

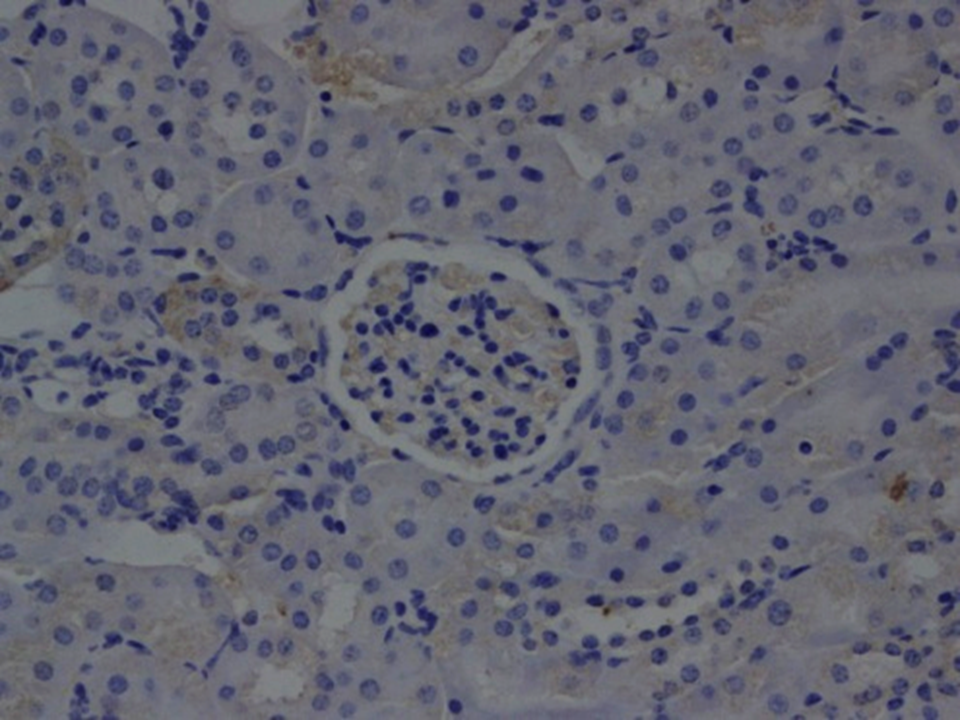

Supplement: S7 File — (ZIP) [file pone.0327042.s007.zip › TNFα IHC Original images/Con-2 4w.tif]

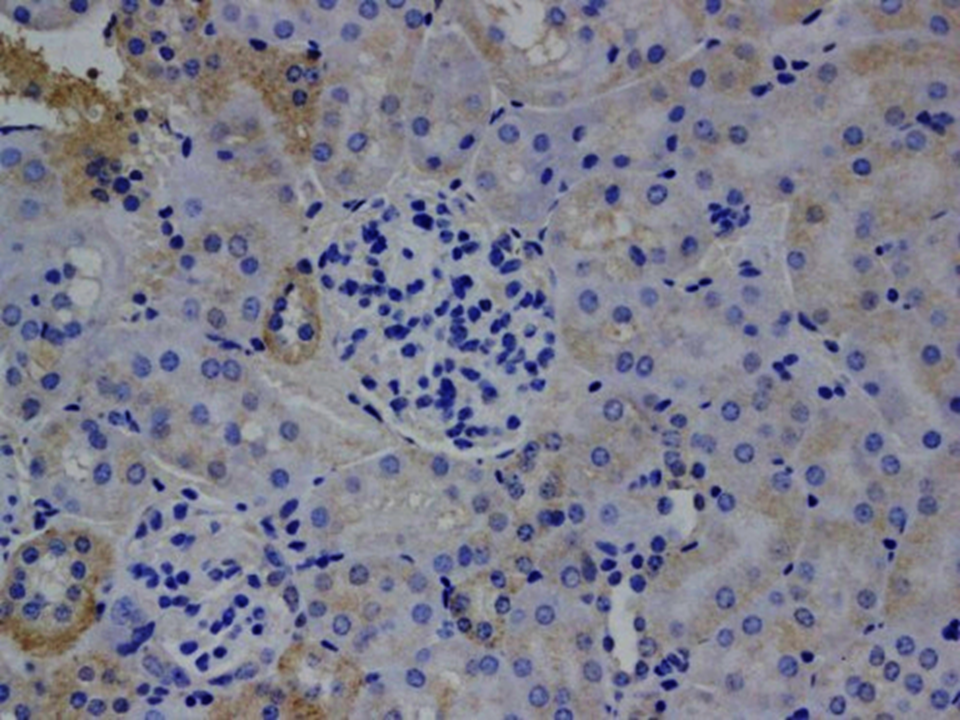

Supplement: S7 File — (ZIP) [file pone.0327042.s007.zip › TNFα IHC Original images/Con-2 8w.tif]

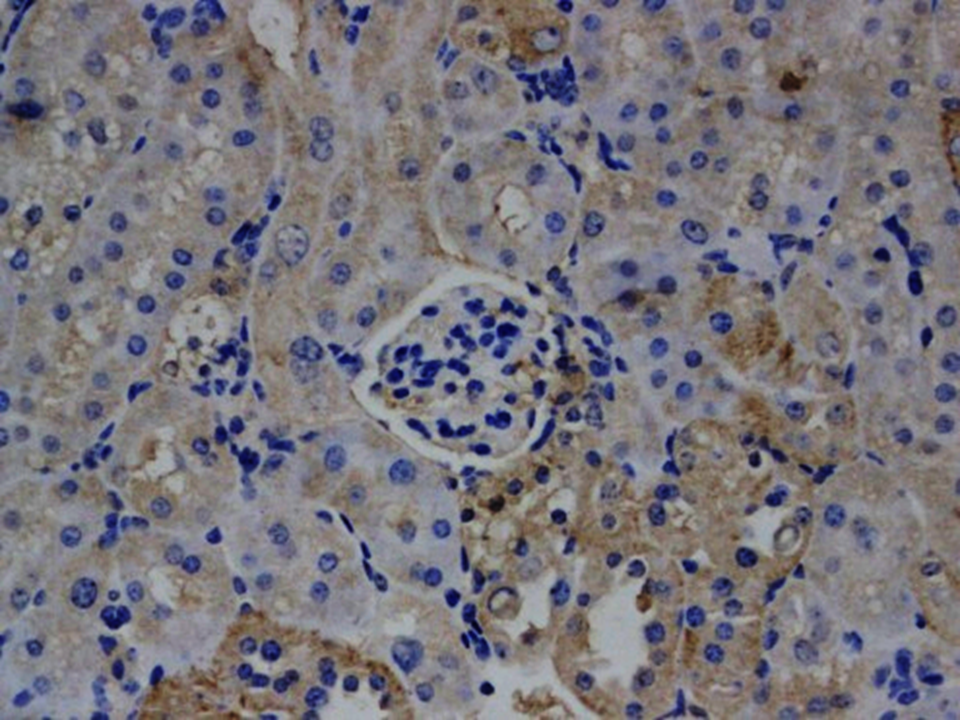

Supplement: S7 File — (ZIP) [file pone.0327042.s007.zip › TNFα IHC Original images/DM 25mGy 4w-2.tif]

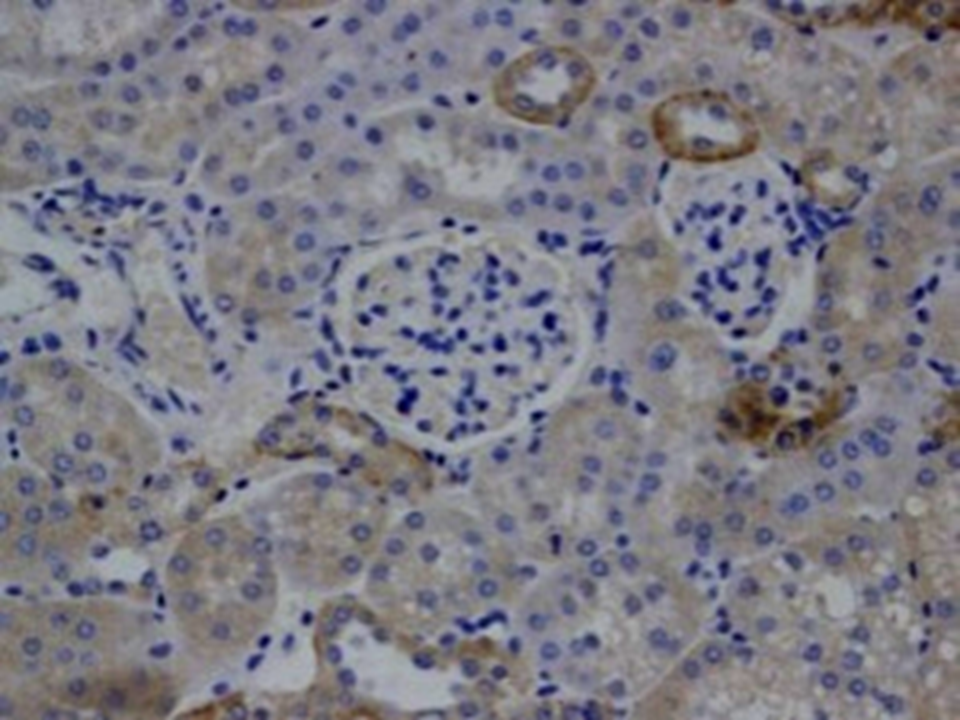

Supplement: S7 File — (ZIP) [file pone.0327042.s007.zip › TNFα IHC Original images/DM 25mGy 8w-1(Used publication).tif]

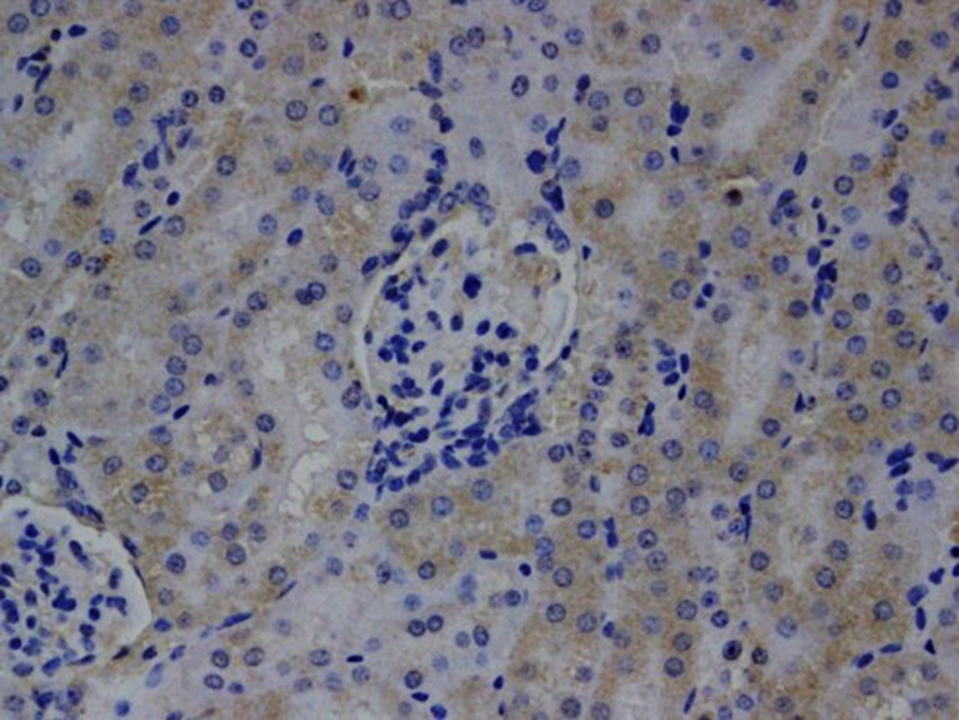

Supplement: S7 File — (ZIP) [file pone.0327042.s007.zip › TNFα IHC Original images/DM 25mGy 8w-2.tif]

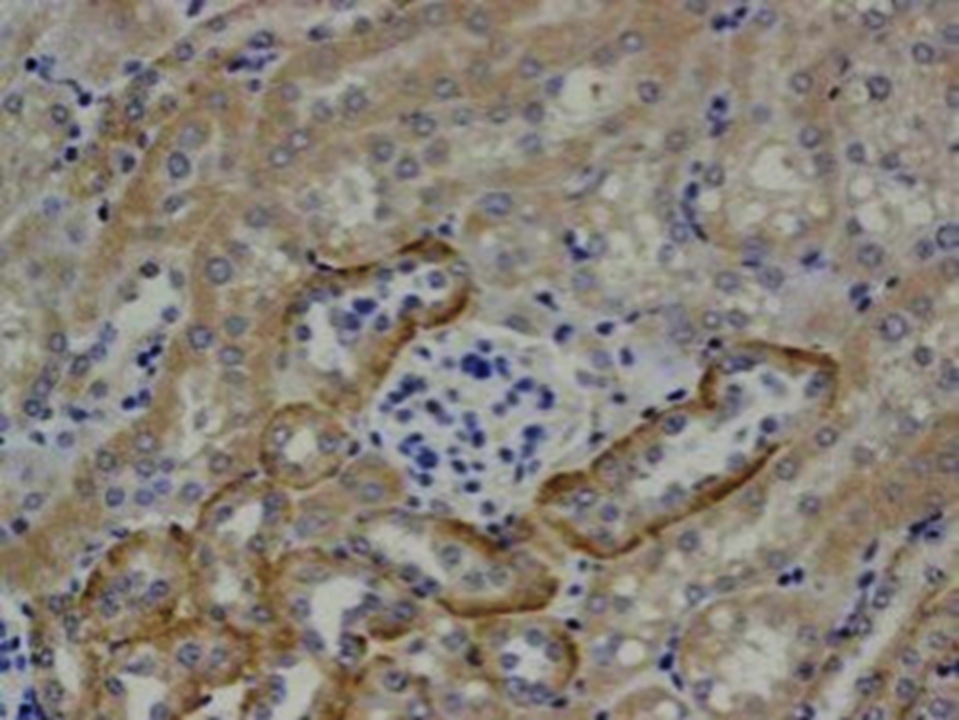

Supplement: S7 File — (ZIP) [file pone.0327042.s007.zip › TNFα IHC Original images/DM 4w -1(Used publication).tif]

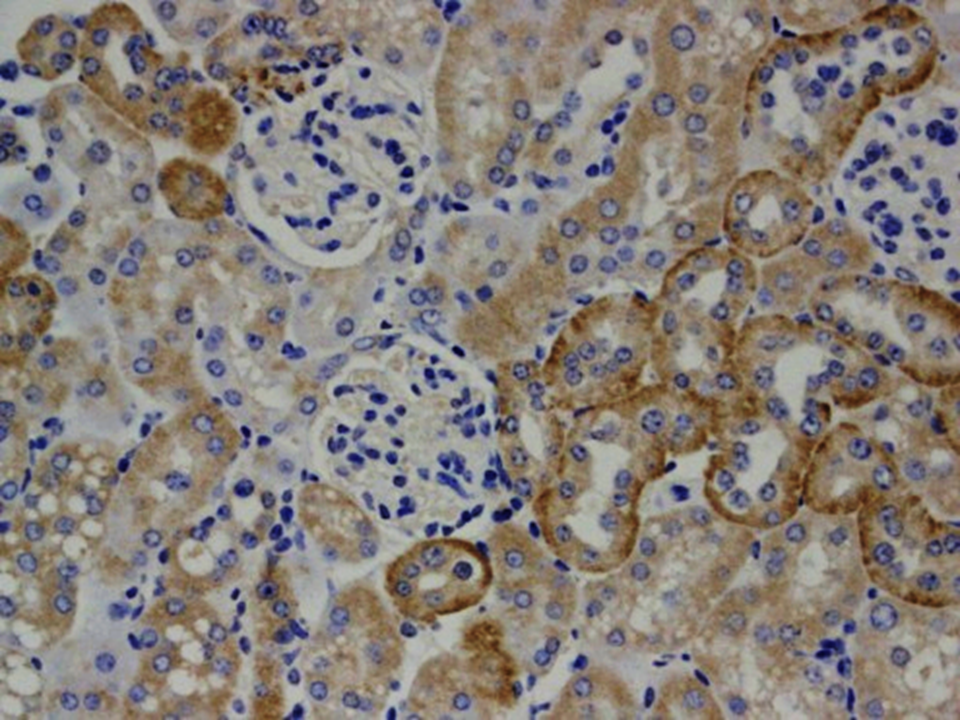

Supplement: S7 File — (ZIP) [file pone.0327042.s007.zip › TNFα IHC Original images/DM 4w -1_2(Incorrectly used for DM 4w 25mGy group).tif]
