# Supplementary figures and images for: Expression of Concern: Multiple Low-Dose Radiation Prevents Type 2 Diabetes-Induced Renal Damage through Attenuation of Dyslipidemia and Insulin Resistance and Subsequent Renal Inflammation and Oxidative Stress (part 2 of 3)
Source: PLoS One. 2025 Jun 30;20(6):e0327042. doi: 10.1371/journal.pone.0327042 (PMC12208481; doi:10.1371/journal.pone.0327042)

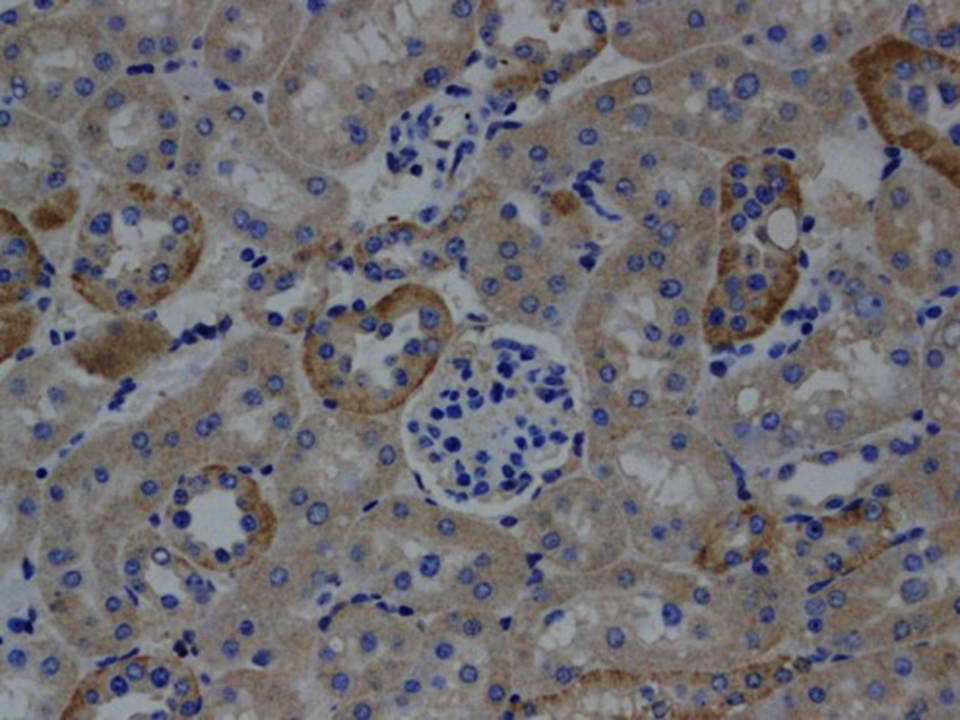

Supplement: S7 File — (ZIP) [file pone.0327042.s007.zip › TNFα IHC Original images/DM 4w 25mGy-1(Used publication).tif]

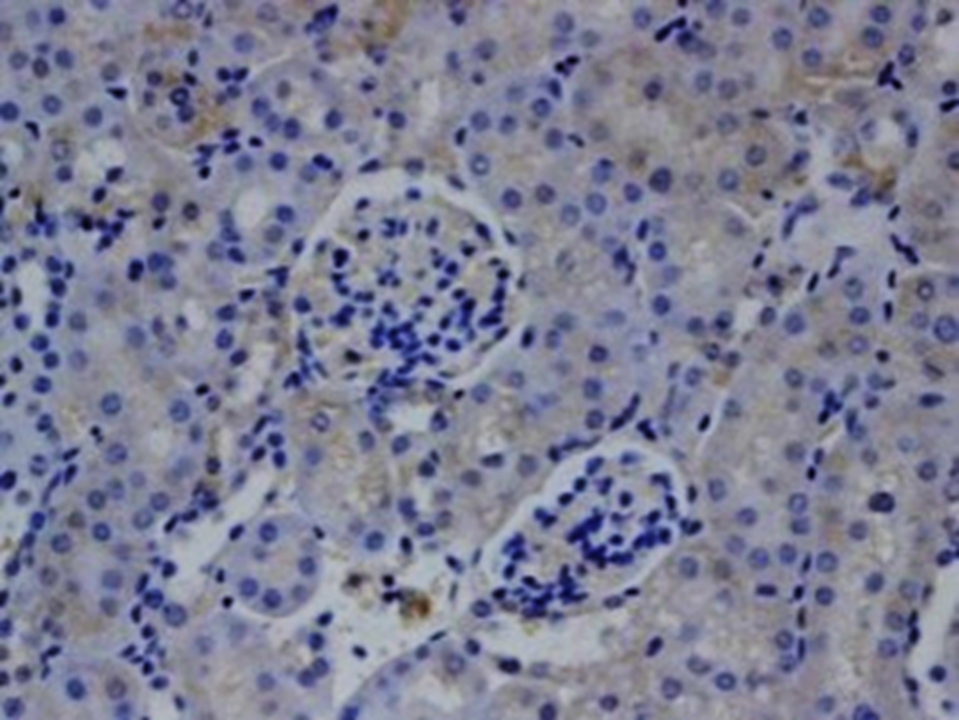

Supplement: S7 File — (ZIP) [file pone.0327042.s007.zip › TNFα IHC Original images/DM 50mGy 4w-1(Used publication).tif]

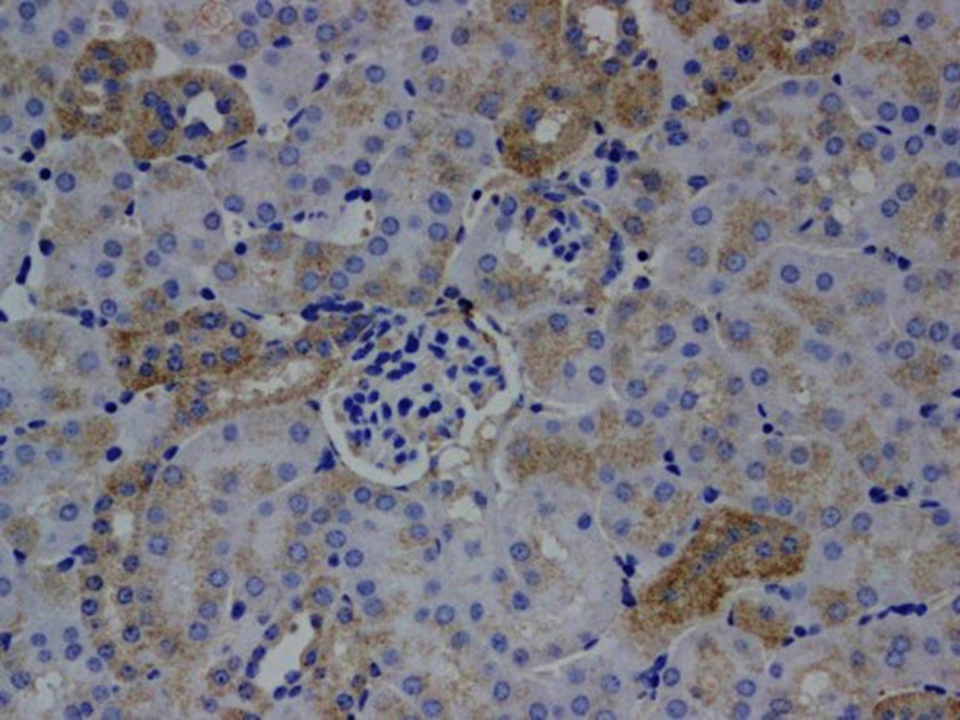

Supplement: S7 File — (ZIP) [file pone.0327042.s007.zip › TNFα IHC Original images/DM 50mGy 4w-2.tif]

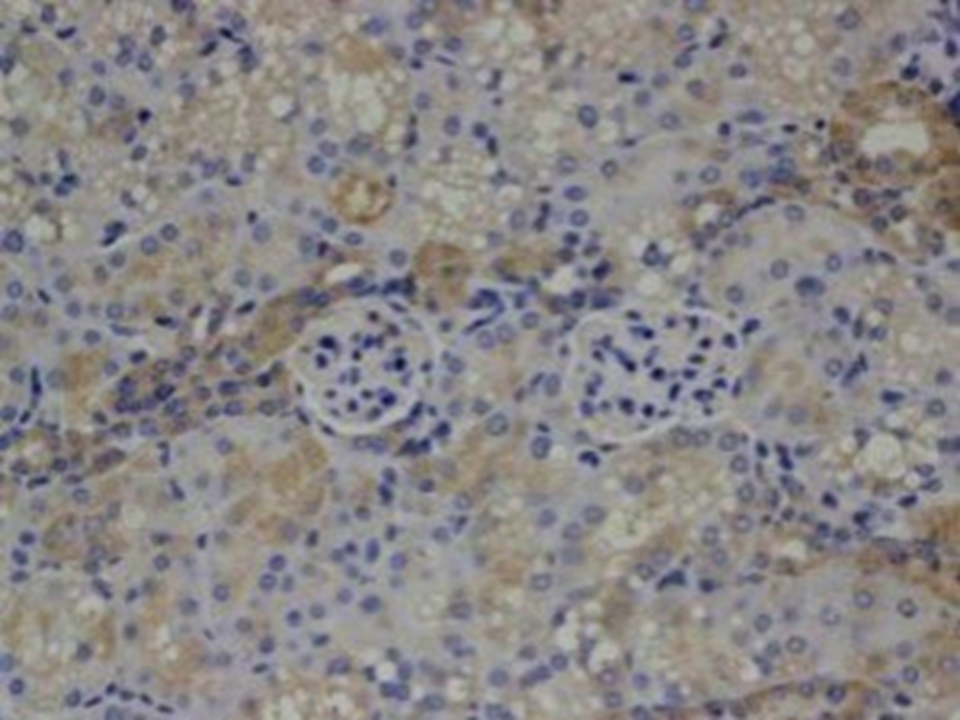

Supplement: S7 File — (ZIP) [file pone.0327042.s007.zip › TNFα IHC Original images/DM 50mGy 8w-1(Used publication).tif]

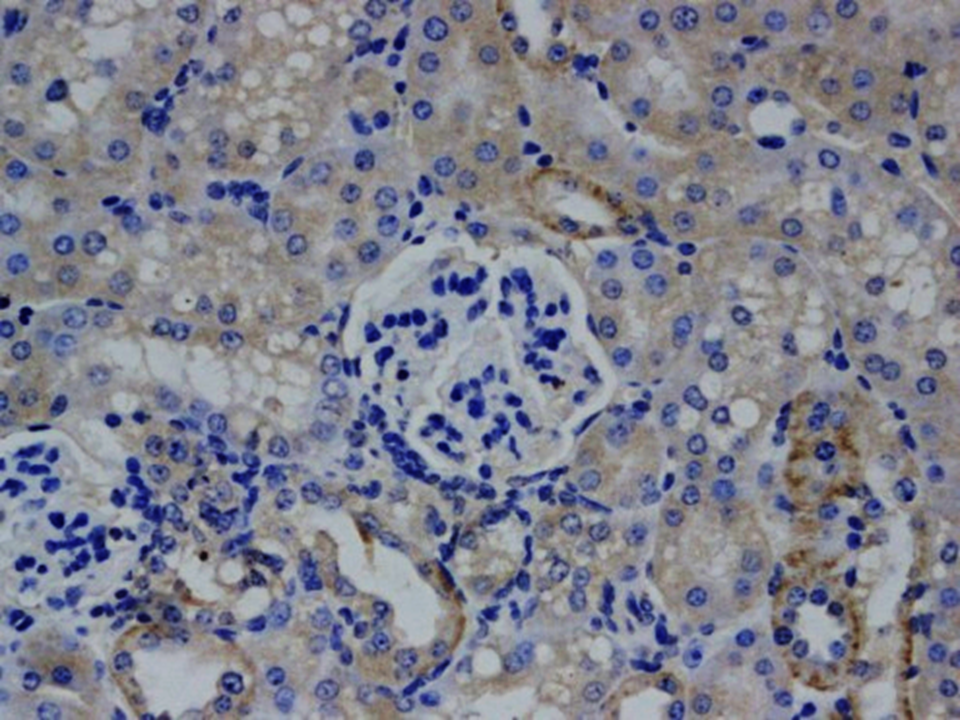

Supplement: S7 File — (ZIP) [file pone.0327042.s007.zip › TNFα IHC Original images/DM 50mGy 8w-2.tif]

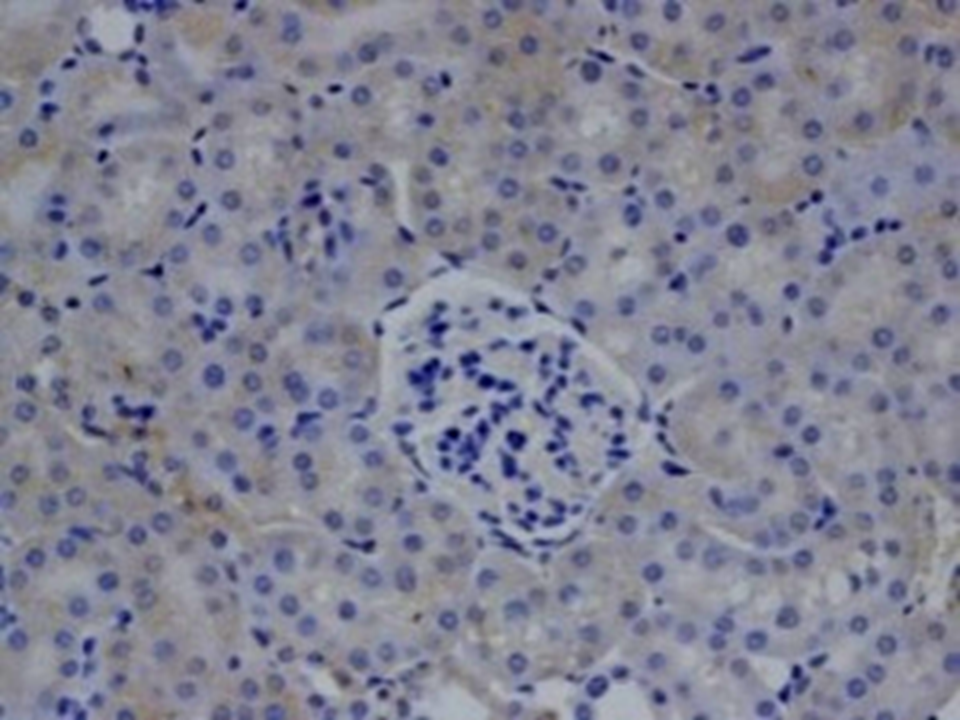

Supplement: S7 File — (ZIP) [file pone.0327042.s007.zip › TNFα IHC Original images/DM 75mGy 4w-1(Used publication).tif]

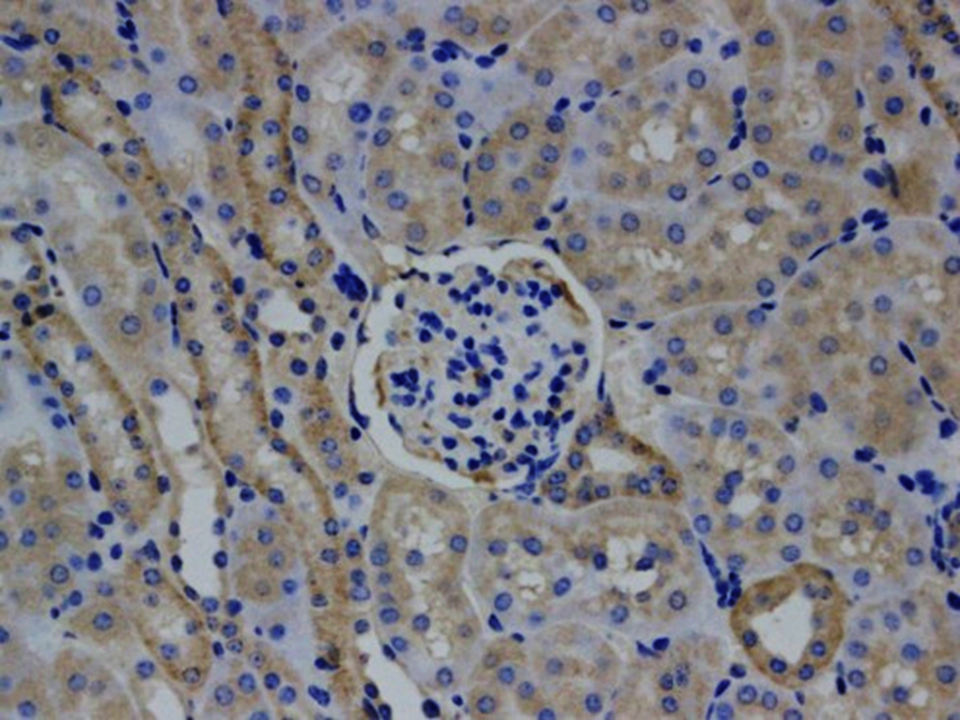

Supplement: S7 File — (ZIP) [file pone.0327042.s007.zip › TNFα IHC Original images/DM 75mGy 4w-2.tif]

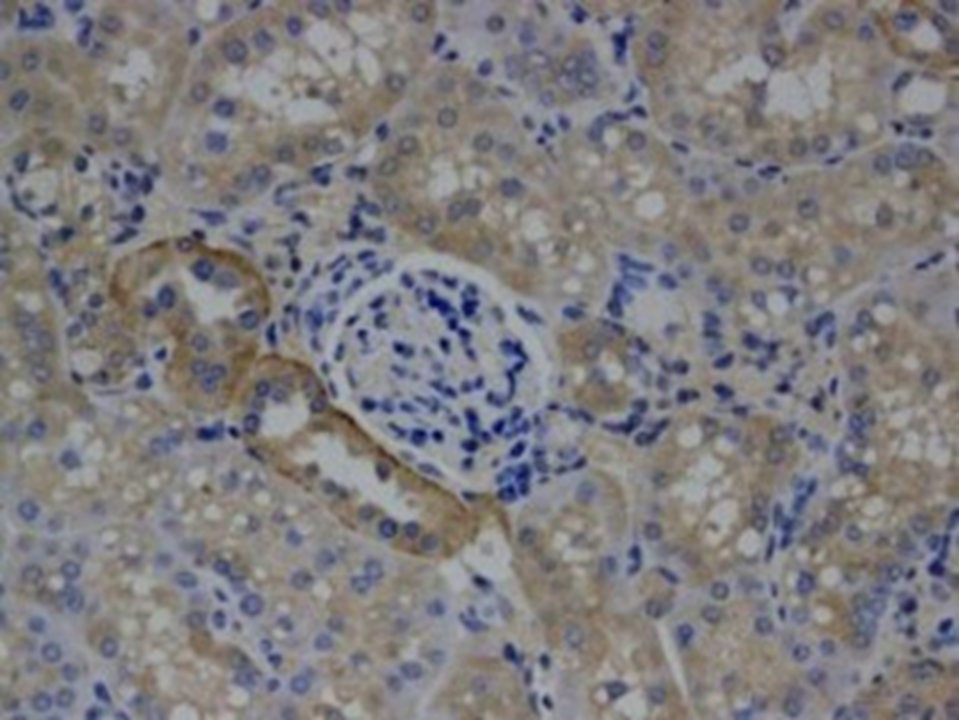

Supplement: S7 File — (ZIP) [file pone.0327042.s007.zip › TNFα IHC Original images/DM 75mGy 8w-1(Used publication).tif]

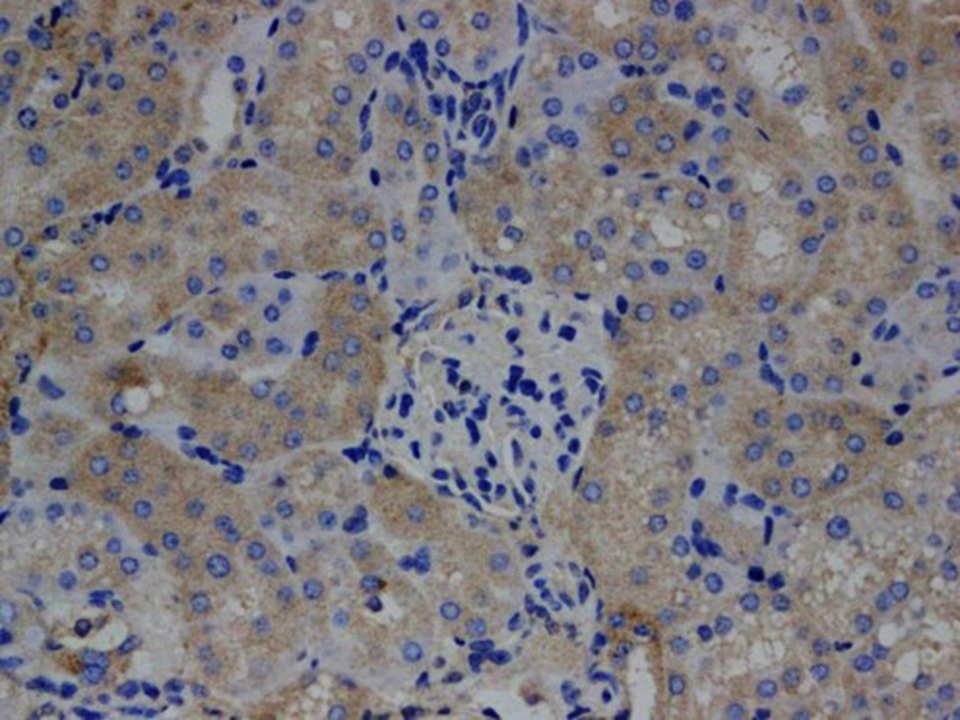

Supplement: S7 File — (ZIP) [file pone.0327042.s007.zip › TNFα IHC Original images/DM 75mGy 8w-2.tif]

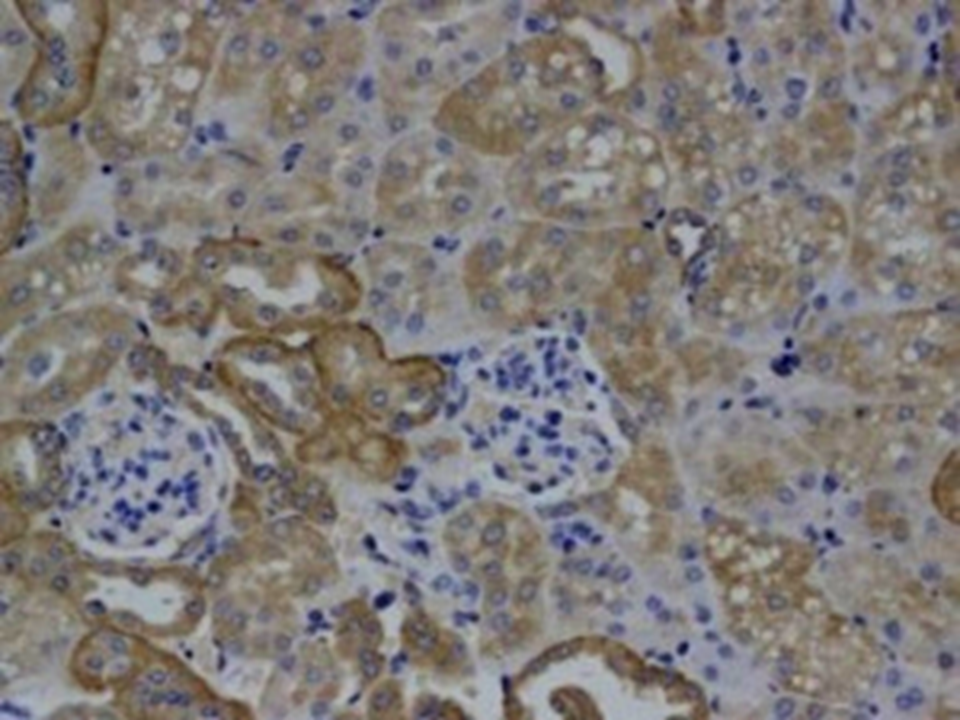

Supplement: S7 File — (ZIP) [file pone.0327042.s007.zip › TNFα IHC Original images/DM-1 8w(Used publication).tif]

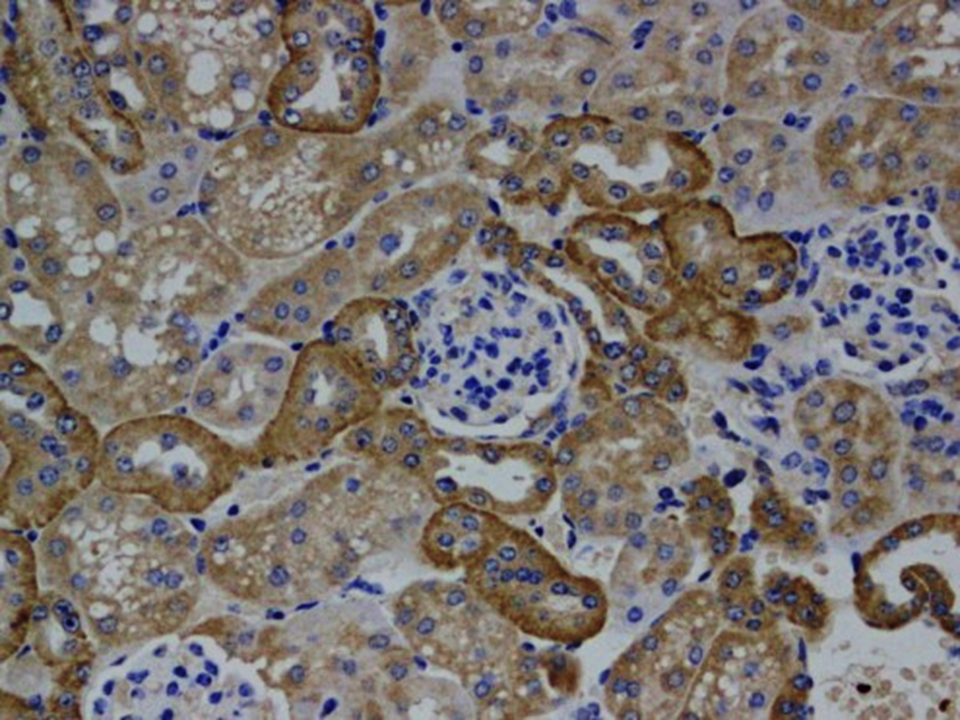

Supplement: S7 File — (ZIP) [file pone.0327042.s007.zip › TNFα IHC Original images/DM-1_2 8w.tif]

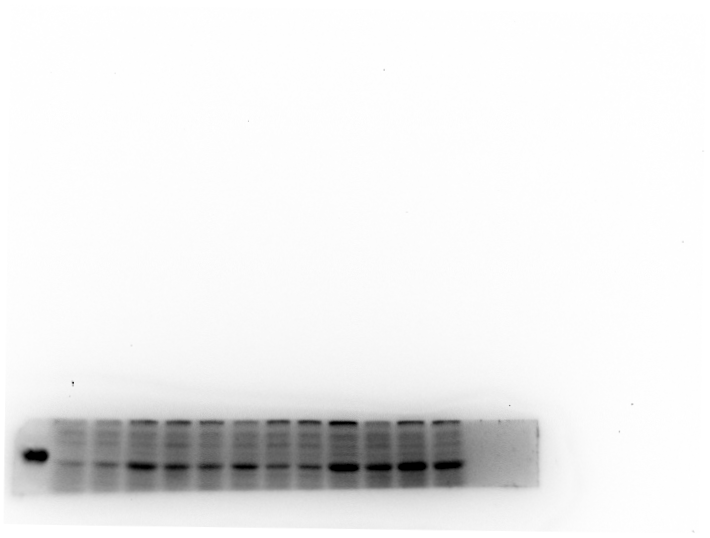

Supplement: S8 File — (ZIP) [file pone.0327042.s008.zip › Fig 5B_PAI-1 blot.tif]

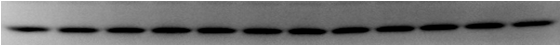

Supplement: S8 File — (ZIP) [file pone.0327042.s008.zip › actin PAI.TIF]

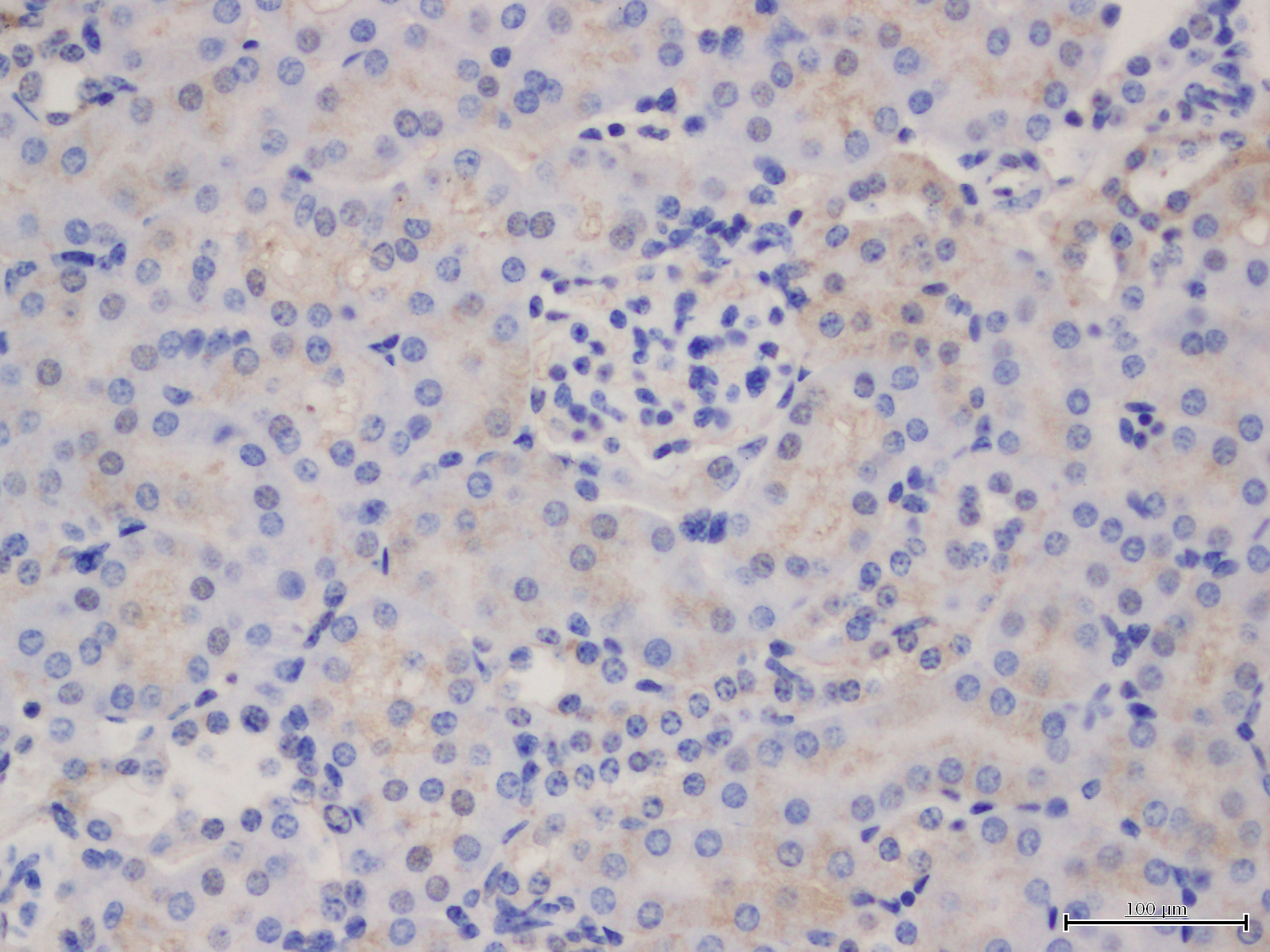

Supplement: S9 File — (ZIP) [file pone.0327042.s009.zip › 8w con-1(Used publication).TIF]

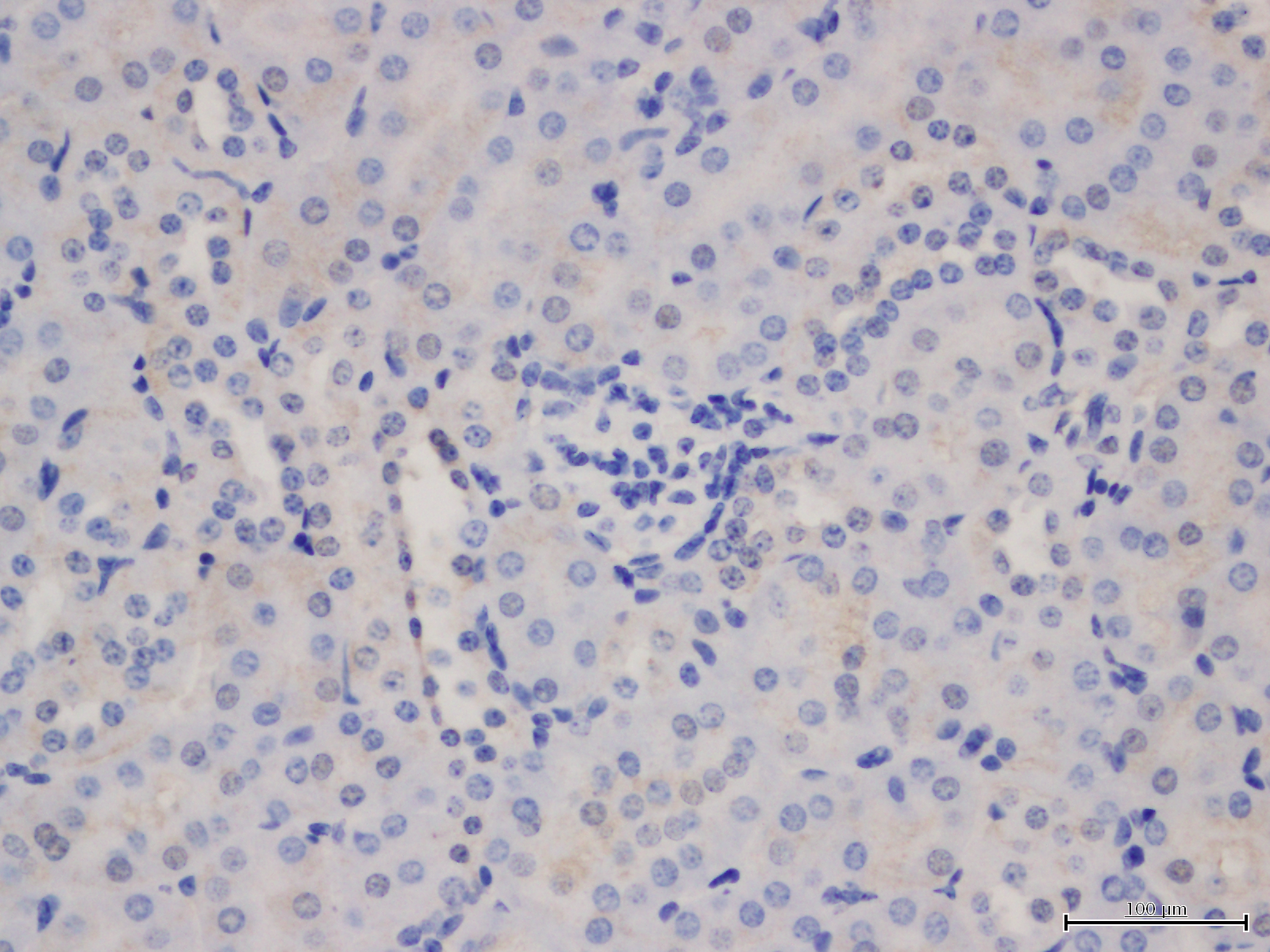

Supplement: S9 File — (ZIP) [file pone.0327042.s009.zip › 4w Con 50mGy-1(Used publication).TIF]

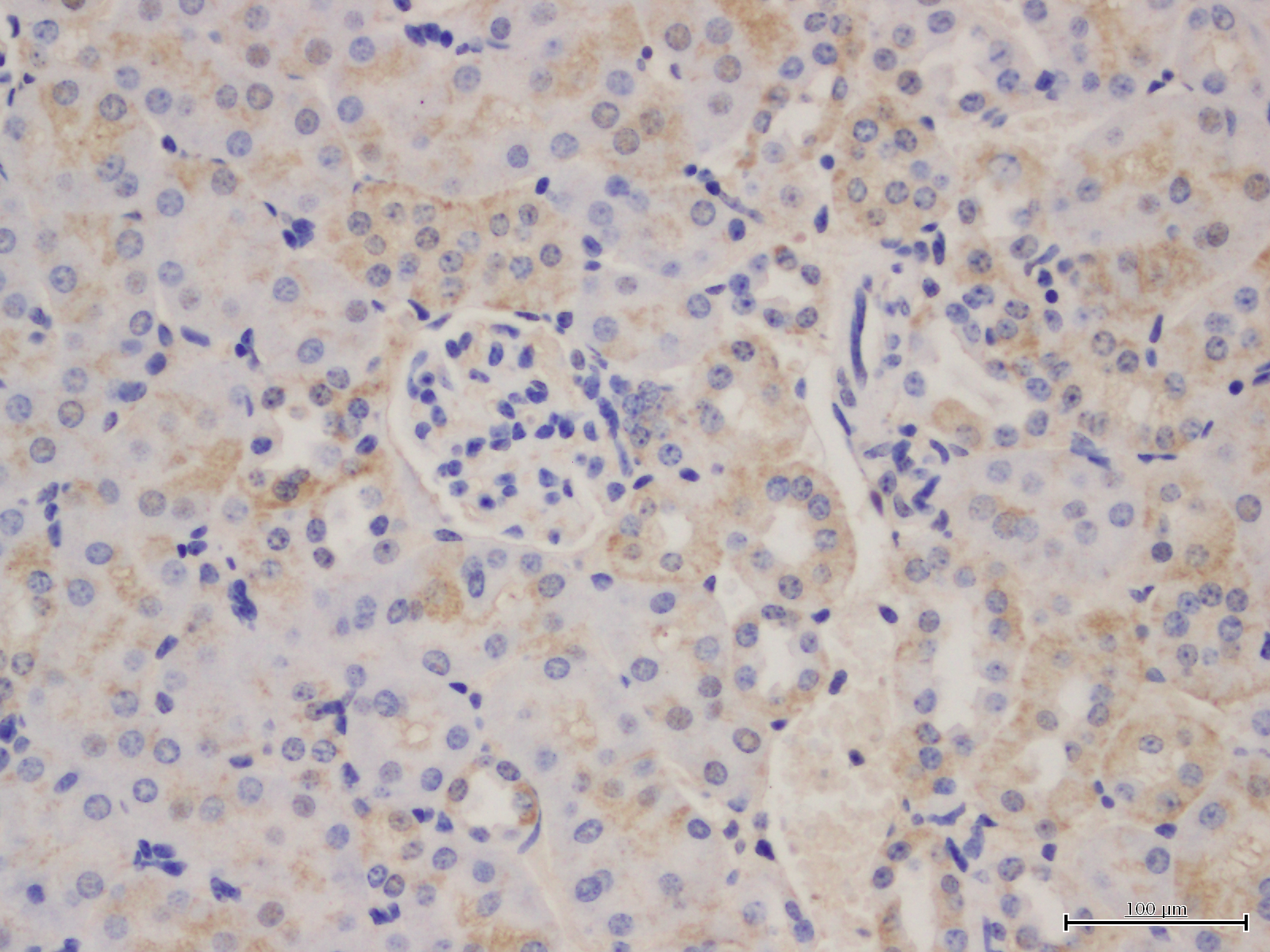

Supplement: S9 File — (ZIP) [file pone.0327042.s009.zip › 4w con 50mGy-2.TIF]

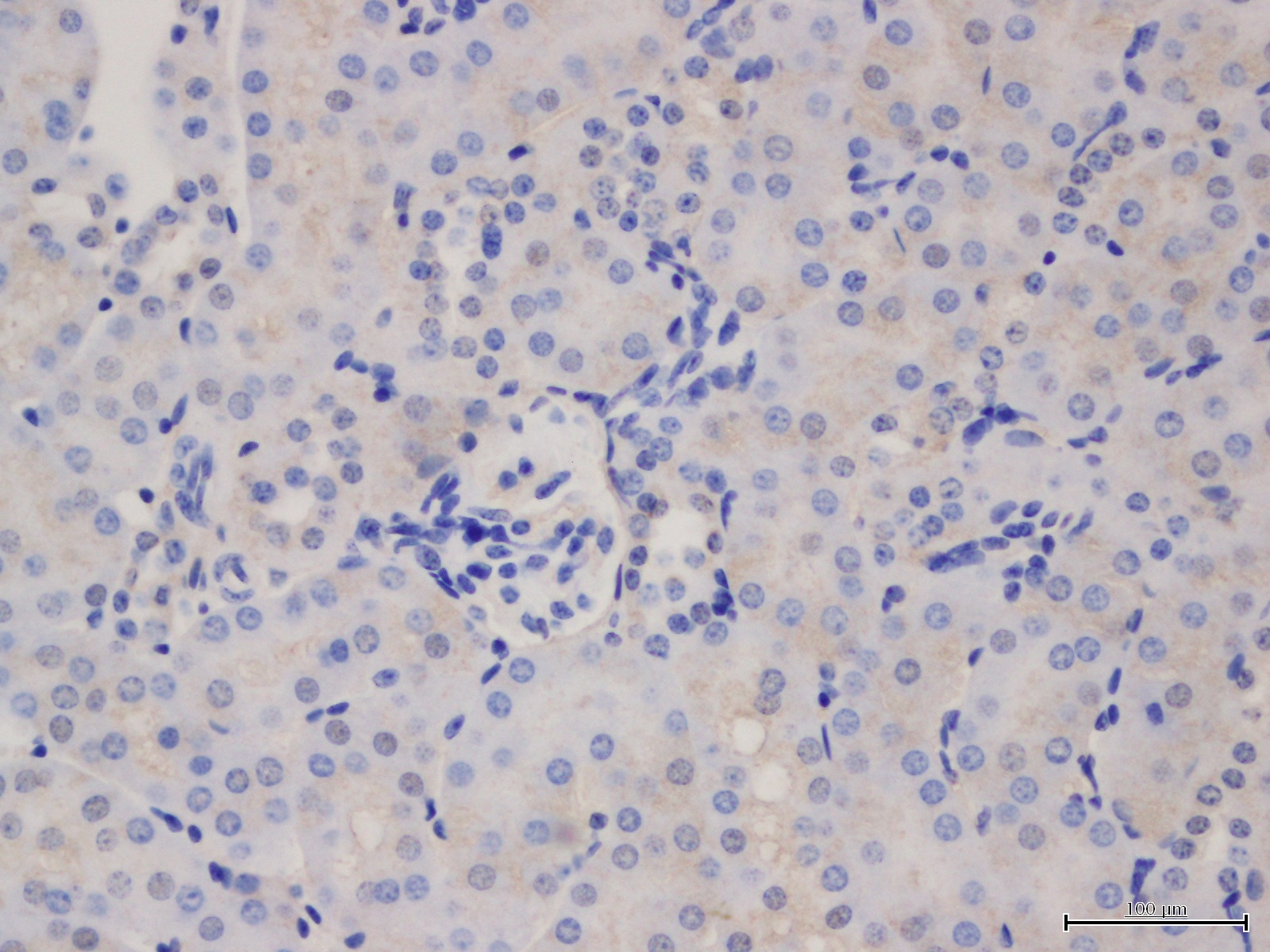

Supplement: S9 File — (ZIP) [file pone.0327042.s009.zip › 4w Con-1(Used publication).TIF]

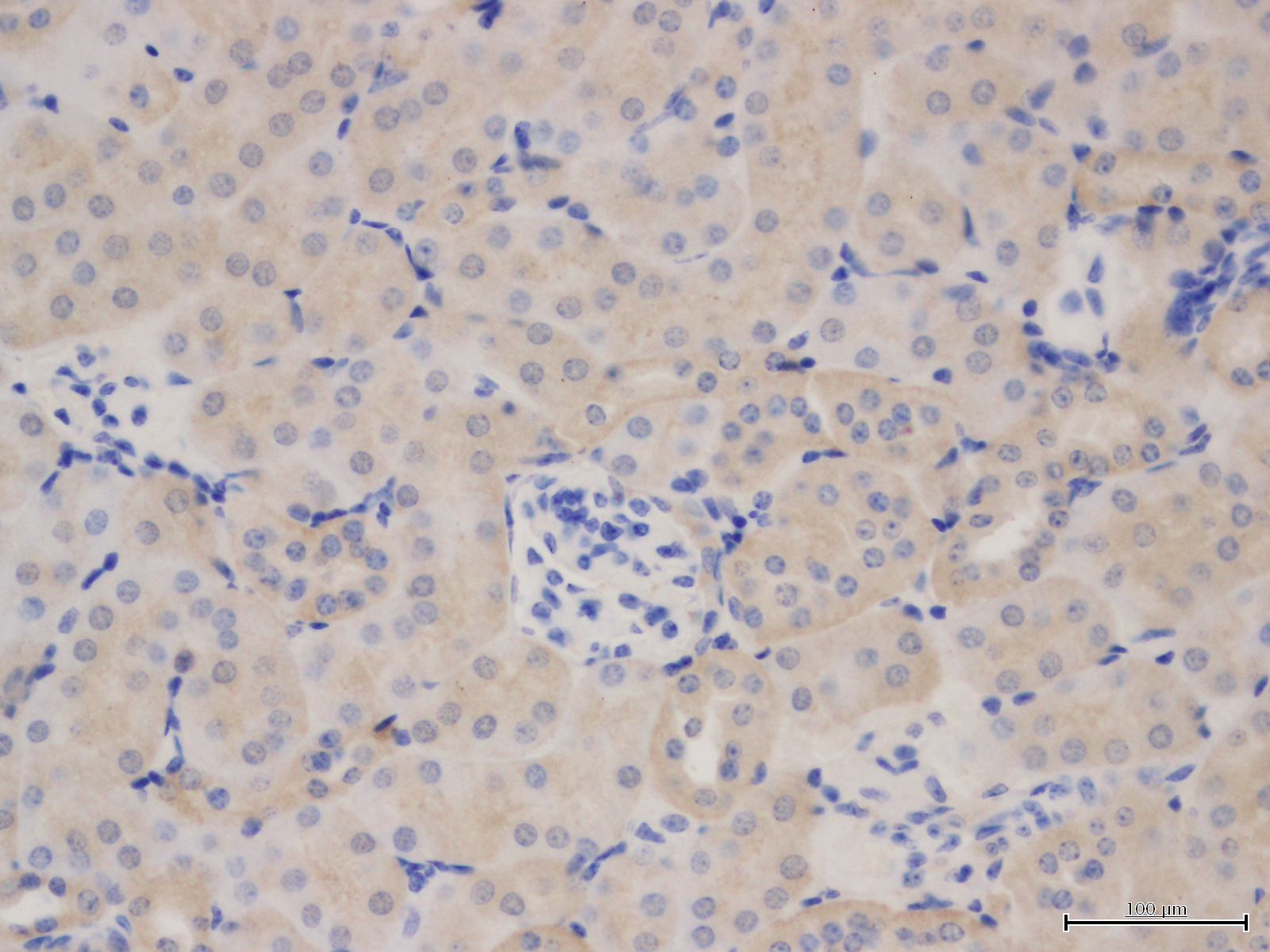

Supplement: S9 File — (ZIP) [file pone.0327042.s009.zip › 4w Con-2.TIF]

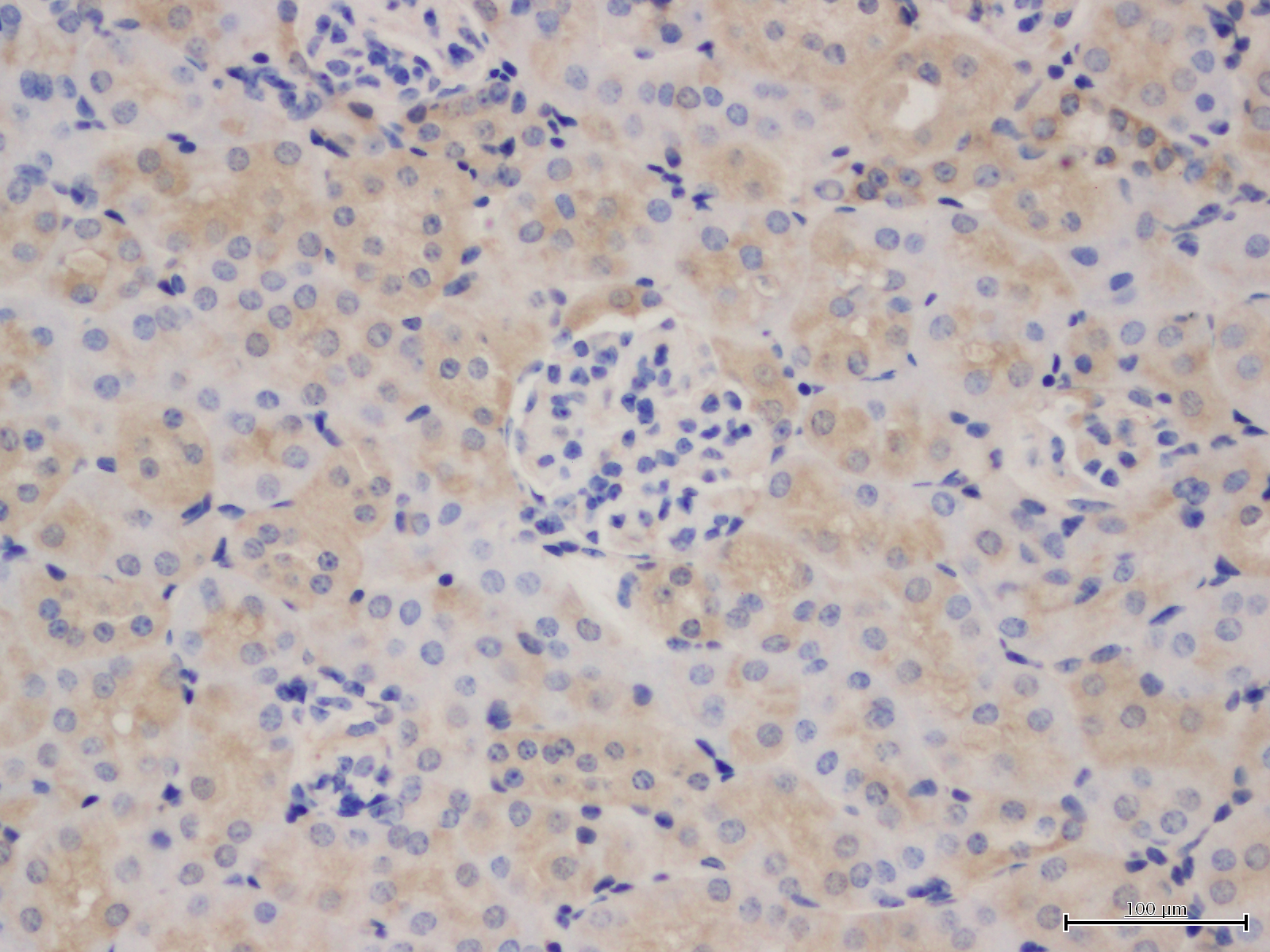

Supplement: S9 File — (ZIP) [file pone.0327042.s009.zip › 4w Con-3.TIF]

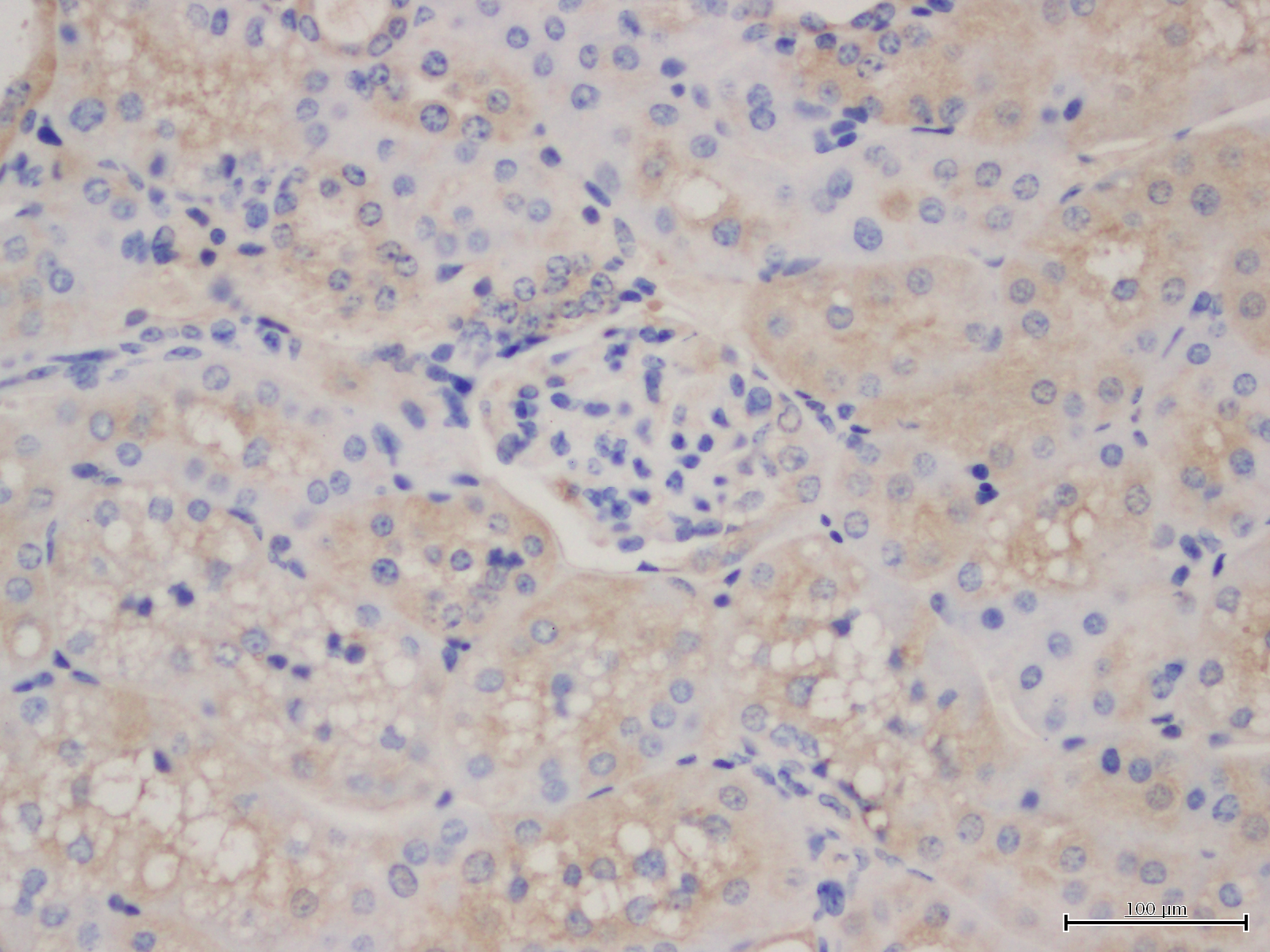

Supplement: S9 File — (ZIP) [file pone.0327042.s009.zip › 4w DM 25 mGy-1(Used publication).TIF]

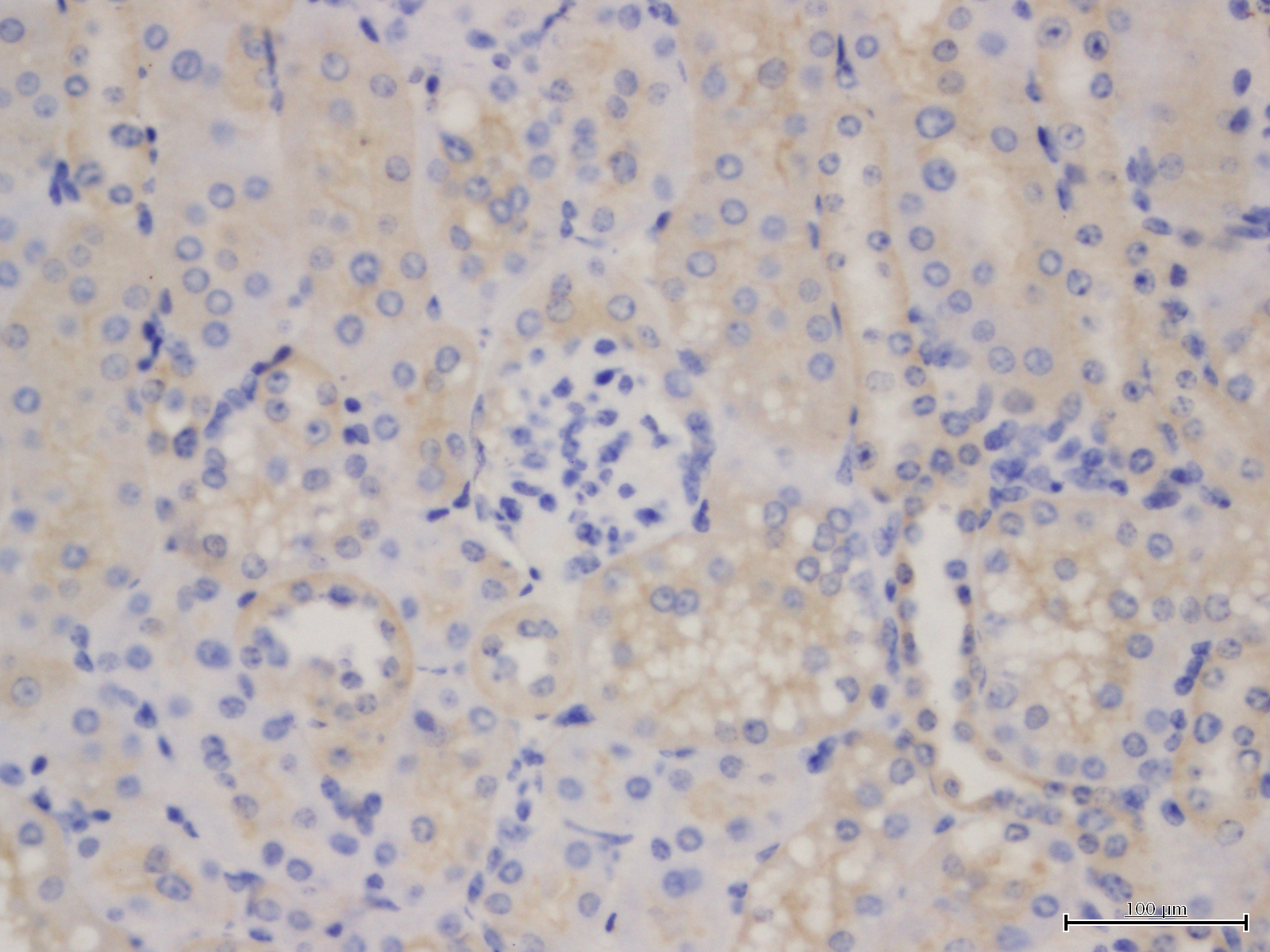

Supplement: S9 File — (ZIP) [file pone.0327042.s009.zip › 4w DM 25 mGy-2.TIF]

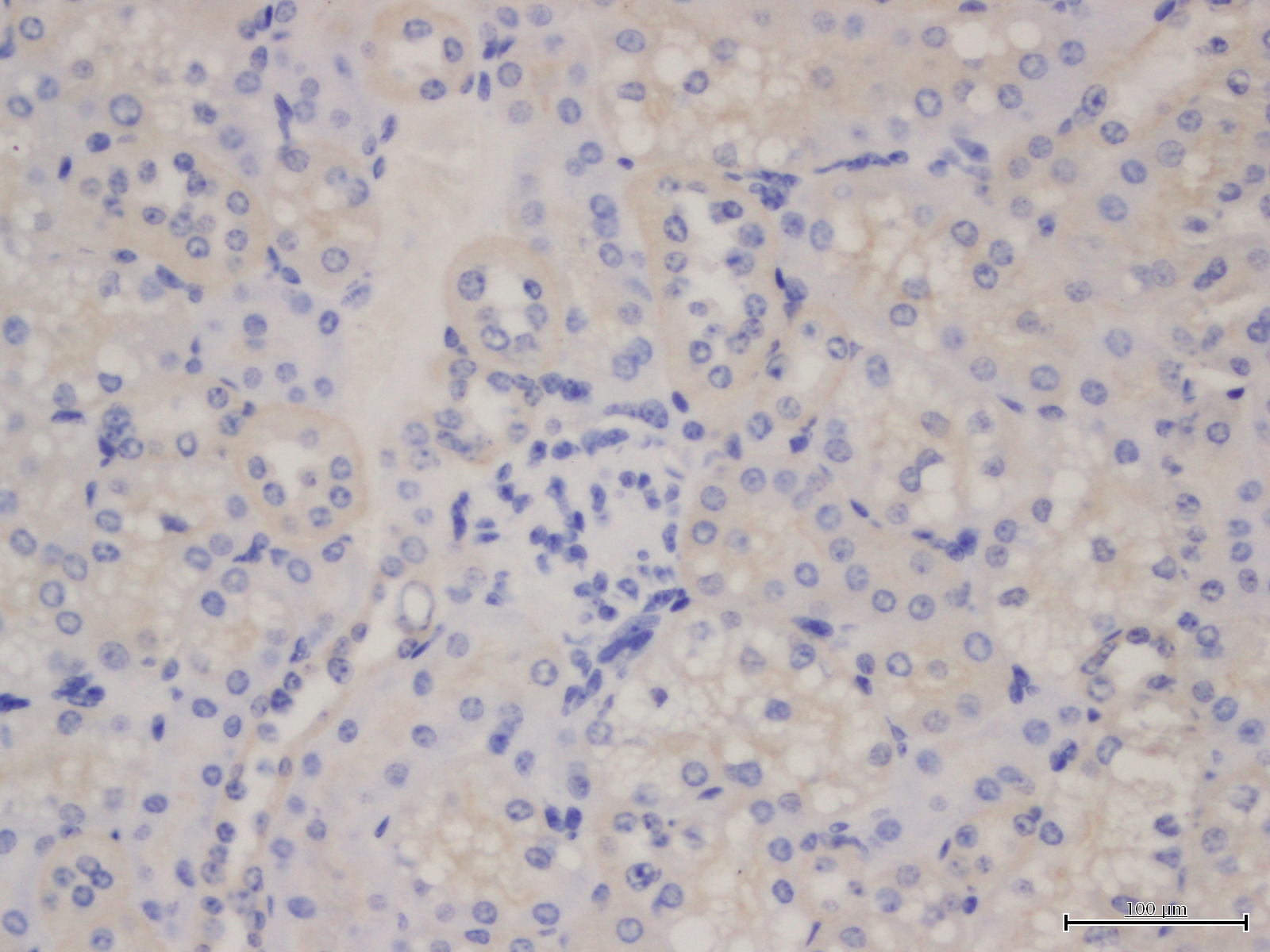

Supplement: S9 File — (ZIP) [file pone.0327042.s009.zip › 4w DM 25mGy-3.TIF]

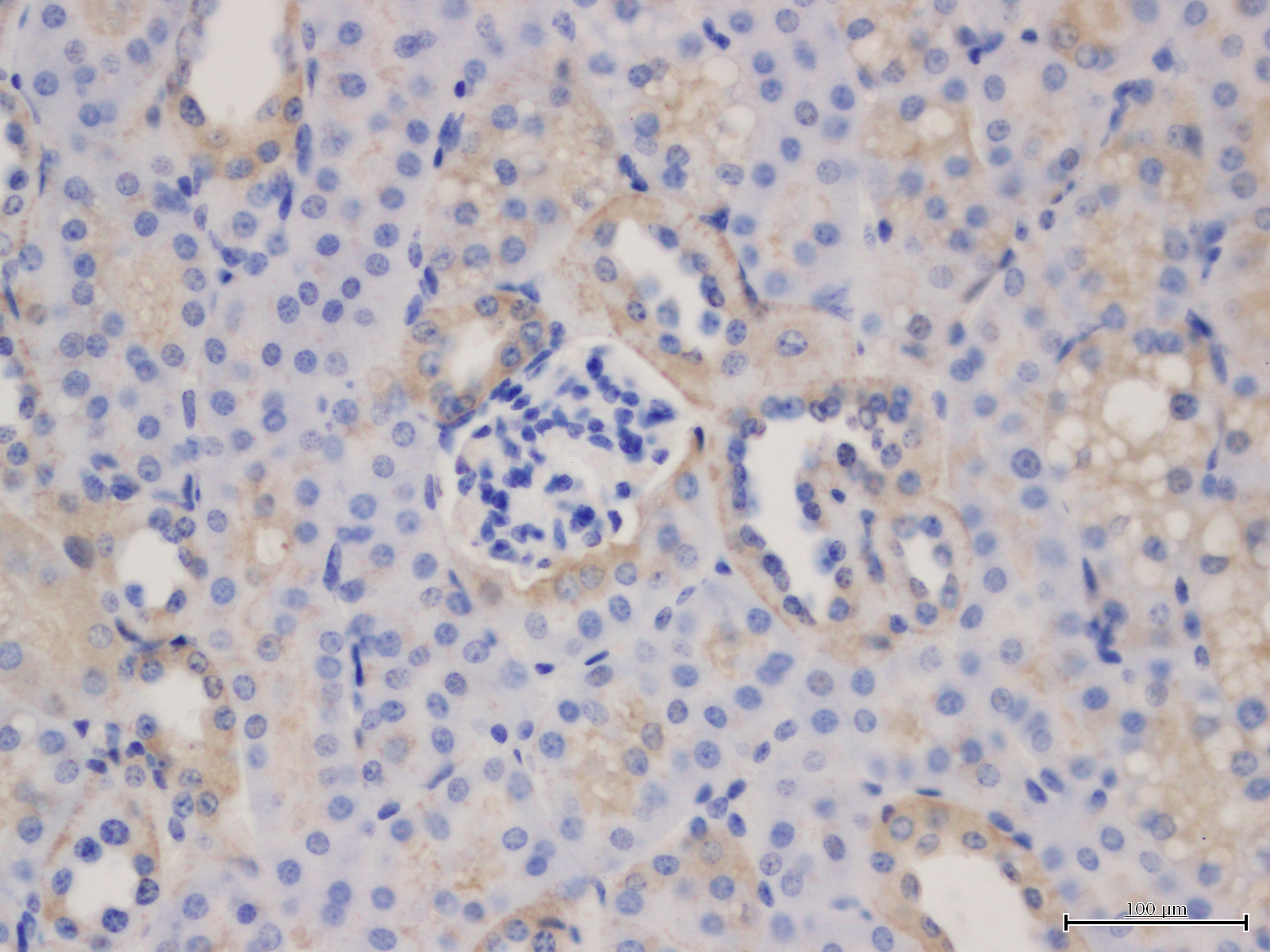

Supplement: S9 File — (ZIP) [file pone.0327042.s009.zip › 4w DM 50mGy-1(Used publication).TIF]

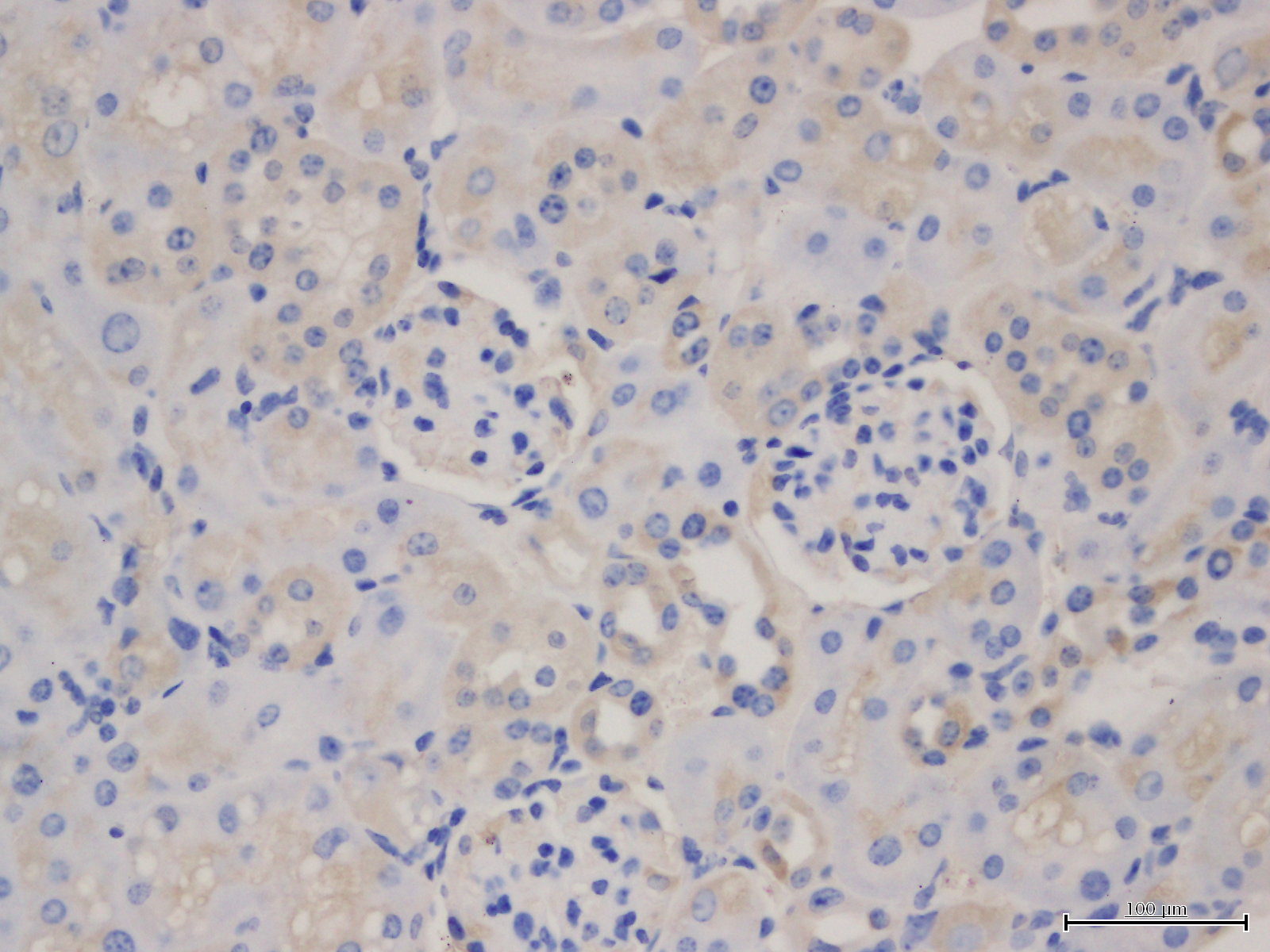

Supplement: S9 File — (ZIP) [file pone.0327042.s009.zip › 4w DM 50mGy-2.TIF]

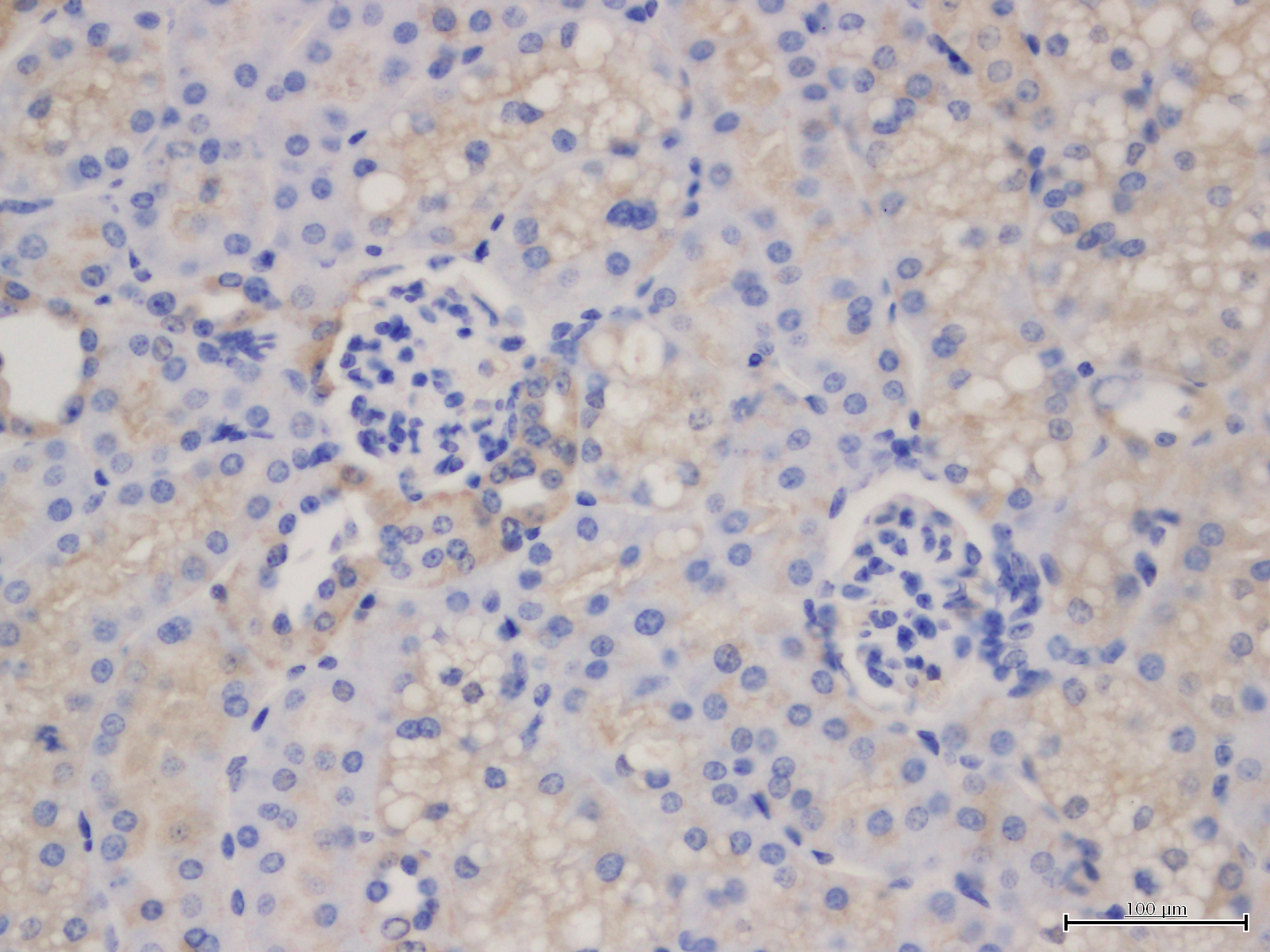

Supplement: S9 File — (ZIP) [file pone.0327042.s009.zip › 4w DM 75mGy-1(Used publication).TIF]

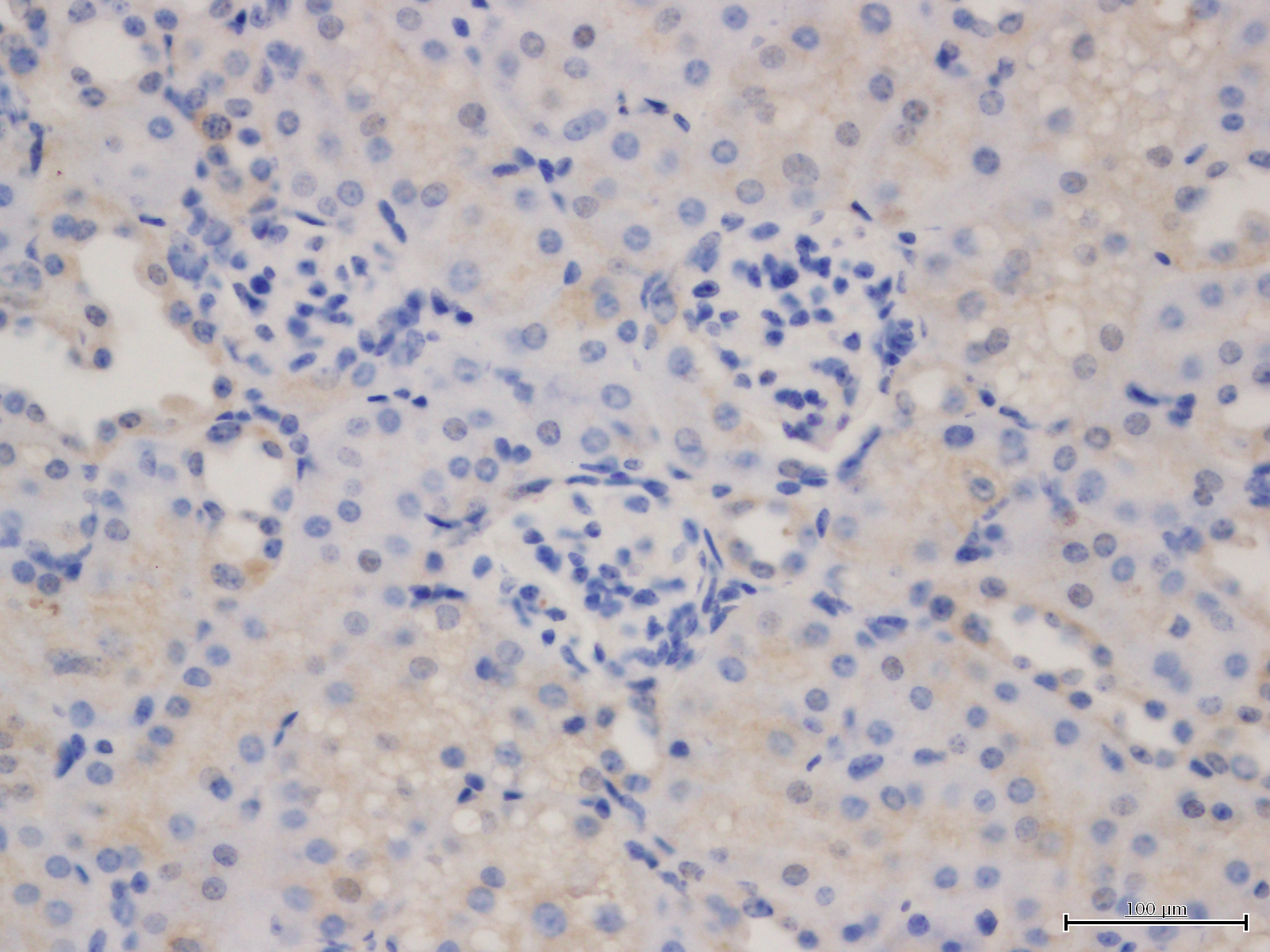

Supplement: S9 File — (ZIP) [file pone.0327042.s009.zip › 4w DM 75mGy-2.TIF]

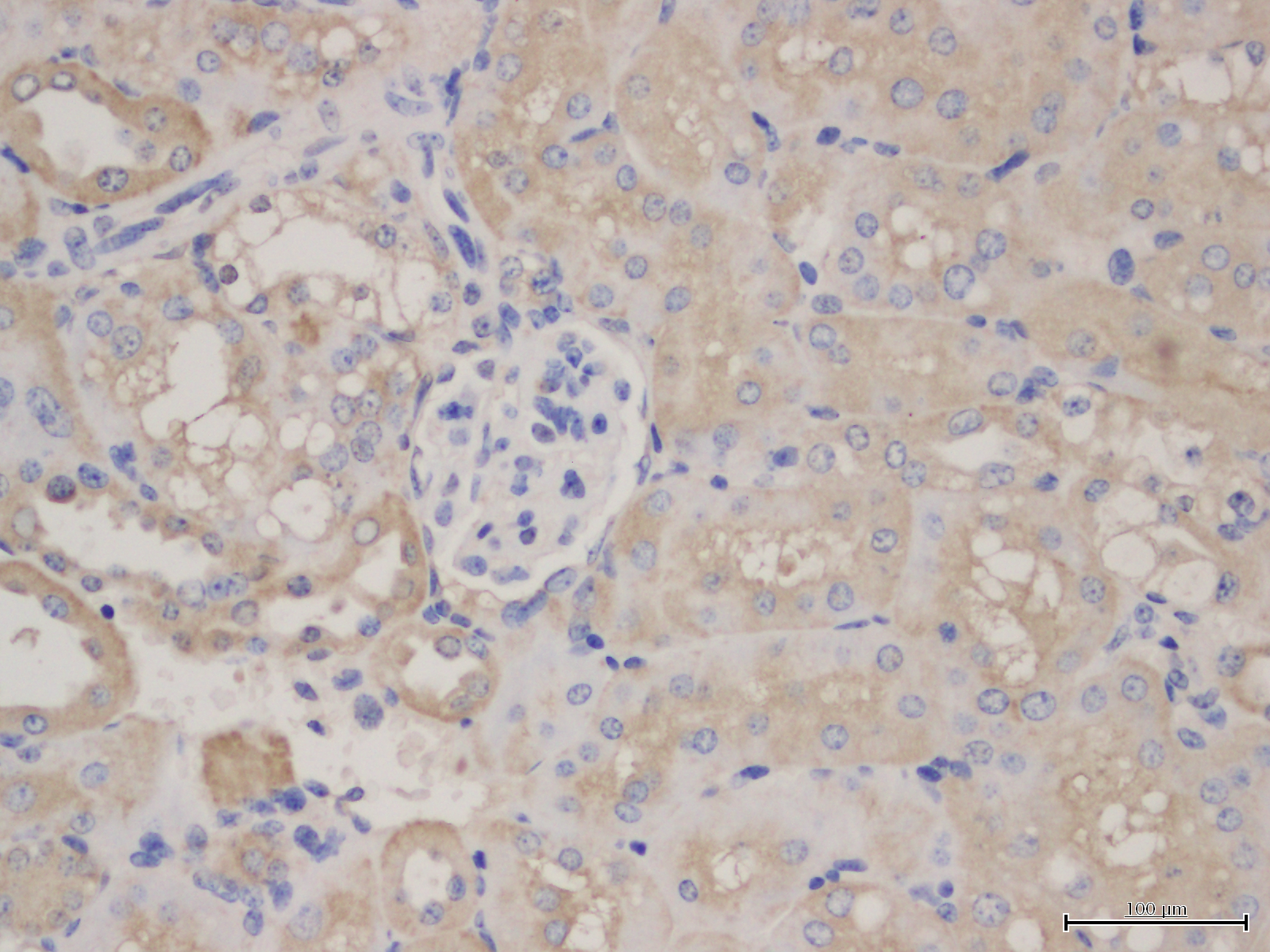

Supplement: S9 File — (ZIP) [file pone.0327042.s009.zip › 4w DM-1(Used publication).TIF]

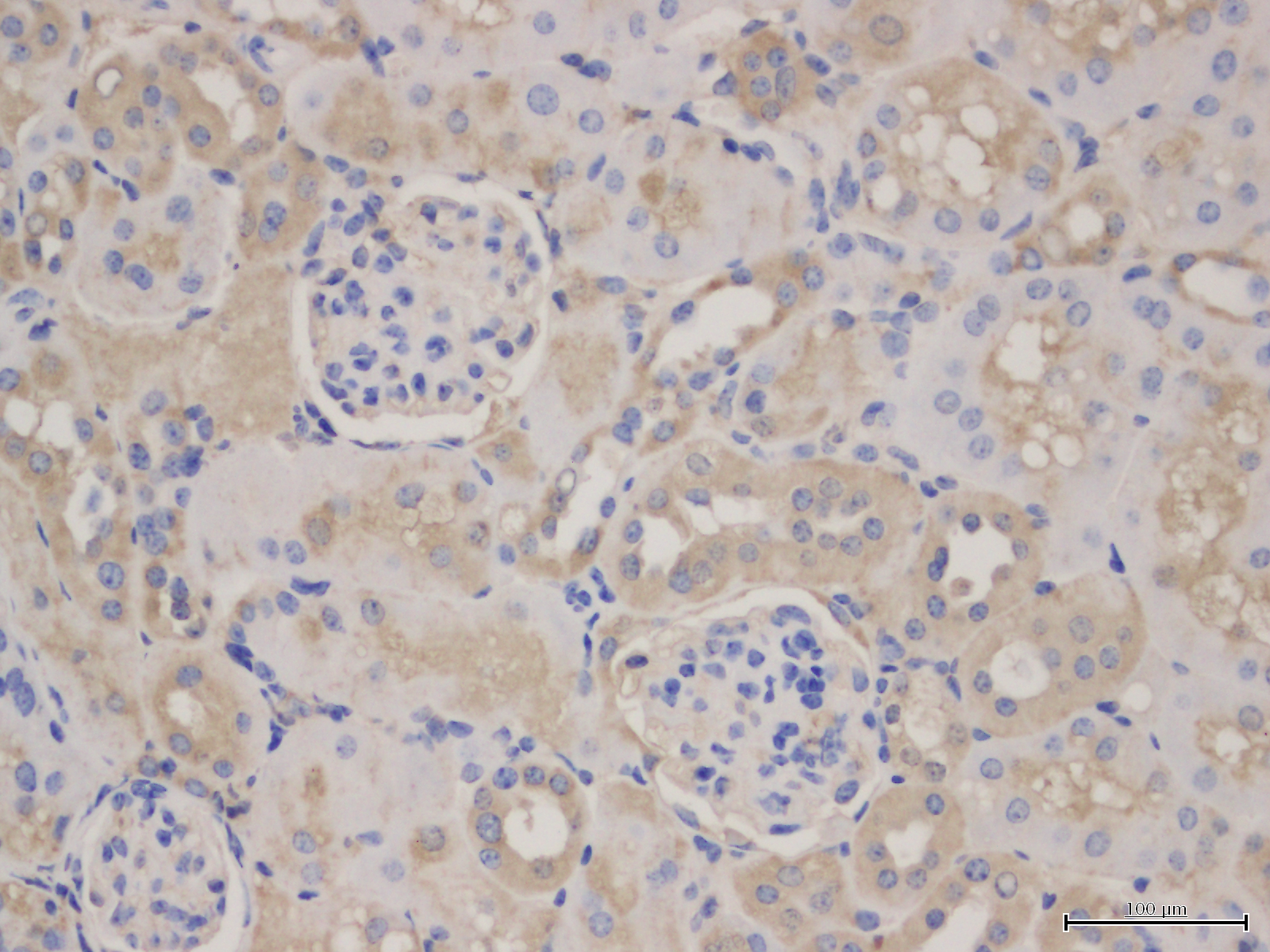

Supplement: S9 File — (ZIP) [file pone.0327042.s009.zip › 4w DM-2.TIF]

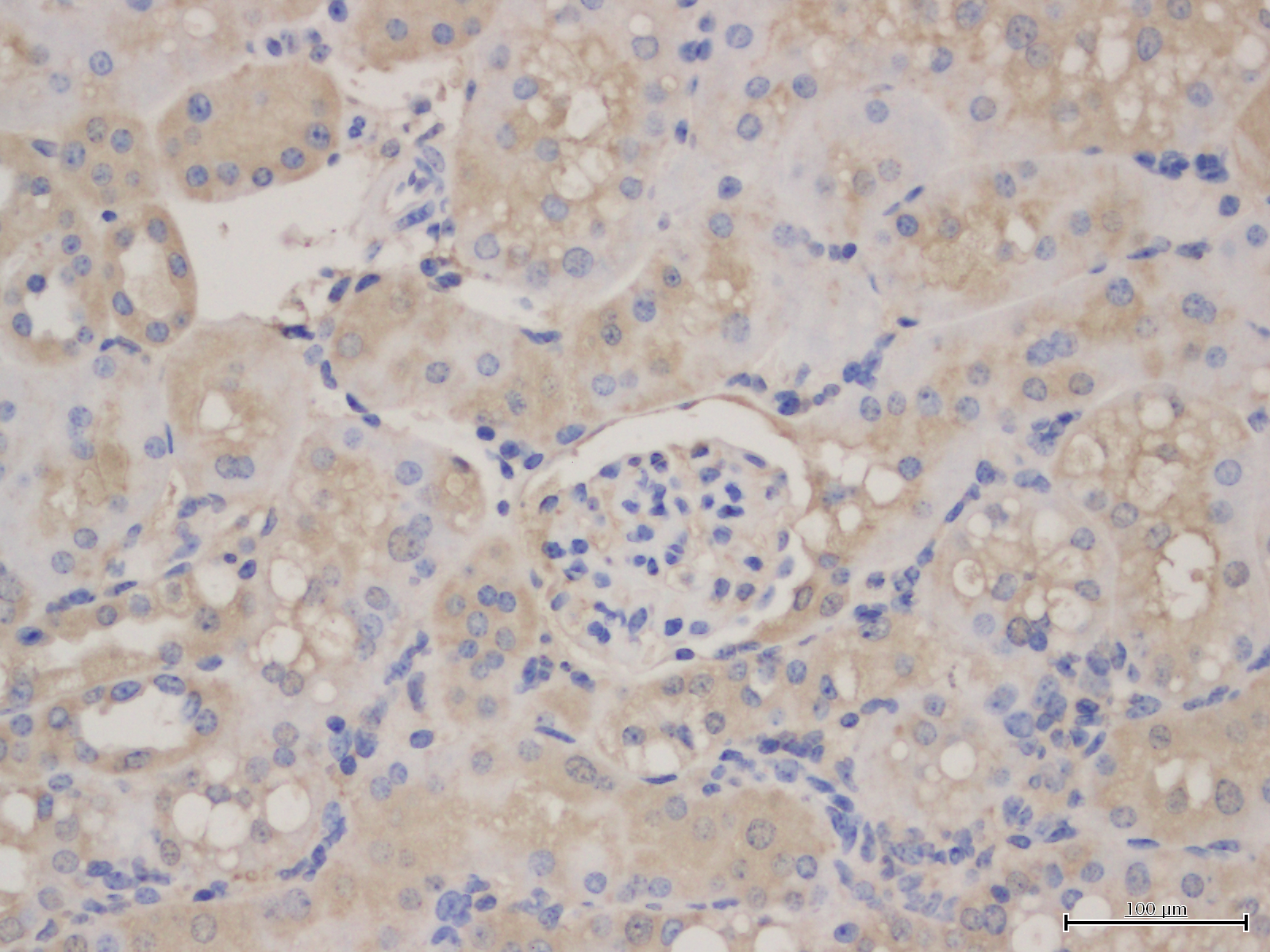

Supplement: S9 File — (ZIP) [file pone.0327042.s009.zip › 4w DM-3.TIF]

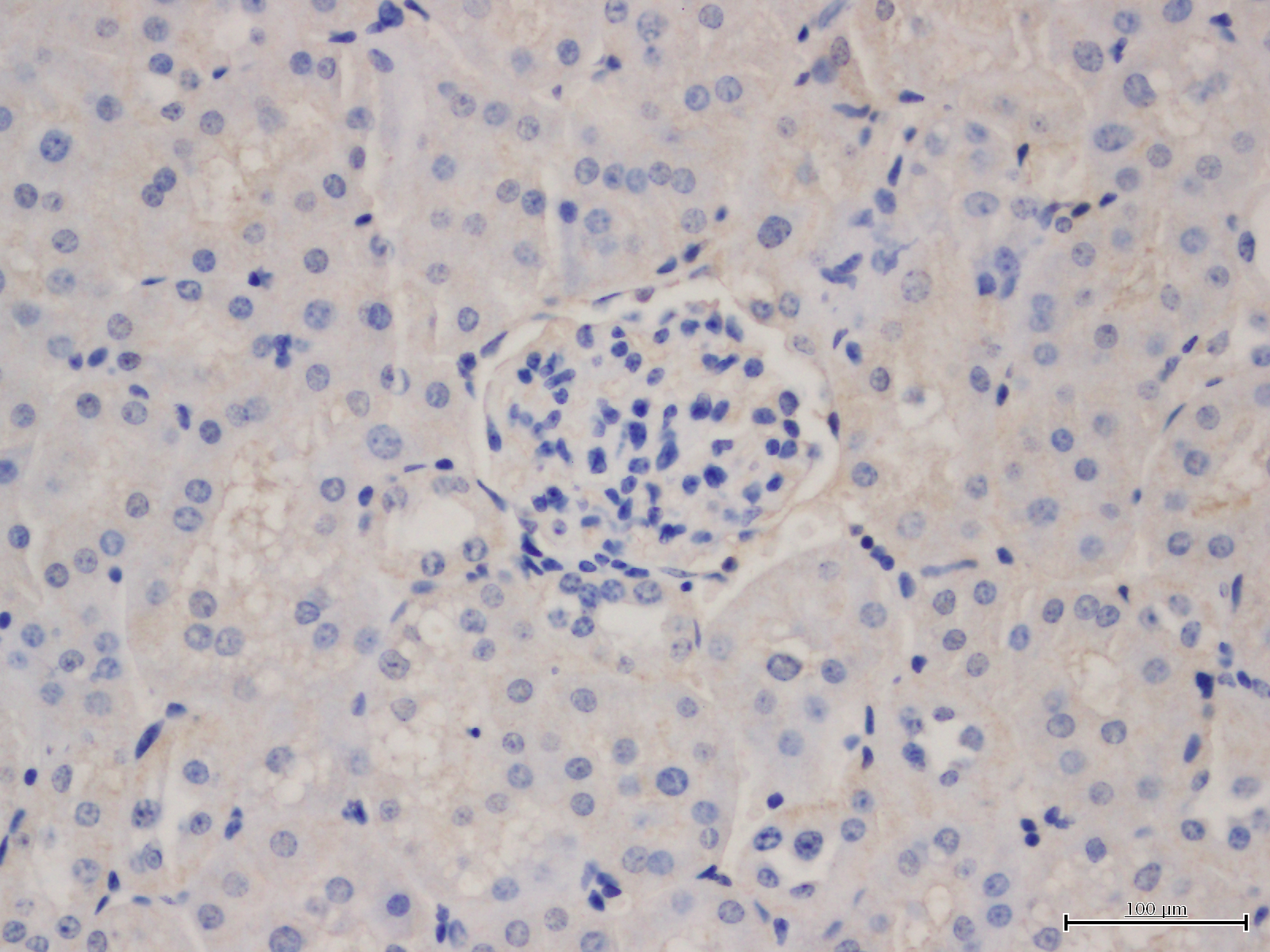

Supplement: S9 File — (ZIP) [file pone.0327042.s009.zip › 8w Con 50mGy-1(Used publication).TIF]

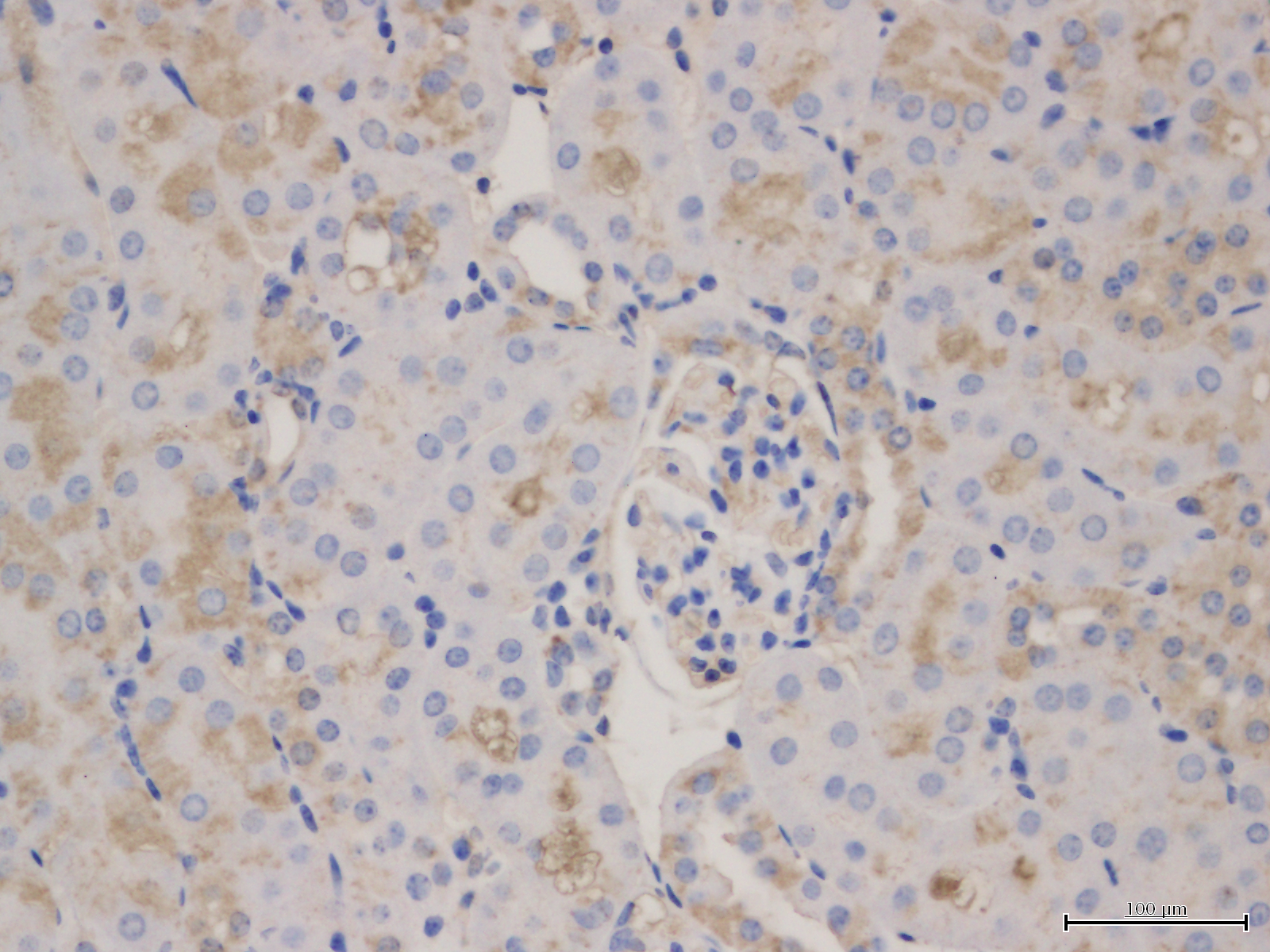

Supplement: S9 File — (ZIP) [file pone.0327042.s009.zip › 8w Con 50mGy-2.TIF]

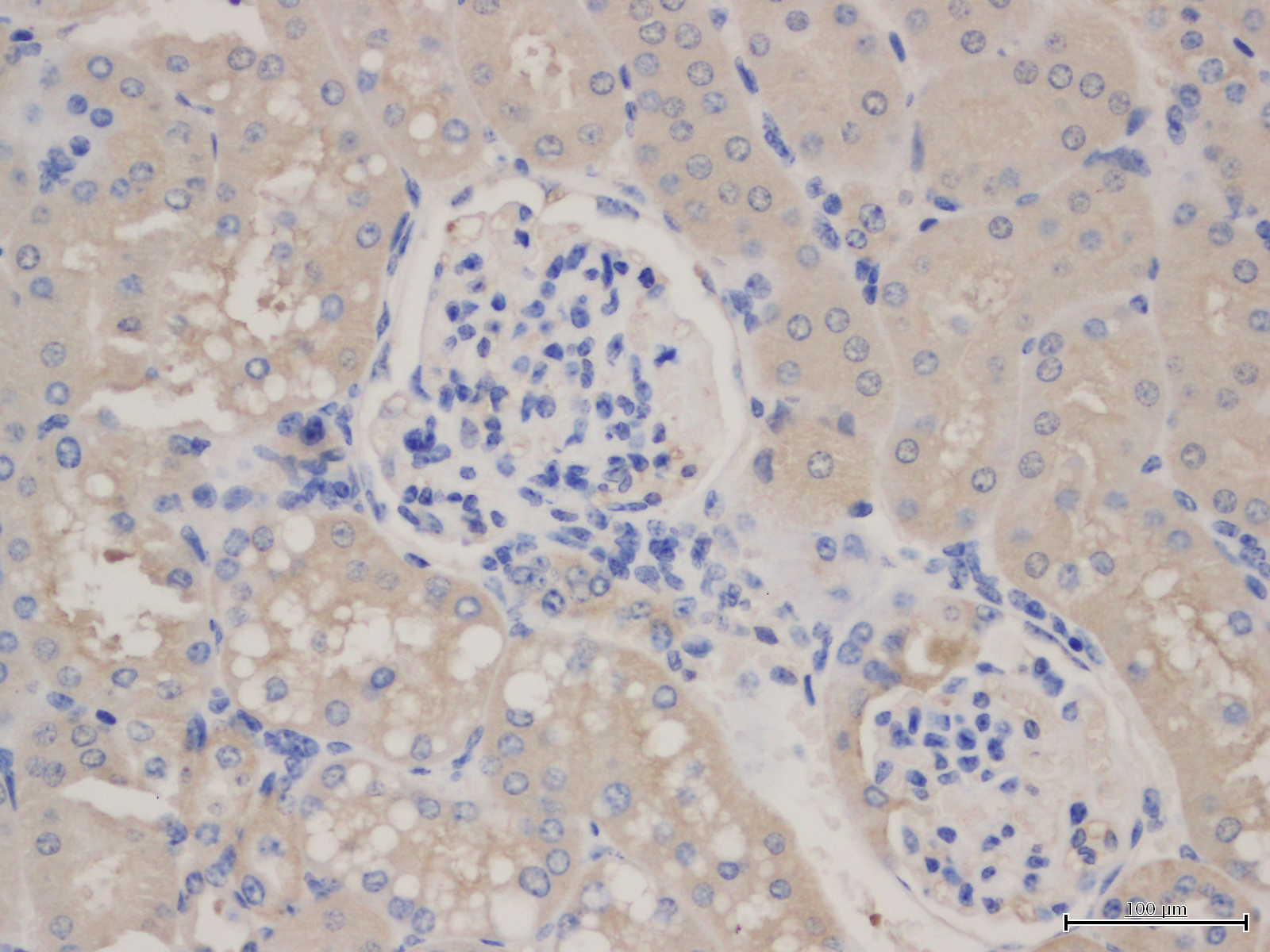

Supplement: S10 File — (ZIP) [file pone.0327042.s010.zip › 8w DM 50mGy-3.TIF]

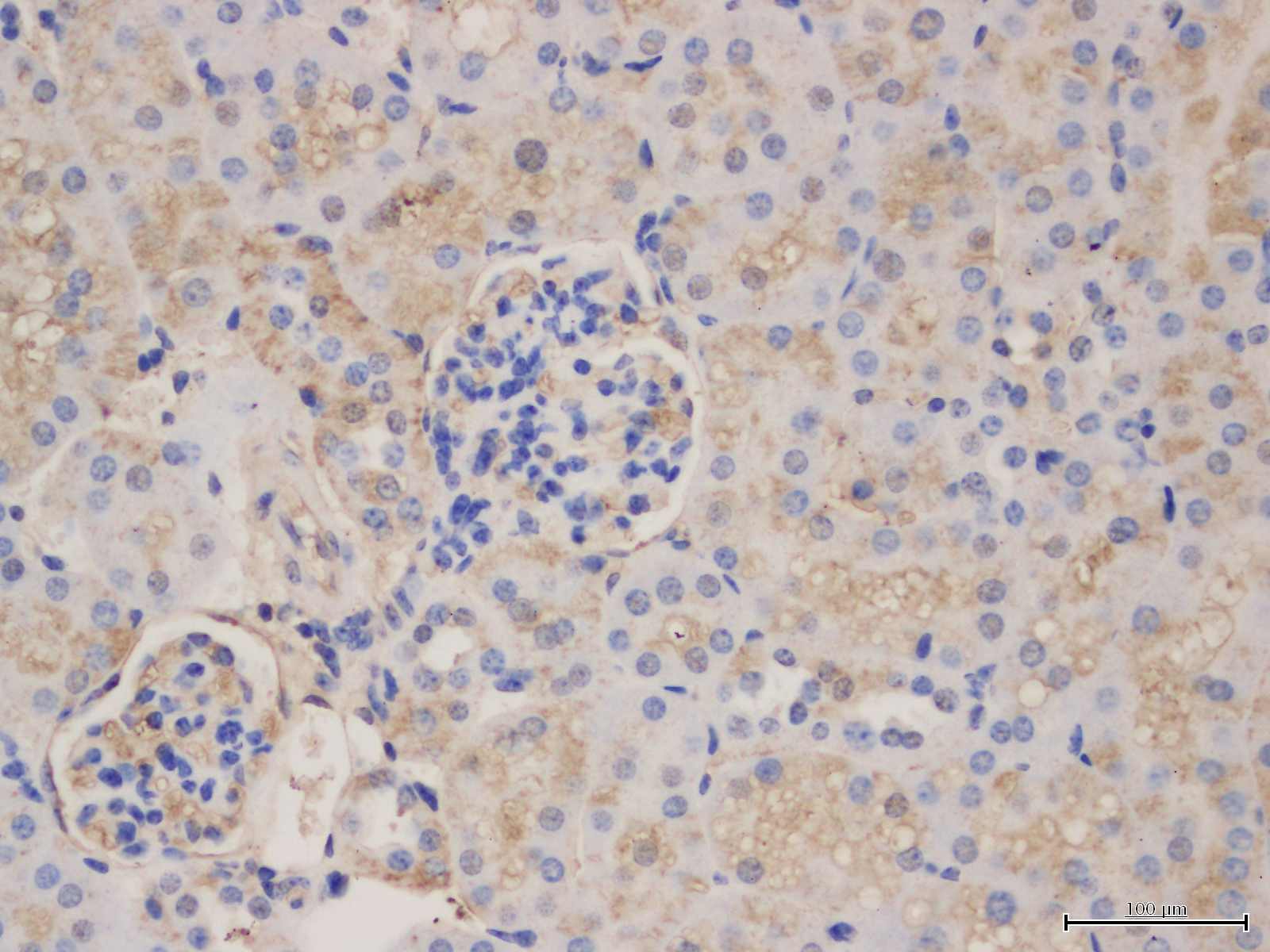

Supplement: S10 File — (ZIP) [file pone.0327042.s010.zip › 8w DM 75 mGy-1.TIF]

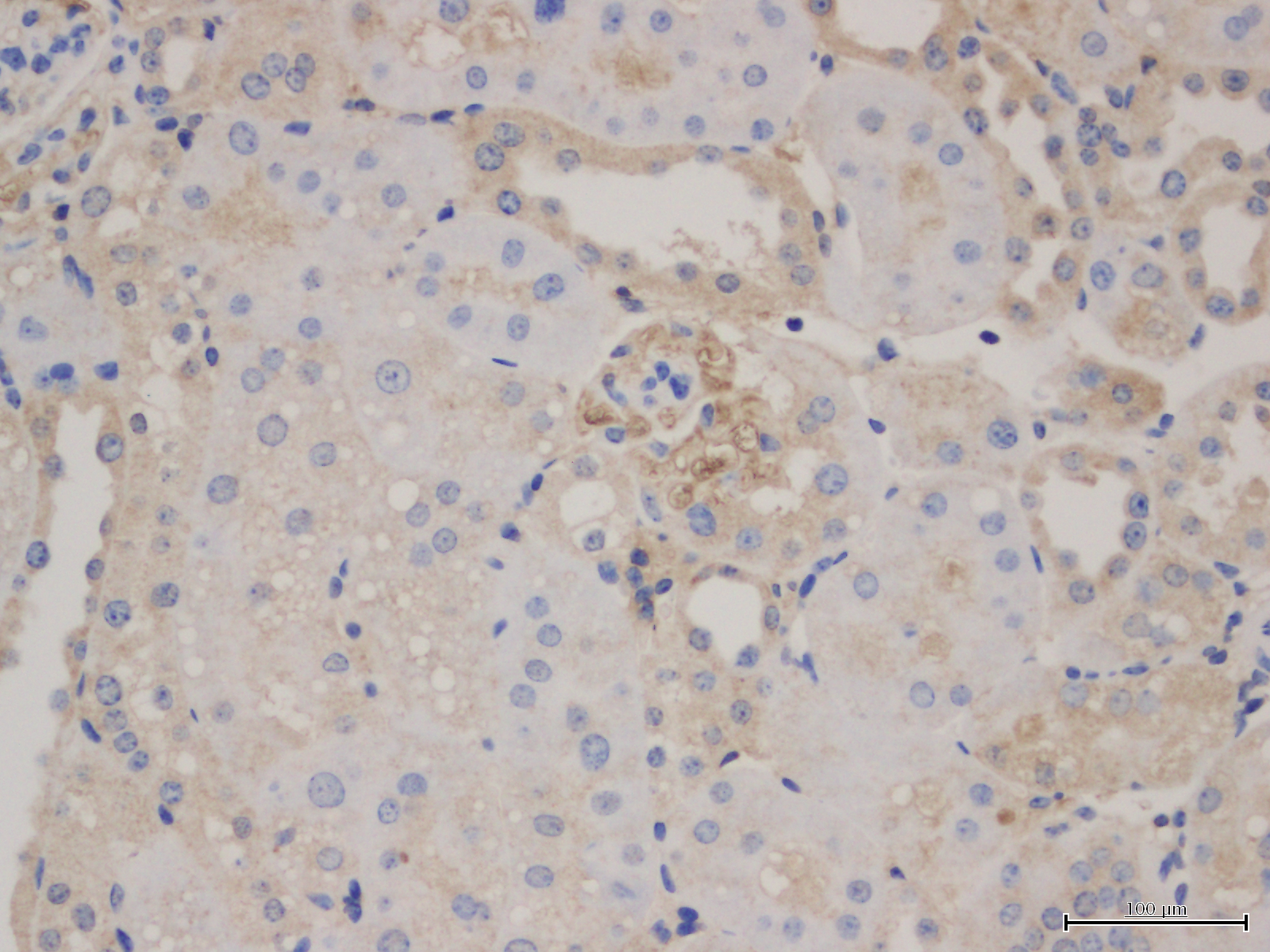

Supplement: S10 File — (ZIP) [file pone.0327042.s010.zip › 8w DM 75mGy-2.TIF]

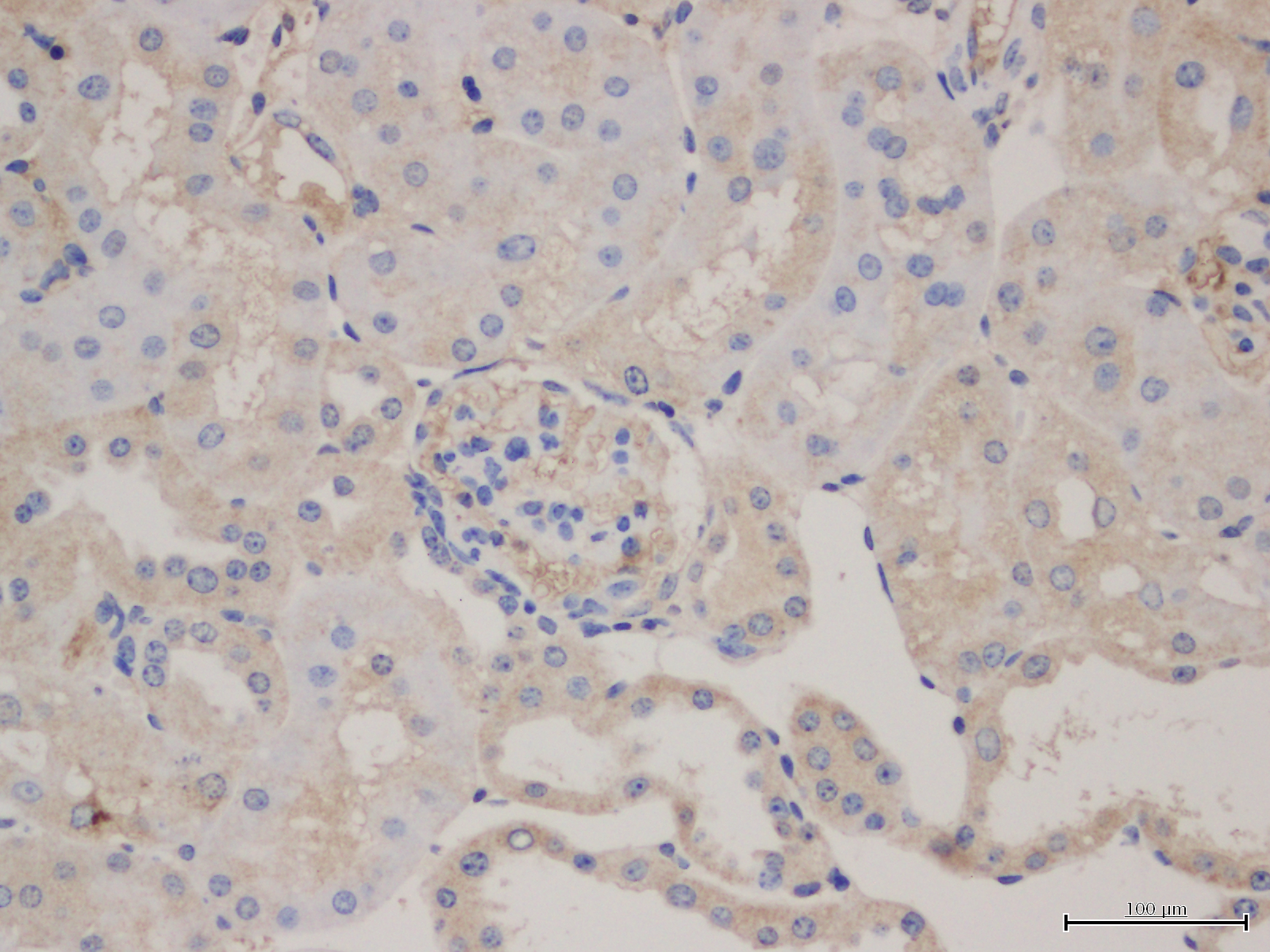

Supplement: S10 File — (ZIP) [file pone.0327042.s010.zip › 8w DM 75mGy-3(Used publication).TIF]

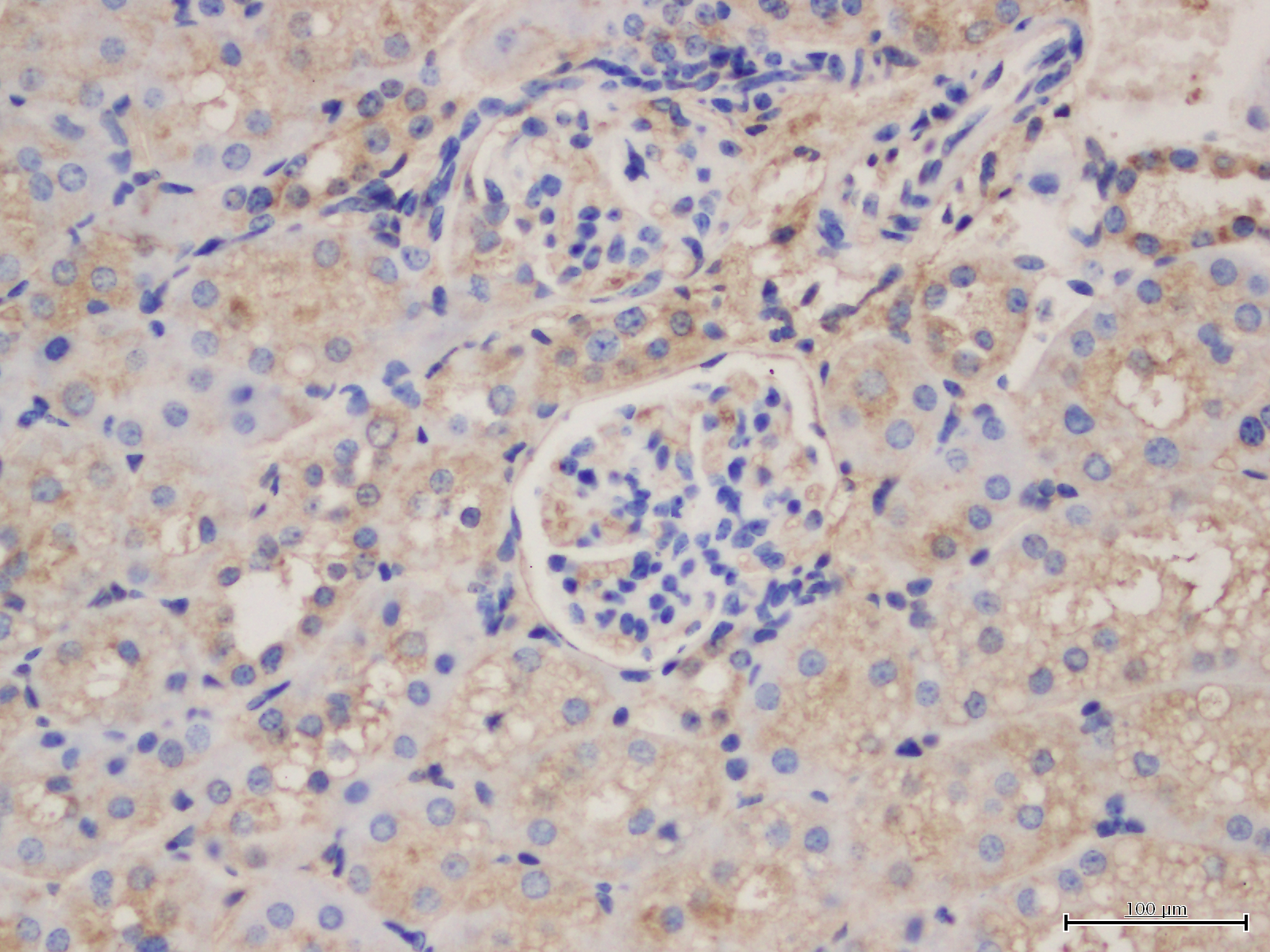

Supplement: S10 File — (ZIP) [file pone.0327042.s010.zip › 8w DM-1(Incorrect used publication for 75mGy 8w group).TIF]

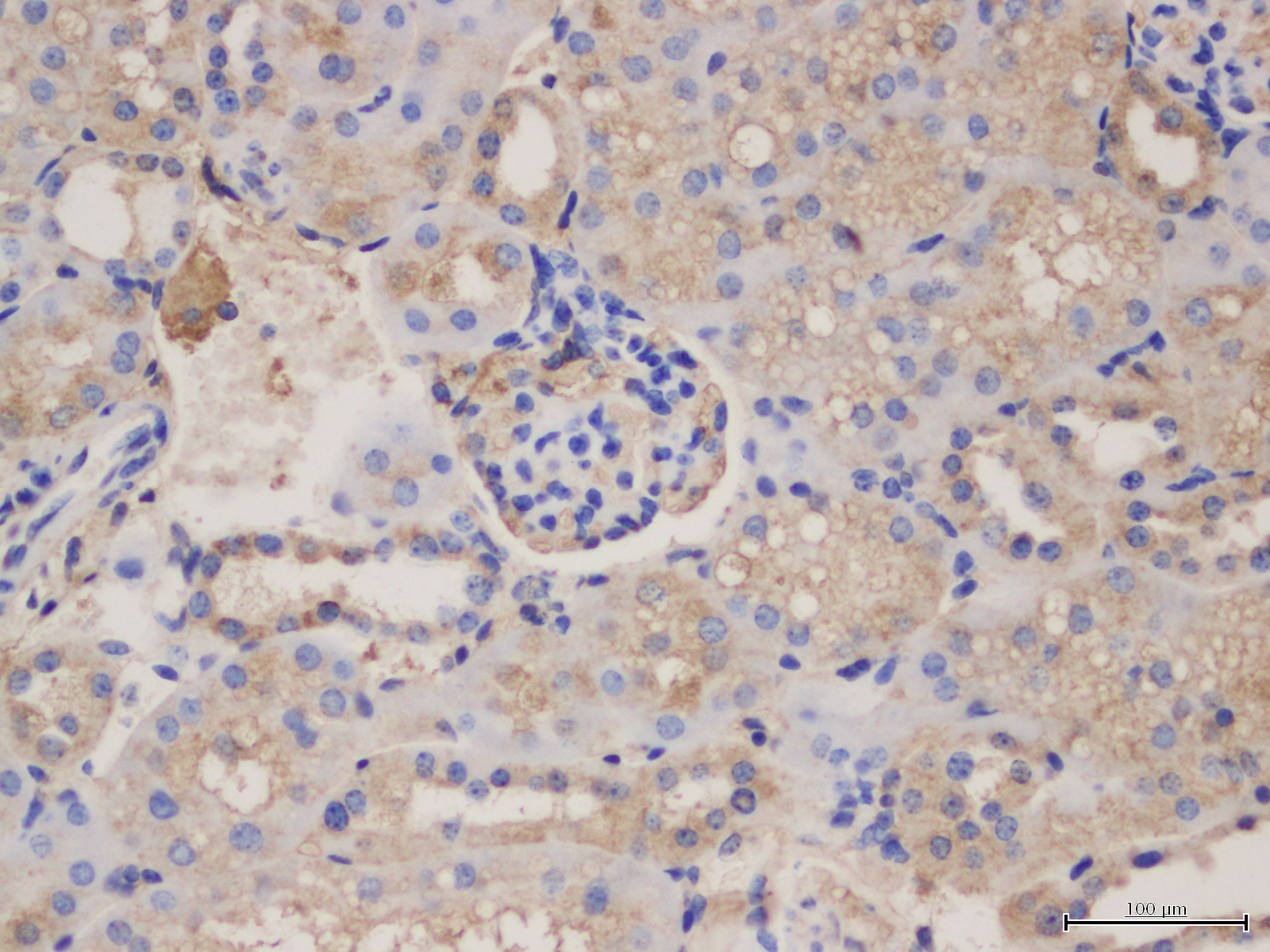

Supplement: S10 File — (ZIP) [file pone.0327042.s010.zip › 8w DM-1_2(Used publication).TIF]

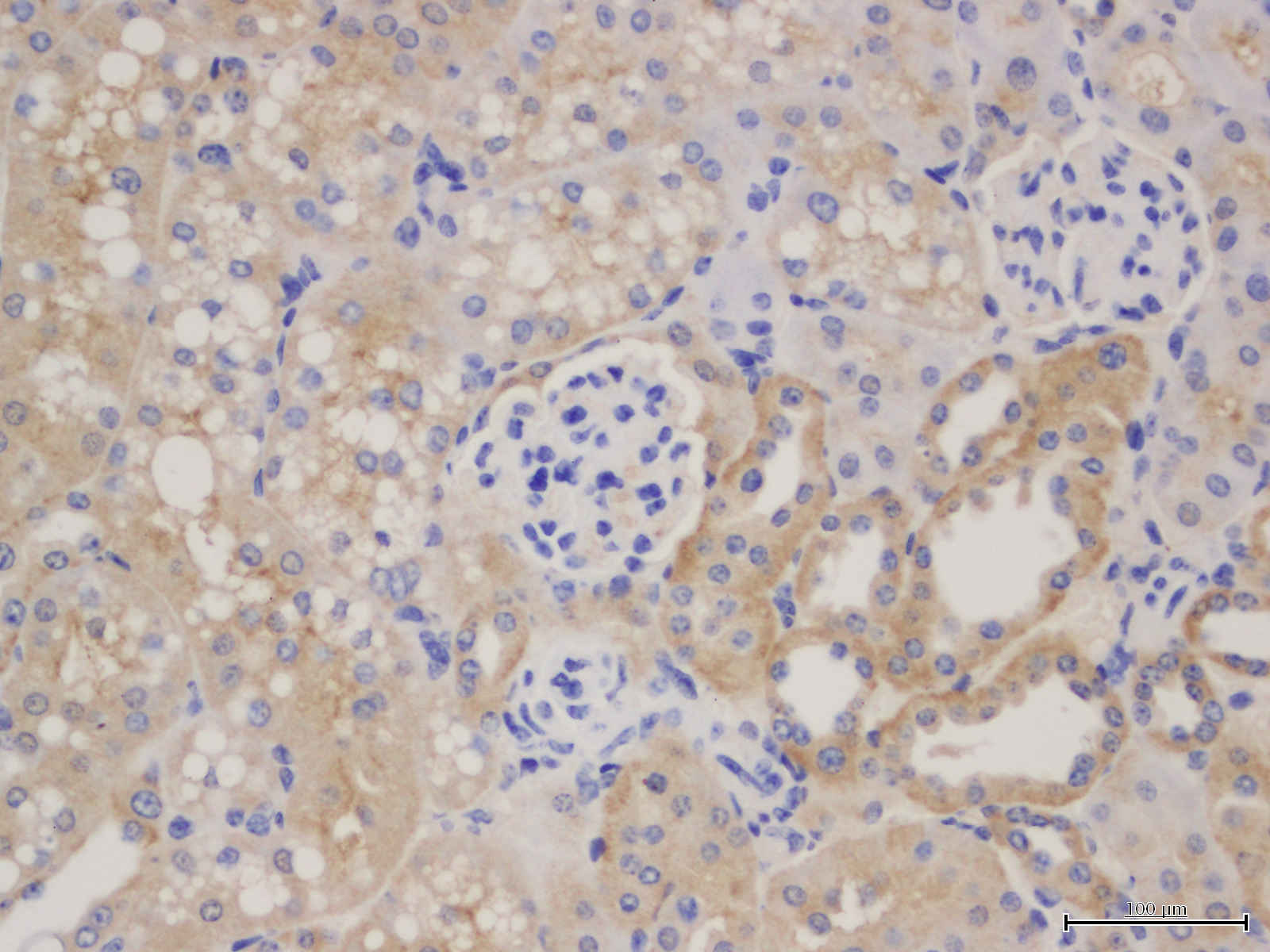

Supplement: S10 File — (ZIP) [file pone.0327042.s010.zip › 8w DM-3.TIF]

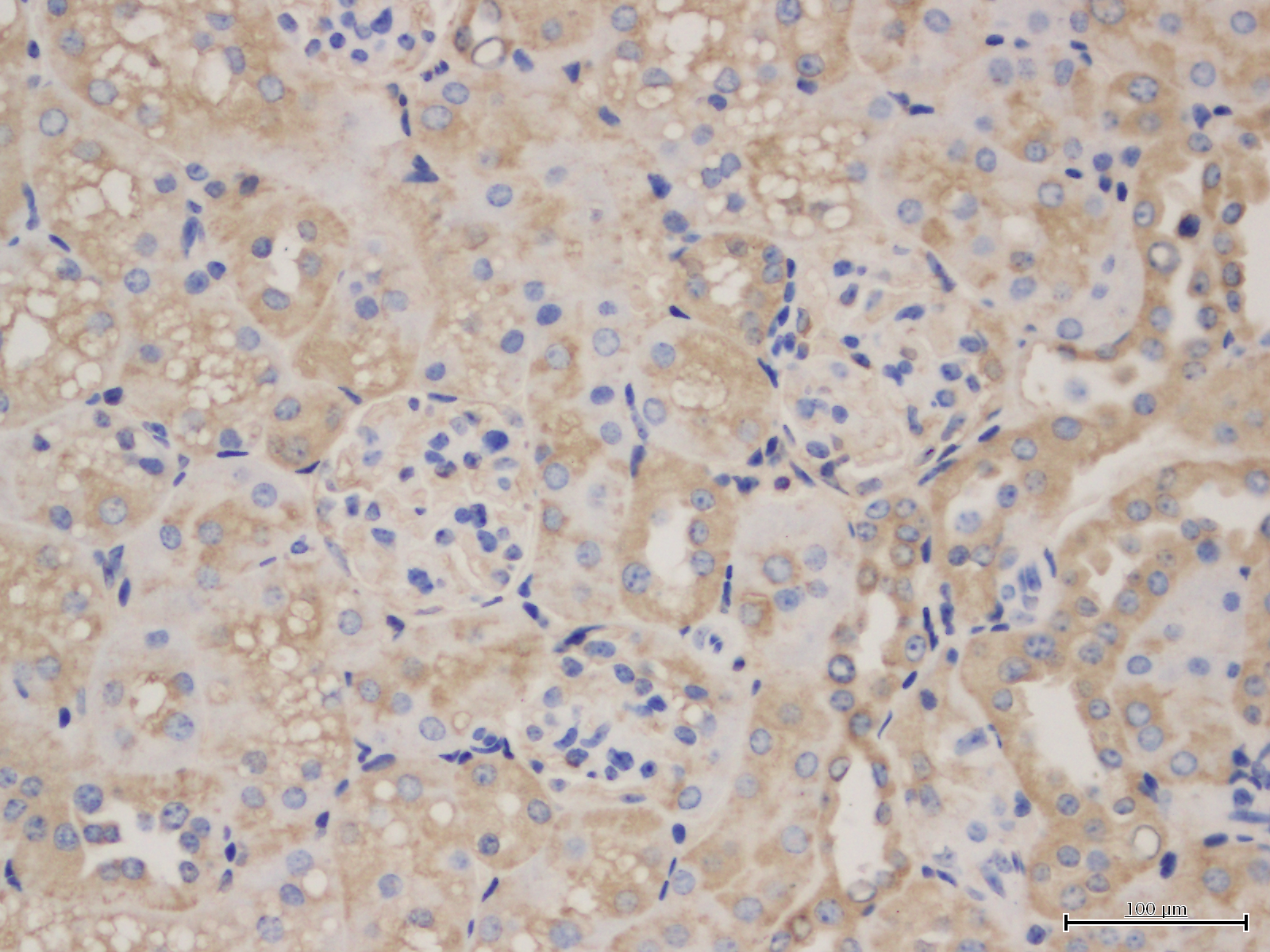

Supplement: S10 File — (ZIP) [file pone.0327042.s010.zip › 8w DM-4.TIF]

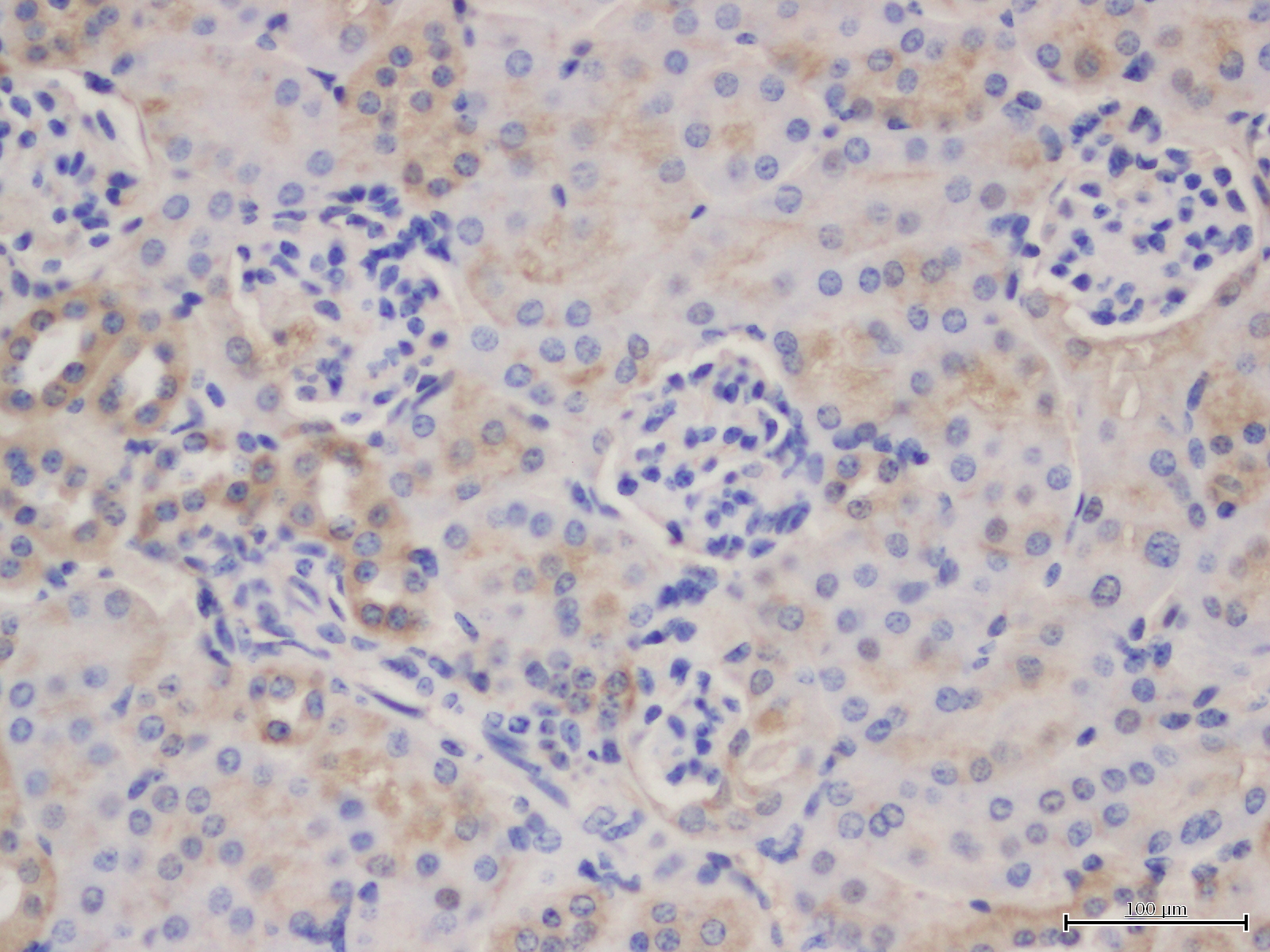

Supplement: S10 File — (ZIP) [file pone.0327042.s010.zip › 8w Con-2.TIF]

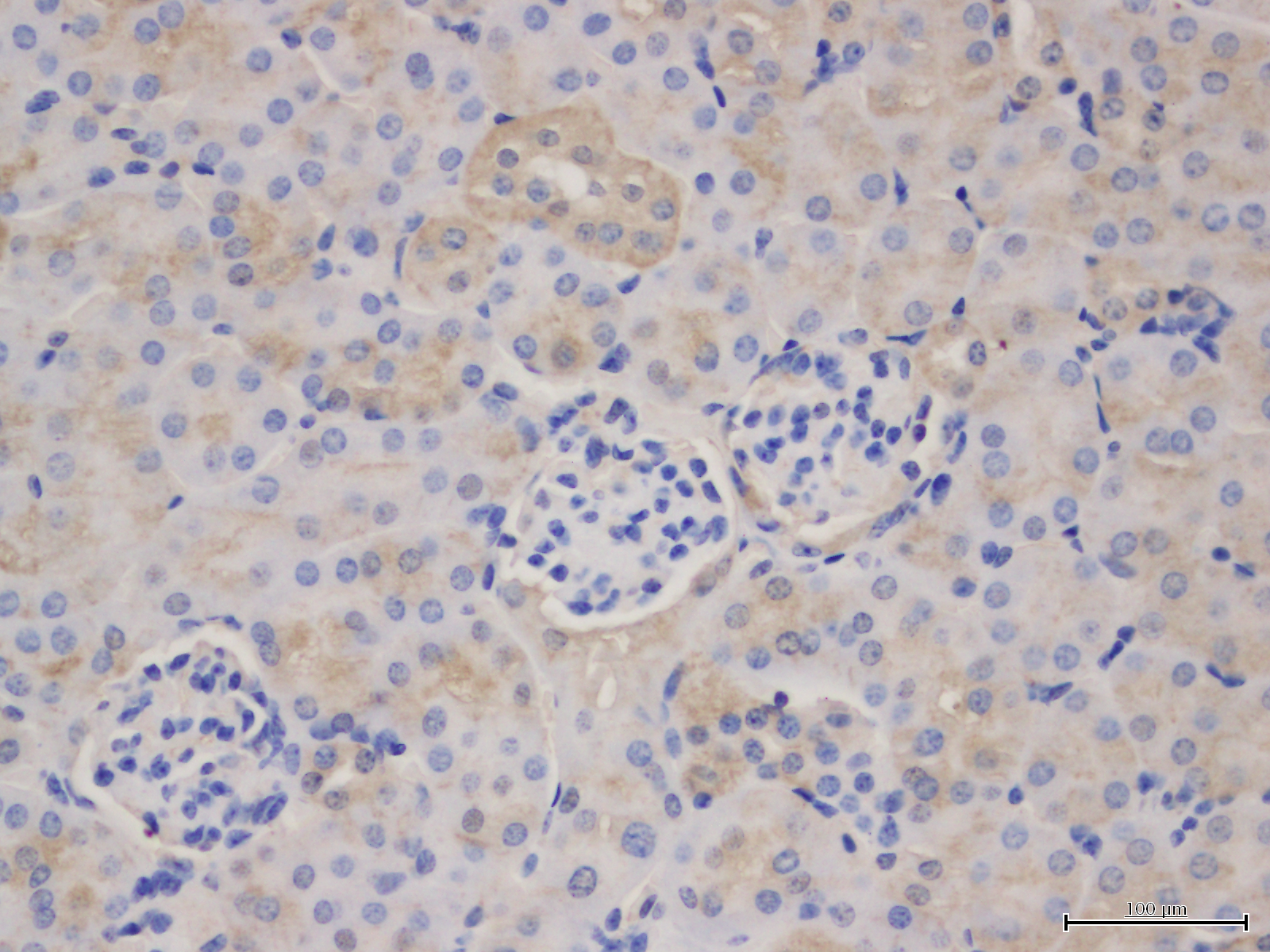

Supplement: S10 File — (ZIP) [file pone.0327042.s010.zip › 8w Con-2_3.TIF]

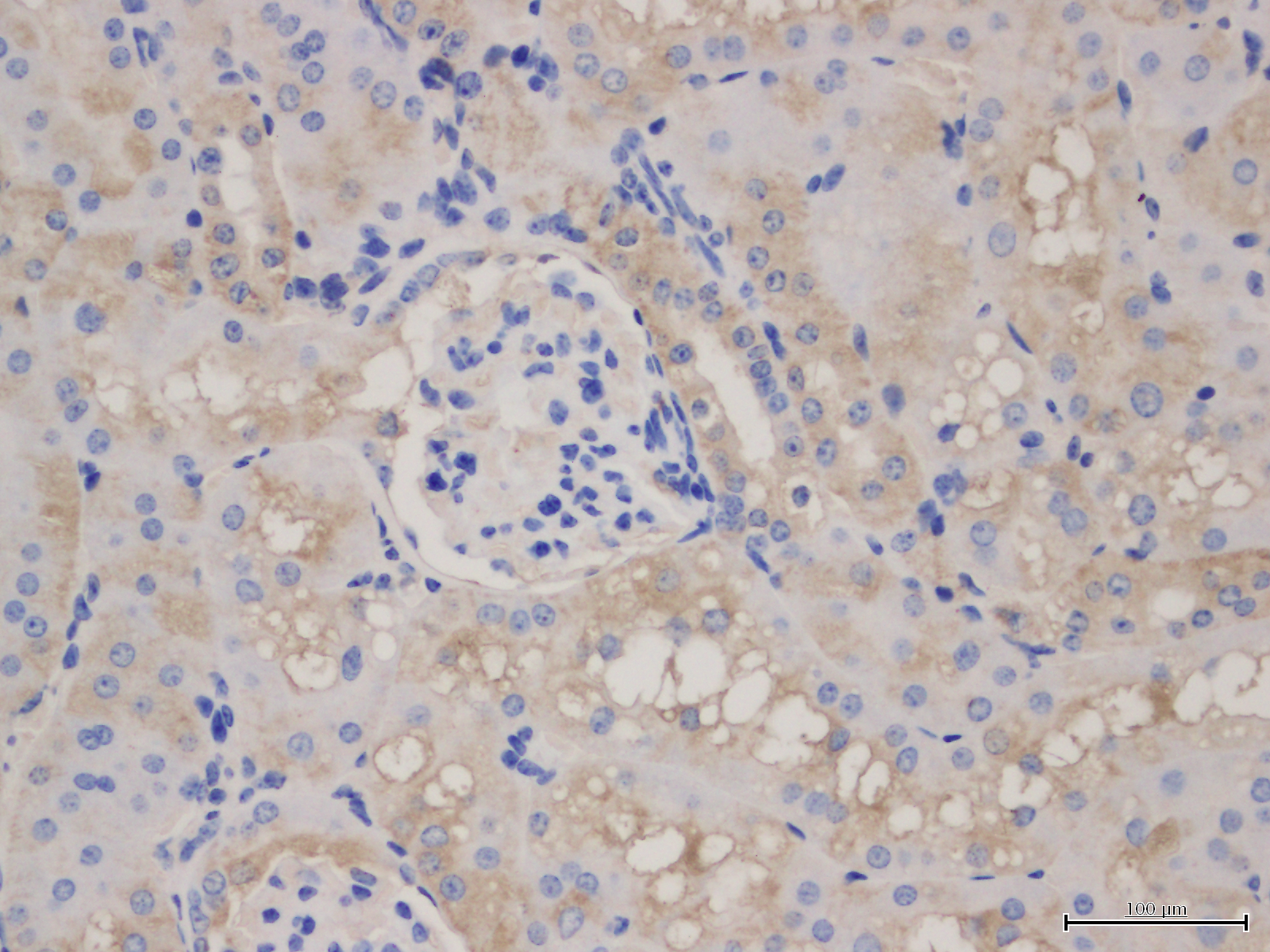

Supplement: S10 File — (ZIP) [file pone.0327042.s010.zip › 8w DM 25mGy-1(Used publication).TIF]

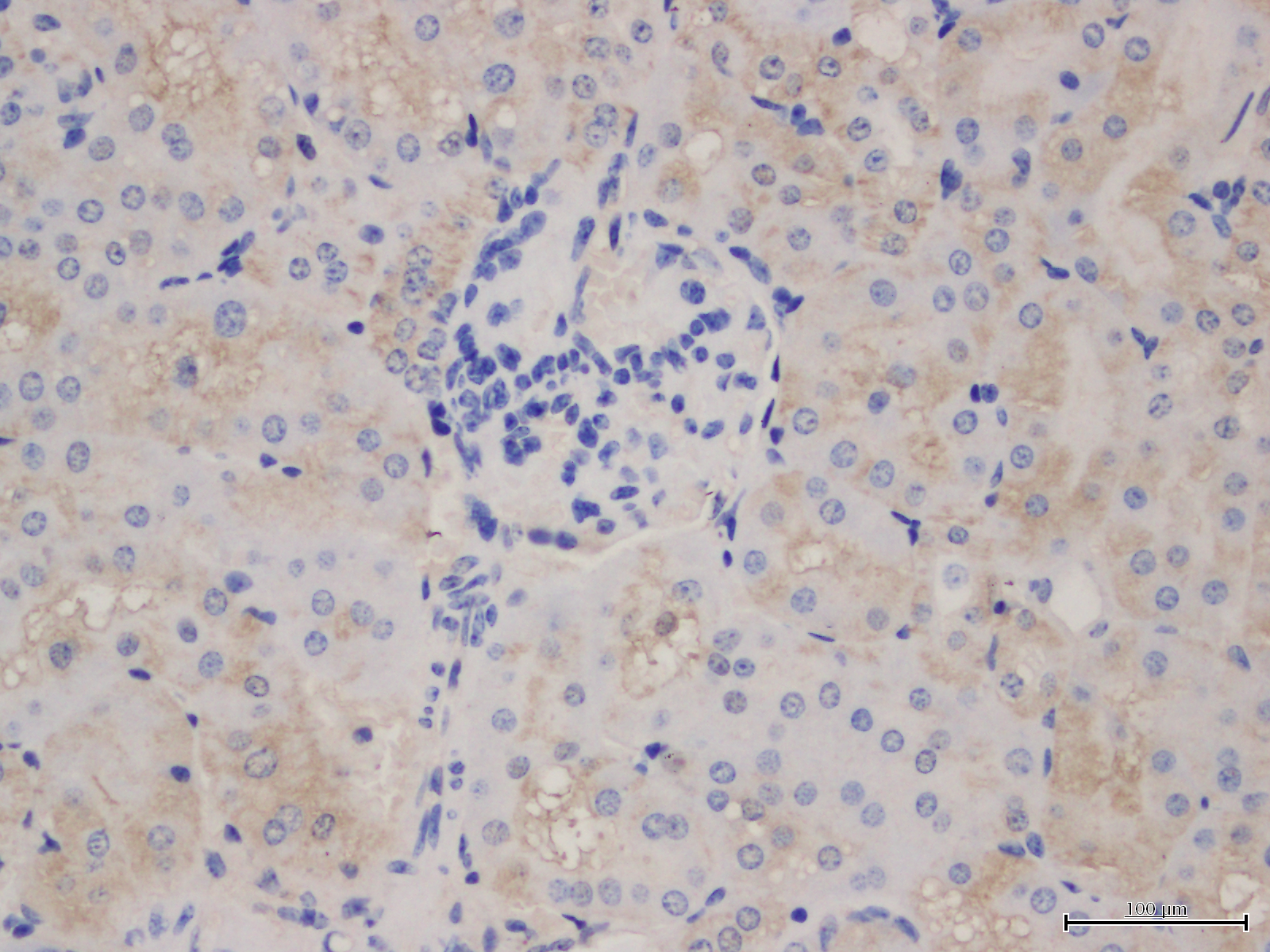

Supplement: S10 File — (ZIP) [file pone.0327042.s010.zip › 8w DM 25mGy-2.TIF]

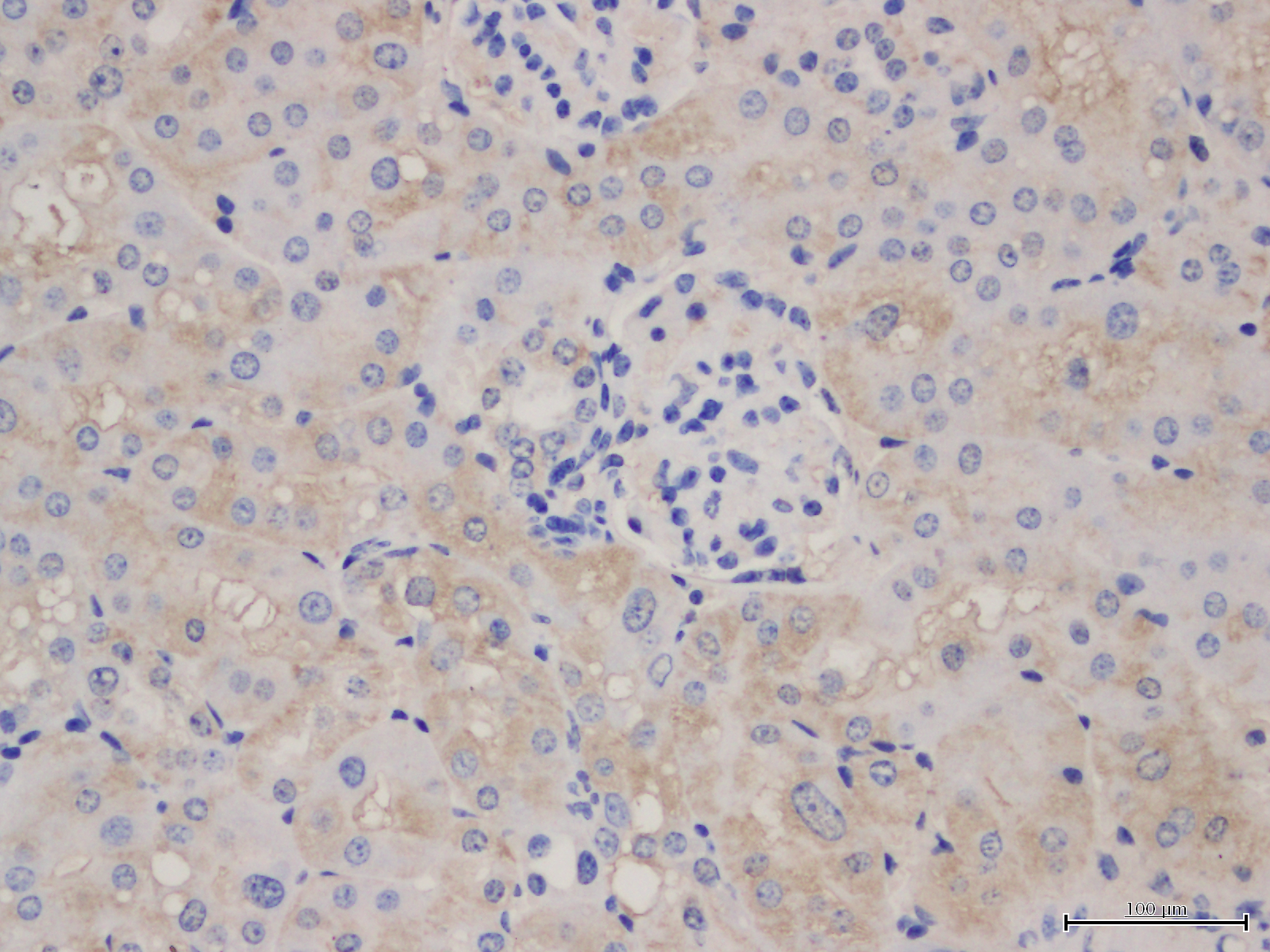

Supplement: S10 File — (ZIP) [file pone.0327042.s010.zip › 8w DM 25mGy-2_3.TIF]

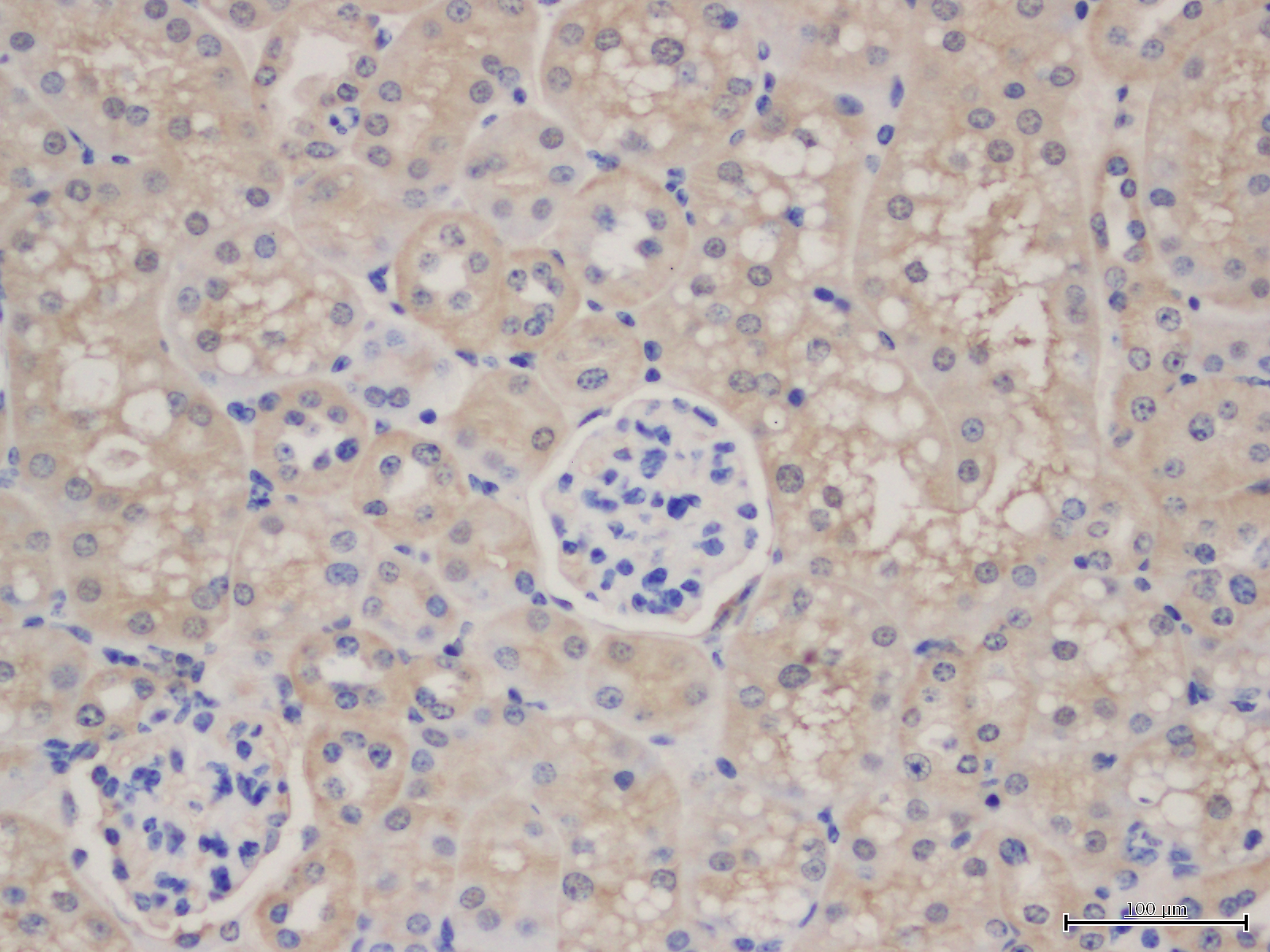

Supplement: S10 File — (ZIP) [file pone.0327042.s010.zip › 8w DM 50mGy-1(Used publication).TIF]

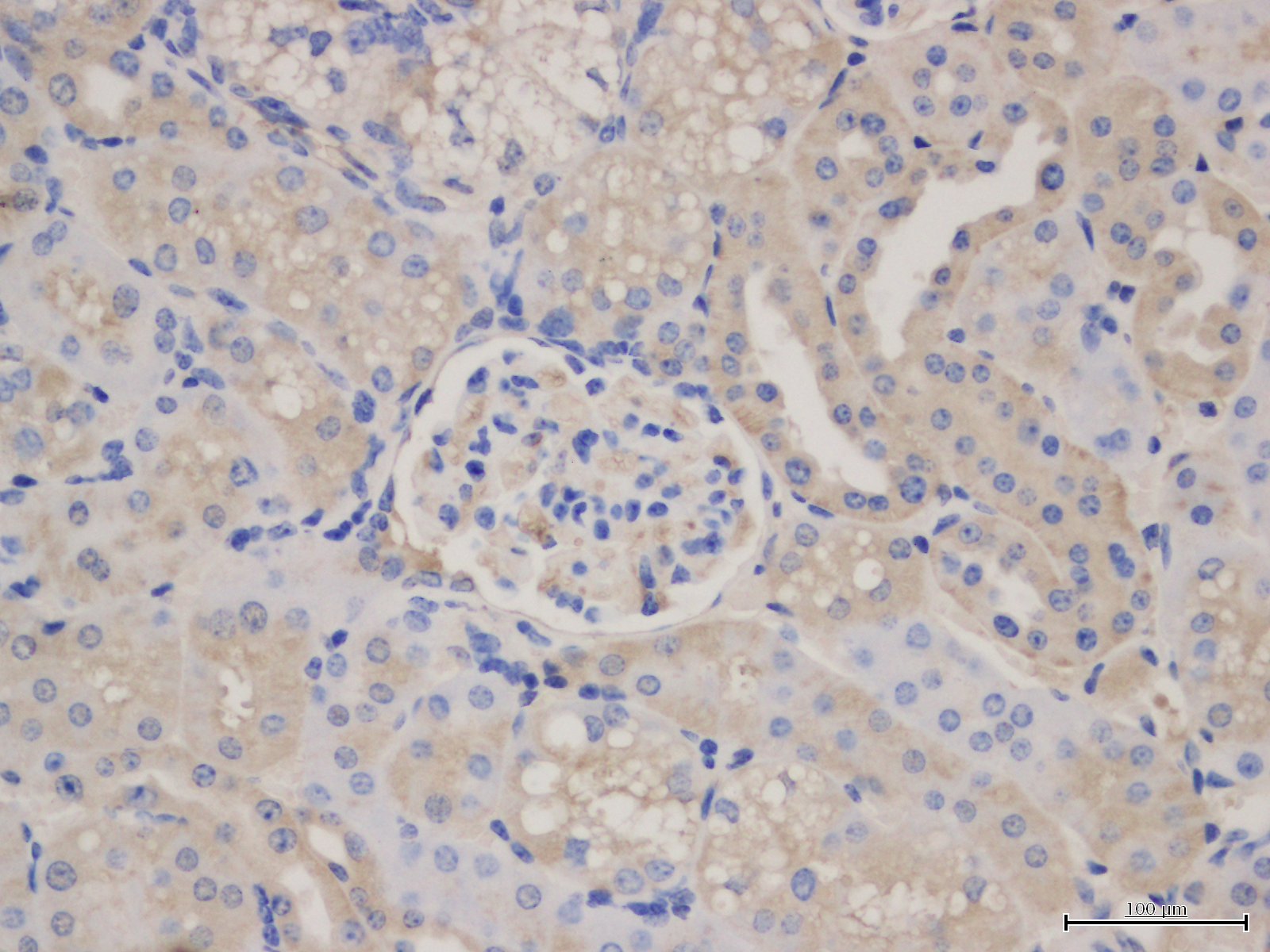

Supplement: S10 File — (ZIP) [file pone.0327042.s010.zip › 8w DM 50mGy-2.TIF]

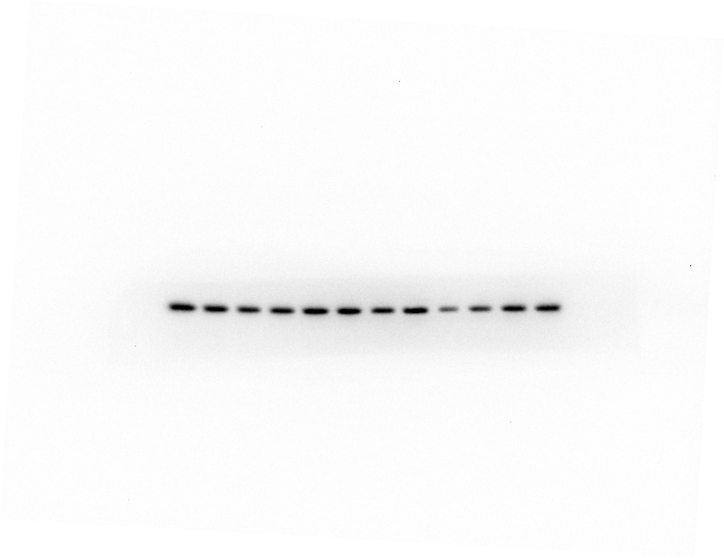

Supplement: S11 File — (ZIP) [file pone.0327042.s011.zip › Fig 7B_Nrf-2 blot.tif]

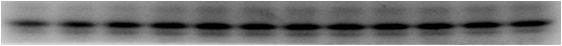

Supplement: S11 File — (ZIP) [file pone.0327042.s011.zip › actin nrf2 .tif]

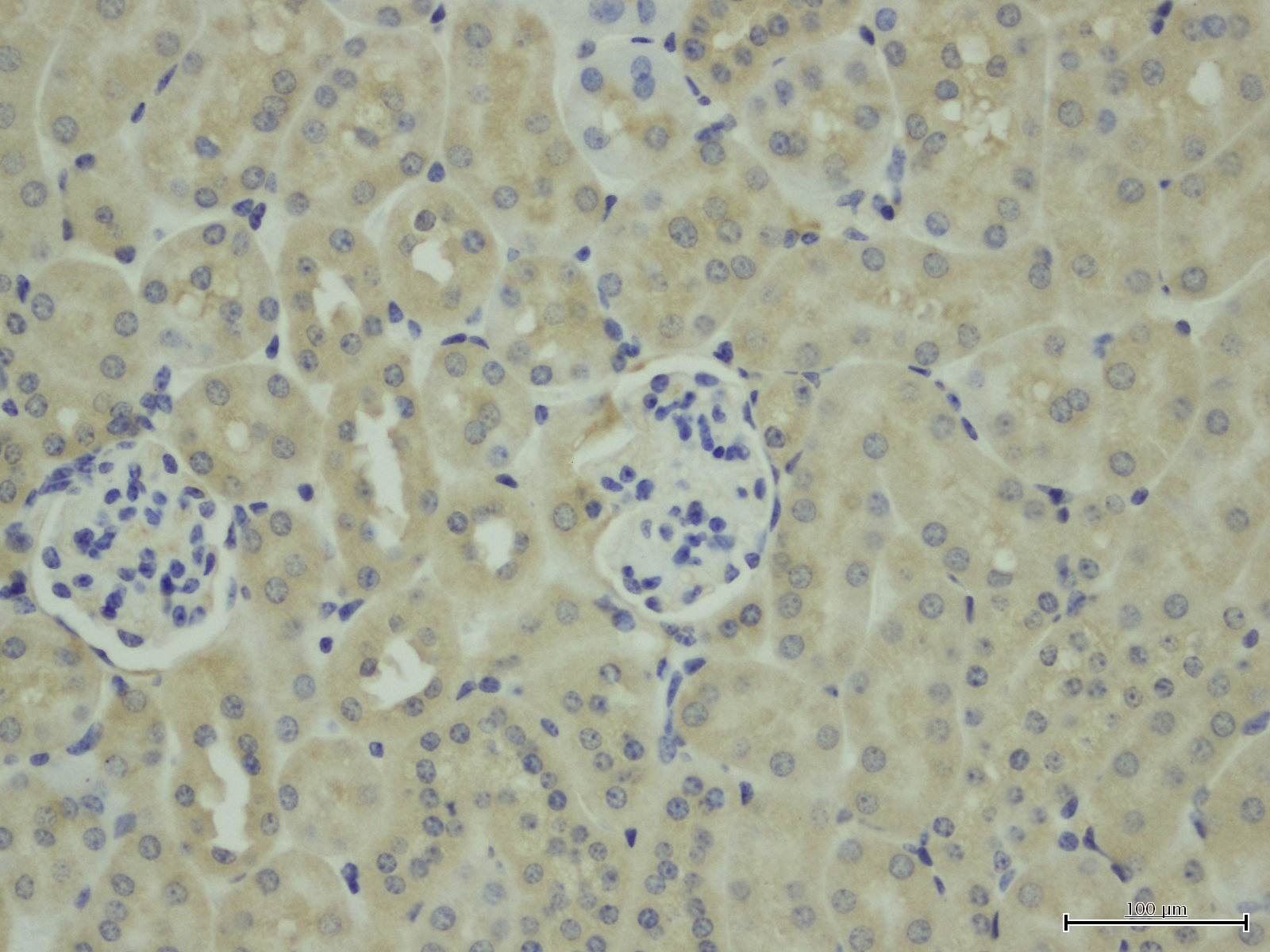

Supplement: S12 File — (ZIP) [file pone.0327042.s012.zip › Con 4w-1(Used publication).tif]

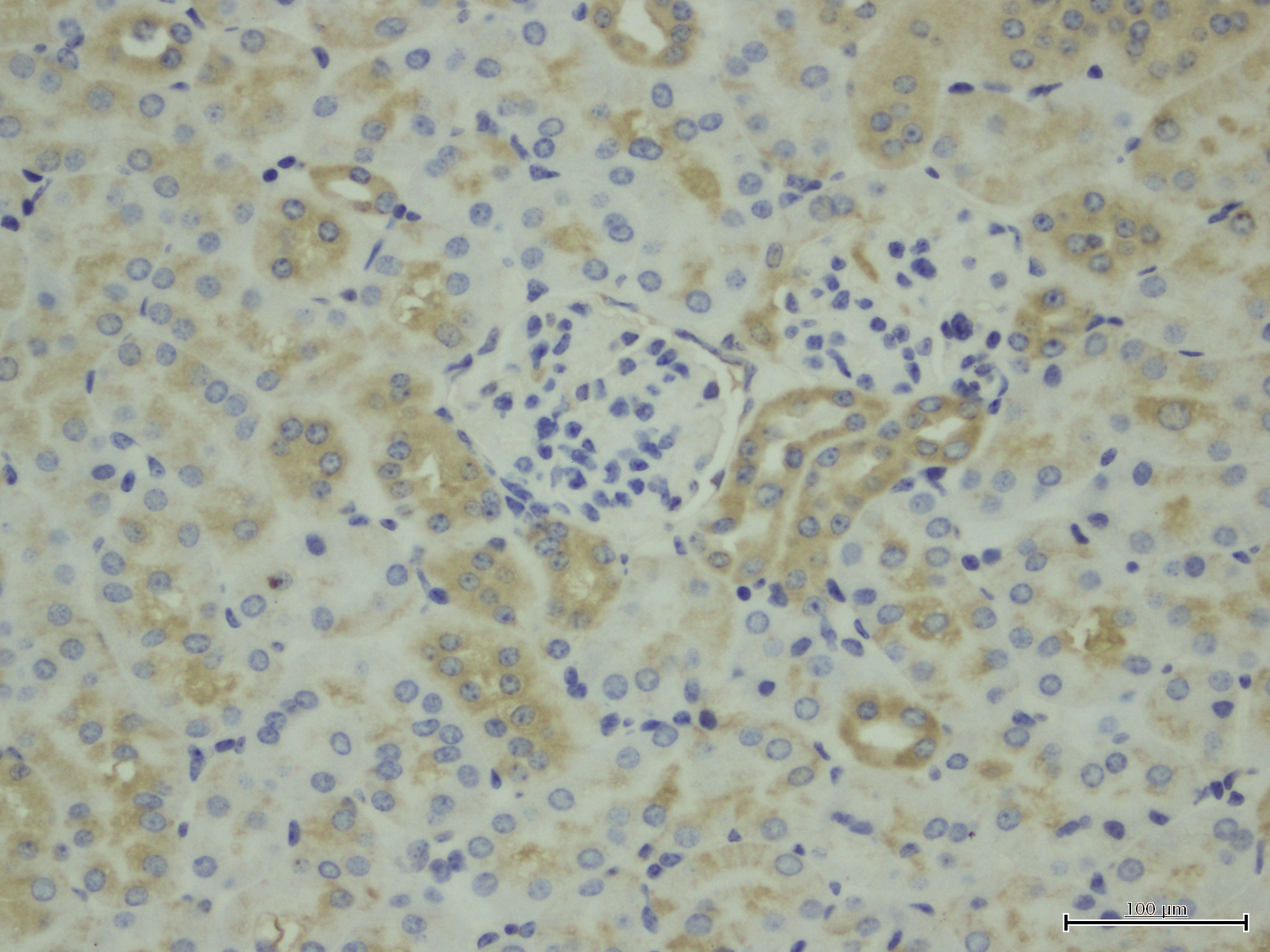

Supplement: S12 File — (ZIP) [file pone.0327042.s012.zip › Con 4w-2_1.tif]

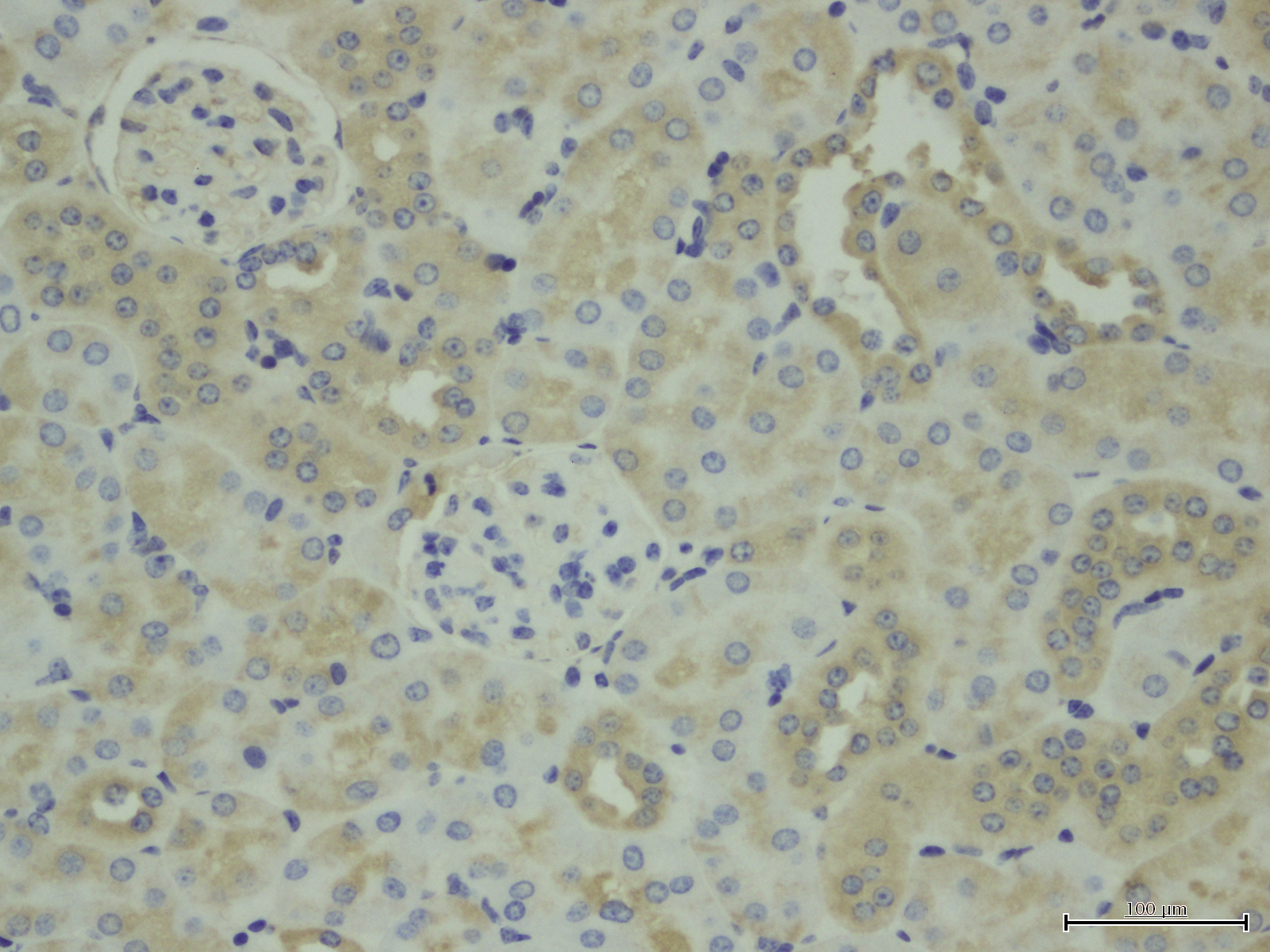

Supplement: S12 File — (ZIP) [file pone.0327042.s012.zip › Con 4w-3.tif]

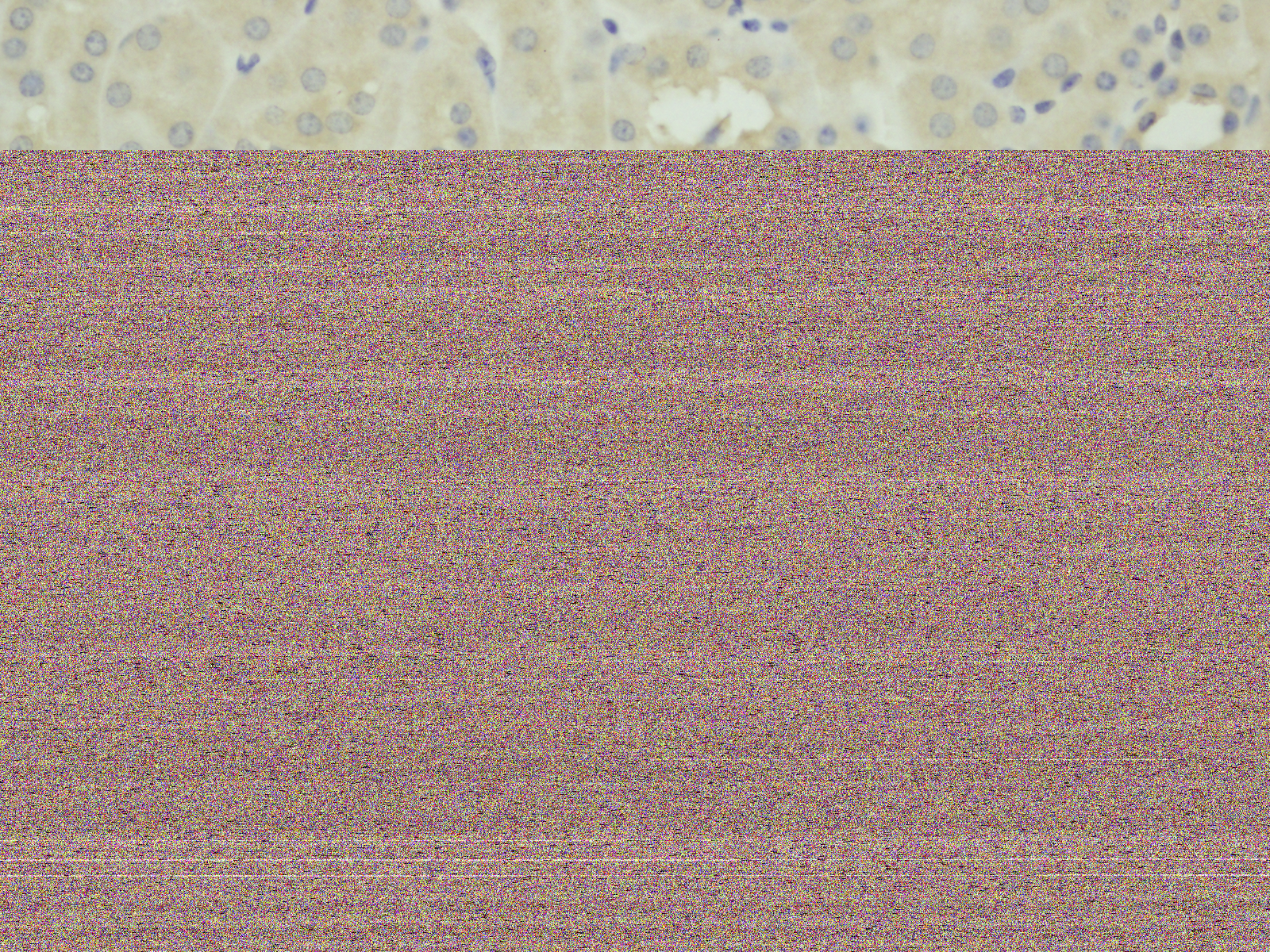

Supplement: S12 File — (ZIP) [file pone.0327042.s012.zip › Con 8w-1_1(Used publication).tif]

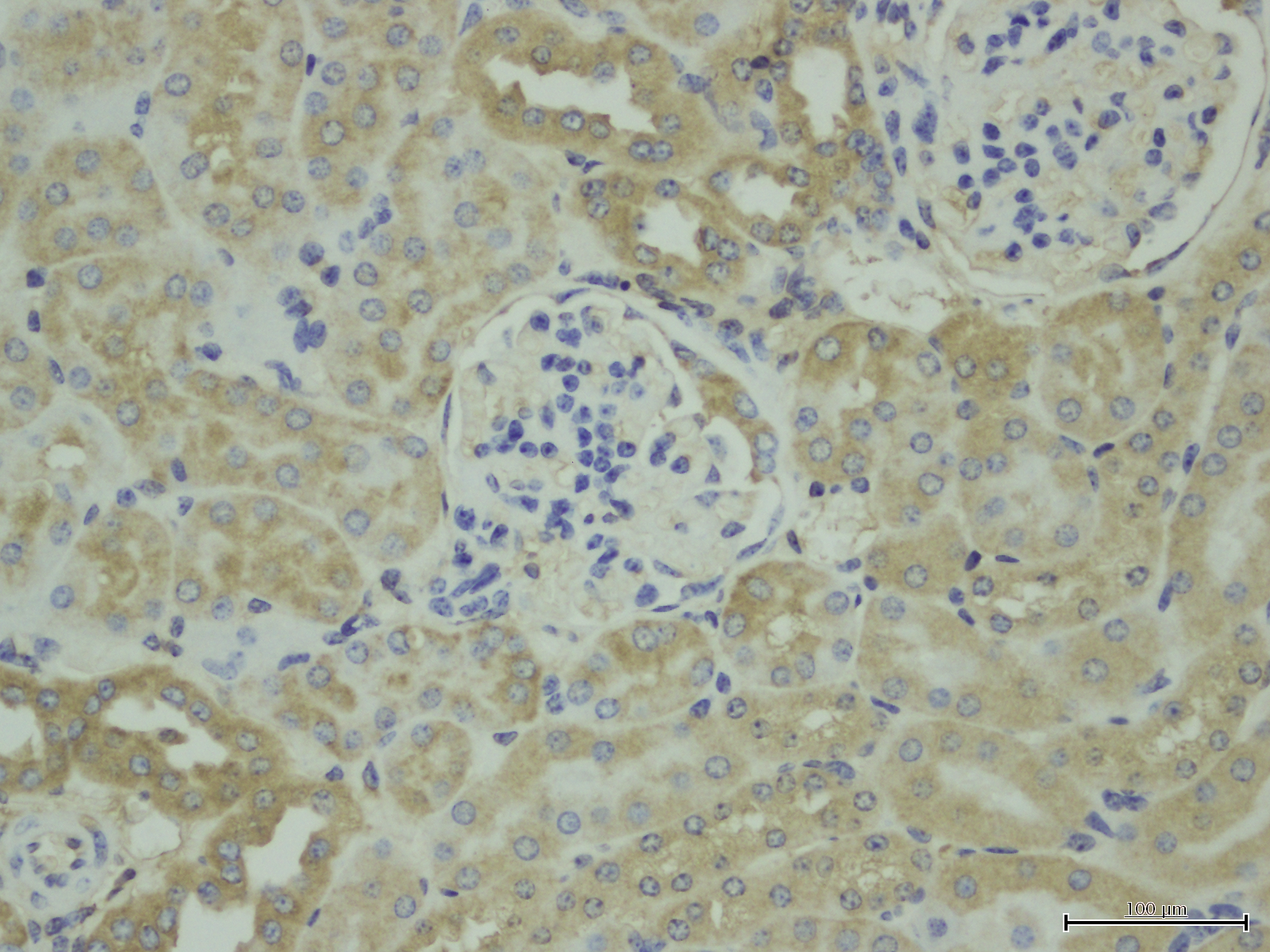

Supplement: S12 File — (ZIP) [file pone.0327042.s012.zip › con 50mGy 4w-1 (Used publication).tif]

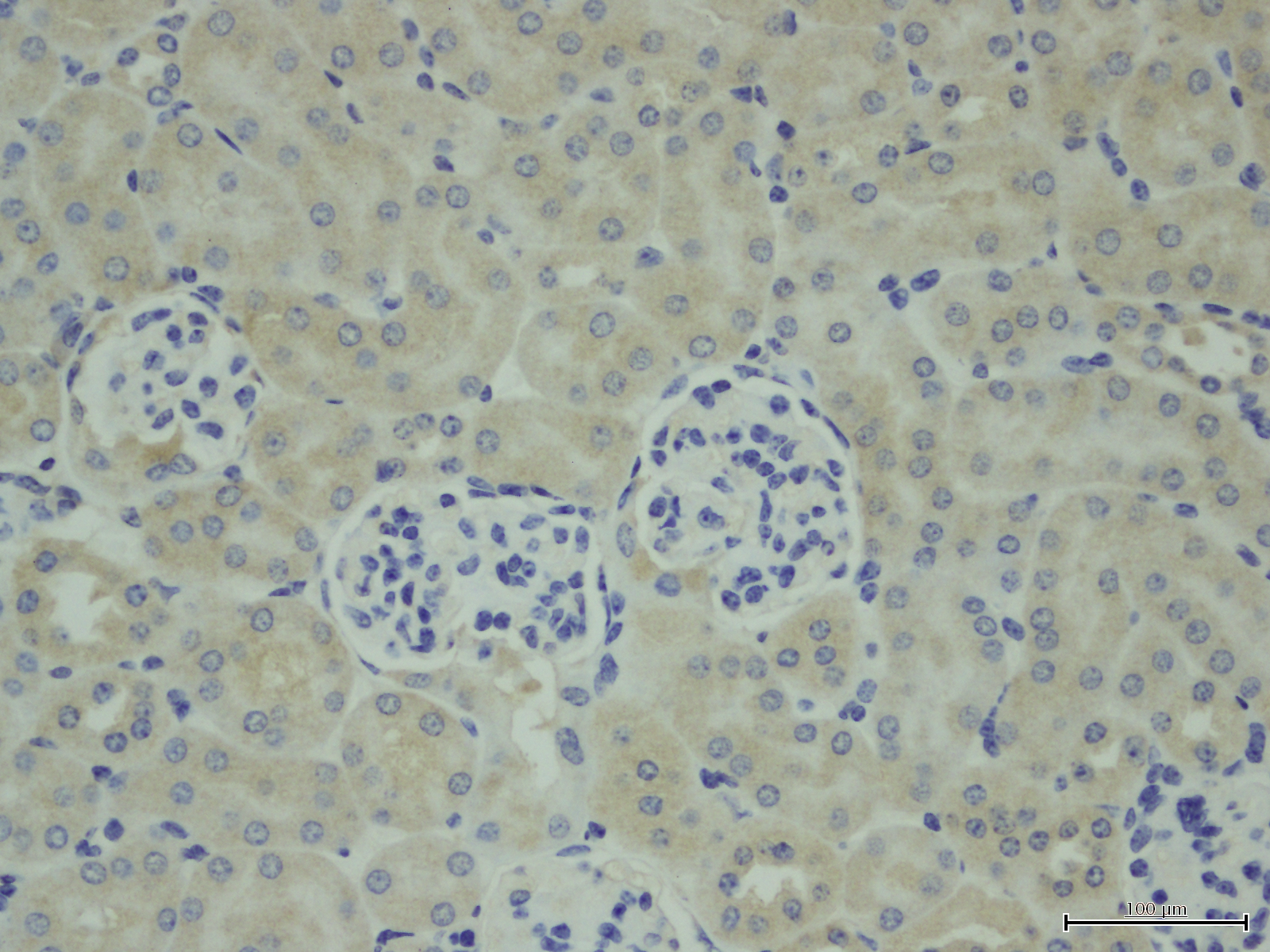

Supplement: S12 File — (ZIP) [file pone.0327042.s012.zip › Con 50mGy 4w-2.tif]

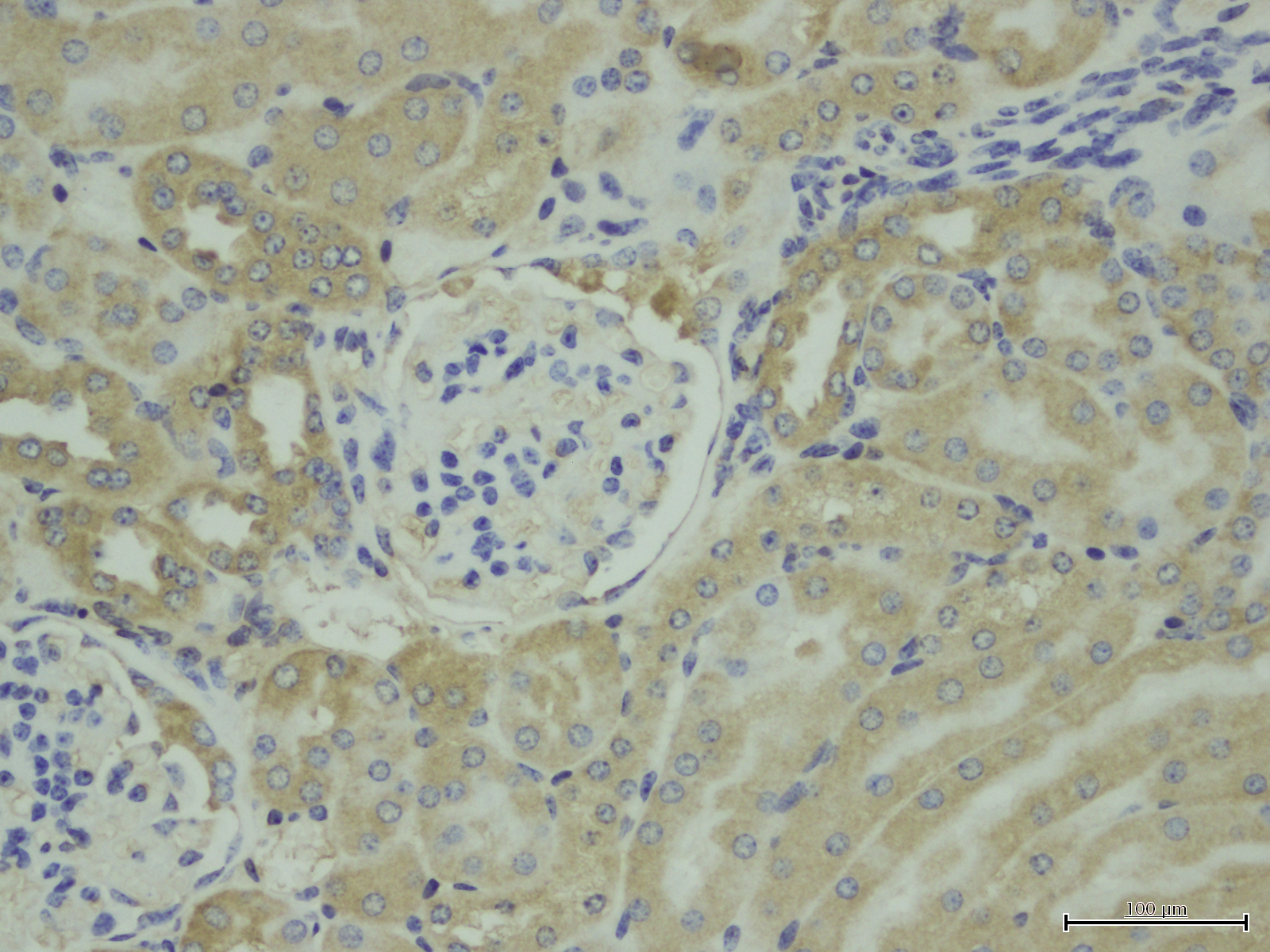

Supplement: S12 File — (ZIP) [file pone.0327042.s012.zip › Con 50mGy 4w-3 ( incorrectly used for Con 50mGy 8w group).tif]

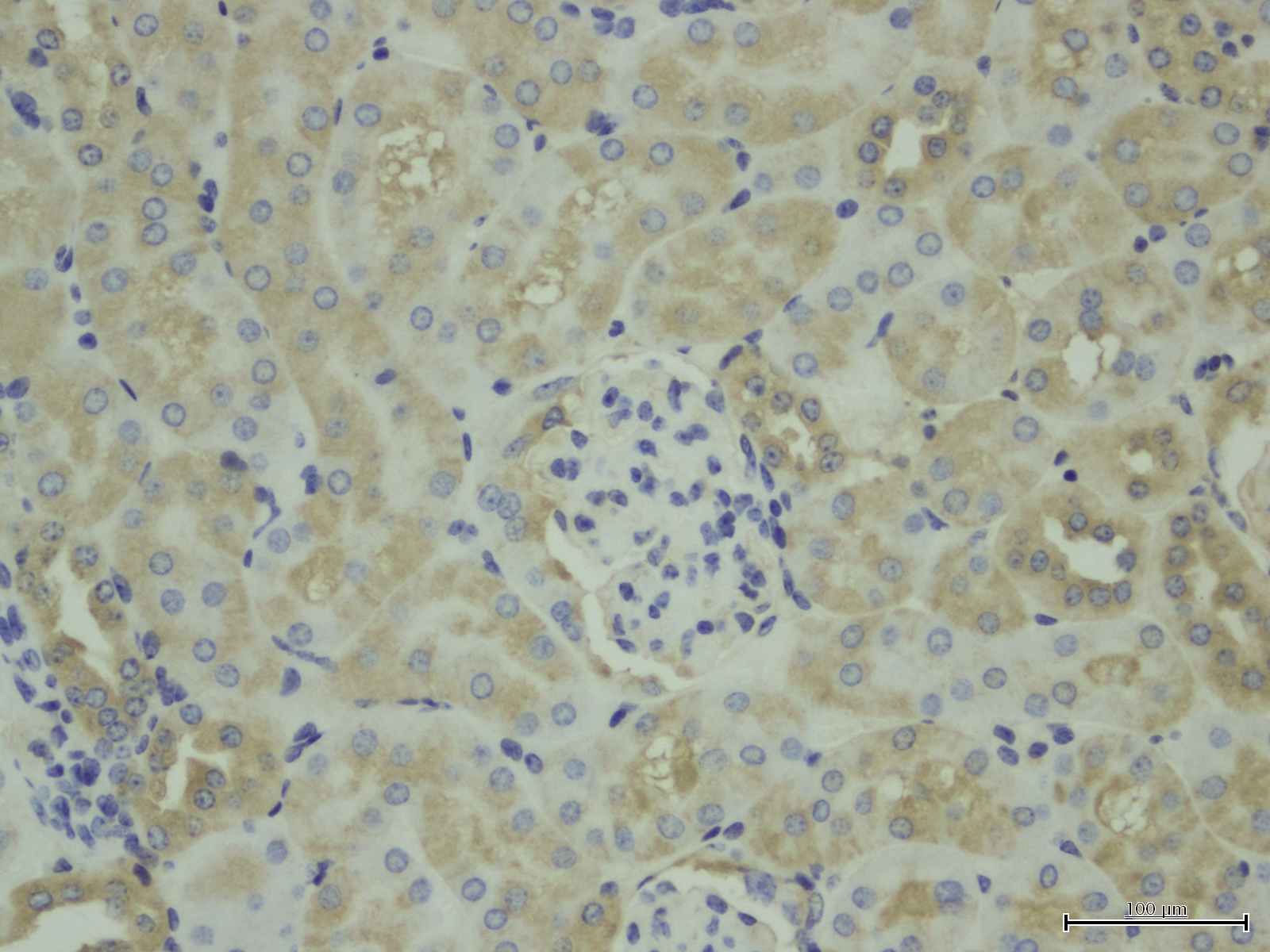

Supplement: S12 File — (ZIP) [file pone.0327042.s012.zip › Con 50mGy 8w -1(Used publication).tif]

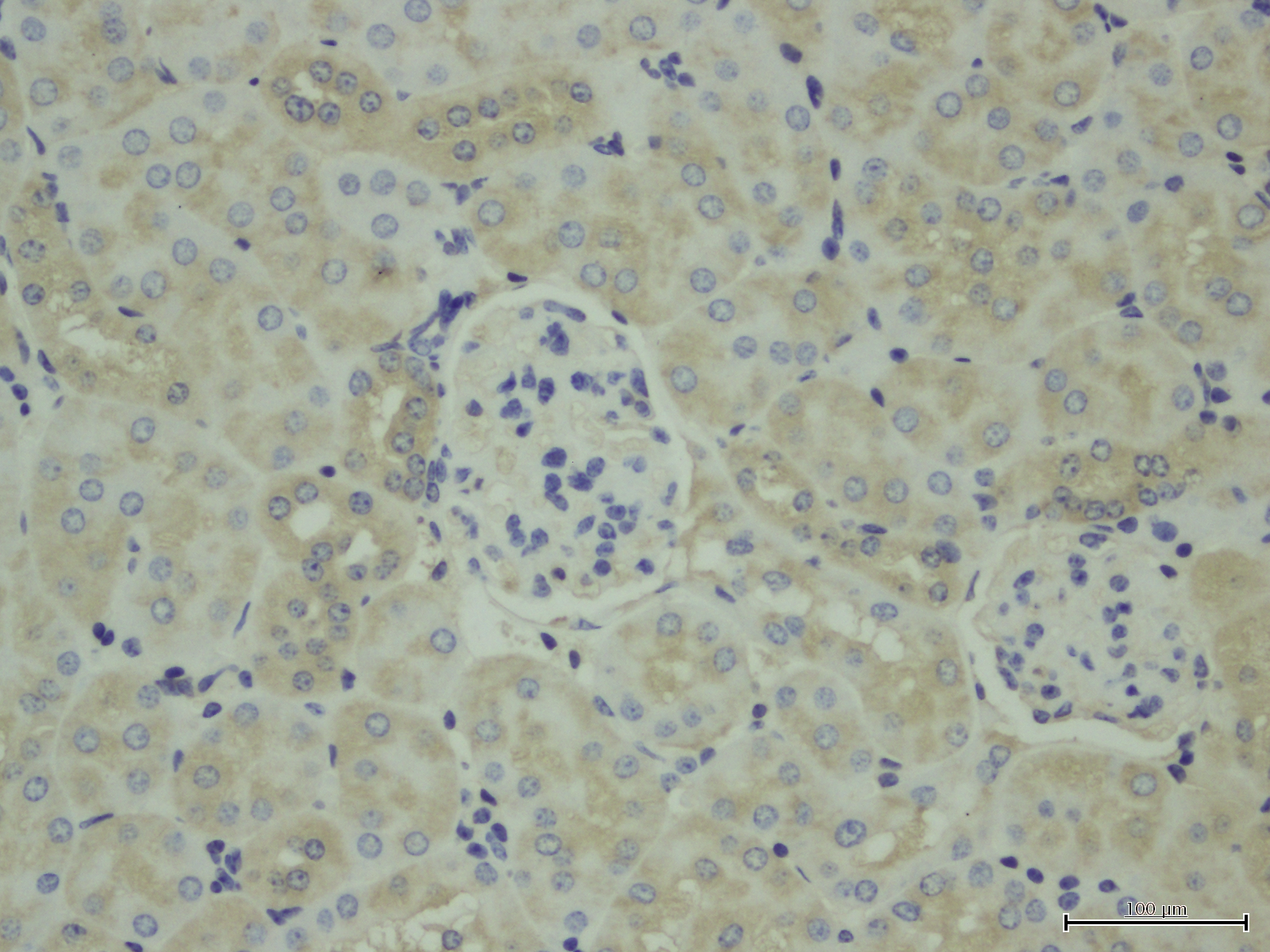

Supplement: S12 File — (ZIP) [file pone.0327042.s012.zip › Con 50mGy 8w -2.tif]

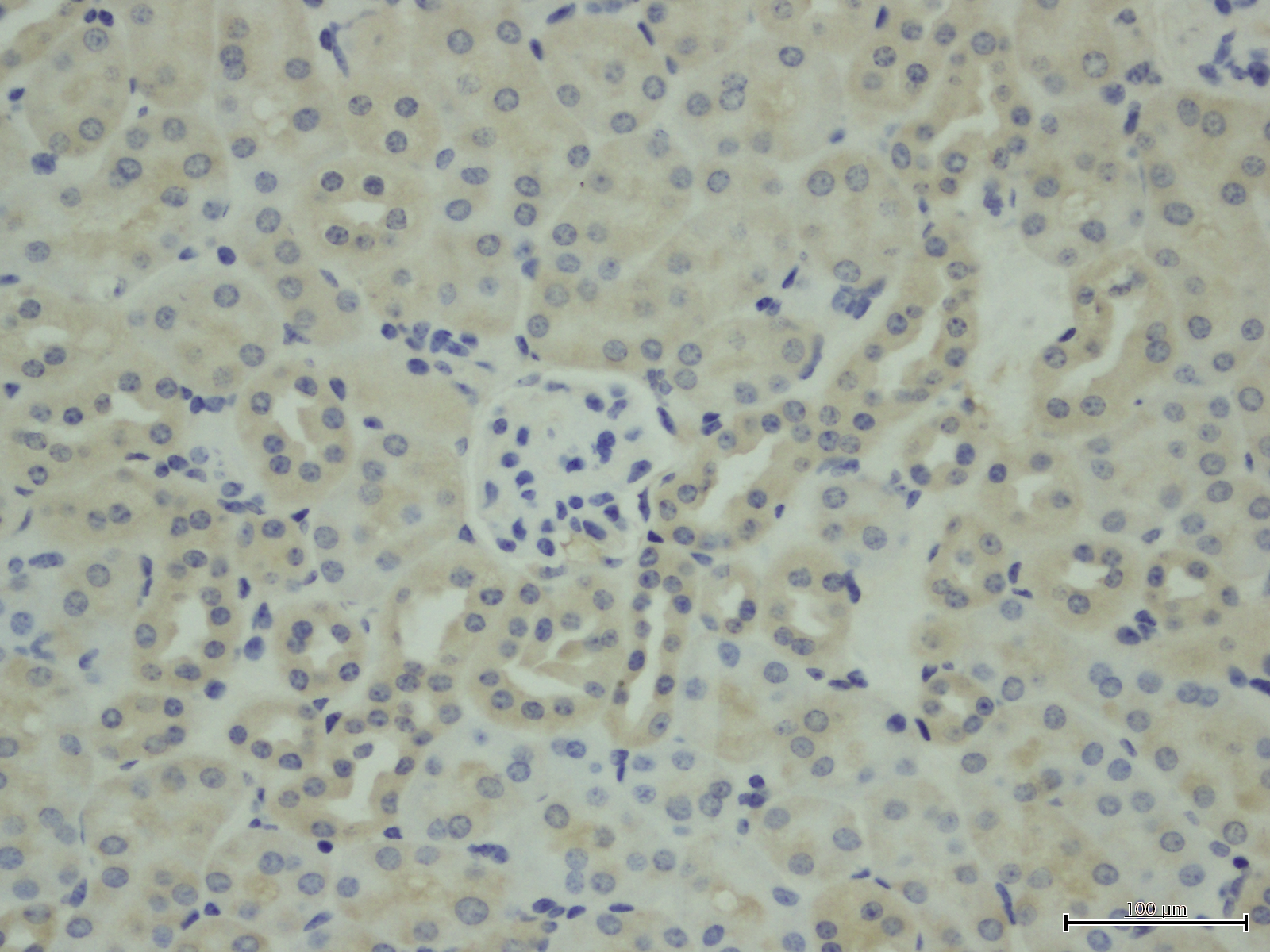

Supplement: S12 File — (ZIP) [file pone.0327042.s012.zip › Con 50mGy 8w -3.tif]

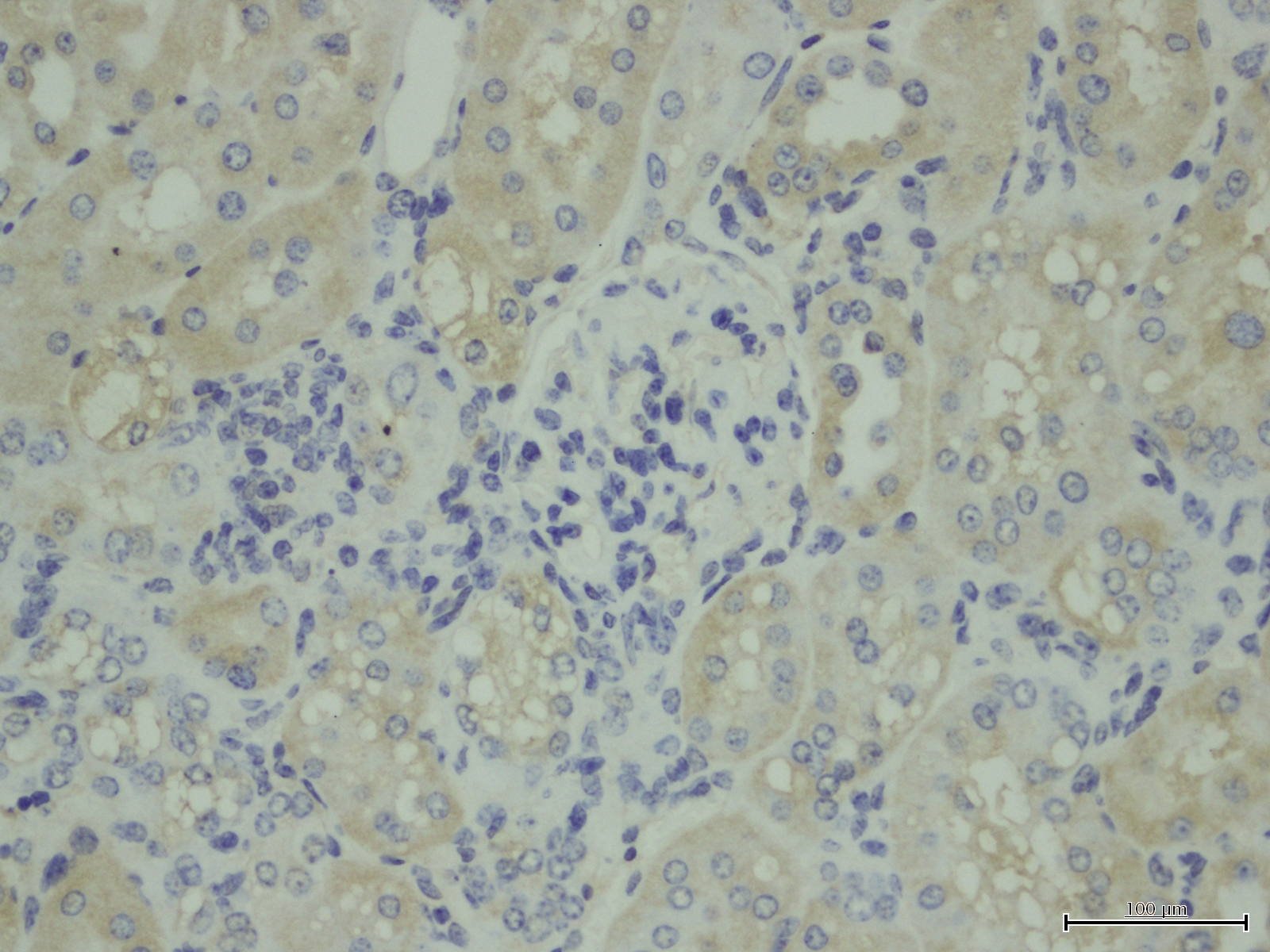

Supplement: S12 File — (ZIP) [file pone.0327042.s012.zip › DM 4w-1 (Used publication).tif]

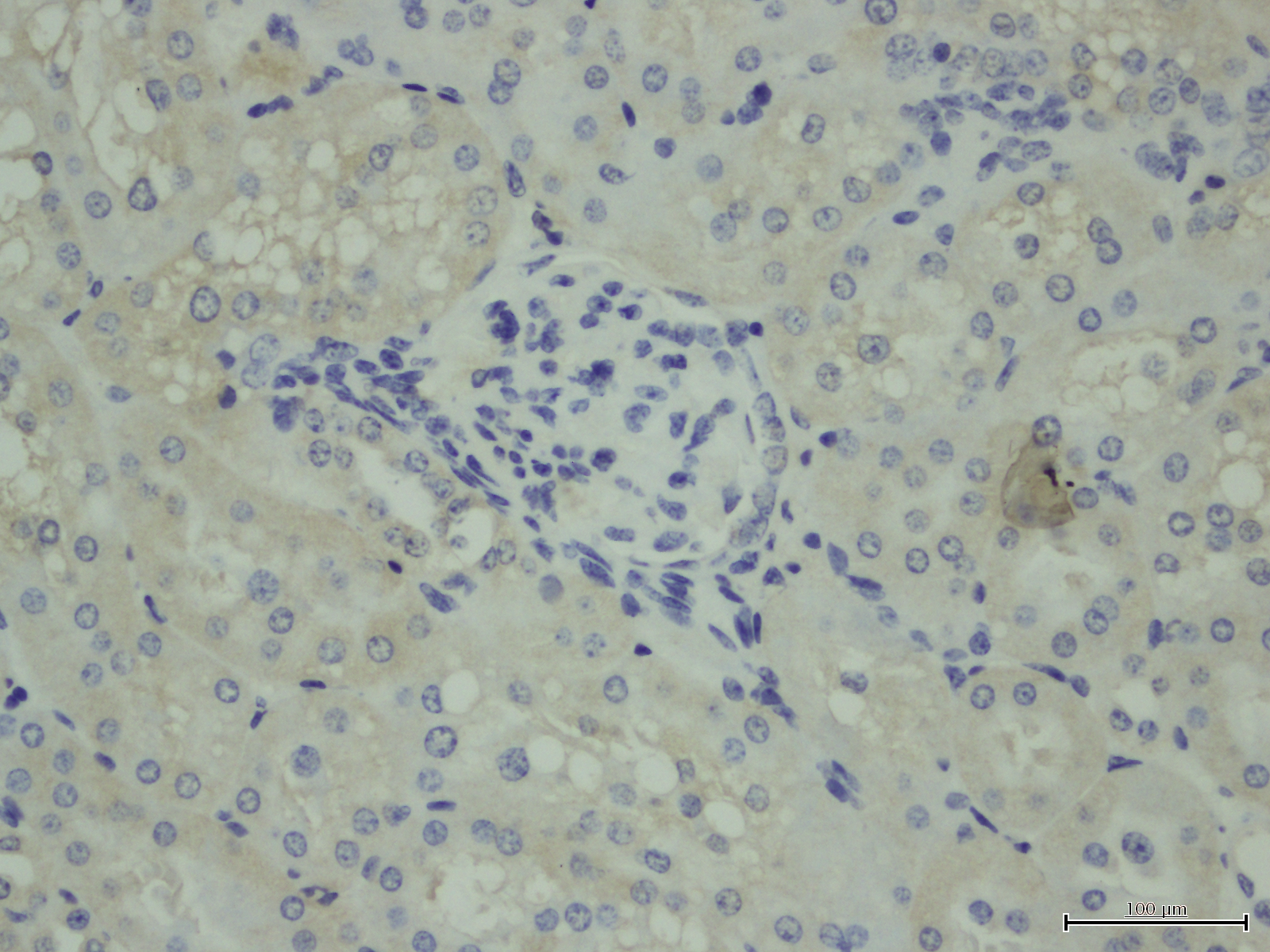

Supplement: S12 File — (ZIP) [file pone.0327042.s012.zip › DM 4w-2.tif]

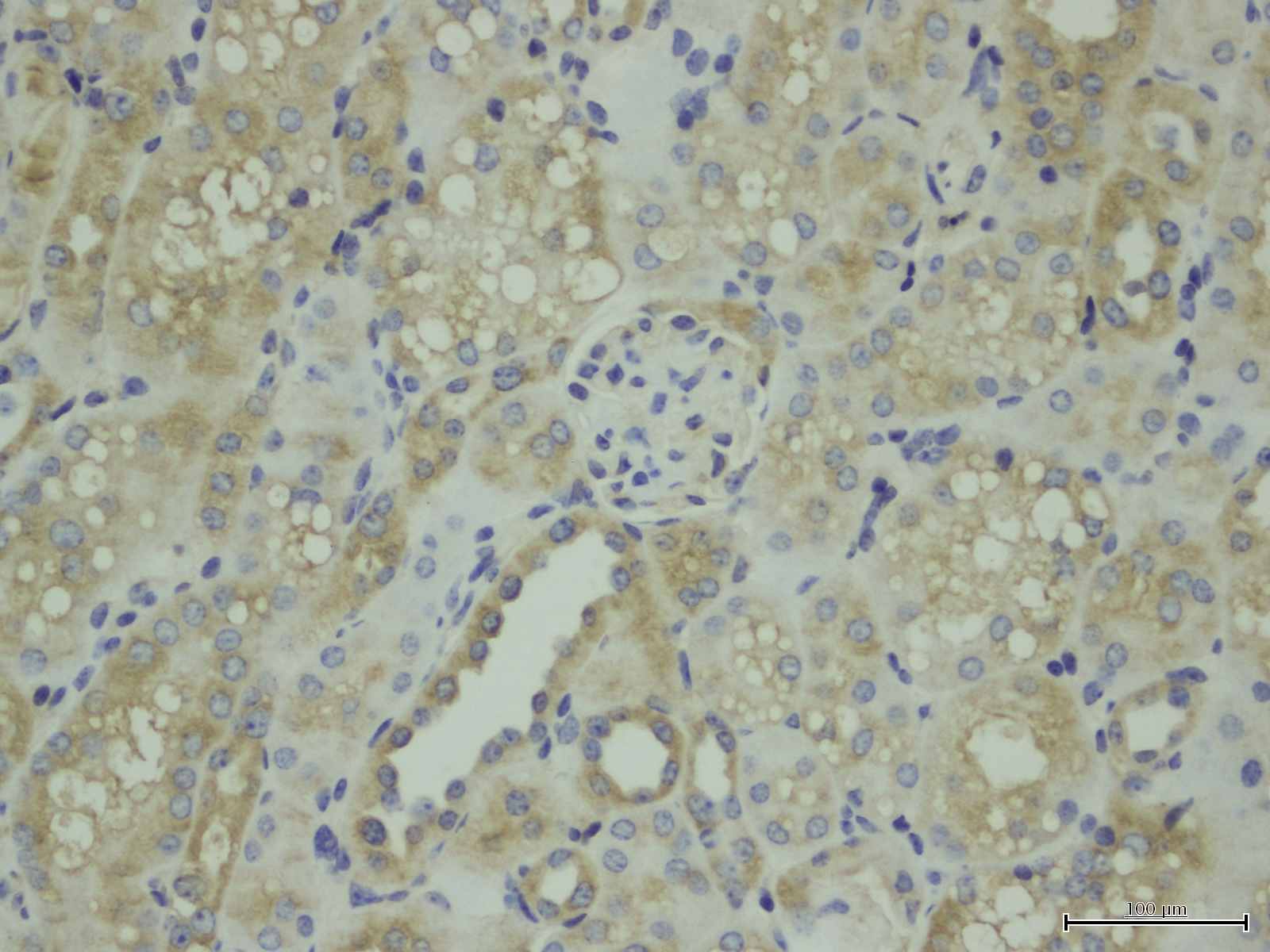

Supplement: S12 File — (ZIP) [file pone.0327042.s012.zip › DM 4w-3.tif]

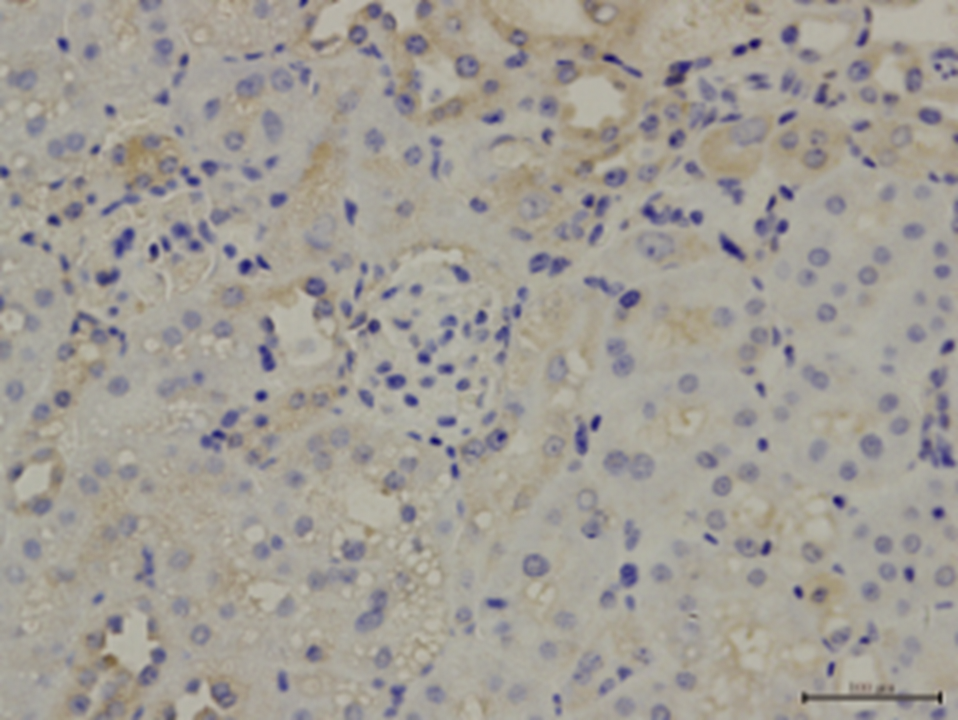

Supplement: S12 File — (ZIP) [file pone.0327042.s012.zip › DM 8w-1(Used publication).tif]

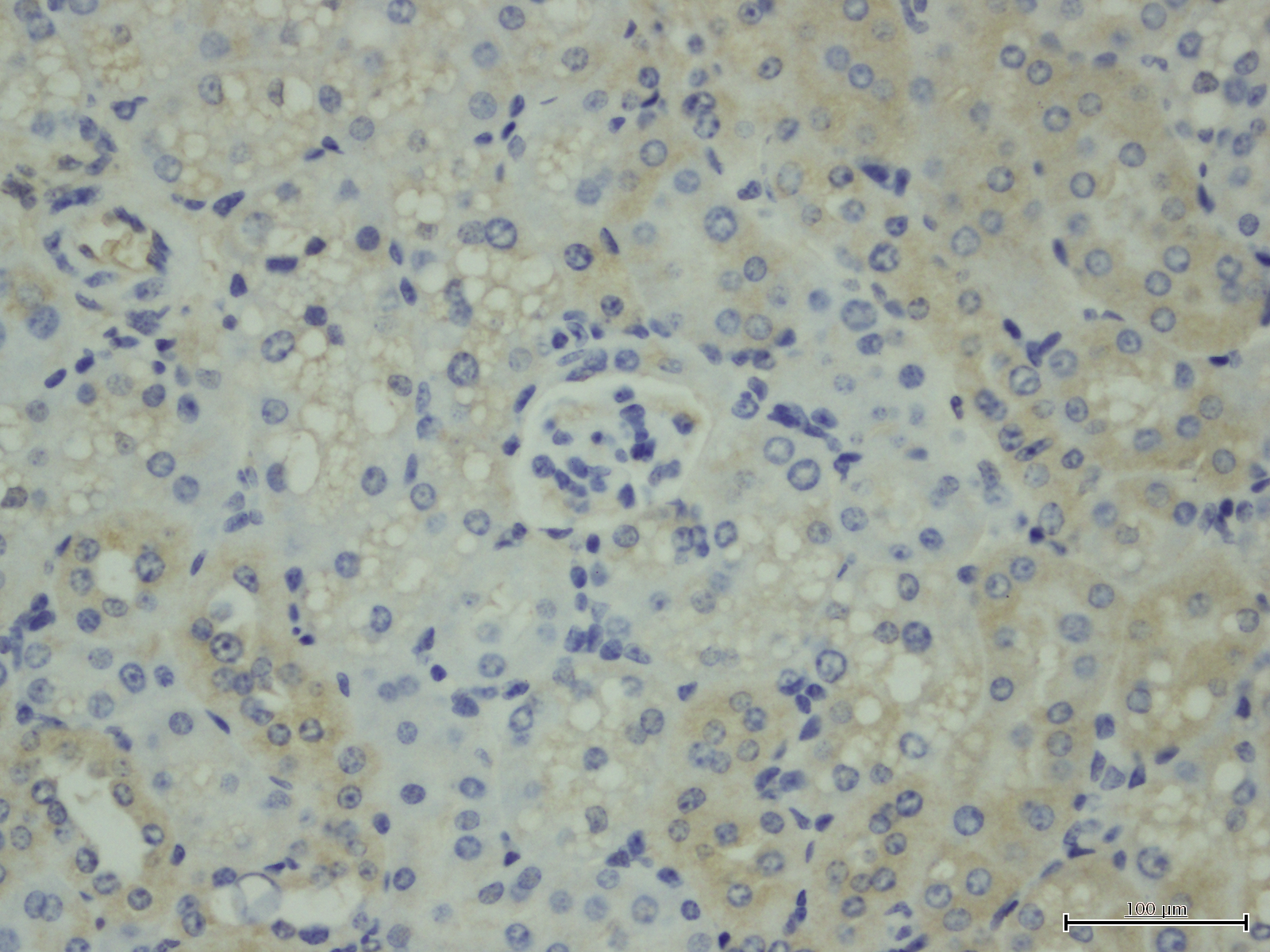

Supplement: S12 File — (ZIP) [file pone.0327042.s012.zip › DM 8w-2.tif]

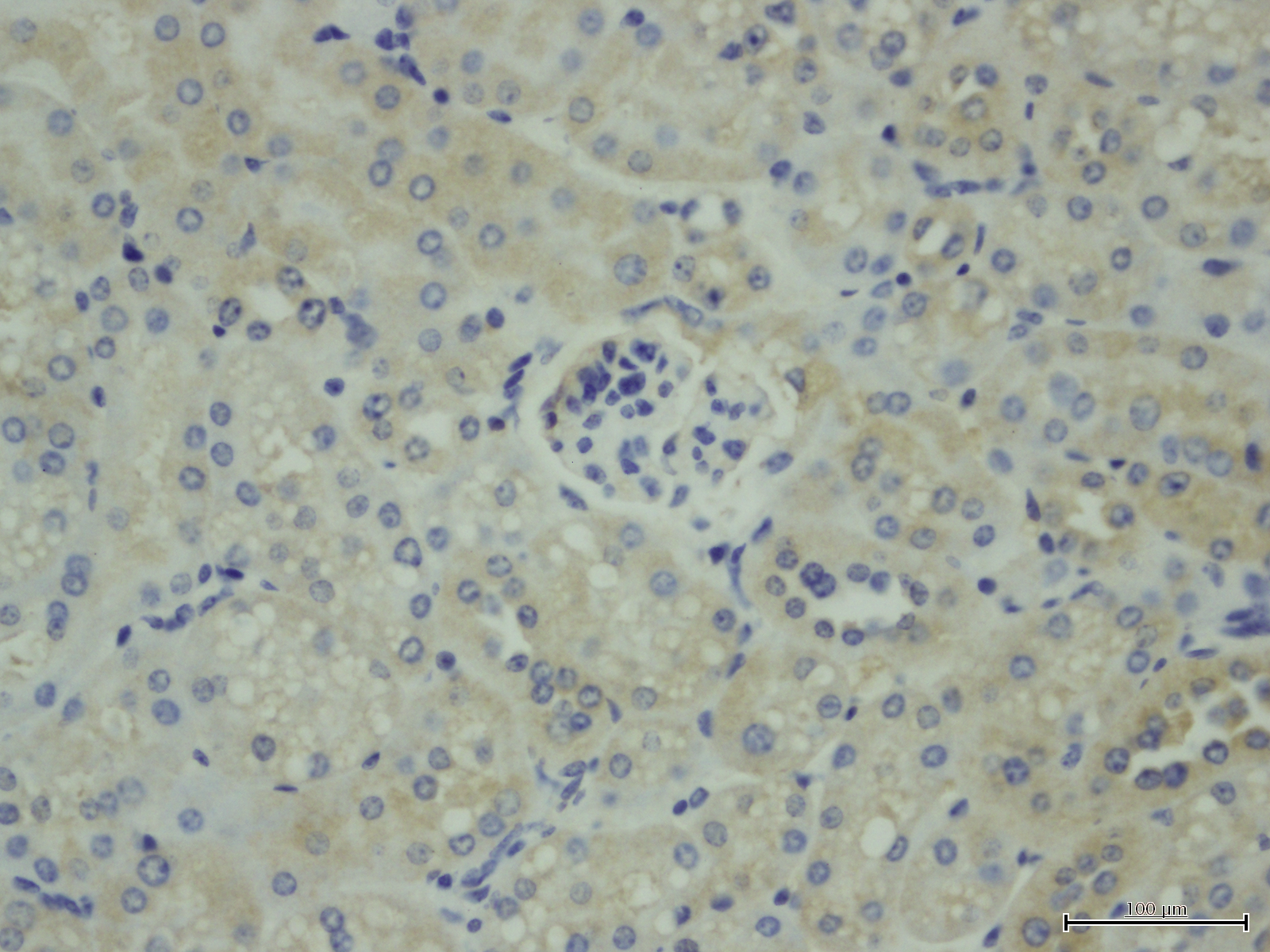

Supplement: S12 File — (ZIP) [file pone.0327042.s012.zip › DM 8w-3.tif]

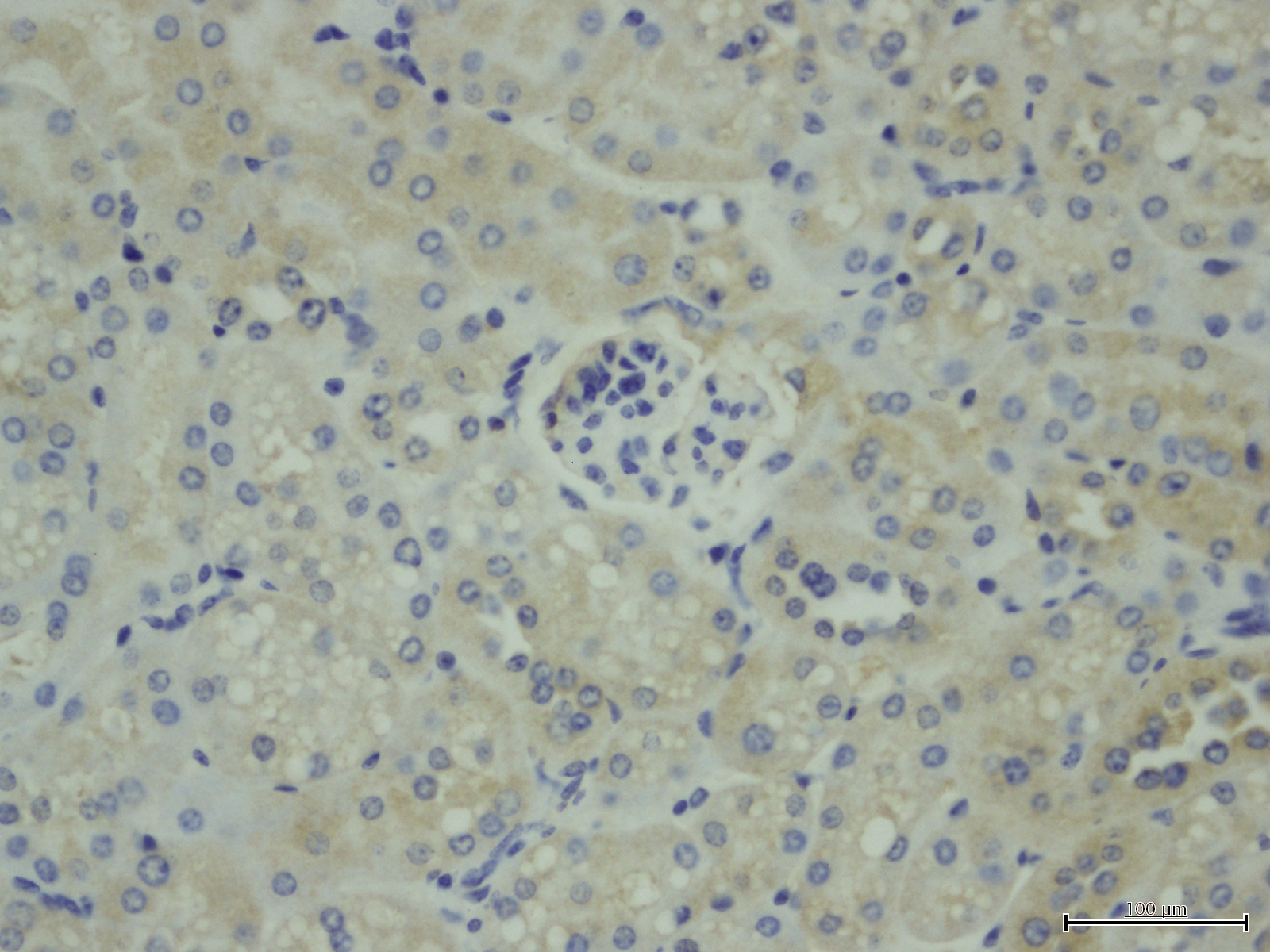

Supplement: S12 File — (ZIP) [file pone.0327042.s012.zip › DM 8w-3_1.tif]

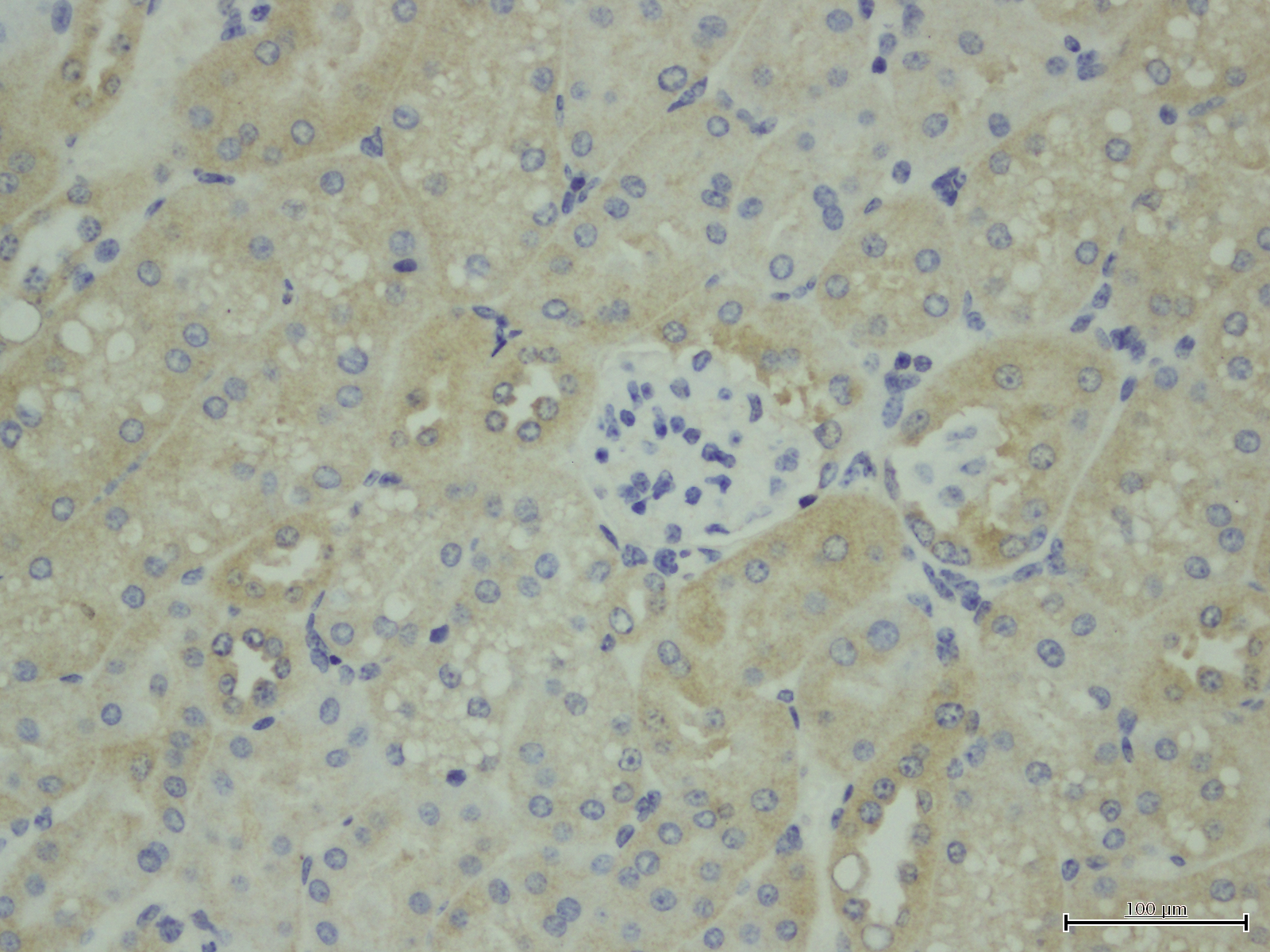

Supplement: S13 File — (ZIP) [file pone.0327042.s013.zip › DM 75mGy 8w-2.tif]

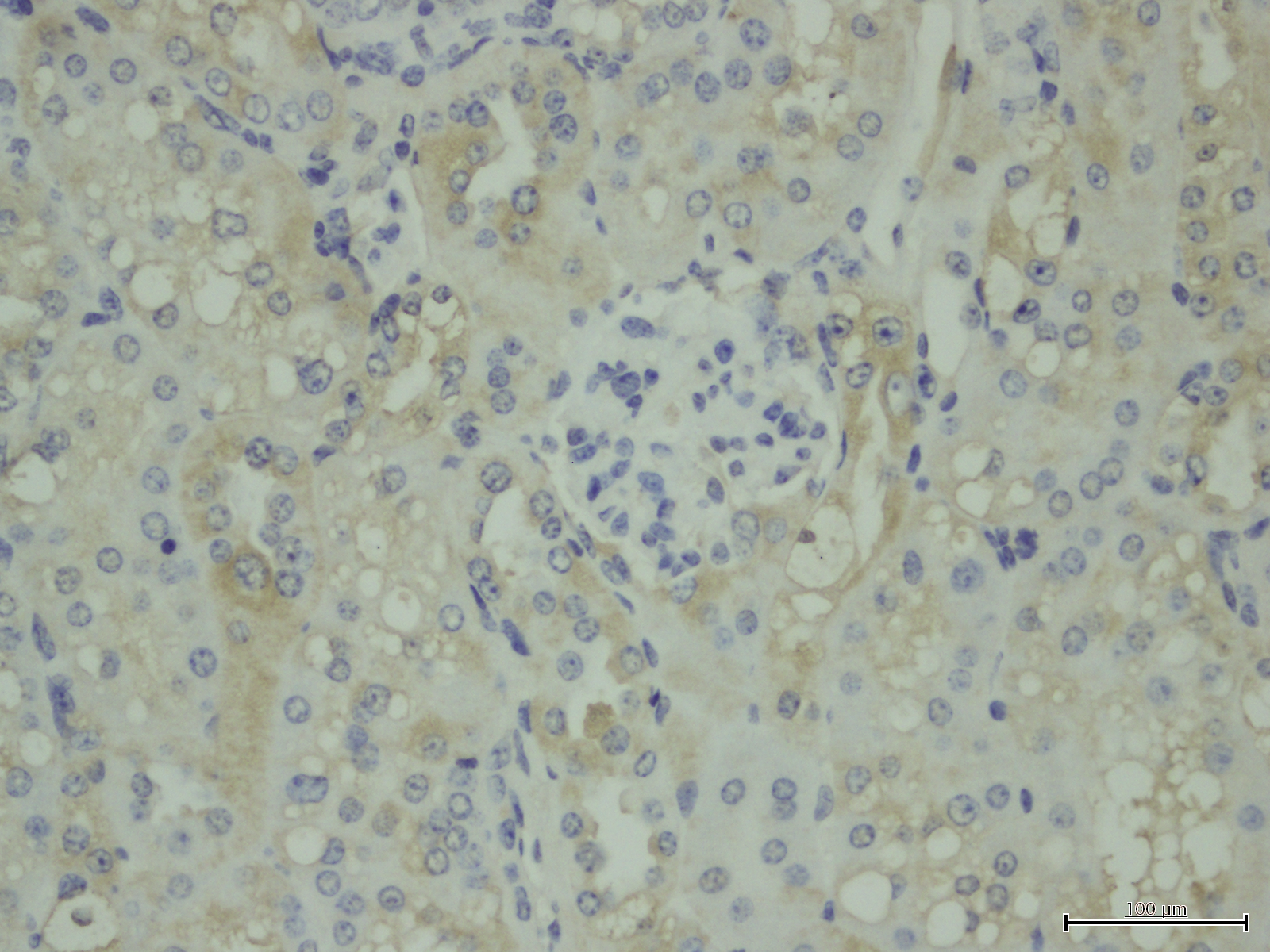

Supplement: S13 File — (ZIP) [file pone.0327042.s013.zip › DM 25mGy 4w-1(Used publication).tif]

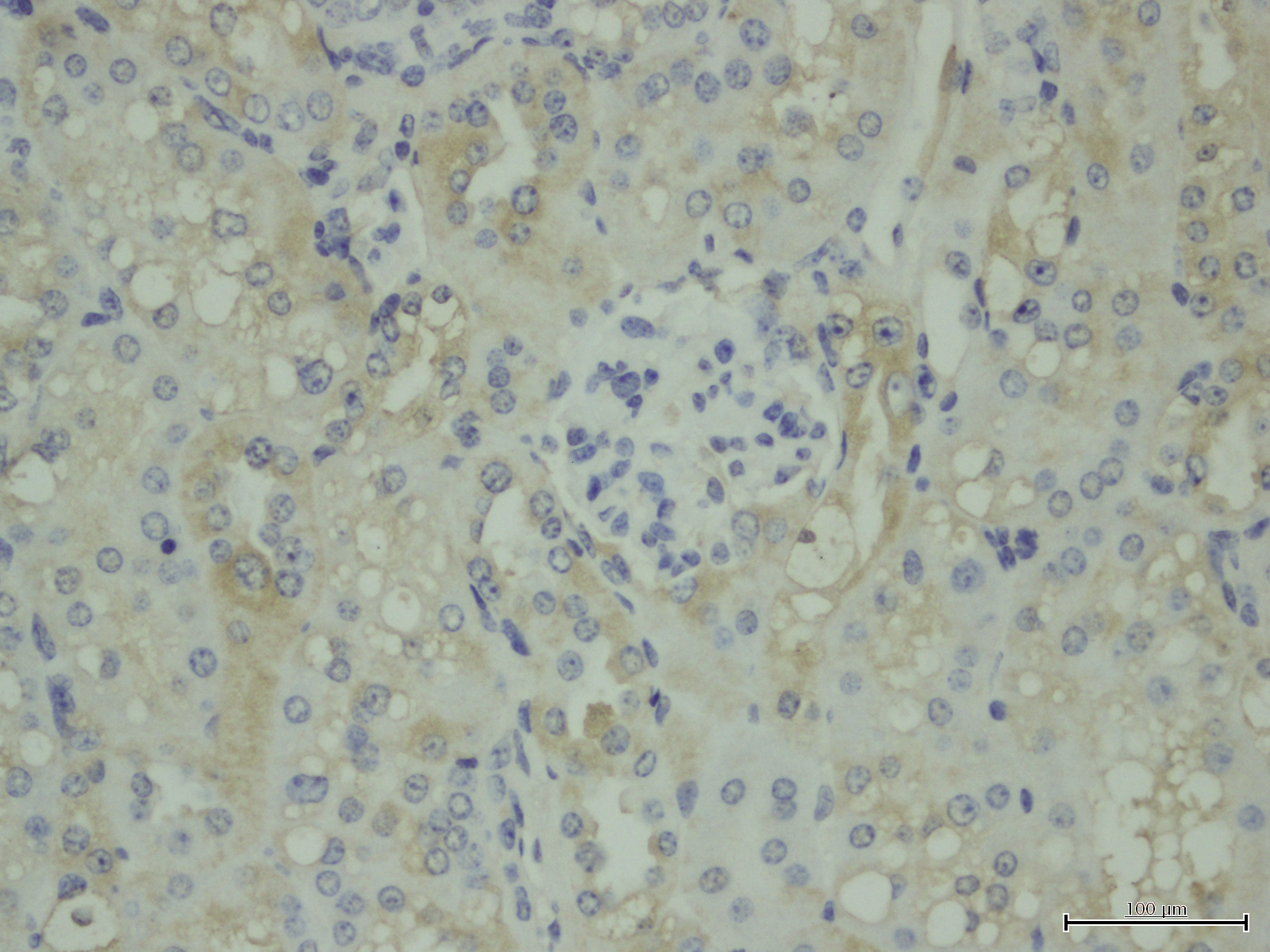

Supplement: S13 File — (ZIP) [file pone.0327042.s013.zip › DM 25mGy 4w-1_1.tif]

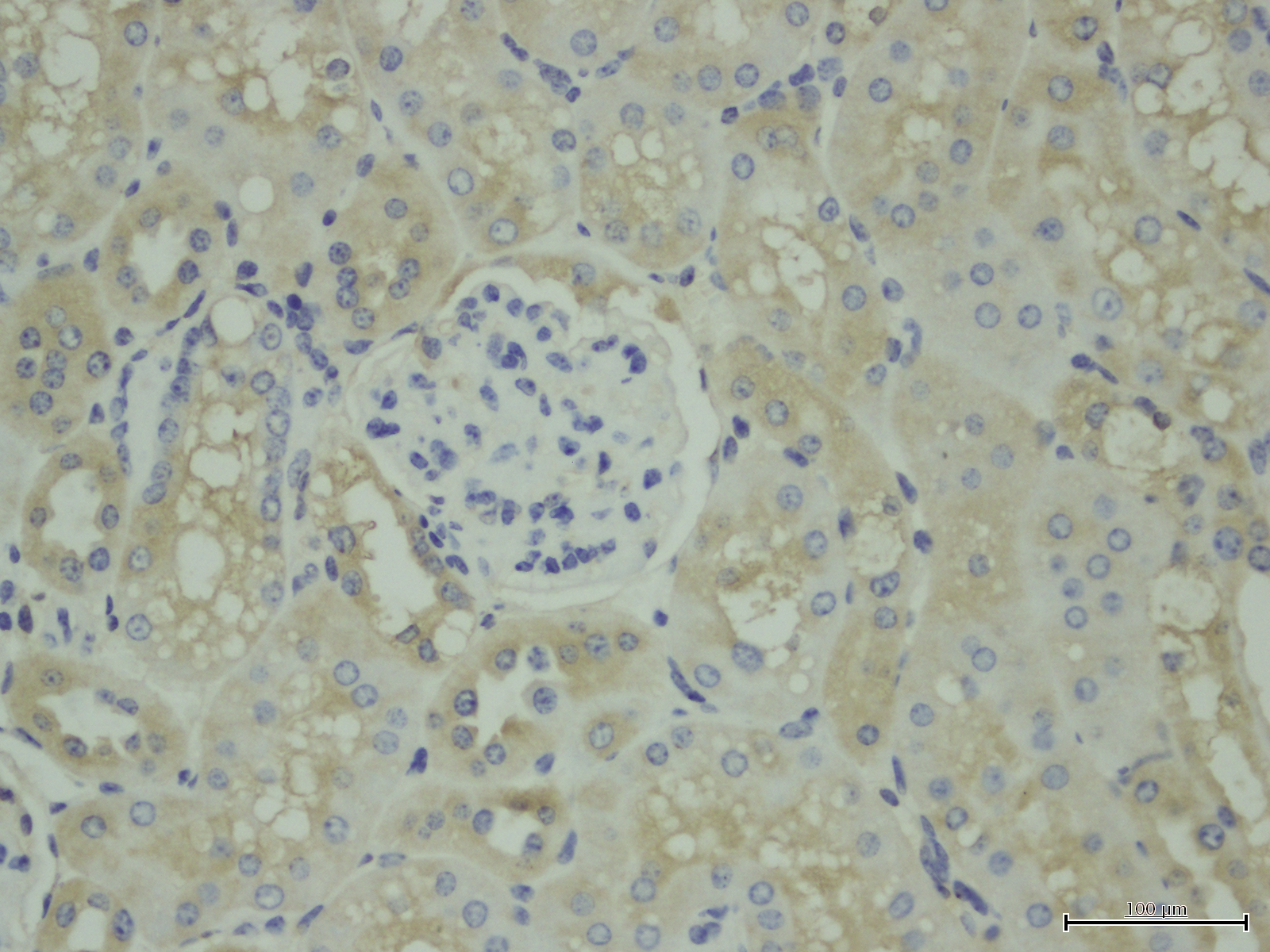

Supplement: S13 File — (ZIP) [file pone.0327042.s013.zip › DM 25mGy 4w-2.tif]

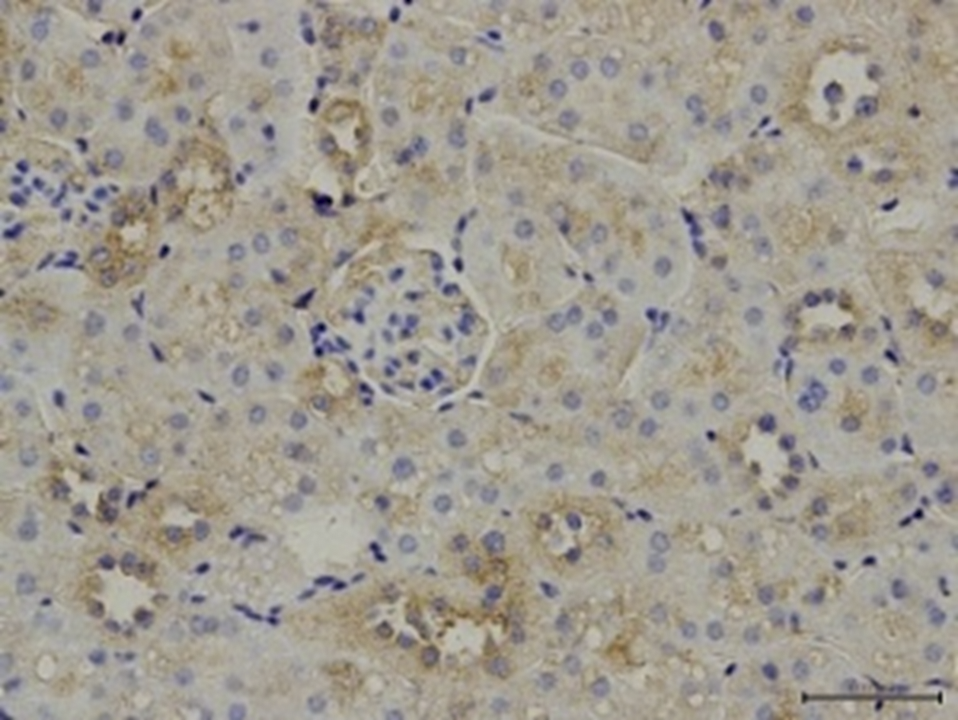

Supplement: S13 File — (ZIP) [file pone.0327042.s013.zip › DM 25mGy 8w -1(Used publication).tif]

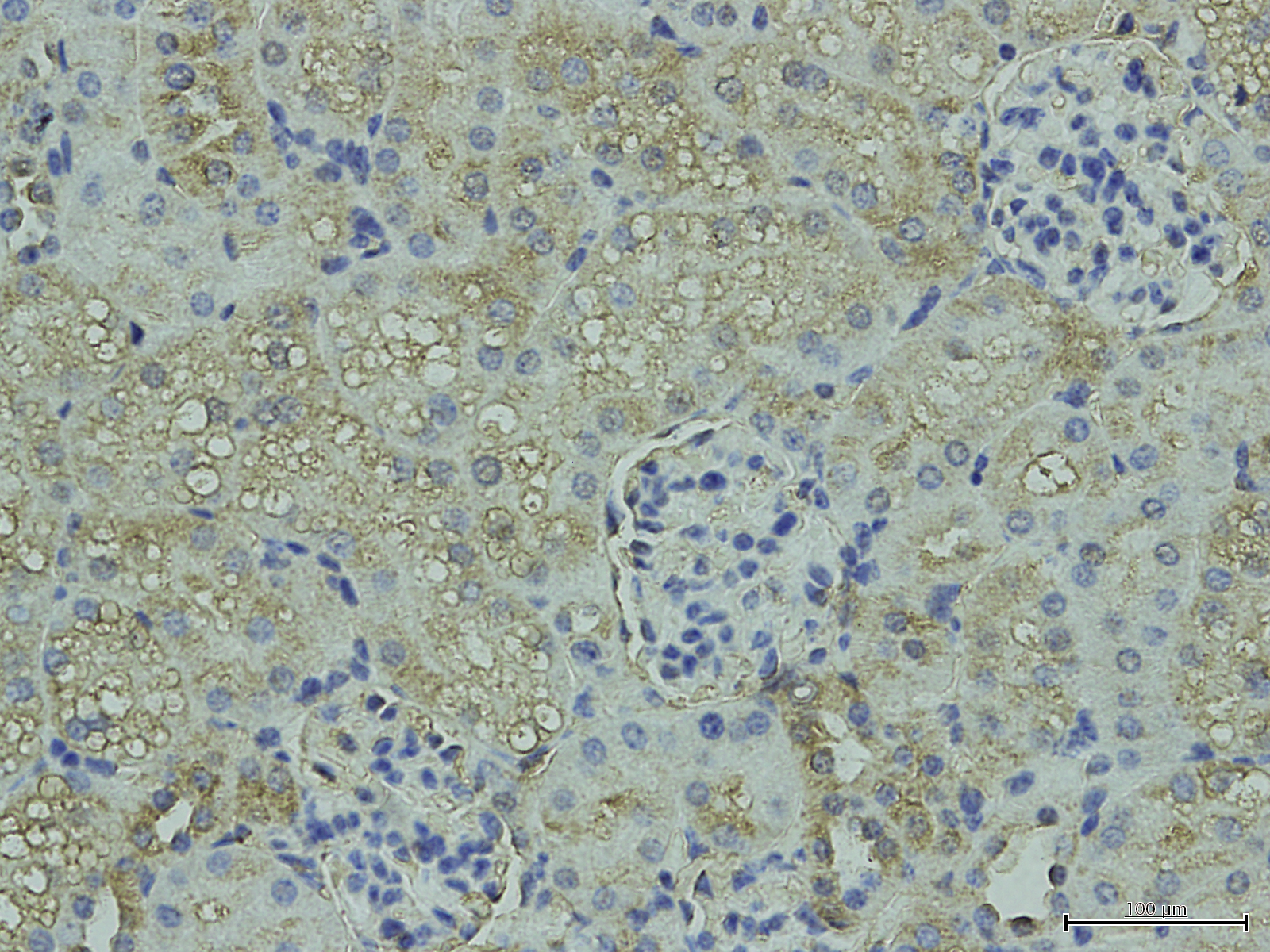

Supplement: S13 File — (ZIP) [file pone.0327042.s013.zip › DM 25mGy 8w-2.tif]

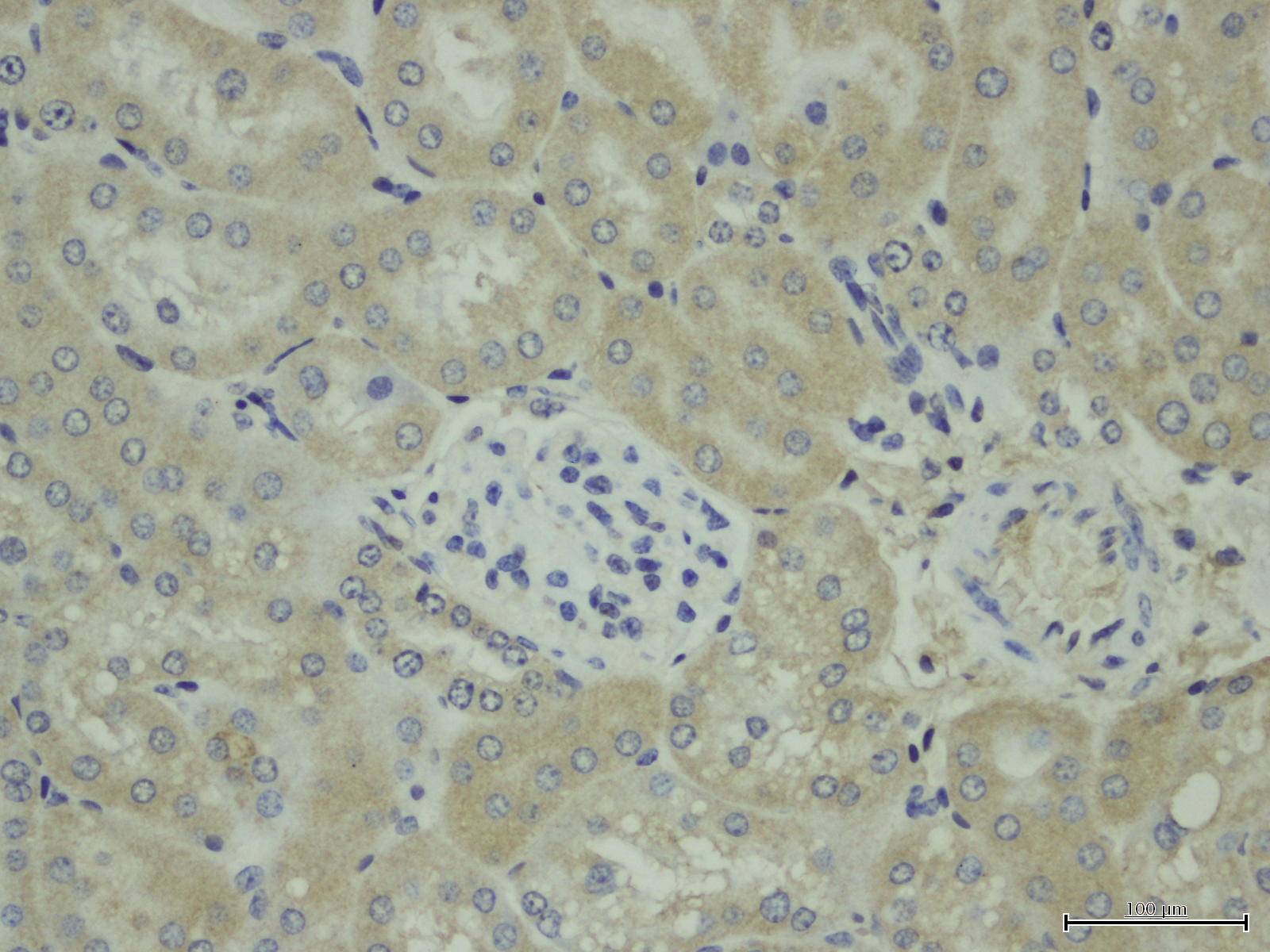

Supplement: S13 File — (ZIP) [file pone.0327042.s013.zip › DM 50mGy 4w-1(Used publication).tif]

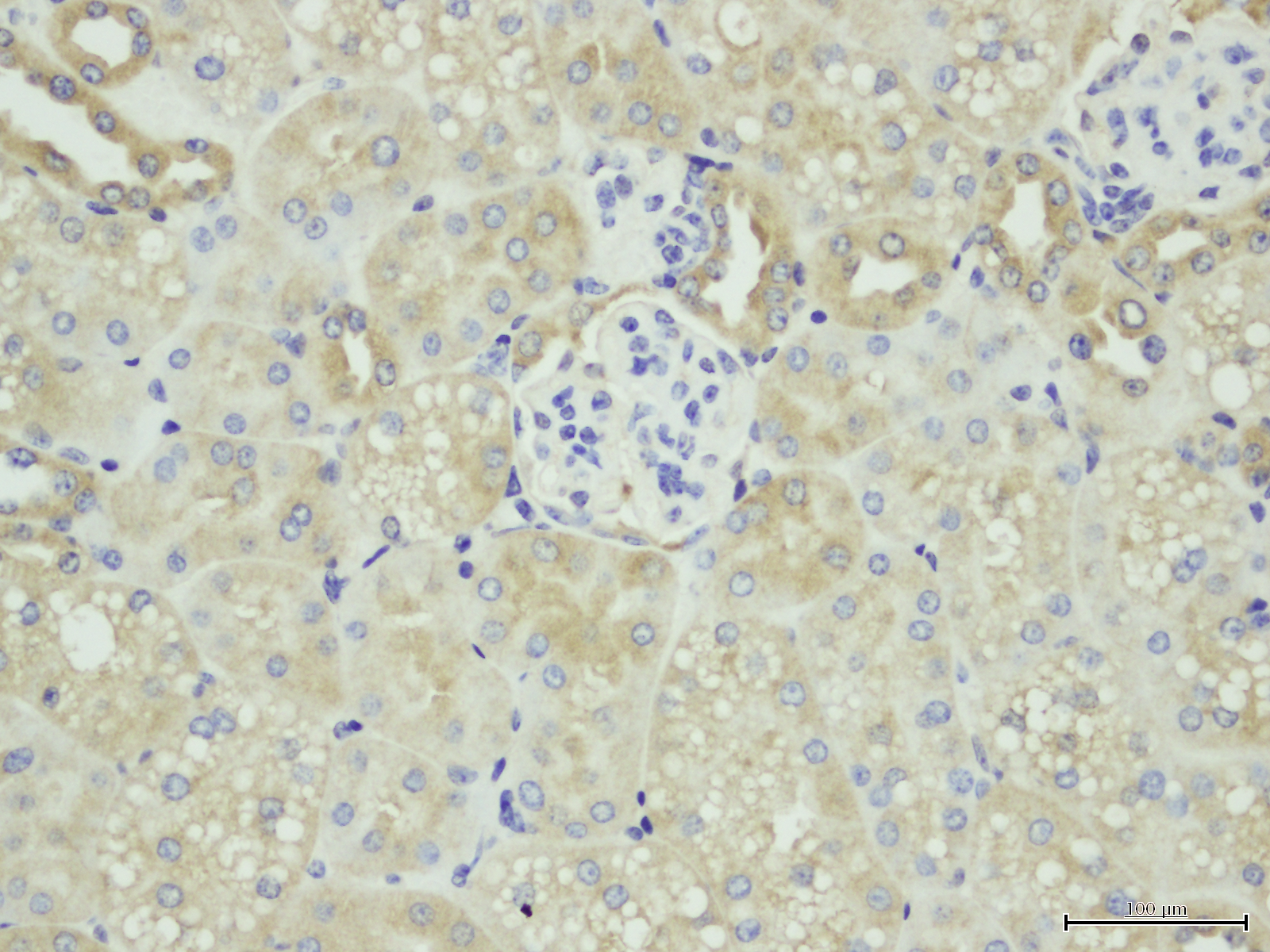

Supplement: S13 File — (ZIP) [file pone.0327042.s013.zip › DM 50mGy 4w-2.tif]

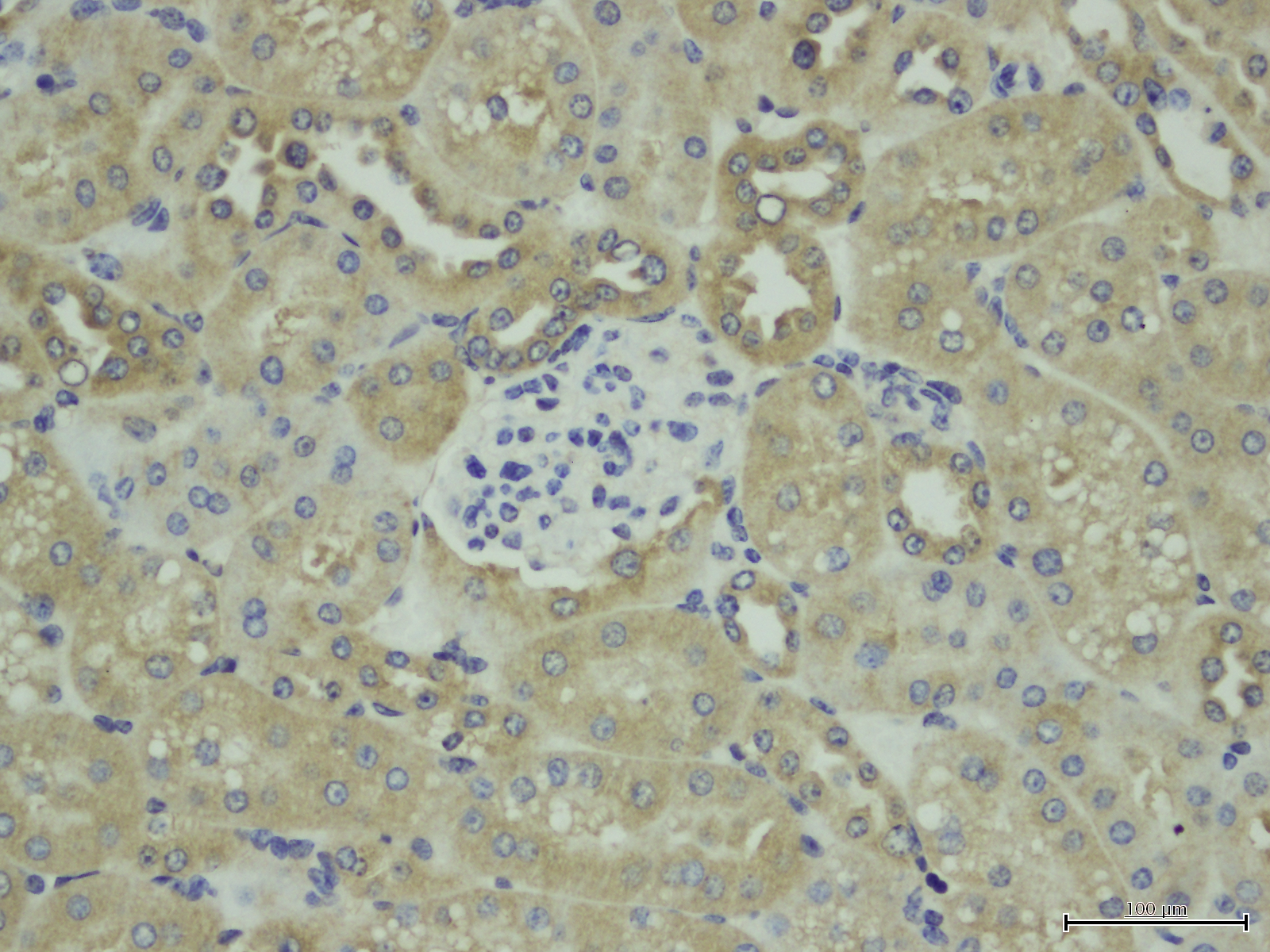

Supplement: S13 File — (ZIP) [file pone.0327042.s013.zip › DM 50mGy 4w-3.tif]

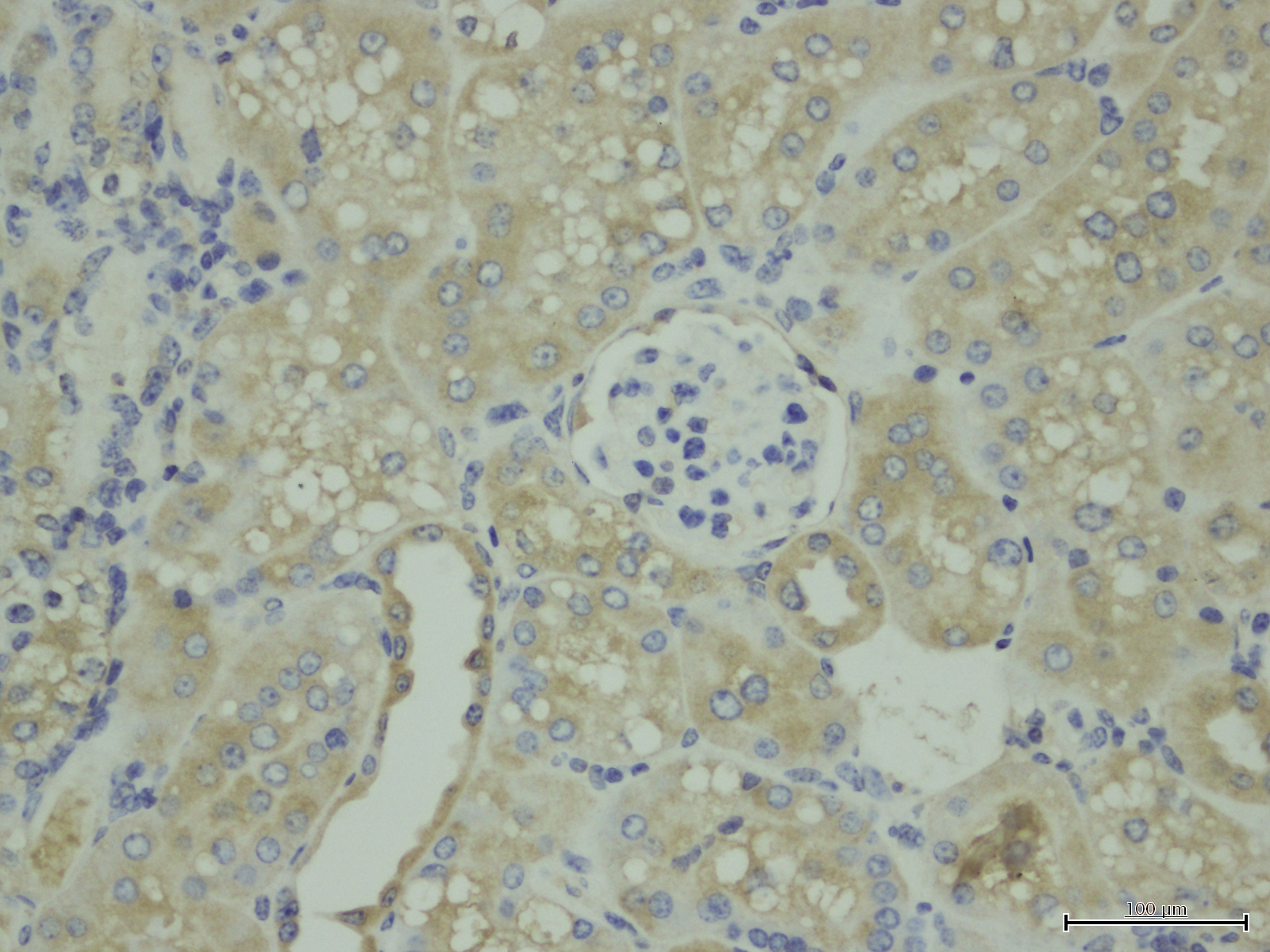

Supplement: S13 File — (ZIP) [file pone.0327042.s013.zip › DM 50mGy 8w -1(Used publication).tif]

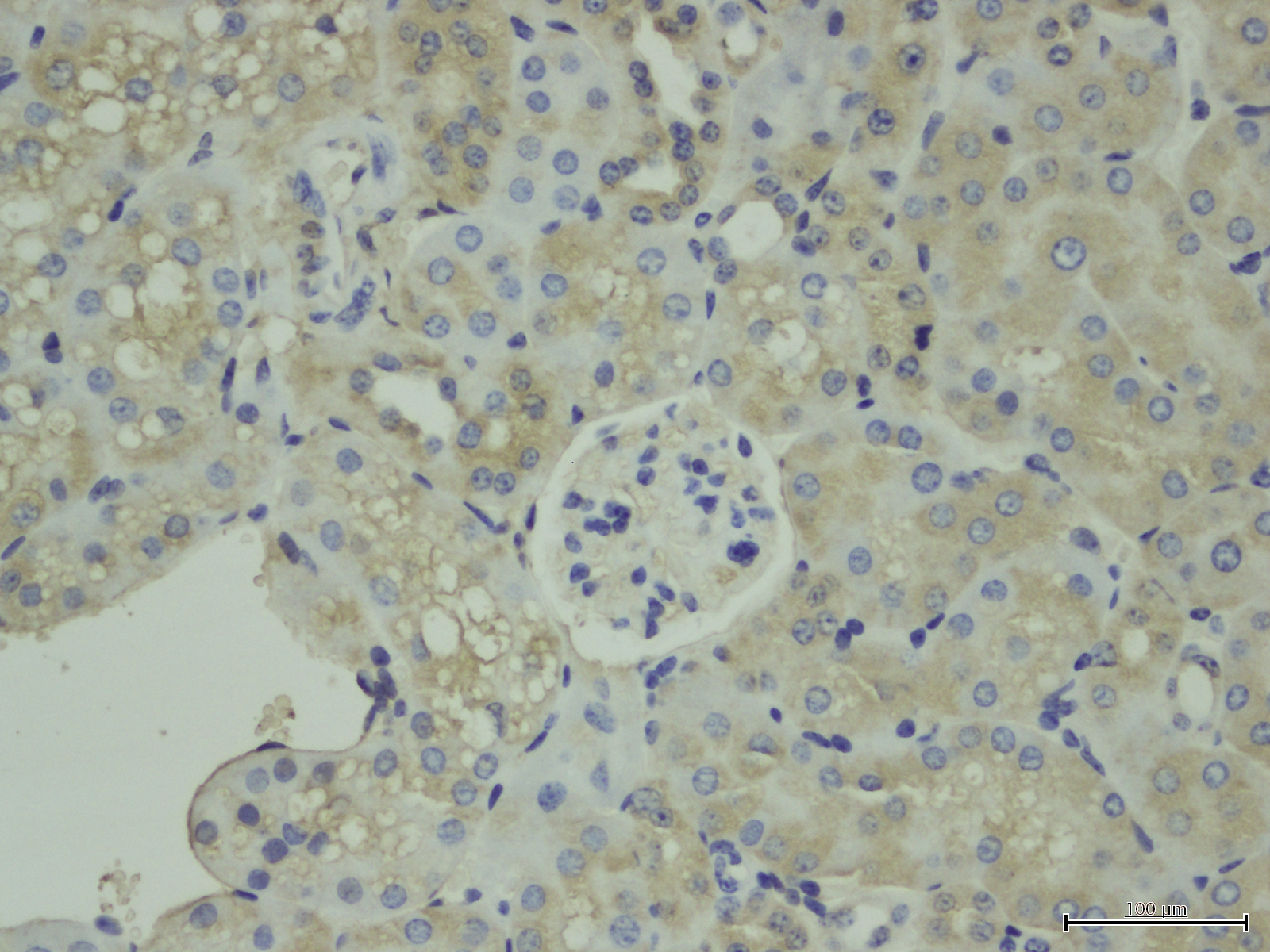

Supplement: S13 File — (ZIP) [file pone.0327042.s013.zip › DM 50mGy 8w-2.tif]

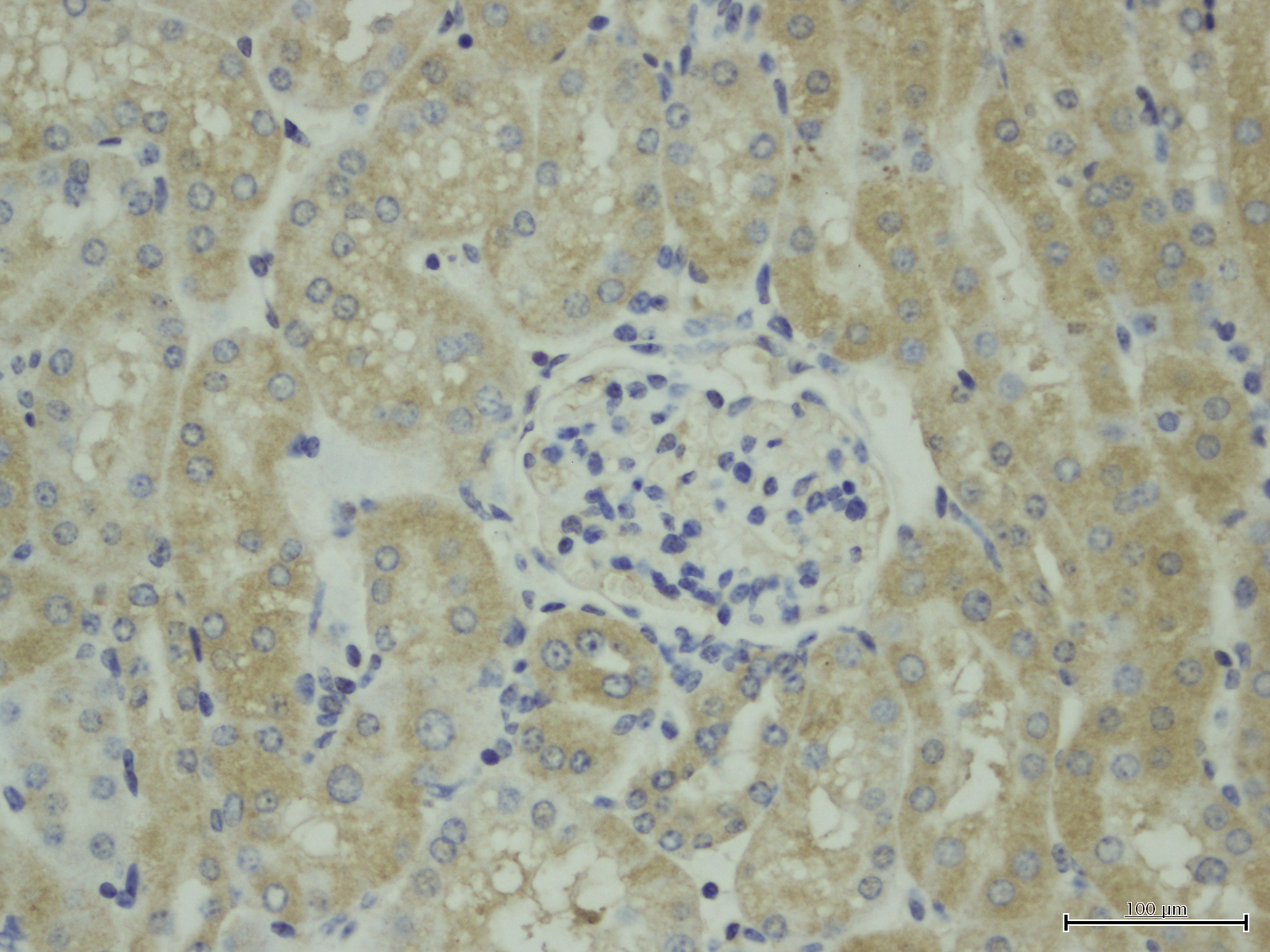

Supplement: S13 File — (ZIP) [file pone.0327042.s013.zip › DM 75mGy 4w -1(Used publication).tif]

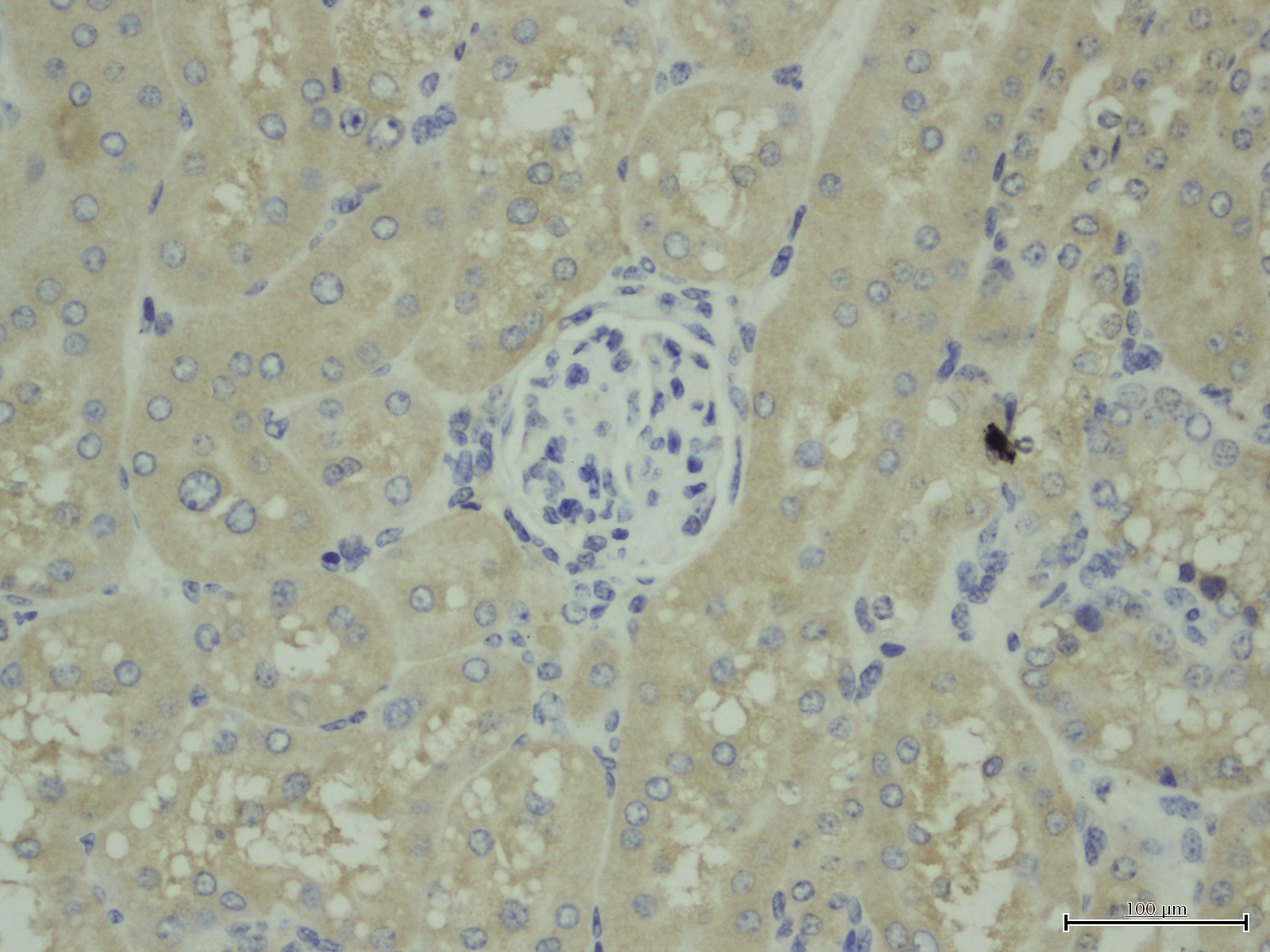

Supplement: S13 File — (ZIP) [file pone.0327042.s013.zip › DM 75mGy 4w -2.tif]

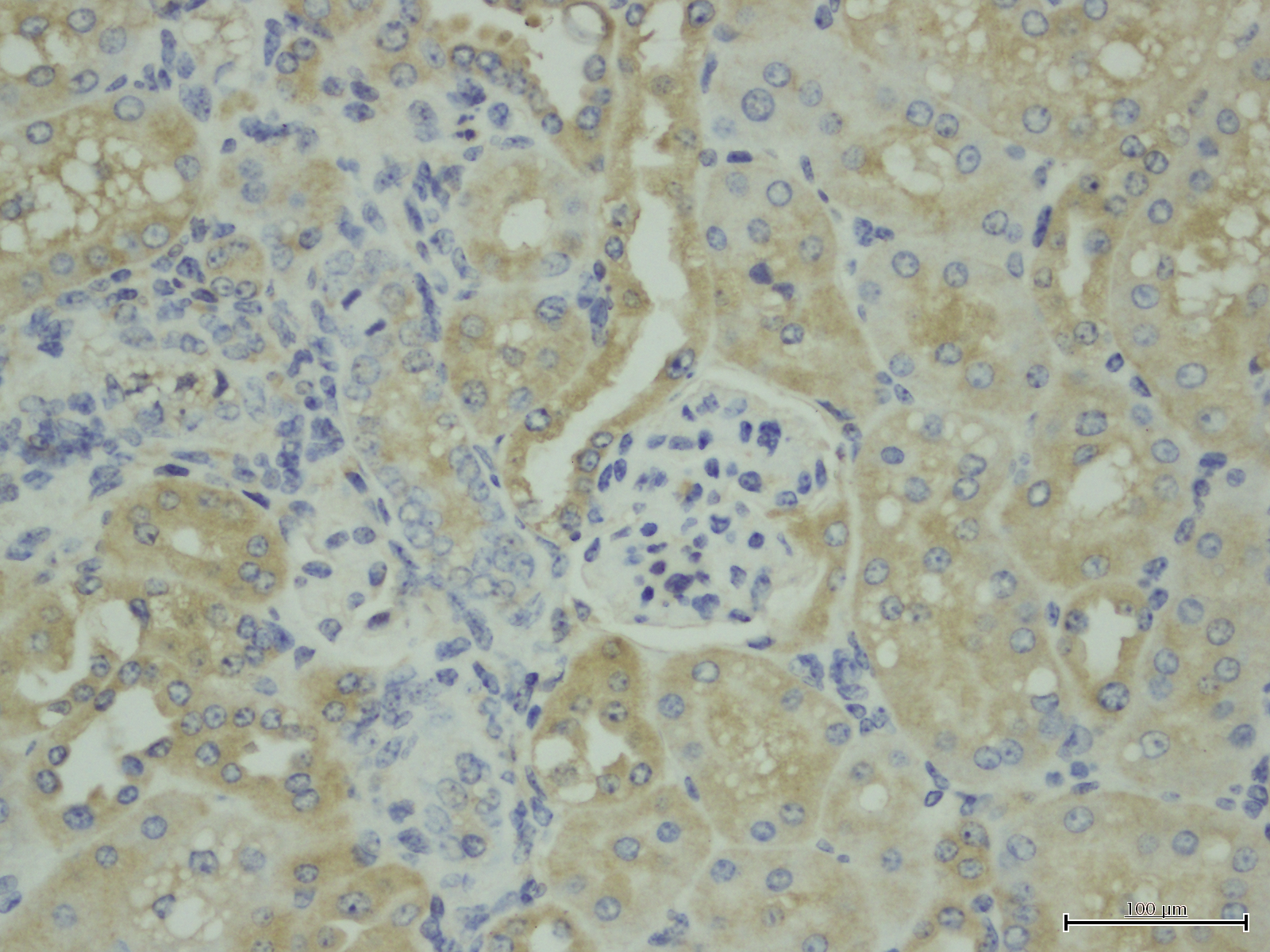

Supplement: S13 File — (ZIP) [file pone.0327042.s013.zip › DM 75mGy 4w-3.tif]

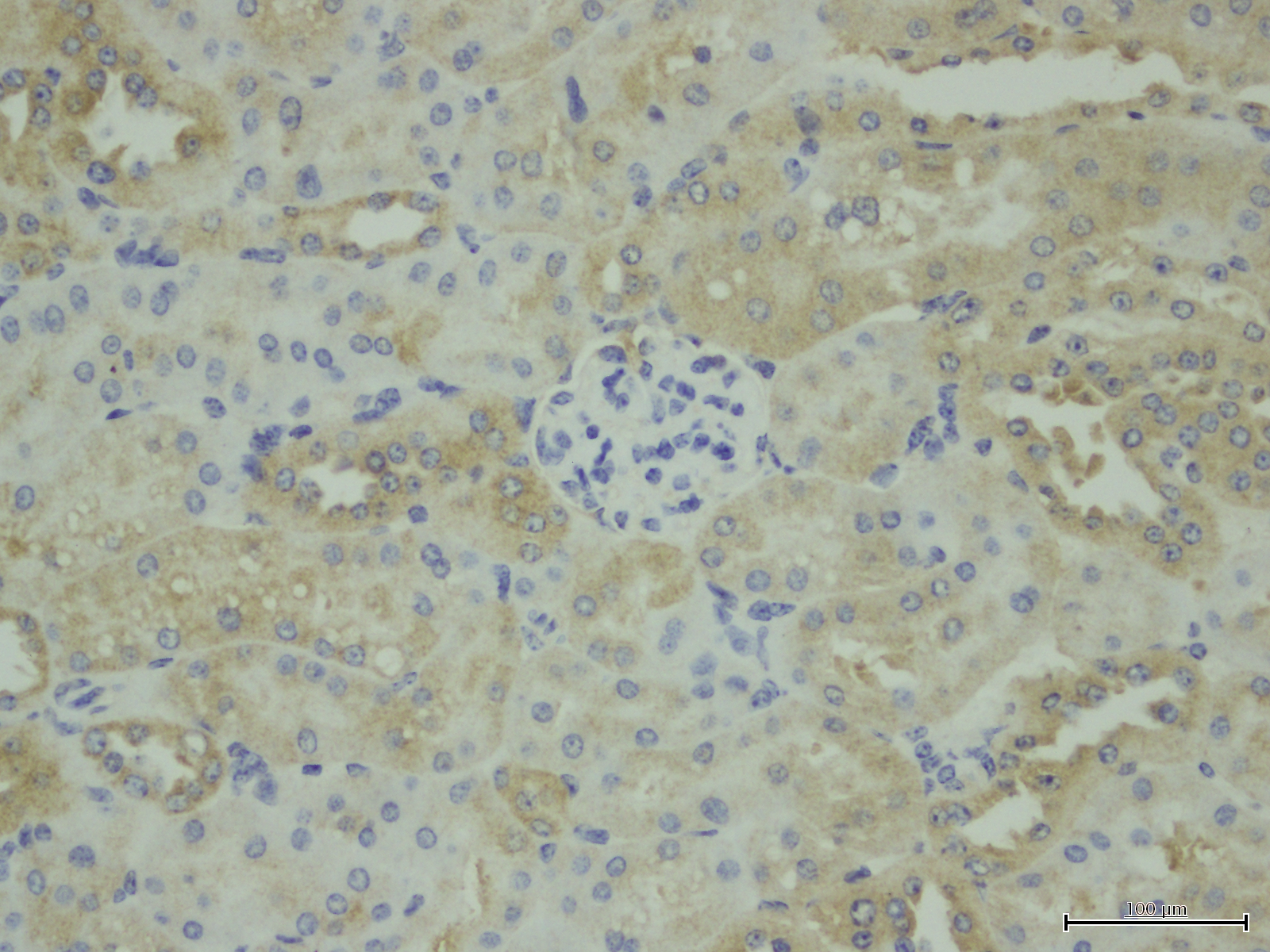

Supplement: S13 File — (ZIP) [file pone.0327042.s013.zip › DM 75mGy 8w-1(Used publication).tif]

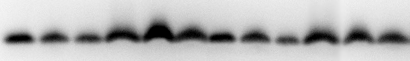

Supplement: S14 File — (TIF) [file pone.0327042.s014.tif]

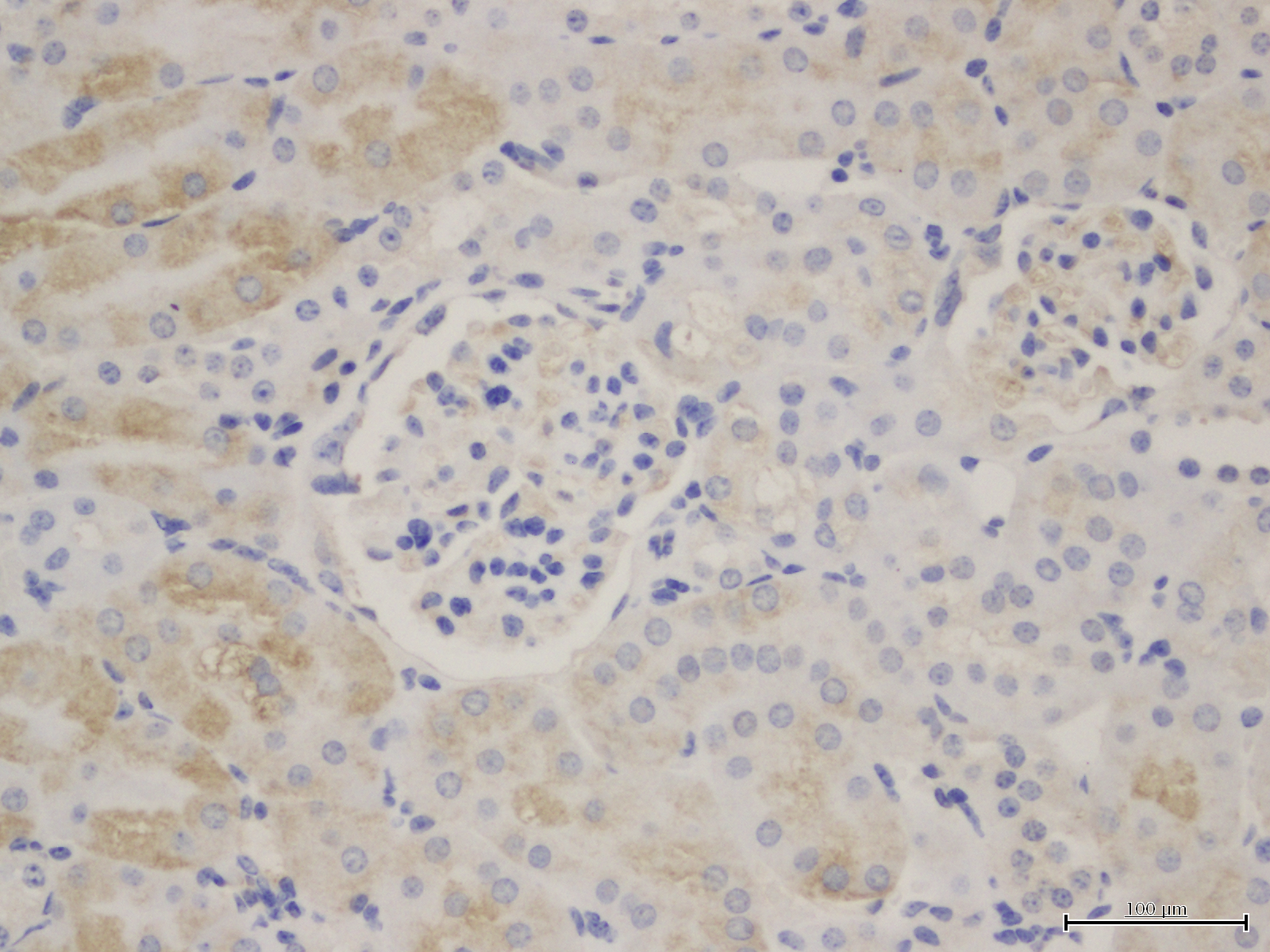

Supplement: S15 File — (ZIP) [file pone.0327042.s015.zip › 4w Con-1(Used publication).tif]

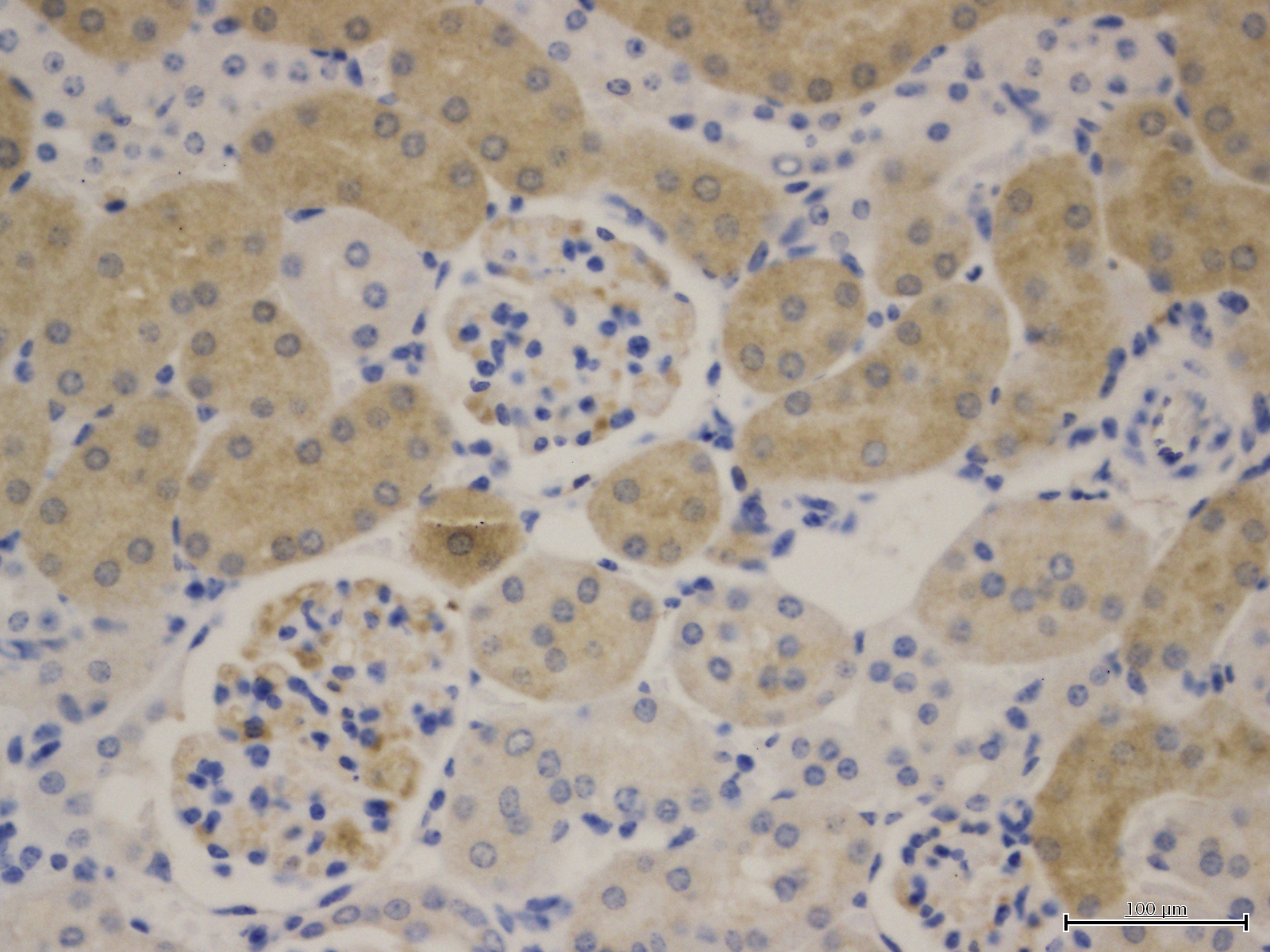

Supplement: S15 File — (ZIP) [file pone.0327042.s015.zip › 4w Con-2.tif]

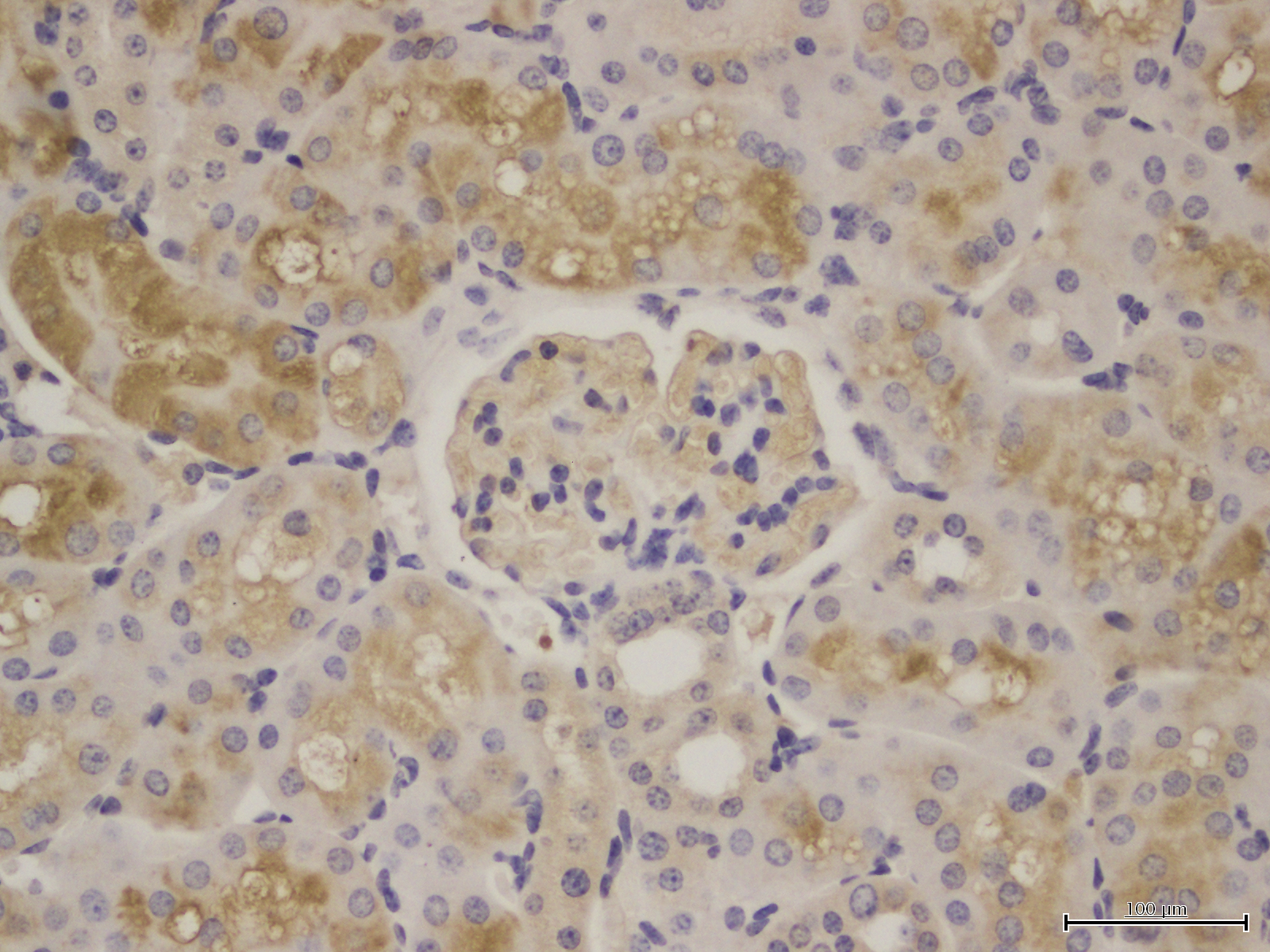

Supplement: S15 File — (ZIP) [file pone.0327042.s015.zip › 4w Con-3.tif]

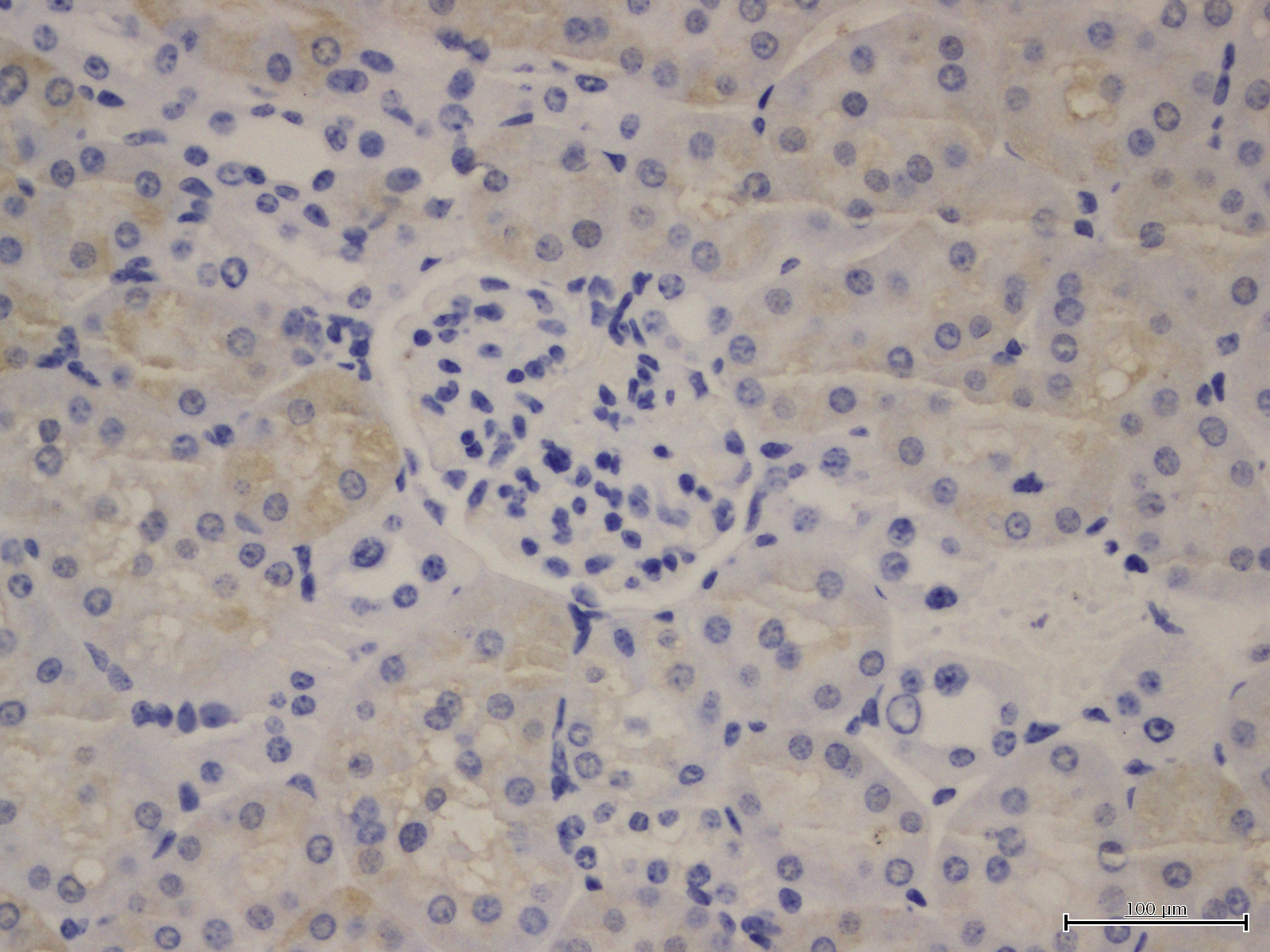

Supplement: S15 File — (ZIP) [file pone.0327042.s015.zip › 4w DM-1(Used publication).tif]

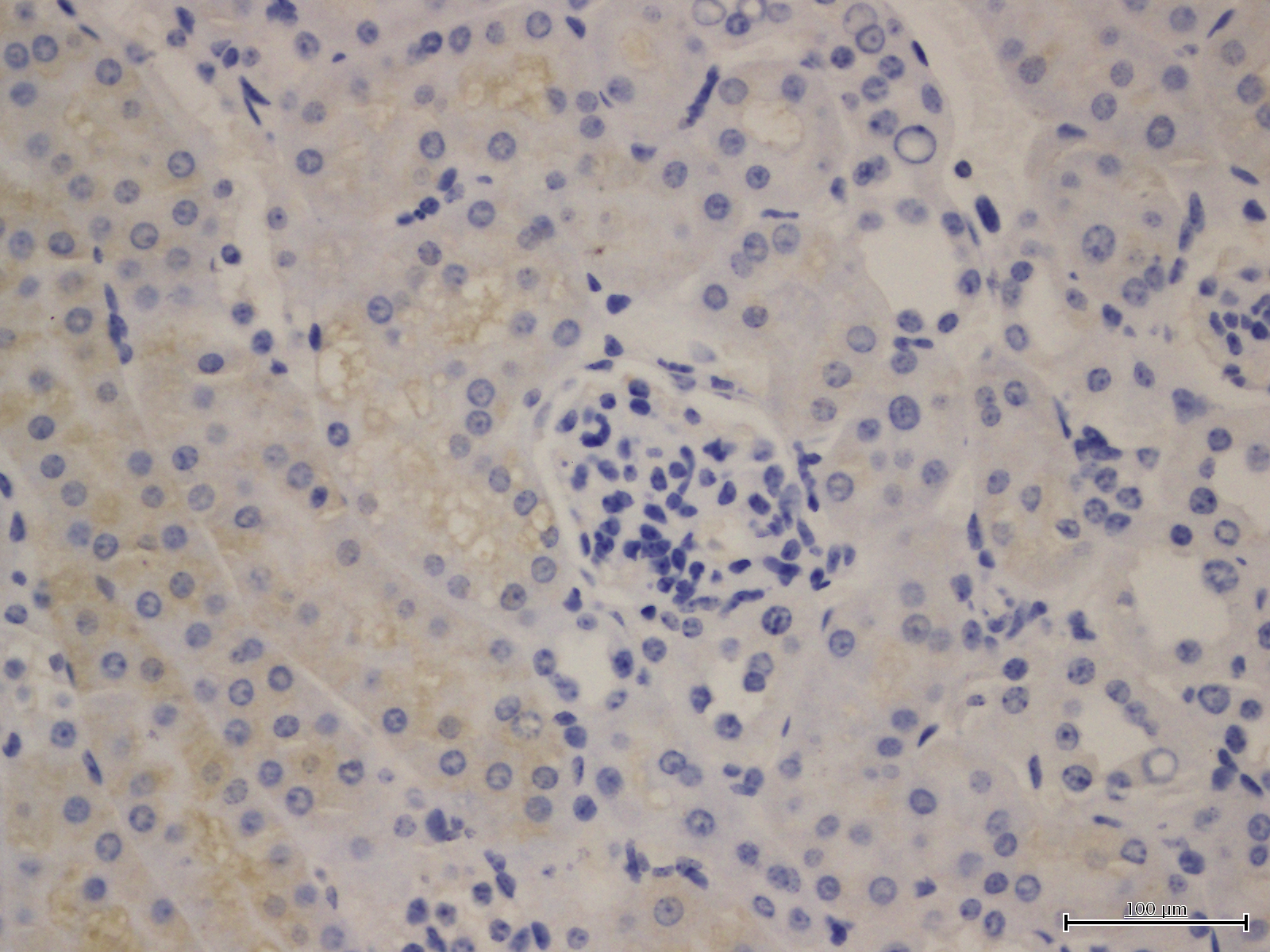

Supplement: S15 File — (ZIP) [file pone.0327042.s015.zip › 4w DM-2.tif]

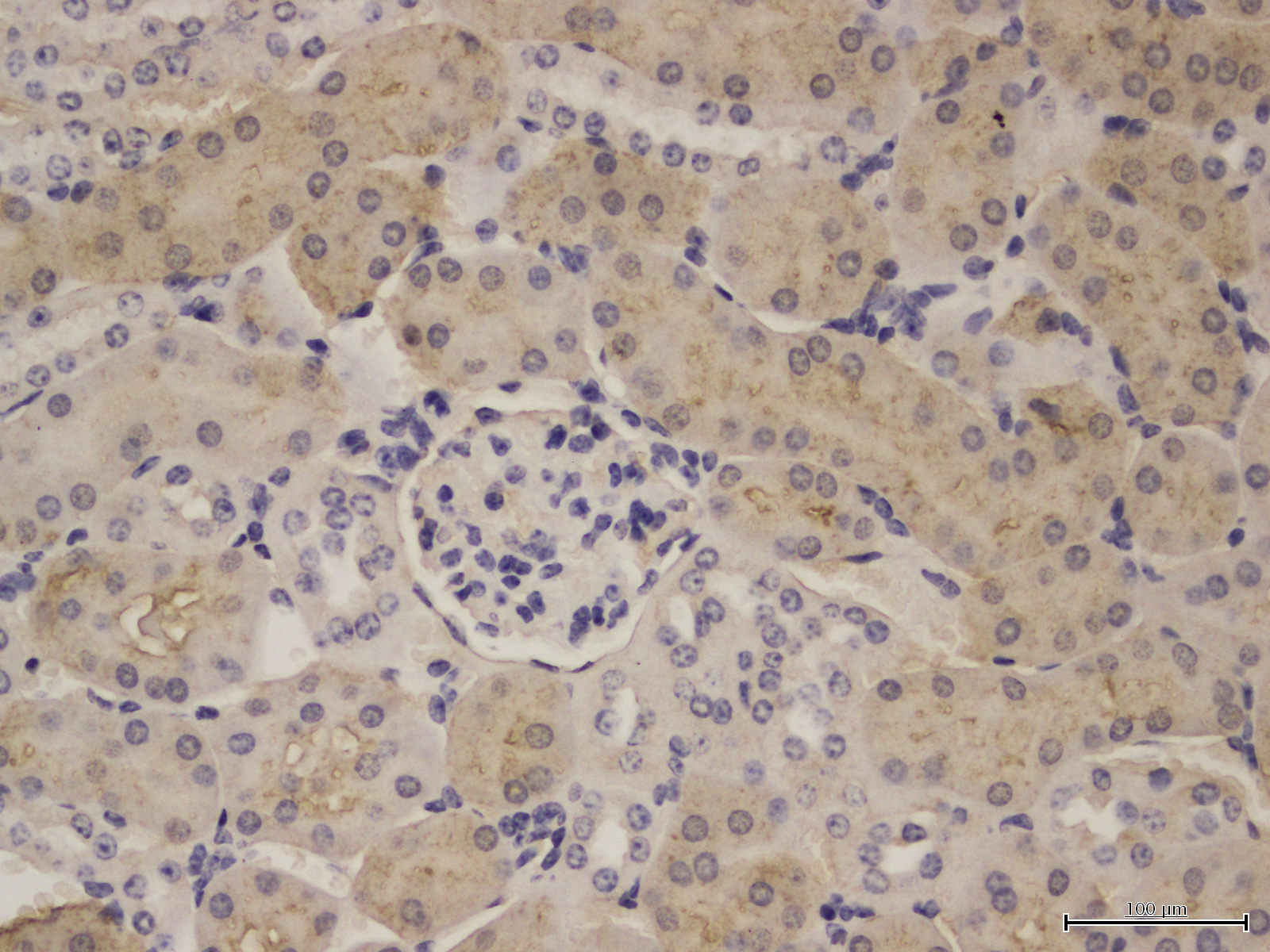

Supplement: S15 File — (ZIP) [file pone.0327042.s015.zip › 8w Con-1.tif]

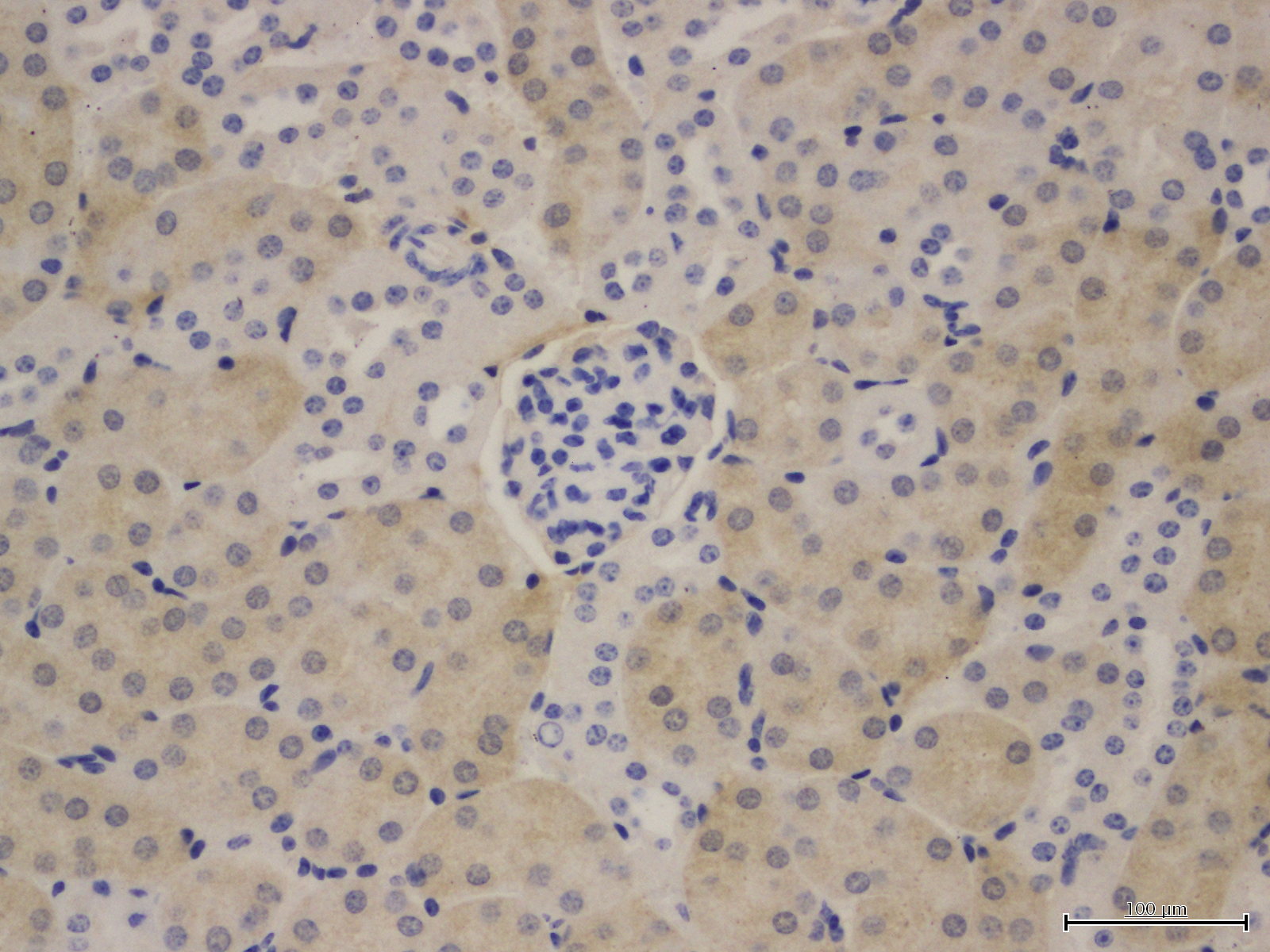

Supplement: S15 File — (ZIP) [file pone.0327042.s015.zip › 8w Con-2 (Used publication).tif]

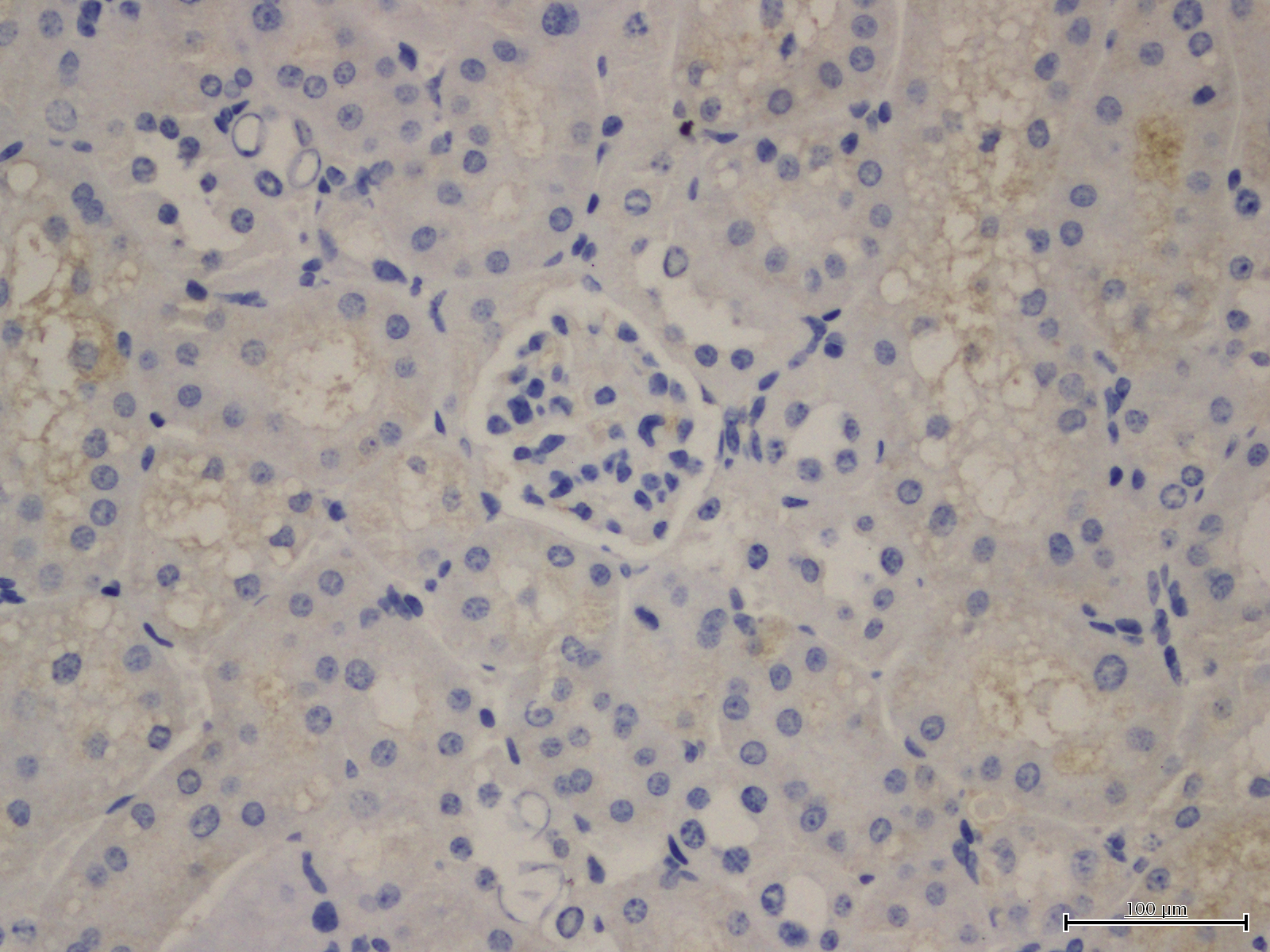

Supplement: S15 File — (ZIP) [file pone.0327042.s015.zip › 8w DM-1(Used publication).tif]

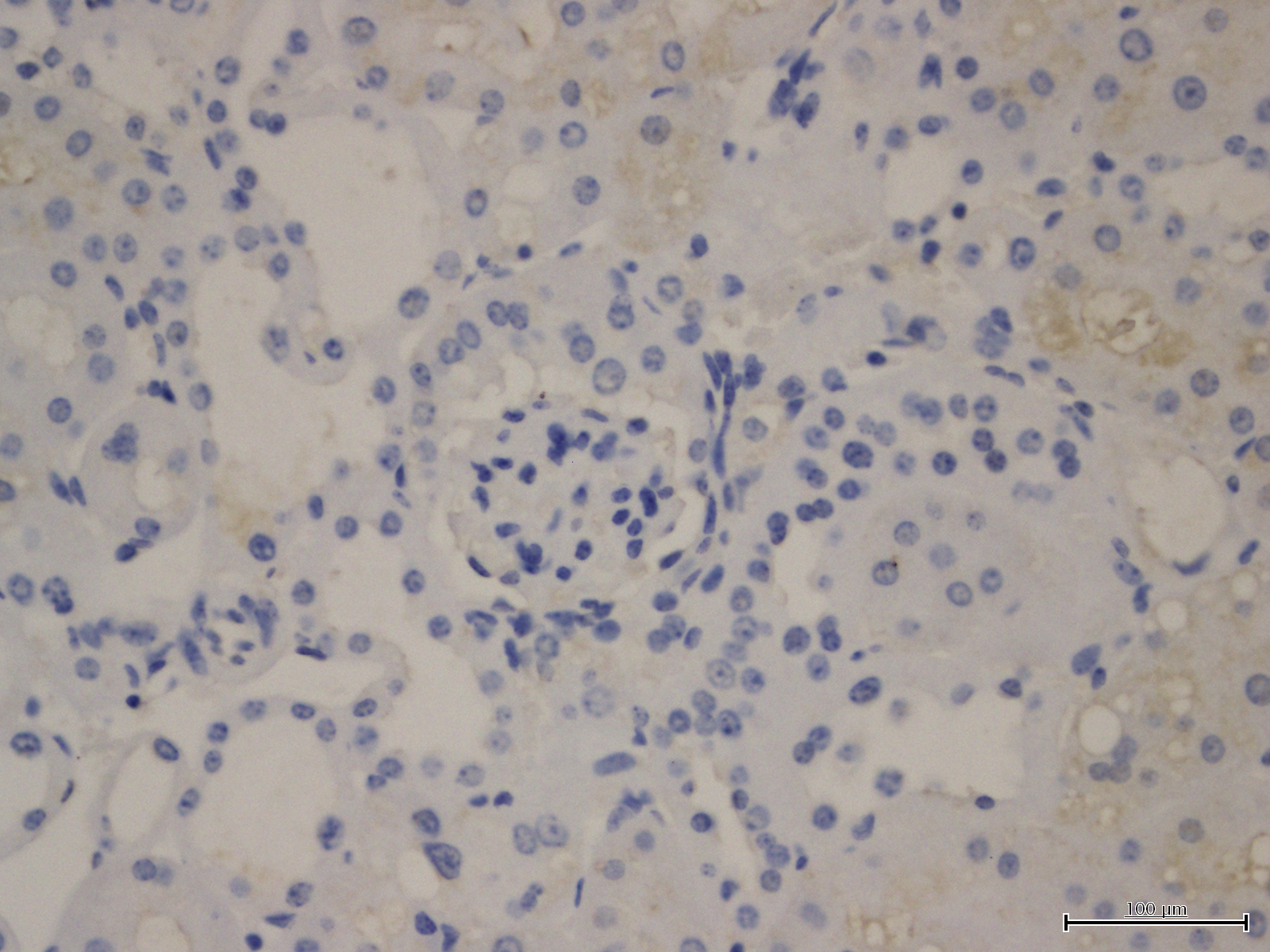

Supplement: S15 File — (ZIP) [file pone.0327042.s015.zip › 8w DM-2.tif]

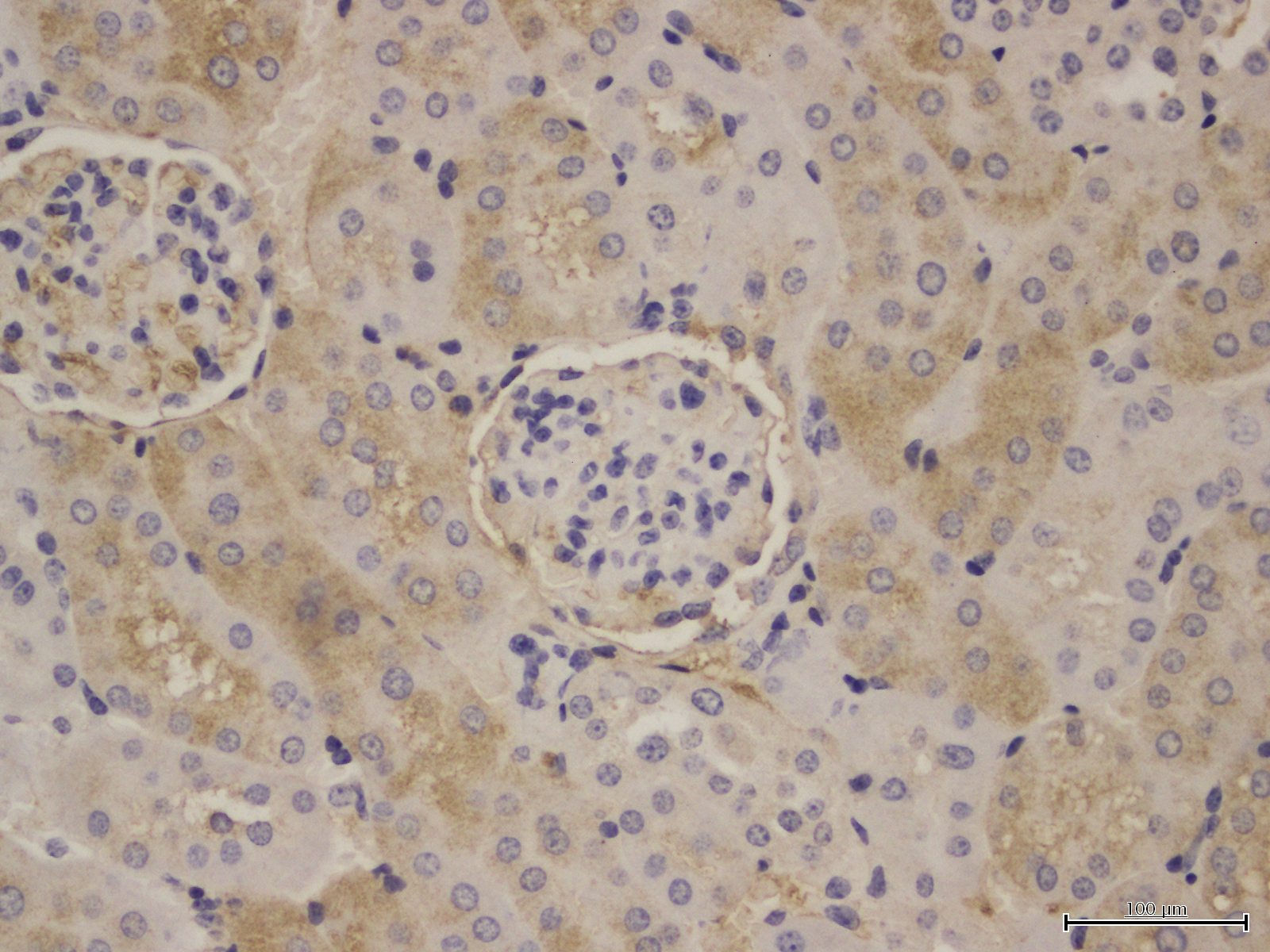

Supplement: S15 File — (ZIP) [file pone.0327042.s015.zip › 25mGy 4w DM-1(Used publication).tif]

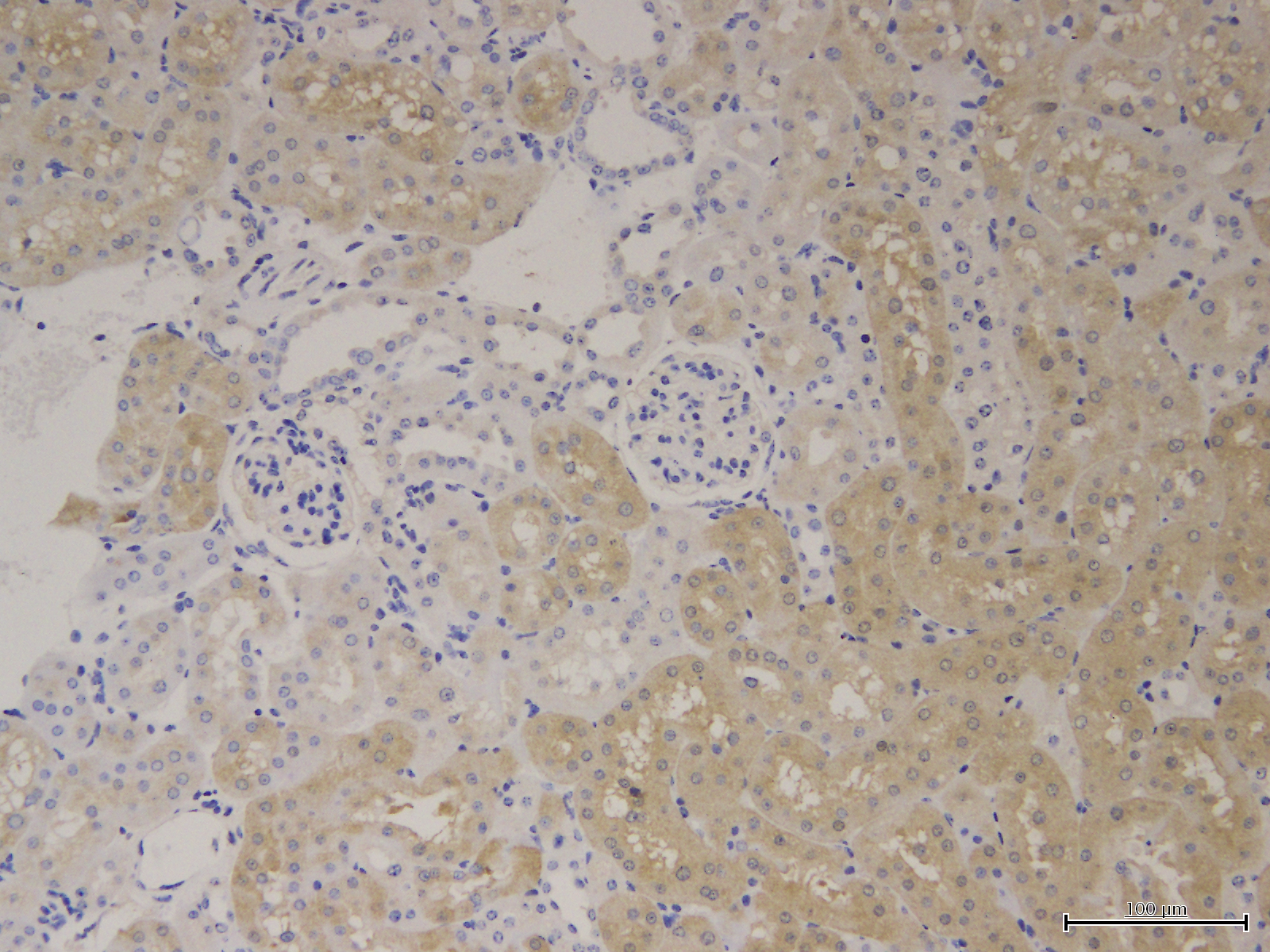

Supplement: S15 File — (ZIP) [file pone.0327042.s015.zip › 25mGy 4w DM-2.tif]

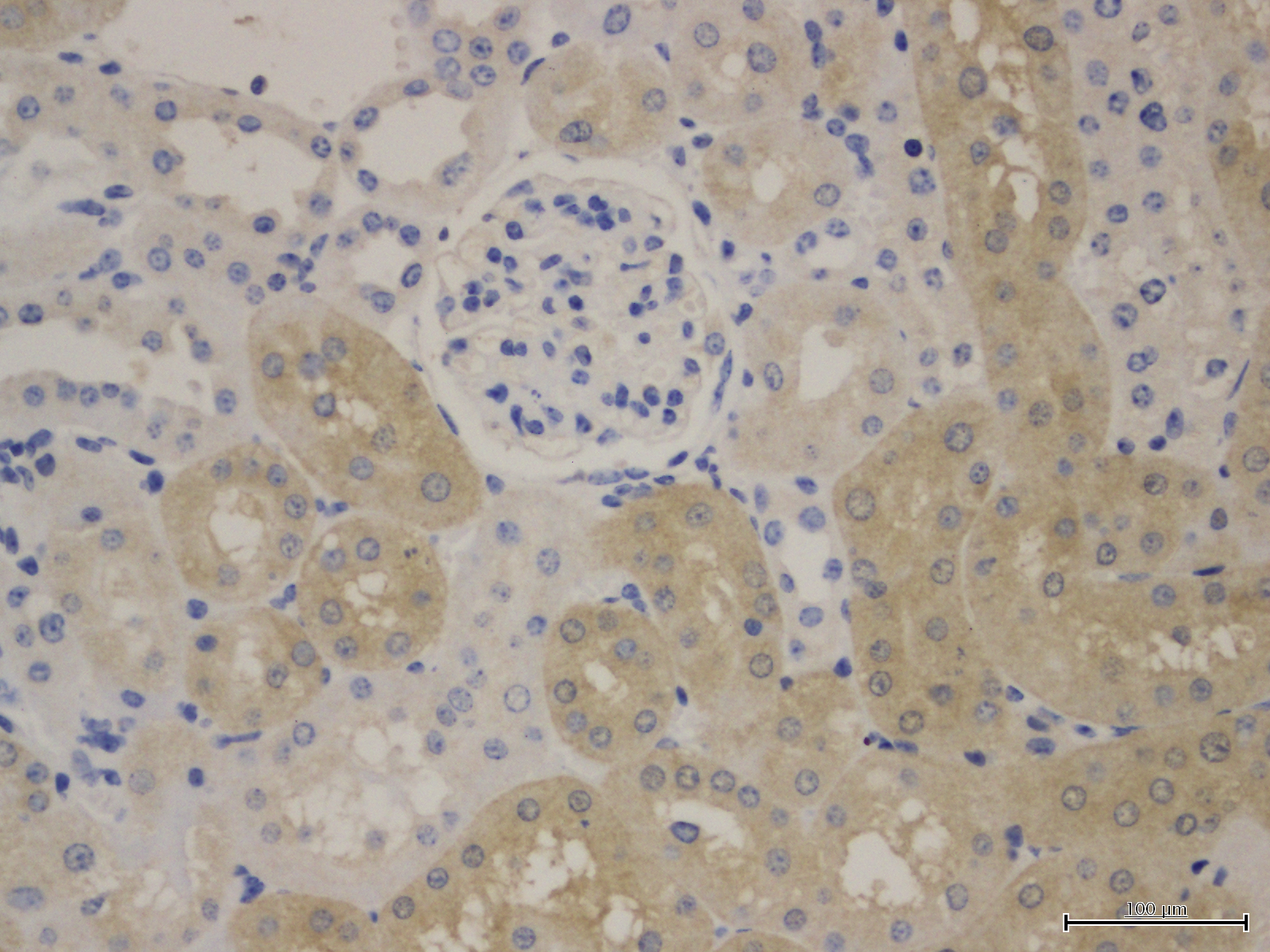

Supplement: S15 File — (ZIP) [file pone.0327042.s015.zip › 25mGy 4w DM-3.tif]

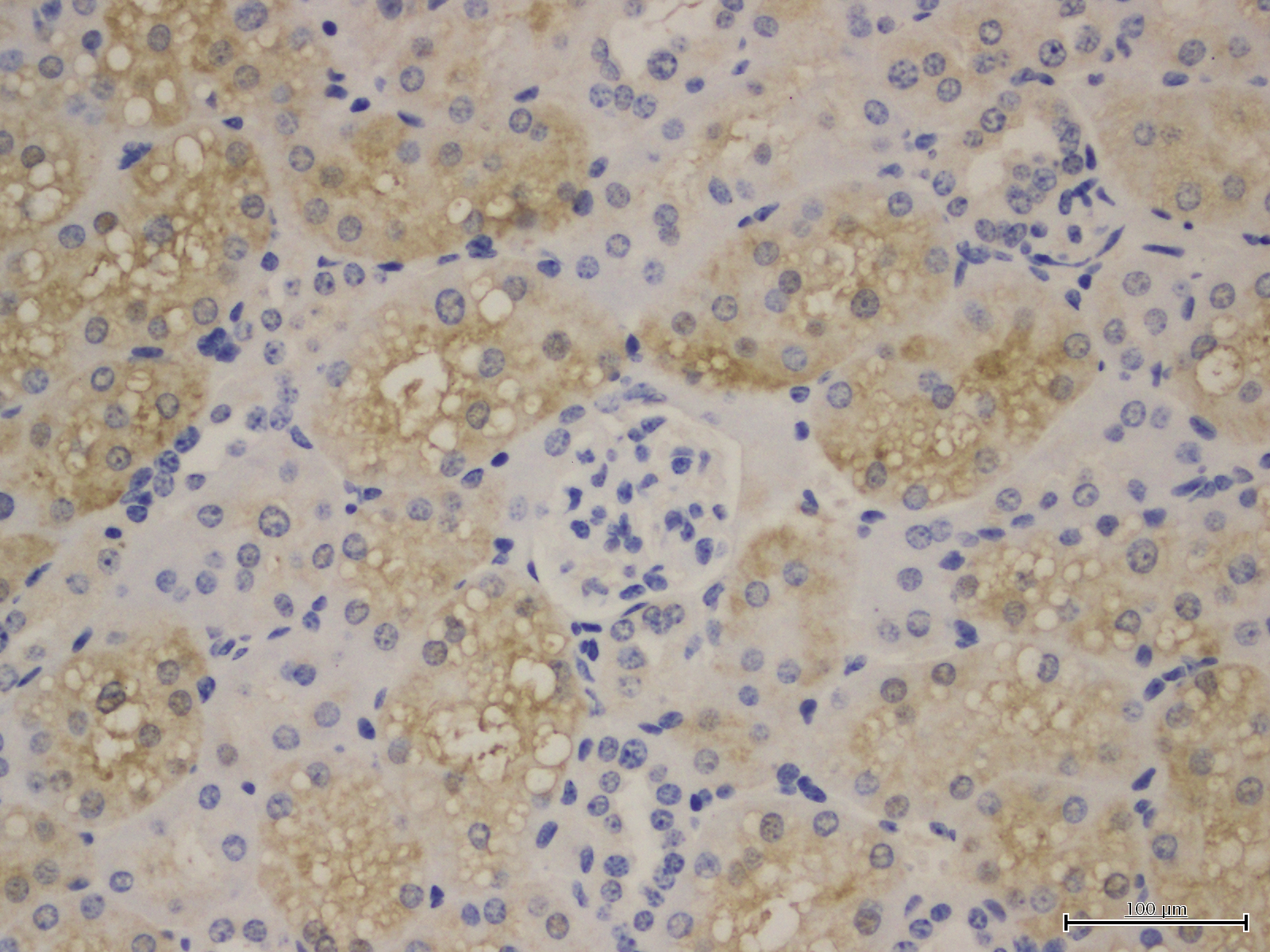

Supplement: S15 File — (ZIP) [file pone.0327042.s015.zip › 25mGy 8w DM-1(Used publication).tif]

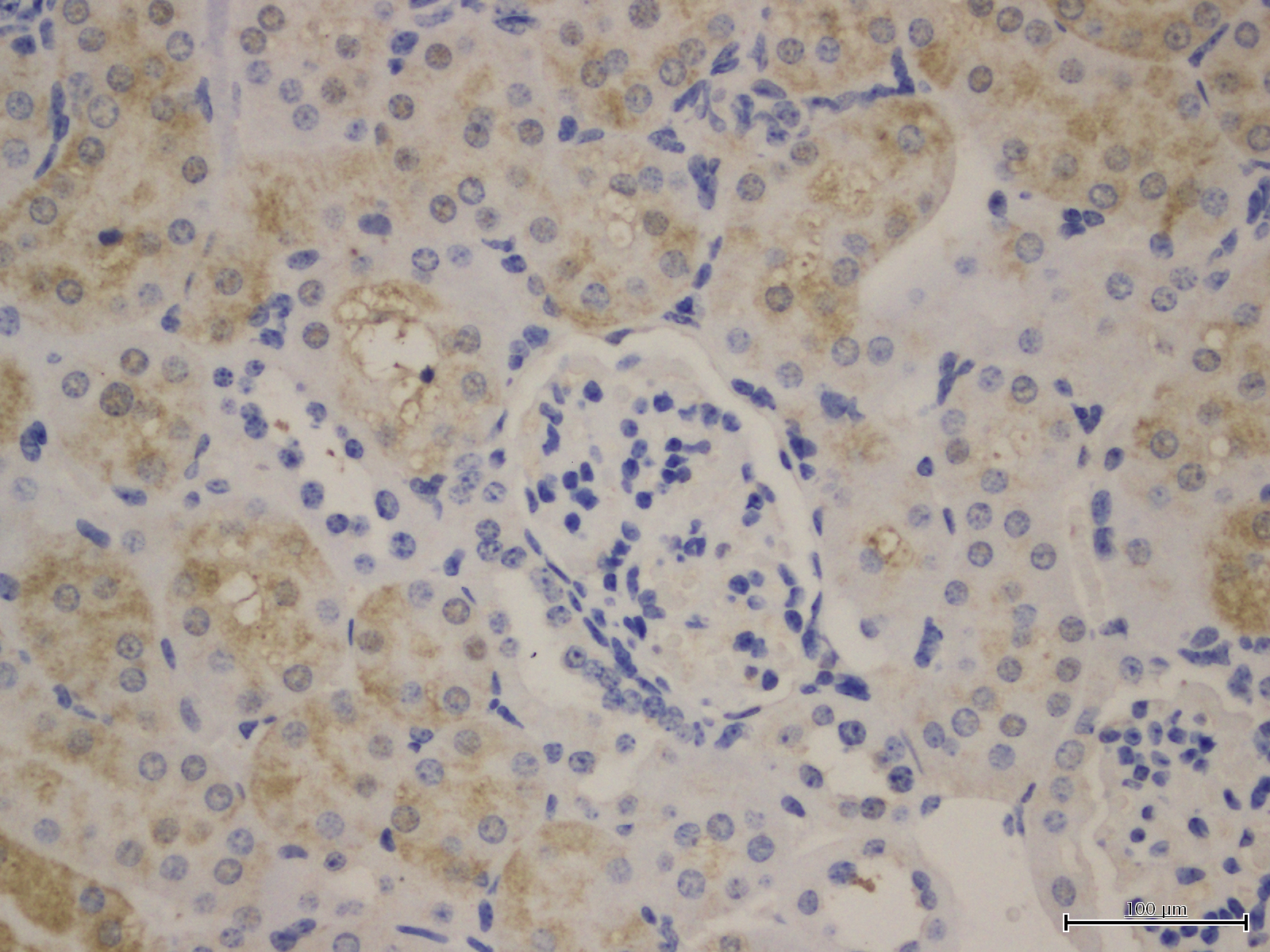

Supplement: S15 File — (ZIP) [file pone.0327042.s015.zip › 25mGy 8w DM-2.tif]

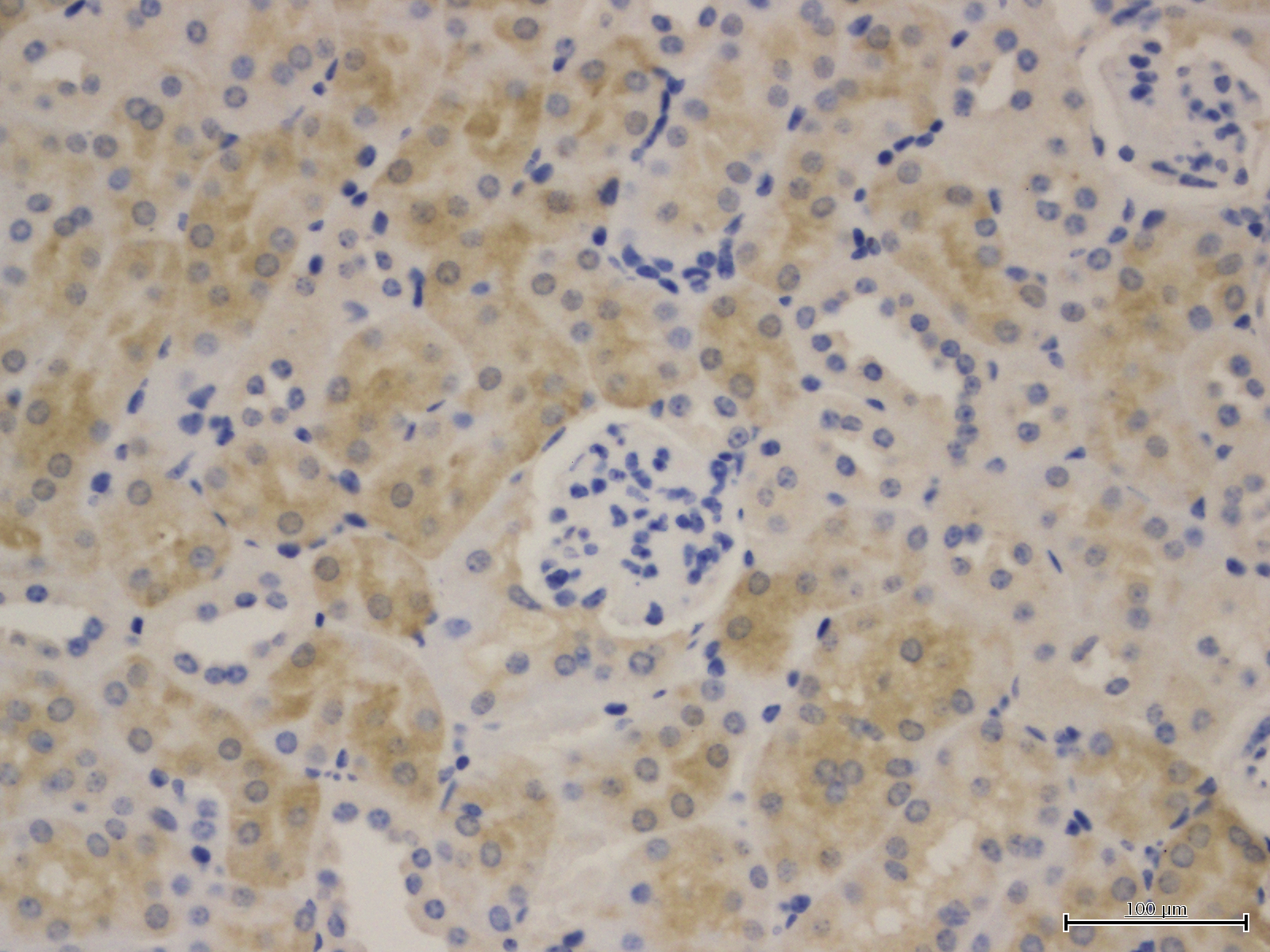

Supplement: S15 File — (ZIP) [file pone.0327042.s015.zip › 50mGy 4w Con-1(Used publication).tif]

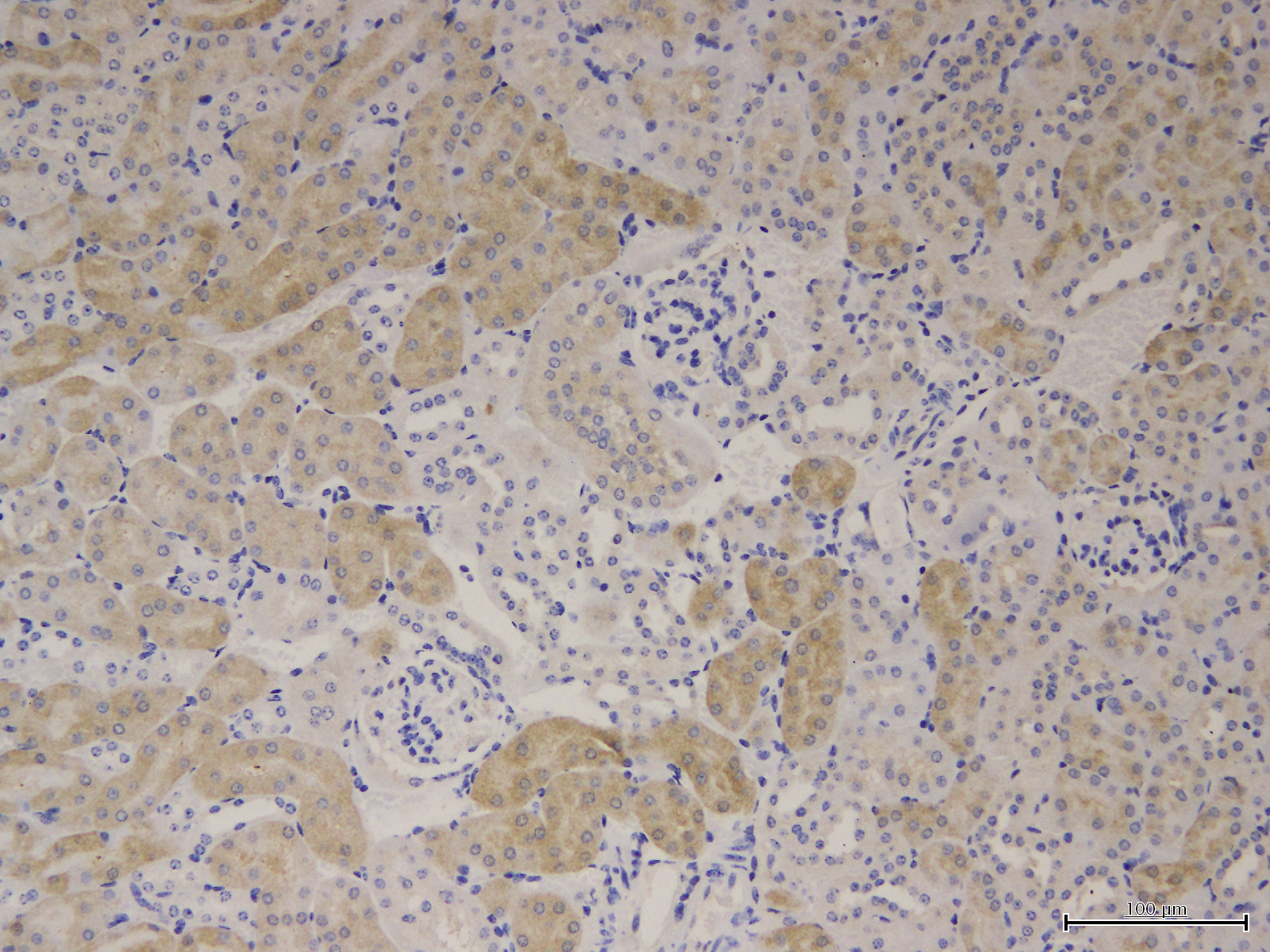

Supplement: S15 File — (ZIP) [file pone.0327042.s015.zip › 50mGy 4w Con-2.tif]

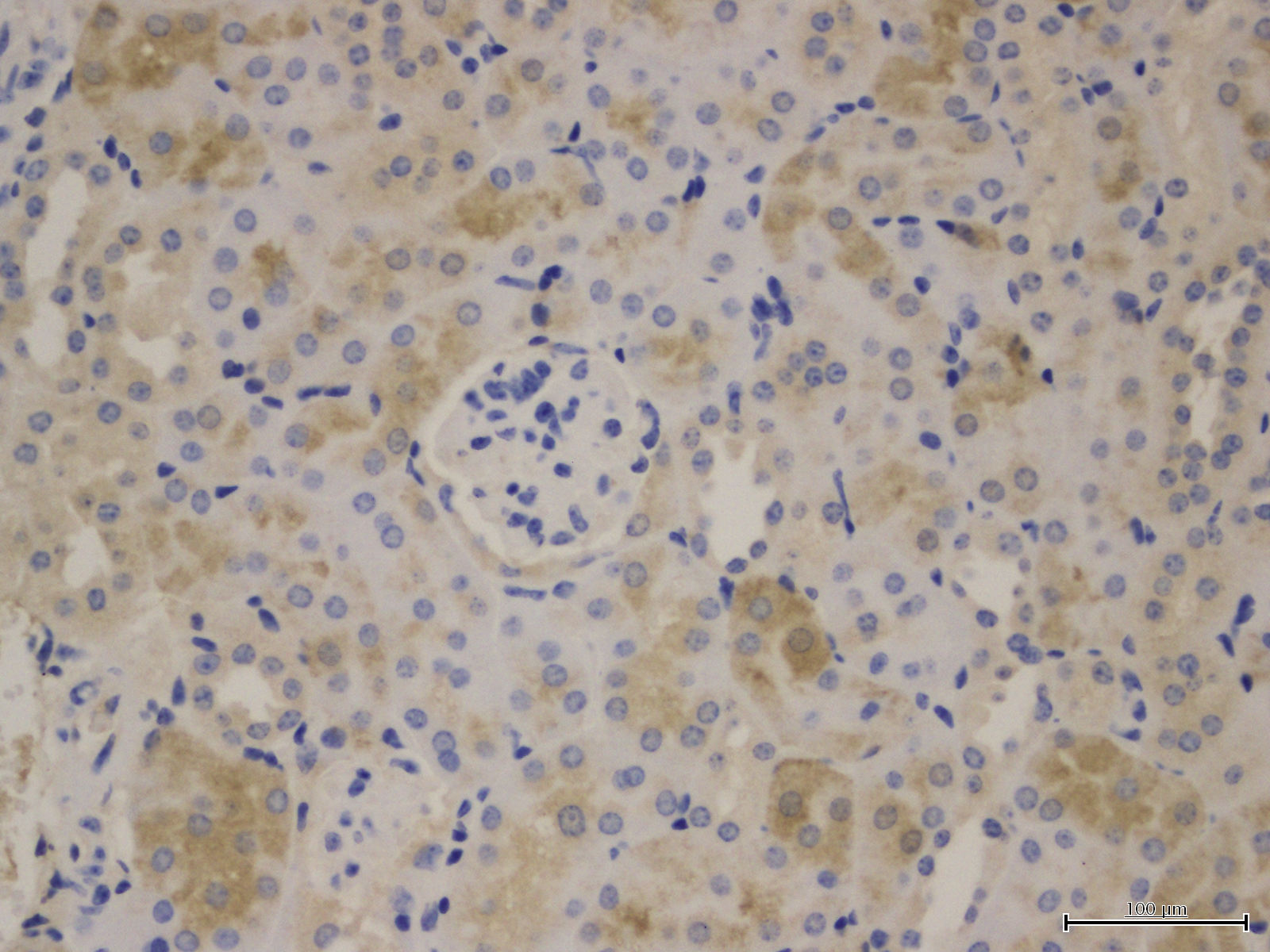

Supplement: S15 File — (ZIP) [file pone.0327042.s015.zip › 50mGy 4w Con-3.tif]

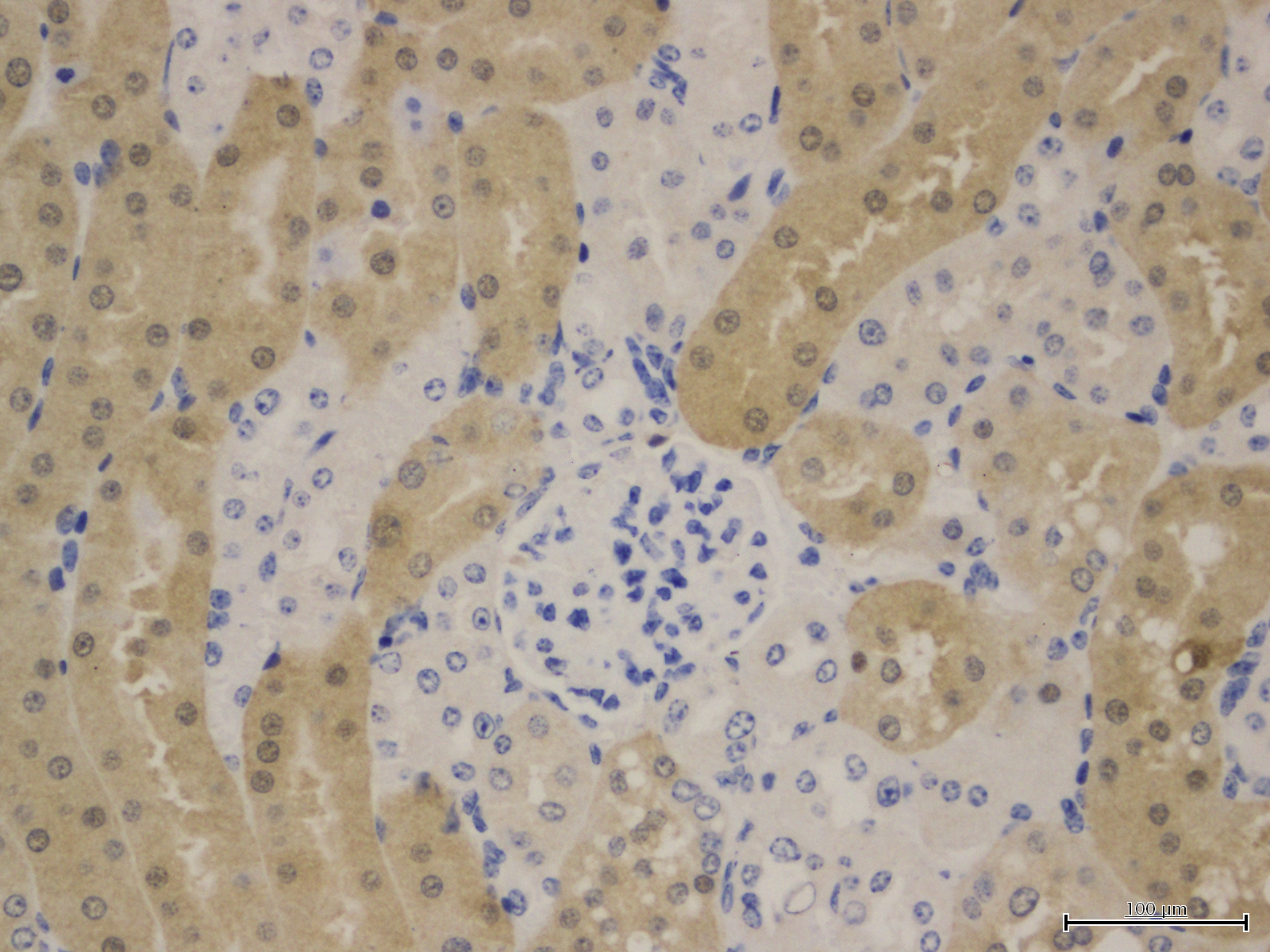

Supplement: S15 File — (ZIP) [file pone.0327042.s015.zip › 50mGy 4w DM-1 (Used publication).tif]

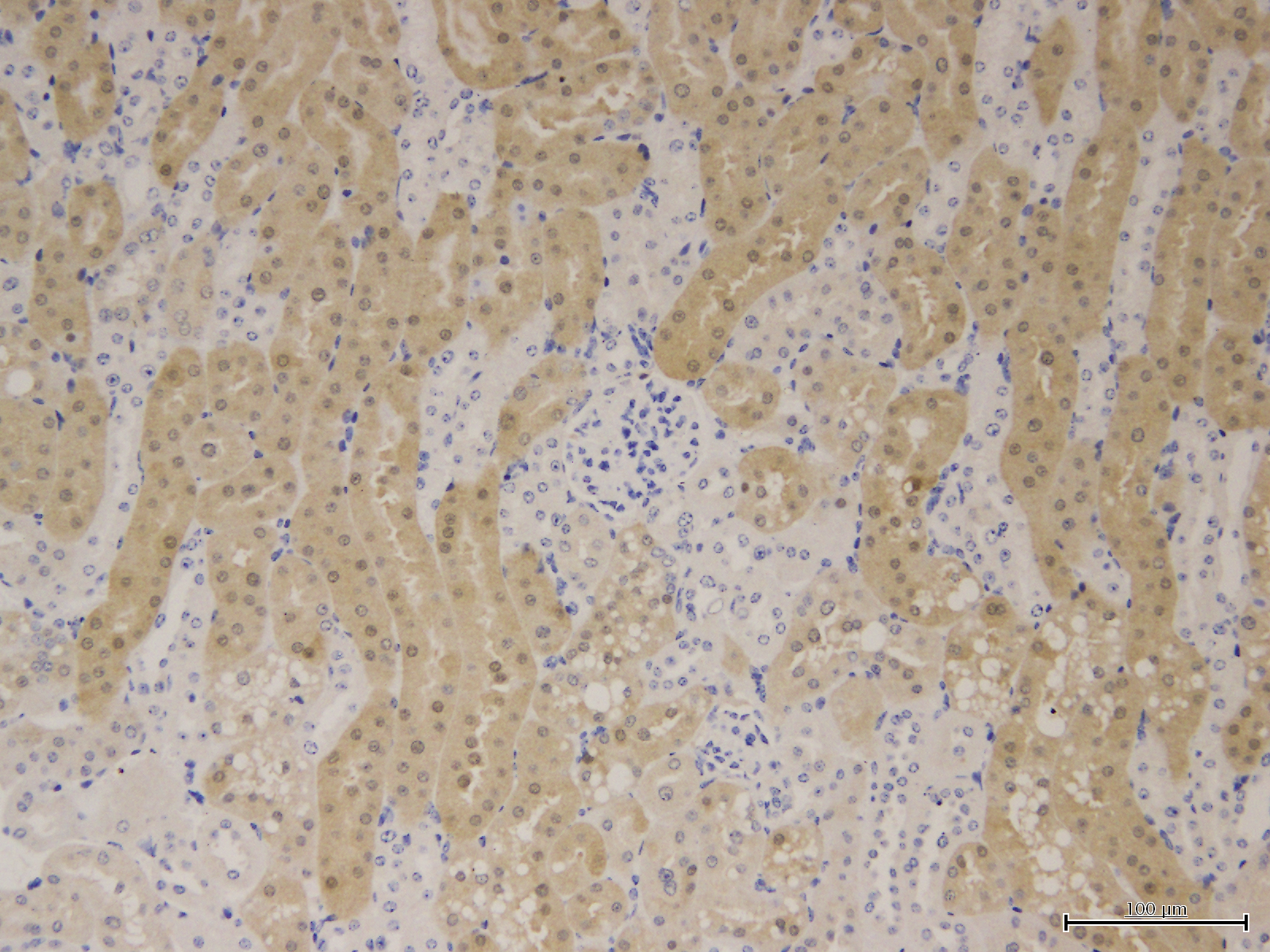

Supplement: S16 File — (ZIP) [file pone.0327042.s016.zip › 50mGy 4w DM-1_2.tif]
