# Supplementary figures and images for: Expression of Concern: Multiple Low-Dose Radiation Prevents Type 2 Diabetes-Induced Renal Damage through Attenuation of Dyslipidemia and Insulin Resistance and Subsequent Renal Inflammation and Oxidative Stress (part 3 of 3)
Source: PLoS One. 2025 Jun 30;20(6):e0327042. doi: 10.1371/journal.pone.0327042 (PMC12208481; doi:10.1371/journal.pone.0327042)

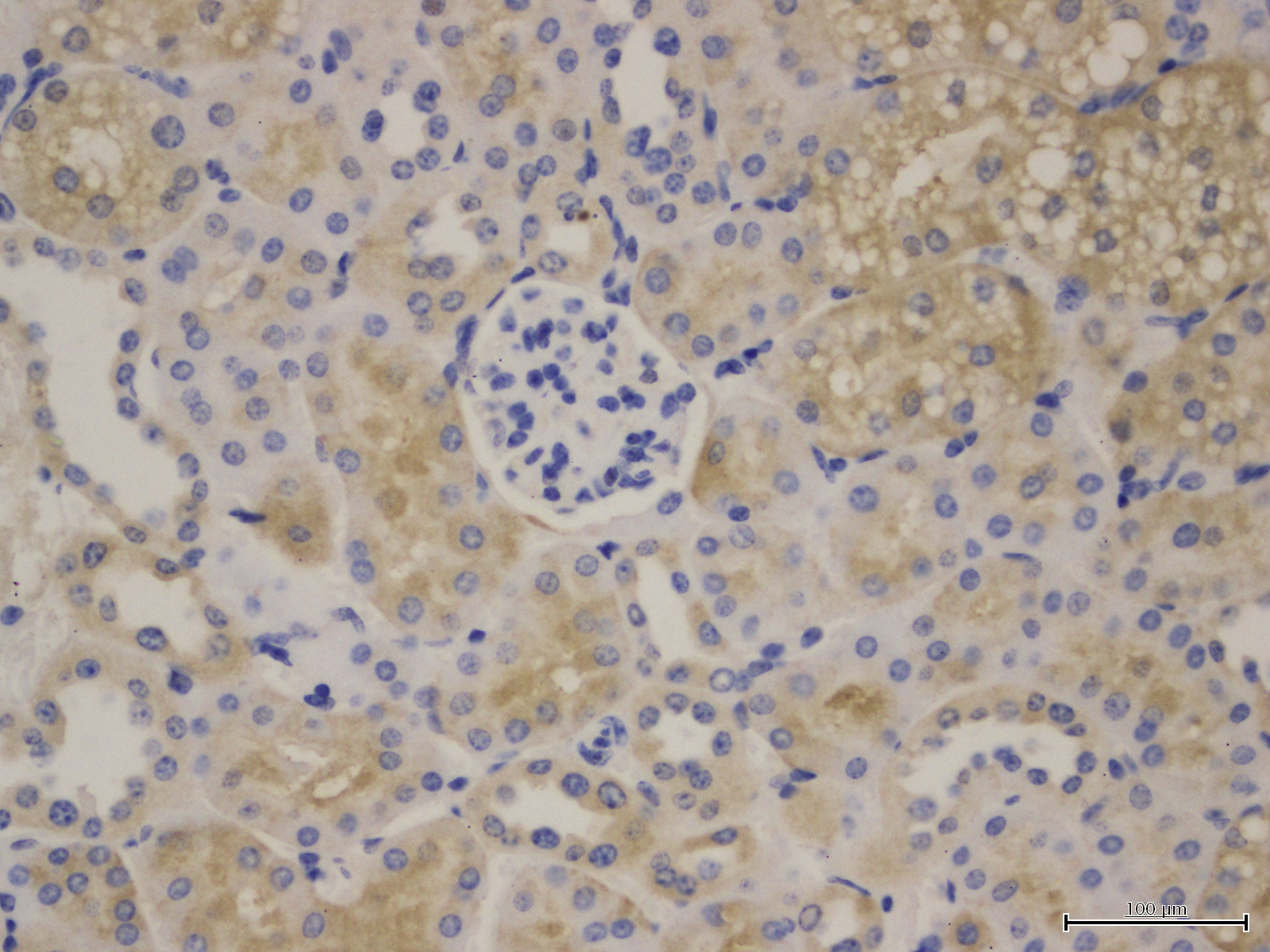

Supplement: S16 File — (ZIP) [file pone.0327042.s016.zip › 50mGy 4w DM-3.tif]

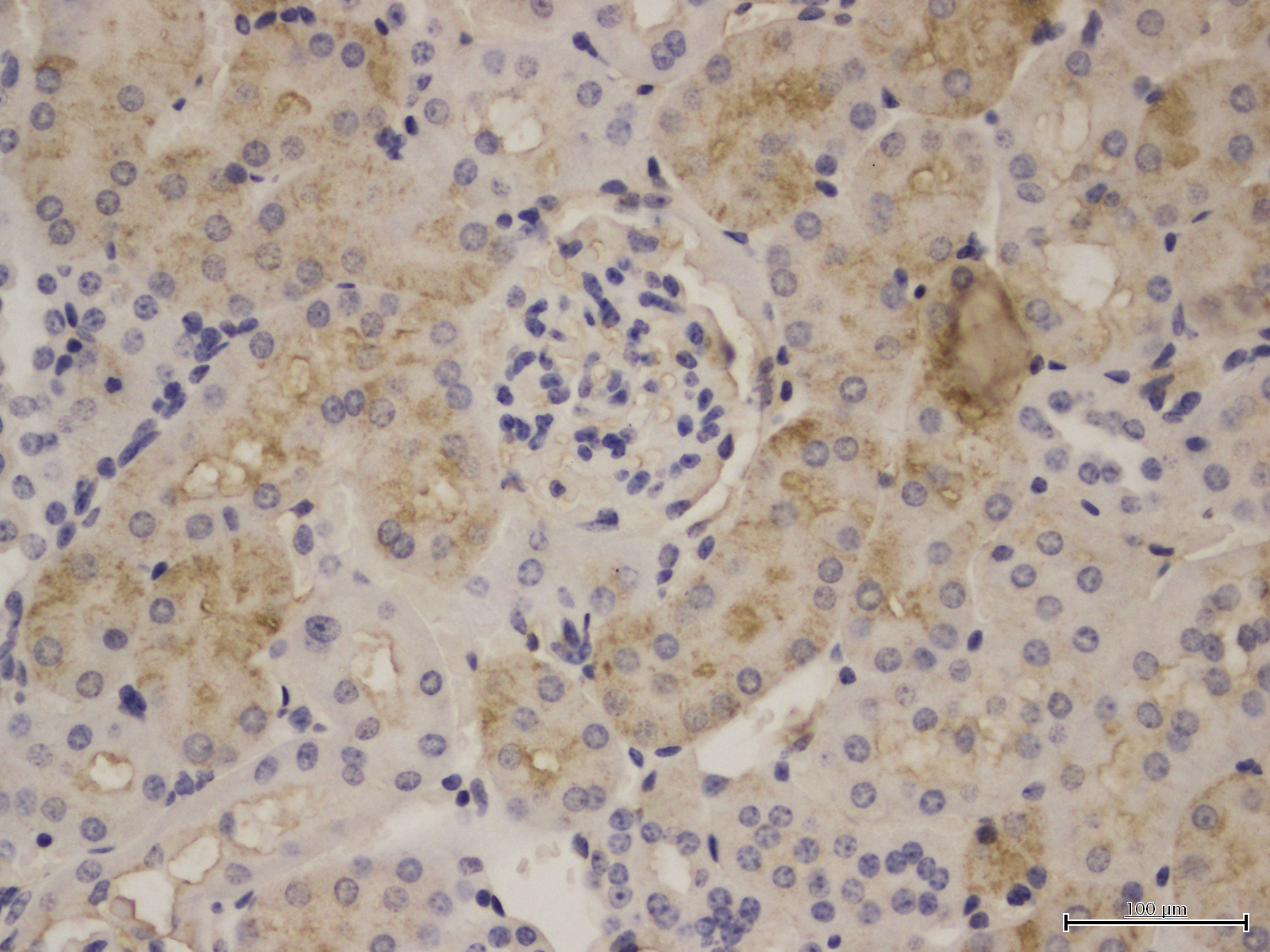

Supplement: S16 File — (ZIP) [file pone.0327042.s016.zip › 50mGy 8w Con-1(Used publication).tif]

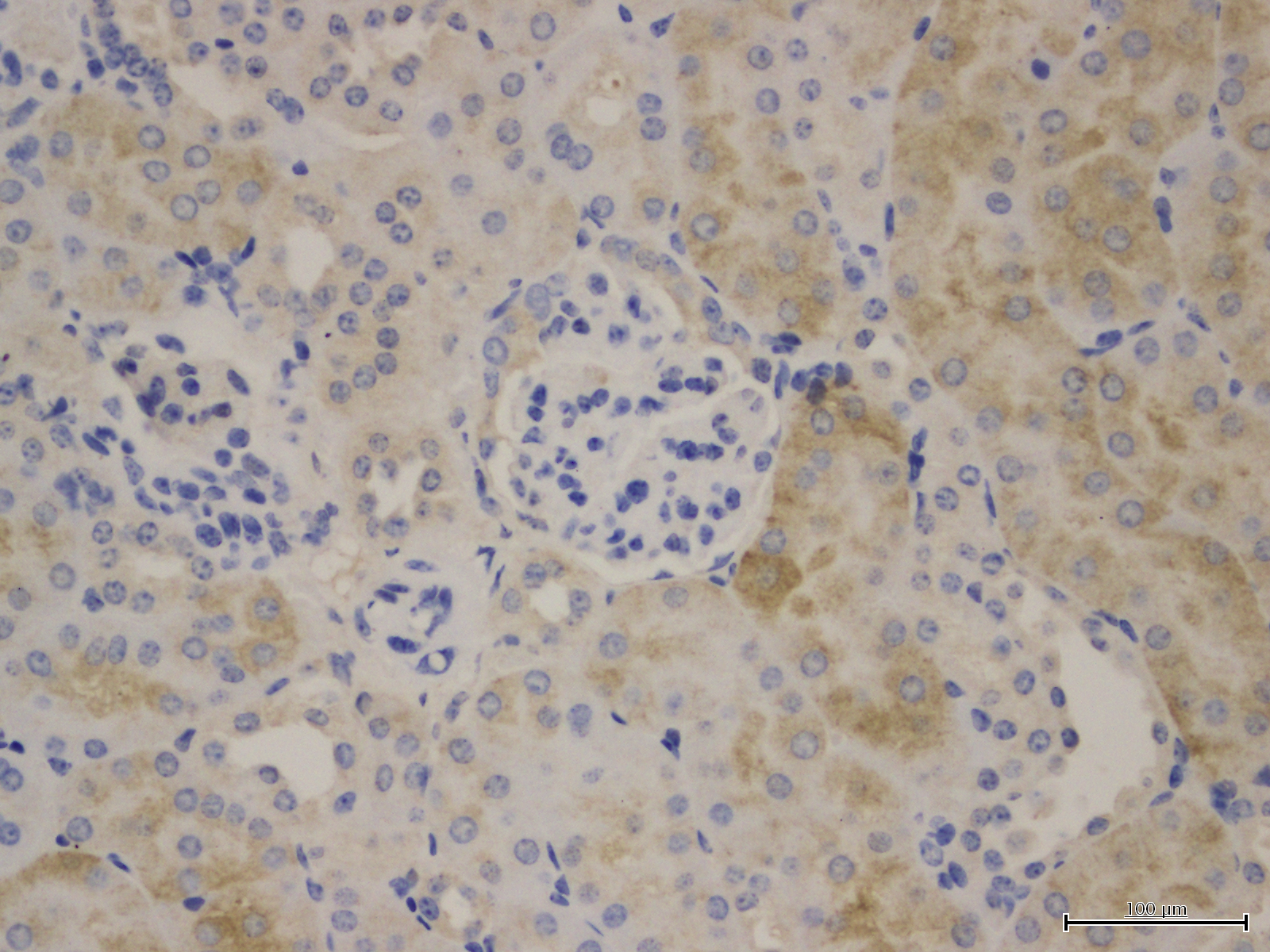

Supplement: S16 File — (ZIP) [file pone.0327042.s016.zip › 50mGy 8w Con-2.tif]

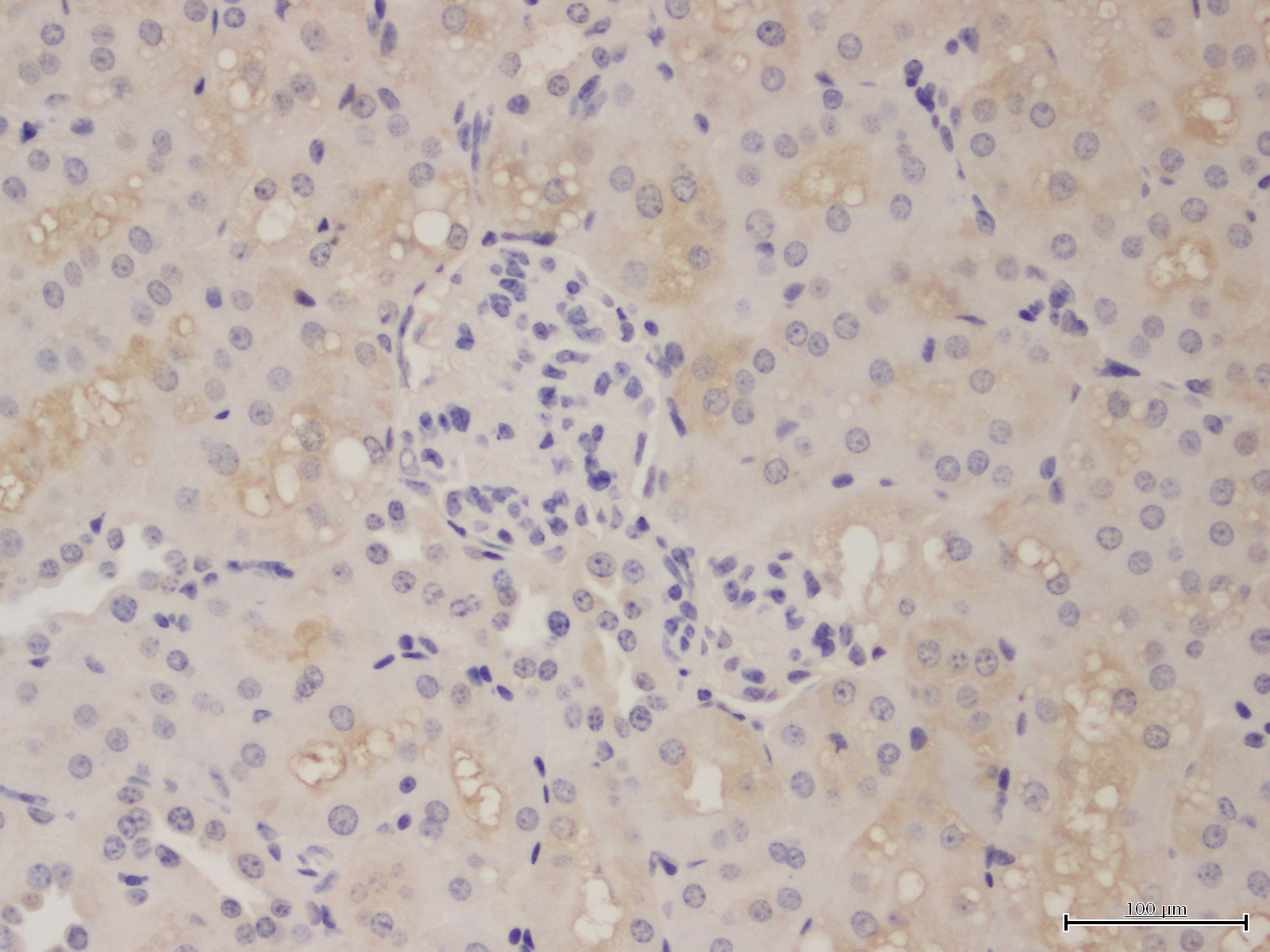

Supplement: S16 File — (ZIP) [file pone.0327042.s016.zip › 50mGy 8w DM-1(Used publication).TIF]

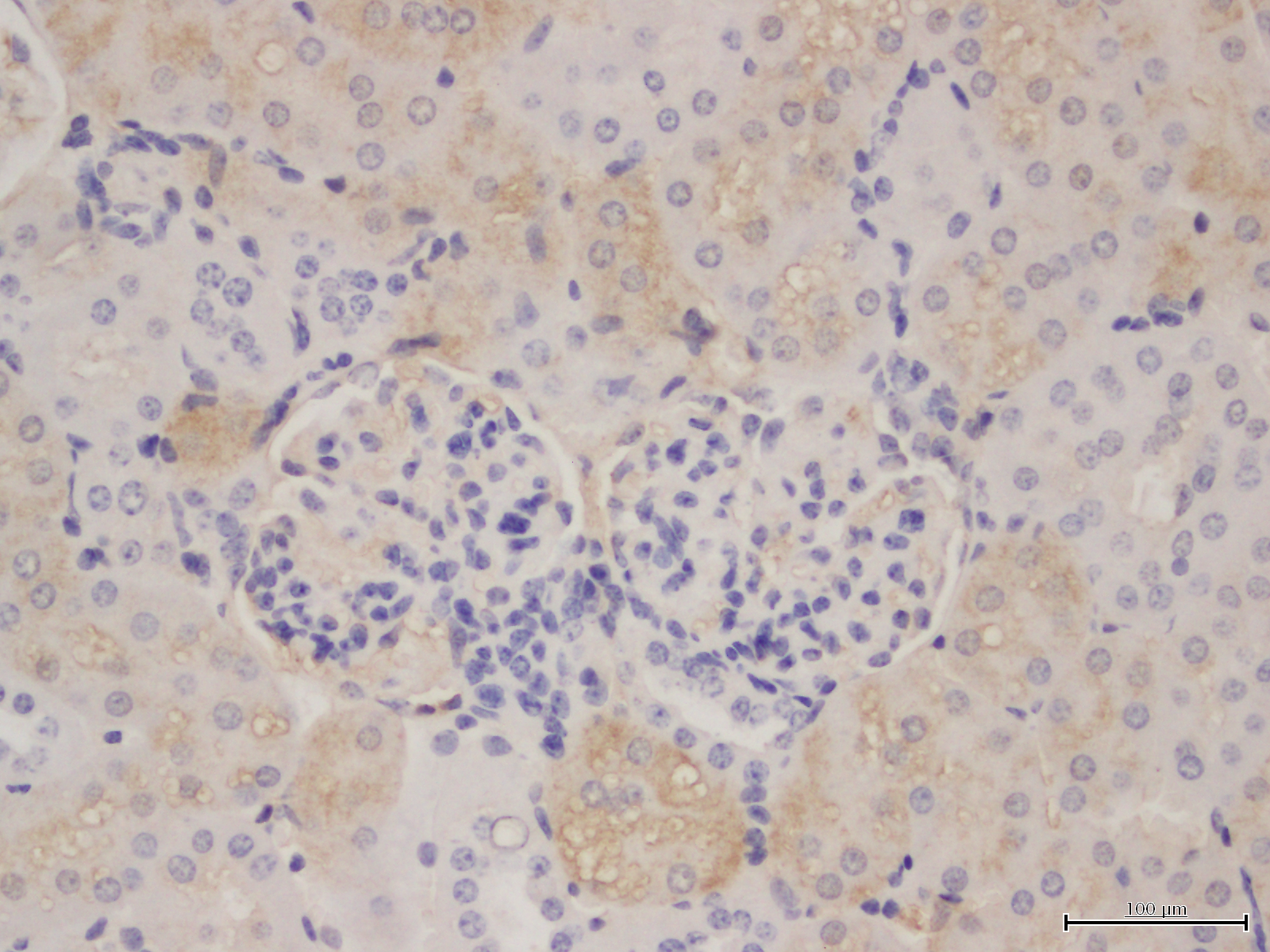

Supplement: S16 File — (ZIP) [file pone.0327042.s016.zip › 50mGy 8w DM-2.TIF]

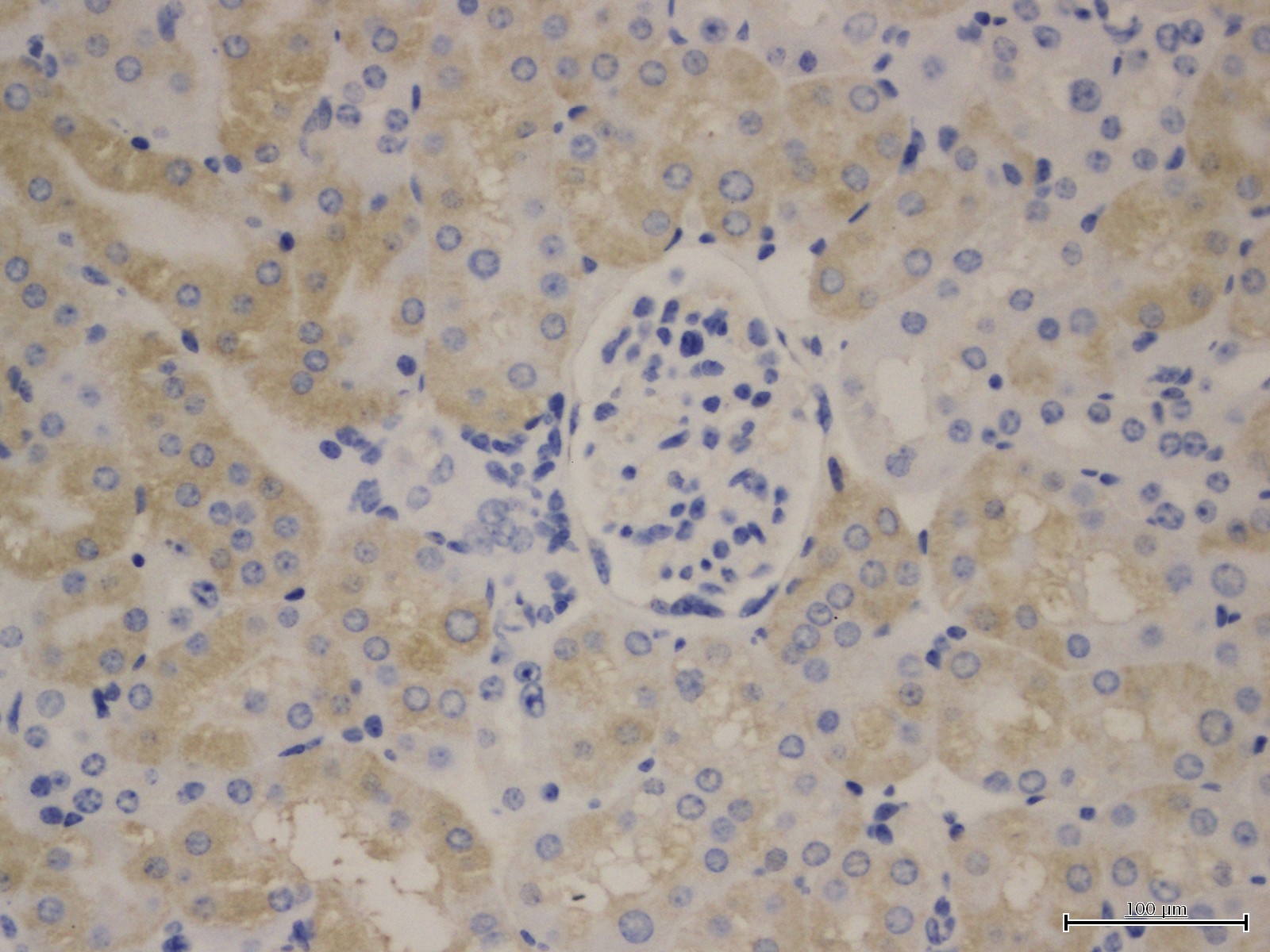

Supplement: S16 File — (ZIP) [file pone.0327042.s016.zip › 75mGy 4w DM-1(used publication but was repeatedly used for 50mGy 4w Con group).tif]

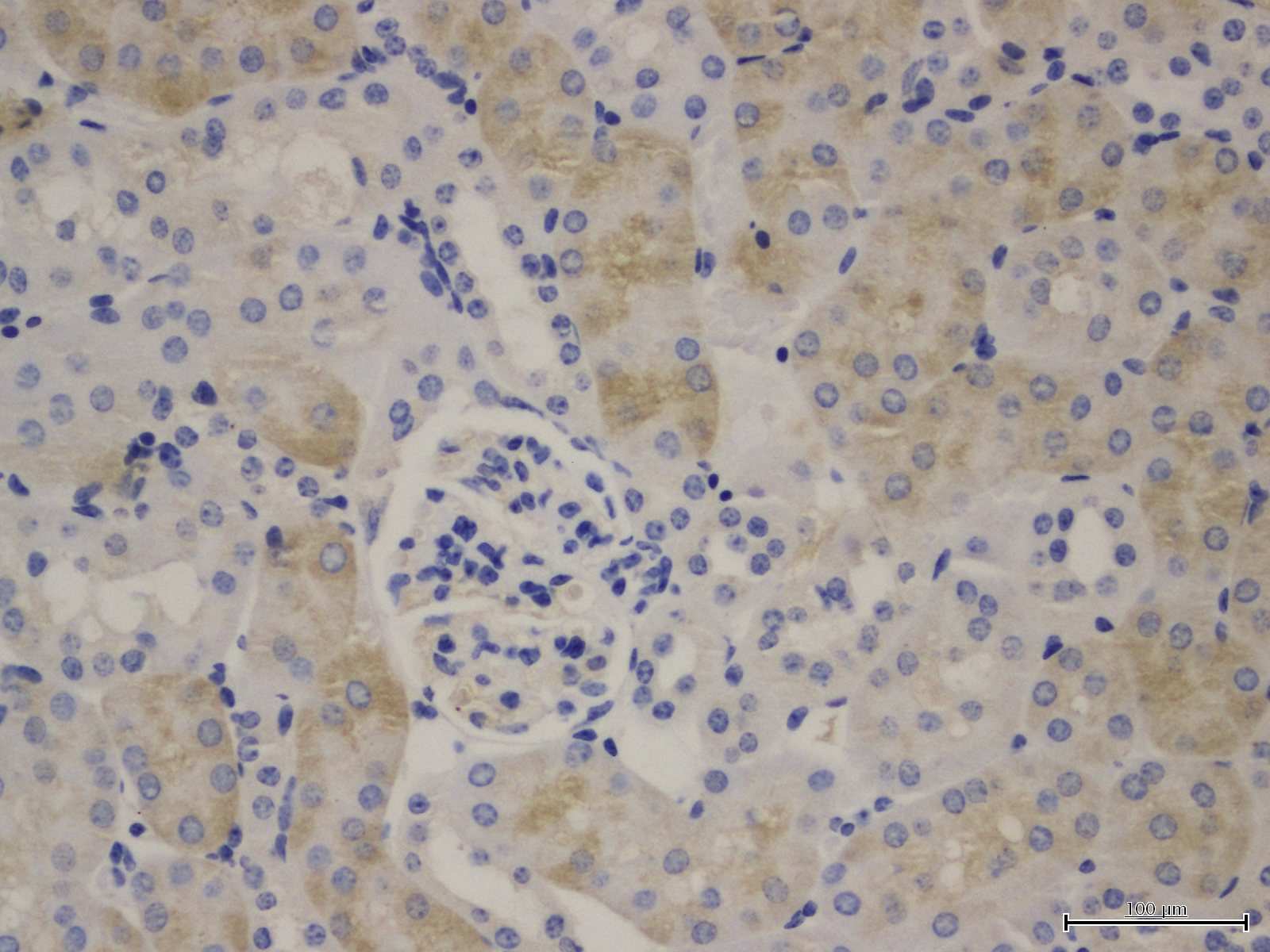

Supplement: S16 File — (ZIP) [file pone.0327042.s016.zip › 75mGy 4w DM-2.tif]

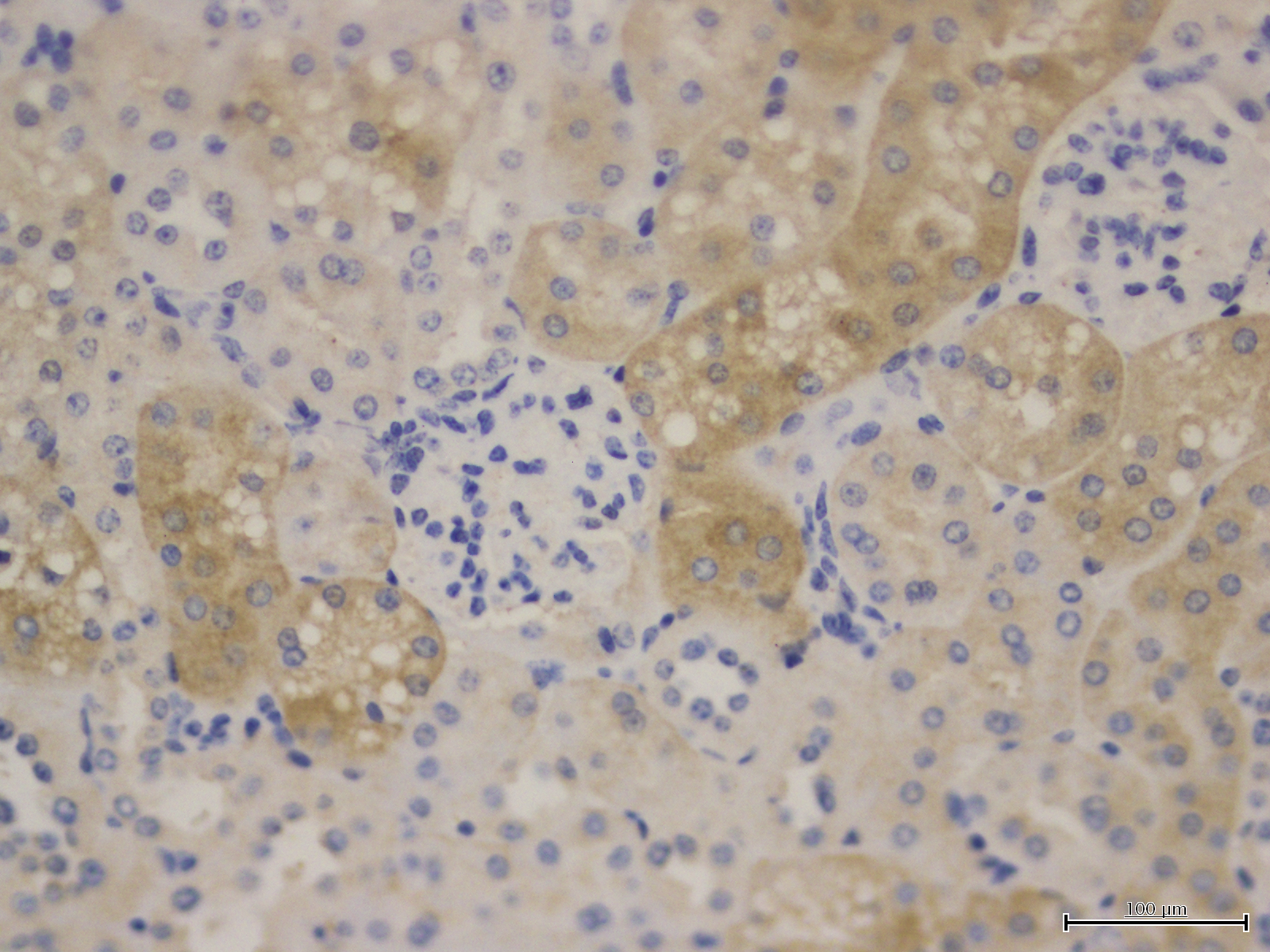

Supplement: S16 File — (ZIP) [file pone.0327042.s016.zip › 75mGy 4w DM-3.tif]

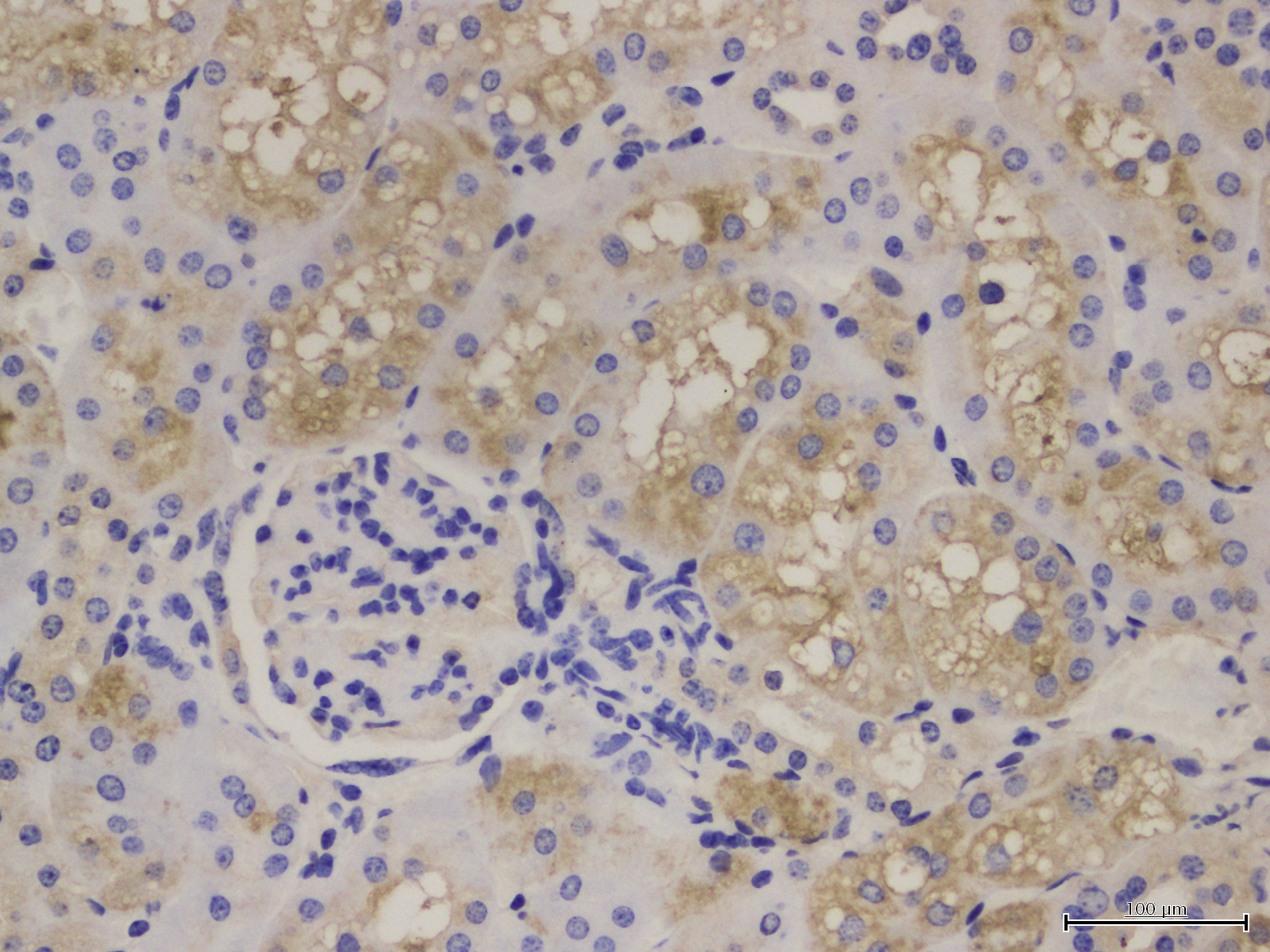

Supplement: S16 File — (ZIP) [file pone.0327042.s016.zip › 75mGy 8w DM-1(Used publication).tif]

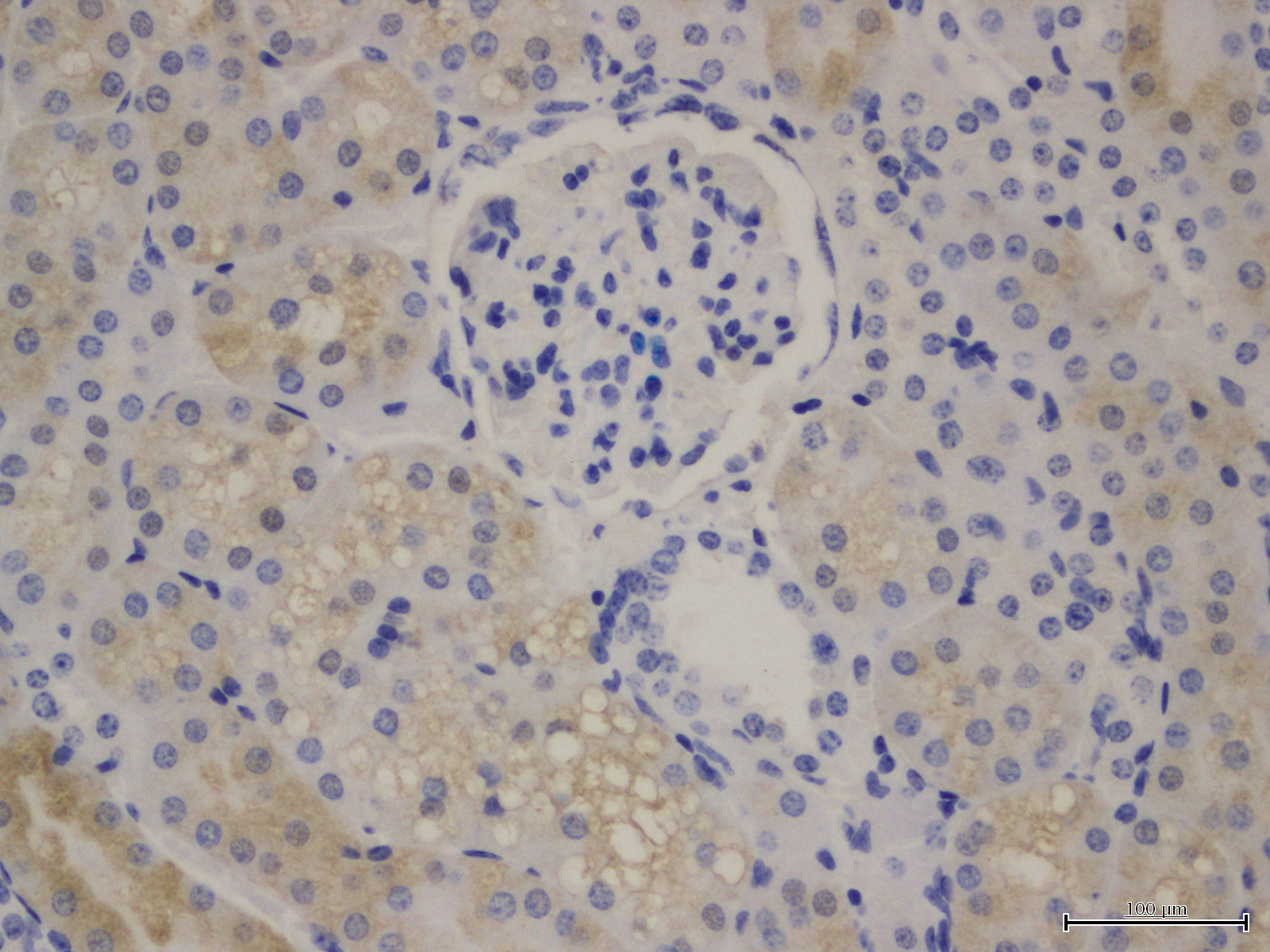

Supplement: S16 File — (ZIP) [file pone.0327042.s016.zip › 75mGy 8w DM-2.tif]

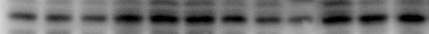

Supplement: S17 File — (ZIP) [file pone.0327042.s017.zip › Fig 9C_ HO1 blot.tif]

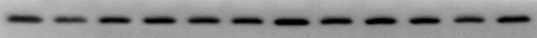

Supplement: S17 File — (ZIP) [file pone.0327042.s017.zip › Fig 9C_ β-actin blot of HO1.tif]

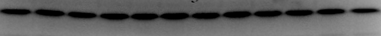

Supplement: S17 File — (ZIP) [file pone.0327042.s017.zip › Fig 9D_ β-actin blot of NQO-1.tif]

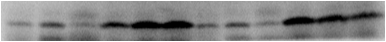

Supplement: S17 File — (ZIP) [file pone.0327042.s017.zip › Fig 9D_NQO-1 blot.tif]
